# Synthetic PS-III Glycoconjugate Vaccines Based on Solid-Phase Assembled Well-Defined Oligosaccharides Confers Protection against *Clostridium difficile*

**DOI:** 10.1021/acscentsci.6c00855

**Published:** 2026-08-15

**Authors:** Yiting Chen, Xiao Liu, Taotao Zhang, Zhen Wang, Yang Li, Jing Zeng, Hanrui Li, Chengli Zong, Qiang Liu

**Affiliations:** † 47879 Guangzhou University of Chinese Medicine, Guangdong 510006, China; ‡ 727956 Zhongshan Institute for Drug Discovery, Zhongshan 528400, China; § School of Pharmaceutical Sciences, Key Laboratory of Tropical Biological Resources of Ministry of Education, 74629 Hainan University, Haikou 570228, China; ∥ State Key Laboratory of Discovery and Utilization of Functional Components in Traditional Chinese Medicine, School of Pharmaceutical Sciences, 74628 Guizhou Medical University, Guiyang 561113, China; ⊥ Carbohydrate-Based Drug Research Center, 58298 Shanghai Institute of Materia Medica, Chinese Academy of Sciences, Shanghai 201203, China; # Department of Blood Transfusion, Zhongnan Hospital of Wuhan University, Wuhan 430071, China; ∇ University of Chinese Academy of Sciences, Beijing 100049, China

## Abstract

*Clostridium difficile* (*C. difficile*), a Gram-positive bacterium responsible for life-threatening diarrhea, causes approximately 12 800 deaths annually in the United States, yet no licensed vaccine is currently available. PS-III, a cell-surface glycan composed of 6,6′-phosphodiester-linked di-*N*-acetylglucosamine (di-GlcNAc) repeats, has been identified as a promising vaccine antigen because of its potent immunogenicity. However, the lack of efficient synthetic method limited the availability of structurally defined PS-III antigens, hampering systematic immunological analysis. Herein, we report a solid-phase synthesis strategy employing Merrifield resin that enables efficient assembly of PS-III oligosaccharides up to a tridecasaccharide—the largest well-defined structure synthesized up to date. Three CRM197-conjugated vaccines bearing precisely defined glycotopes ranging from pentasaccharide to tridecasaccharide were constructed and evaluated in both in vitro and in vivo immunization studies. Glycan microarray profiling of sera from immunized mice revealed glycotope length-dependent IgG/IgM responses, with the nonasaccharide (**1d-CRM197**) and tridecasaccharide conjugate (**1f-CRM197**) eliciting the strongest binding. In a murine challenge model, most glycoconjugate vaccines conferred superior protection against *C. difficile* compared with inactivated whole cell bacteria vaccine. Notably, **1f-CRM197** induced a nearly complete bacterial killing in the opsonophagocytic assay. Collectively, we have established an efficient solid-phase synthesis for well-defined *C. difficile* PS-III glycans. The resulting CRM197-conjugate vaccines with short-to-long glycans showed potent activity in opsonophagocytic and murine models, paving the way for preclinical development of *C. difficile* glycoconjugate vaccines.

## Graphical abstract

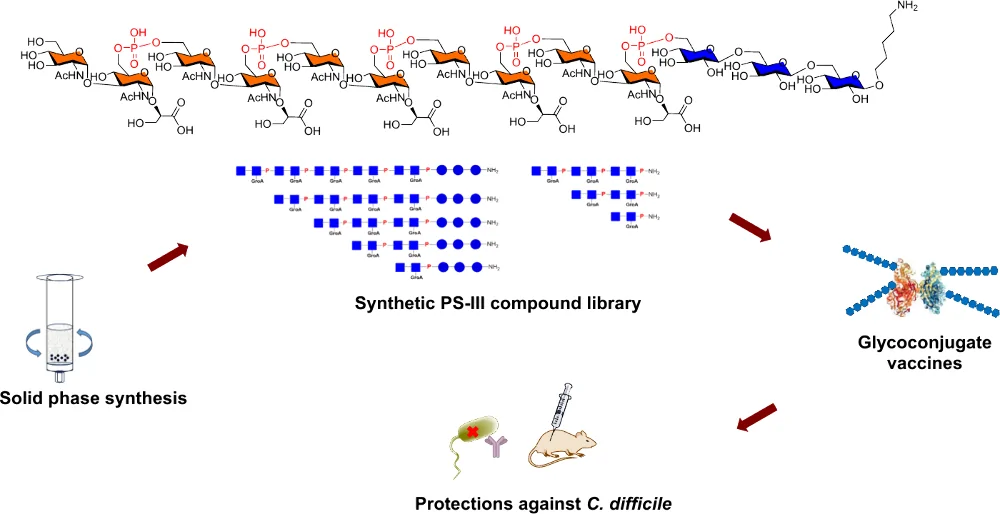

## Introduction

*Clostridium difficile* is a pathogenic gastrointestinal bacterium responsible for *C. difficile* infections (CDI) causing life-threatening diarrhea primarily in individuals over 65 years of age worldwide with high patient mortality and morbidity.^1,2^ In the United States, CDI accounts for over 200 000 infections and approximately 12 800 deaths annually, designated an “urgent” public health threat in 2019 by CDC.^3^ Despite the availability of antibiotics, biotherapeutics, and fecal microbiota transplantation, no vaccine is currently available to prevent infection.^4–7^ Toxin-based vaccines can alleviate disease severity but do not prevent *C. difficile* colonization or transmission. The recent failure of a bivalent toxoid vaccine in a phase III trial further emphasizes the urgent need to explore carbohydrate-based vaccine strategies.^8–10^ The bacterial glycan capsule, particularly capsular polysaccharides (CPS), represents an attractive target for glycoconjugate vaccine design,^11,12^ as exemplified by the success of *Streptococcus pneumoniae* polysaccharide conjugate vaccines.^13,14^ Notably, a pioneering synthetic glycoconjugate vaccine candidate against *C. difficile*, based on PS-II, has recently advanced to Phase I clinical trials for the prevention of CDI. Among the three surface polysaccharides (PS-I, PS-II, and PS-III) of *C. difficile*, PS-III (also called lipoteichoic acid of *C. difficile*; see Figure 1a) is broadly conserved across clinical isolates and has emerged as one of the most promising candidates for glycoconjugate vaccine development against CDI.^15–19^

**Figure 1:**
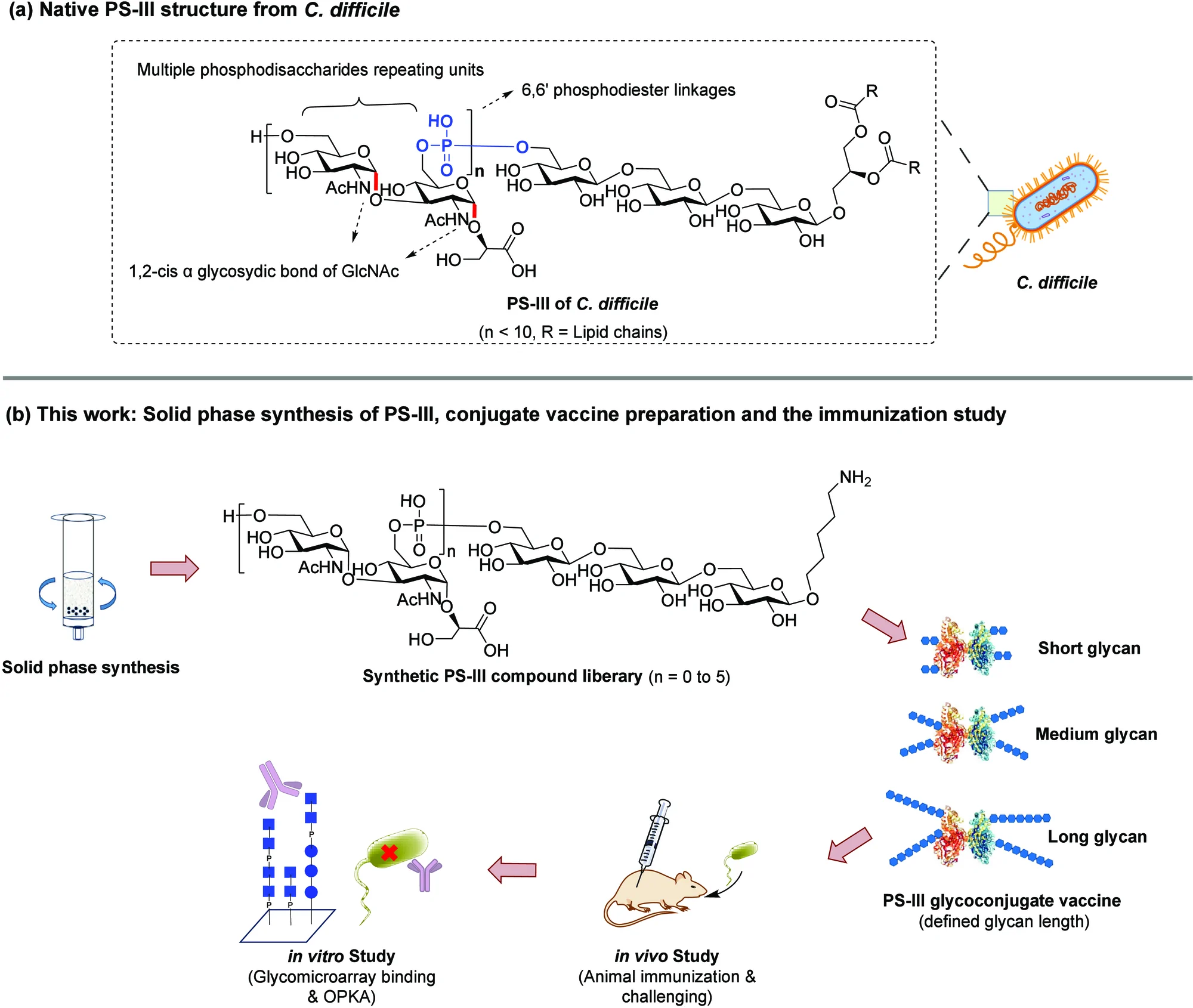
(a) Native structure of PS-III from *C. difficile* and its main synthetic challenges. (b) PS-III compound library construction via solid phase synthesis, synthetic conjugate vaccines preparation and the immunization study of this work. Figure is generated with ChemDraw software and BioGDP.com.

Due to the difficulties in isolating and purifying PS-III from fermentation,^16^ chemically synthesized, structurally defined PS-III serves as a valuable tool for probing the structure–immunogenicity relationship of *C. difficile* glycans and for advancing synthetic glycoconjugate vaccine development.^17,19–21^ However, the synthesis of PS-III remains highly challenging. Its repeating unit comprises a rare 6,6′-phosphodiester-linked GlcNAc-α-(1→3)-GlcNAc disaccharide bearing a glyceric acid substituent (Figure 1a).^16^ The polymer consists of 1 to 10 such phosphodiester-linked units attached to a triglucose anchor that is embedded into the bacterial membrane via a glycerolipid chain at the reducing end.^21^ Accessing such a phosphoglycan is notoriously difficult due to its polyanionic character, high polarity, and the demanding orthogonal protecting group manipulations required. Furthermore, the presence of two 1,2-*cis*-α GlcNAc glycosidic linkages within each repeating unit imposes an additional synthetic hurdle in the construction of PS-III.

In 2014, Pedersen^21^ achieved the total synthesis of a PS-III tridecasaccharide using solution-phase phosphoramidite chemistry. However, the final material contained inseparable shorter oligomers as a result of purification challenges associated with phosphoglycans. Gu^22^ and co-workers later prepared PS-III fragments containing two phosphodisaccharide repeats using an H-phosphonate strategy. More recently, Seeberger’s group^19,23^ synthesized PS-III oligosaccharides up to two repeats and demonstrated the immunogenicity of the conjugate vaccines in mice. These studies established the chemical accessibility and vaccine potential of PS-III. However, solution-phase routes remain constrained by low yields, lengthy steps, and product contamination with short, inseparable oligomers.^21^ To enable systematic investigation of PS-III as a *C. difficile* glycoconjugate vaccine candidate, a robust and scalable synthesis of structurally defined PS-III is urgently needed. Importantly, glycotopes exceeding three repeating units are critical for antibody recognition,^24^ yet no synthetic PS-III conjugates beyond dimer have been described. This represents a major gap, since longer oligosaccharides (>10 monosaccharides) frequently elicit superior immune responses,^25,26^ as shown in the synthetic conjugate vaccines of *Shigella* (SF2a-TT15, in clinical trials)^27,28^ and *Haemophilus influenzae* type B (Quimi-Hib).^29^

Solid-phase synthesis offers distinct advantages for assembling phosphoglycans, including eliminating intermediate isolation, shortening reaction times, and improving yields—proven in bacterial capsular polysaccharide and teichoic acid synthesis.^30–32^ We envisioned that the PS-III backbone could be efficiently constructed through a universal solid-phase strategy, wherein the β-1,6-triglucose motif and phosphodisaccharide repeats are sequentially introduced via on-resin glycosylation and iterative phosphoramidite (P­[III]) chemistry. The robustness of P­[III] coupling on solid support has been validated in our previous syntheses of pyrophosphate-containing biomolecules^33–36^ and microbial phosphoglycans by others.^37,38^ Here, using a Merrifield resin equipped with a photocleavable linker,^39,40^ we achieved the most comprehensive PS-III library to date—up to a tridecasaccharide (five repeating units; see Figure 1b)—through stereoselective construction of the β-1,6-triglucose anchor followed by P­[III]-mediated assembly of 6,6′-linked phosphodisaccharides. Notably, the immunological role of the 1,6-triglucose moiety in the PS-III conjugate vaccine remains unexplored, as prior work has focused on the phospho-di-GlcNAc repeats.^17,19,23^ Our library of full-length PS-III glycans therefore provides a powerful toolkit for future investigations. The purified defined PS-III fragments were conjugated to CRM197 and evaluated by in vivo animal immunization and bacteria challenge studies, in vitro glycan microarray and opsonophagocytic killing assays (OPKA), demonstrating the efficacy of PS-III conjugate vaccines with precisely controlled glycotope length (Figure 1b).

## Results and Discussion

### Retrosynthesis and Key Phosphoramidite Synthesis

Targeting a series of PS-III glycans (**1**, *n* = 0 to 5) with varying repeat lengths, we aimed to systematically evaluate the impact of phosphoglycan chain length on immunological activity. Each compound was designed to include an aminopentyl linker at the reducing end to facilitate conjugation to carrier proteins or immobilization on microarrays, a 1,6-linked triglucose moiety, and a backbone of 6,6′-linked phosphodisaccharide repeats (phospho-di-GlcNAc). Retrosynthetically (Scheme 1), we envisioned assembling the phosphodiester linkages via a solid-phase strategy using phosphoramidite **2f** as the key P­(III) donor and resin-bound acceptor **3**. The solid support was based on a photocleavable linker-functionalized Merrifield resin **8** that reported by the Seeberger group,^41,42^ chosen for its mild cleavage profile and proven compatibility with oligosaccharide synthesis.^42^ Assembly of resin **3** was achieved through iterative glycosylation of resin **8** with glucose donor **7**. Meanwhile, the pivotal disaccharide phosphoramidite **2f** could be efficiently constructed via sequential α-selective glycosylations of glyceric acid acceptor **6** with 2-azido-2-deoxy glucosyl donors **5** and **4**.

**Scheme 1:**
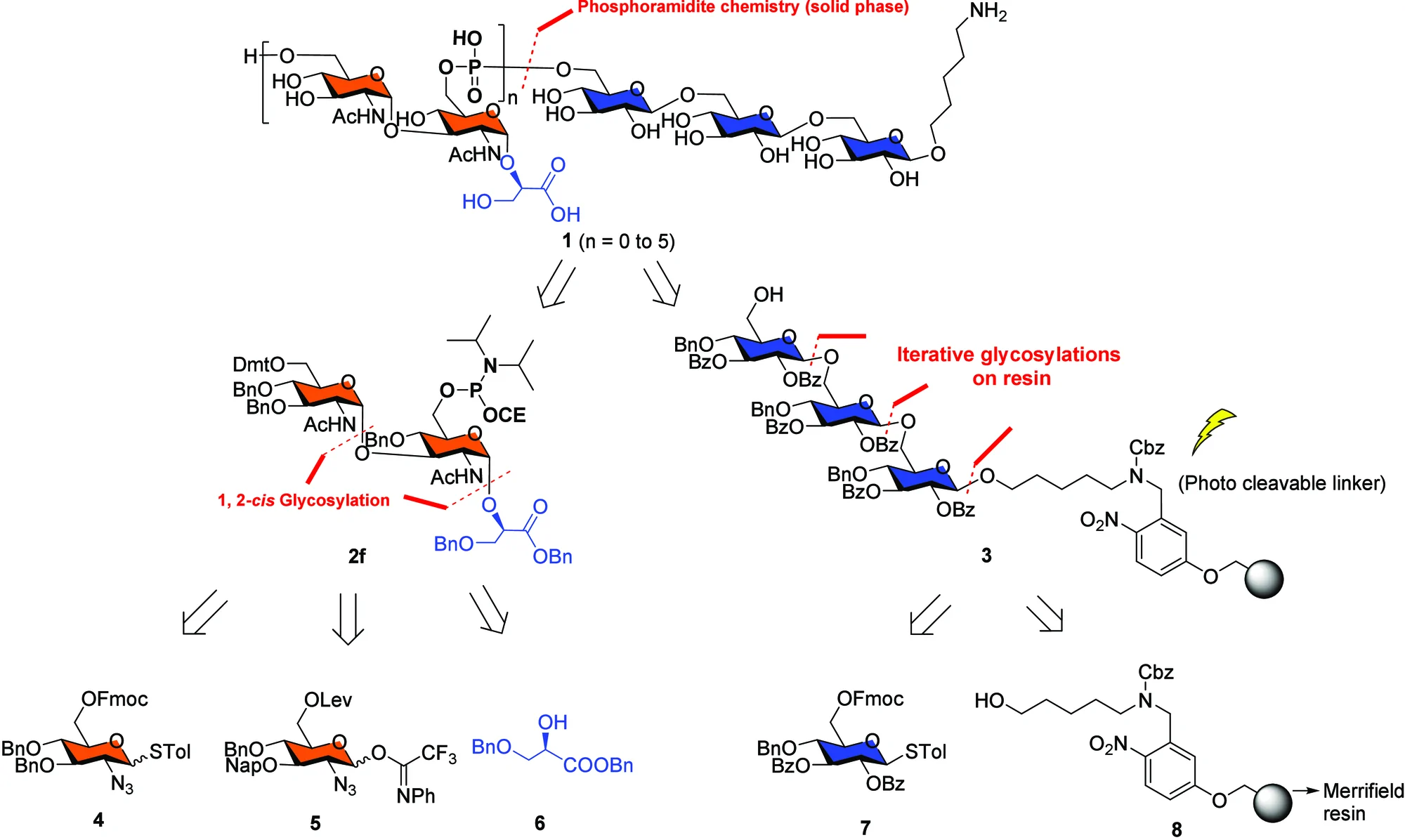
Retrosynthetic Analysis of PS-III (**1**)­^a^

a Legend: Fmoc, 9-fluorenylmethyloxycarbonyl; Bn, benzyl; STol, *p*-tolylthio; Nap, 2-naphthylmethyl; Lev, levulinyl; Ph, phenyl; Bz, benzoyl; Cbz, benzyloxycarbonyl; Dmt, 4,4′-dimethoxytrityl; CE, 2-cyanoethyl.

The synthesis of the key building block, phosphoramidite **2f**, is outlined in Scheme 2. Commercially available 2-deoxyl-2-azido-peracetylated glucose **9** (see the Supporting Information (SI)) was first reacted with *p*-toluenethiol (TolSH), followed by deacetylation under NaOMe to afford **10**. Protection of the 4,6-diol as a benzylidene acetal yielded the free 3-OH compound **11**, which was subsequently protected with a 1-naphthylmethyl (Nap) group to furnish fully protected **12**. Selective cleavage of the benzylidene acetal with dichloro­(phenyl)­borane (PhBCl_2_) generated primary alcohol **13**, which was levulinoylated to give **14**. Direct glycosylation of thiodonor **14** with acceptor **6** gave low yields; therefore, **14** was converted to imidate donor **5** via *N*-iodosuccinimide (NIS)-mediated oxidative activation, followed by treatment with 2,2,2-trifluoro-*N*-phenylacetimidoyl chloride, providing **5** in high yield.^43^ Glycosylation of **5** with glycerate acceptor **6** afforded **16** in good yield (76%) and excellent α-selectivity. Subsequent 2,3-dichloro-5,6-dicyano-1,4-benzoquinone (DDQ)-mediated Nap deprotection under aqueous buffer conditions gave acceptor **17**. Glycosylation of the 2-deoxyl-2-azido glucose donor **4** (prepared from **11** in three steps; see Scheme S2 in the SI) with acceptor **17** produced the key disaccharide **18** in moderate yield (49%) and good α-stereoselectivity. Two azido groups in **18** were reduced with Zn and acetylated in situ with acetic anhydride (Ac_2_O) to furnish **19**.^44^ Fmoc deprotection under basic conditions gave **20**, whose 6-OH was capped with a 4,4′-dimethoxytrityl (Dmt) group, a common temporary protecting group in solid-phase synthesis,^31^ to afford **21** in high yield. Subsequent Lev removal furnished **22**, and phosphitylation yielded the key disaccharide phosphoramidite **2f**. The synthesis of **2f** is efficient and readily scalable (1.5 g, 1.2 mmol; see the SI), enabling the preparation of longer PS-III oligomers. Notably, other phosphoramidite analogues bearing alternative protecting groups or unmodified azides exhibited poor stability or low coupling efficiency (Figure S1 in the SI). Thus, **2f** was selected for subsequent solid-phase synthesis.

**Scheme 2:**
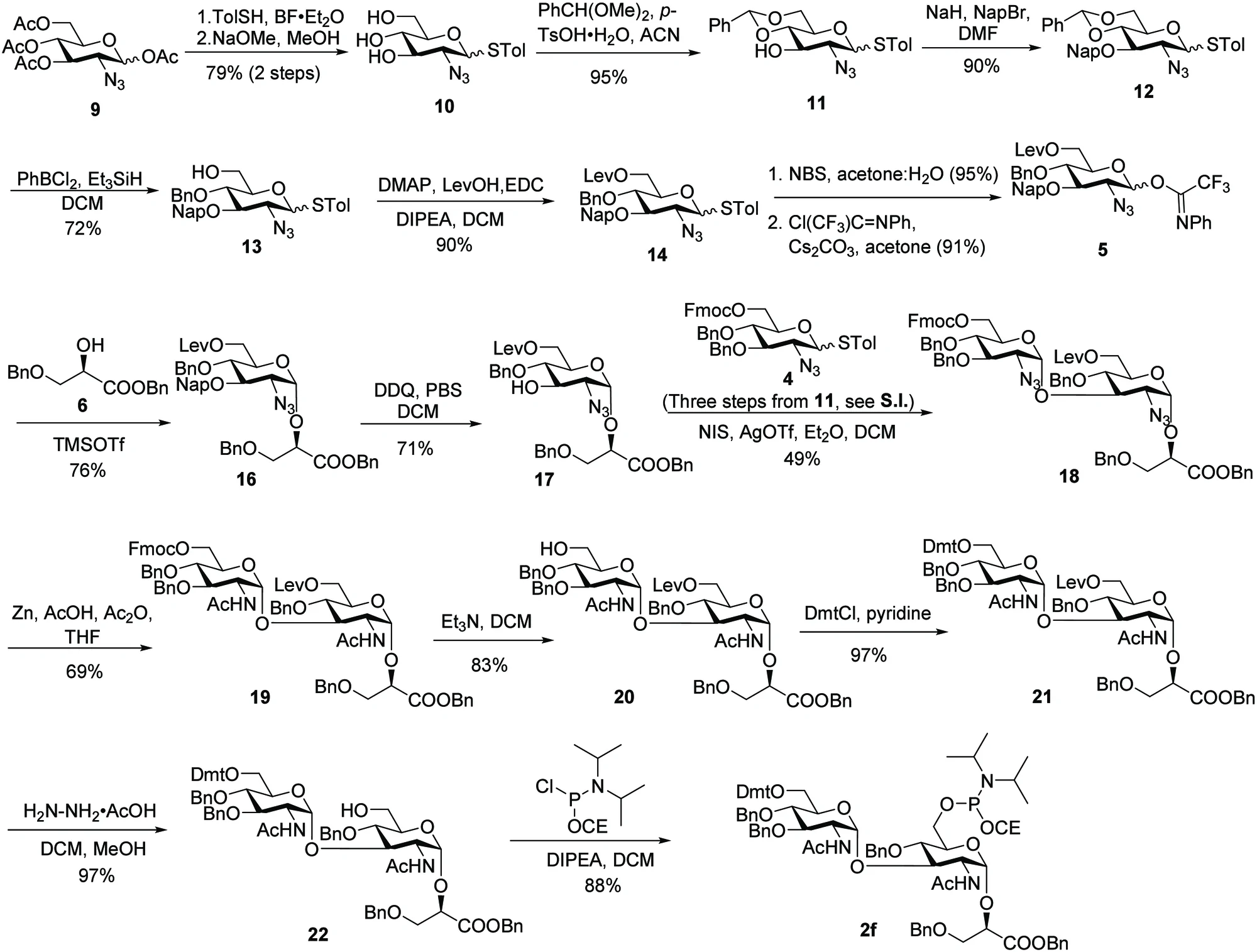
Synthesis of Key Disaccharide Phosphoramidite **2f** ^a^

a Legend: NapBr = 2-(bromomethyl)­naphthalene; DMAP = 4-dimethylaminopyridine; EDC = 1-ethyl-3-(3-dimethylaminopropyl)­carbodiimide; DIPEA = *N*,*N*-diisopropylethylamine; NBS = *N*-bromosuccinimide; DDQ = 2,3-dichloro-5,6-dicyano-1,4-benzoquinone; NIS = *N*-iodosuccinimide; DmtCl = 4,4′-dimethoxytrityl chloride.

### Solid-Phase Synthesis of PS-III Oligosaccharides via Iterative Glycosylation and Phosphoramidite Coupling

With the key phosphoramidite **2f** in hand, we explored its applicability in P­(III)-based chemistry on Merrifield resin—a scarcely reported approach (Scheme 3). Photocleavable linker-functionalized resin **8**, obtained from reported method,^41^ was reacted with **2f** under manual phosphoramidite protocol: Resin **8** was treated with activator 5-(ethylthio)-1H-tetrazole (ETT) and **2f** in a sealed syringe reactor (using shaker for better mixing, see Figure S2 in the SI) to form the P­(III) intermediate after wash, which was then oxidized to P­(V) species using (1*S*)-(+)-(10-camphorsulfonyl)­oxaziridine (CSO).^45^ After capping with Ac_2_O and Dmt deblock with trichloroacetonitrile (TCA), the coupling cycle was finished *n* times (*n* = 1 to 3) to install multiple phosphodisaccharide repeats (**23**). A detailed experimental instruction of manual solid-phase synthesis can be found in the Supporting Information (Schemes S4–S6). Global deprotection with 1,8-diazabicyclo[5.4.0]­undec-7-ene (DBU), UV cleavage and hydrogenolysis treatment afforded target compounds **24a**–**24c**, whose structures were confirmed by liquid chromatography–mass spectroscopy (LC-MS) and nuclear magnetic resonance (NMR).

**Scheme 3:**
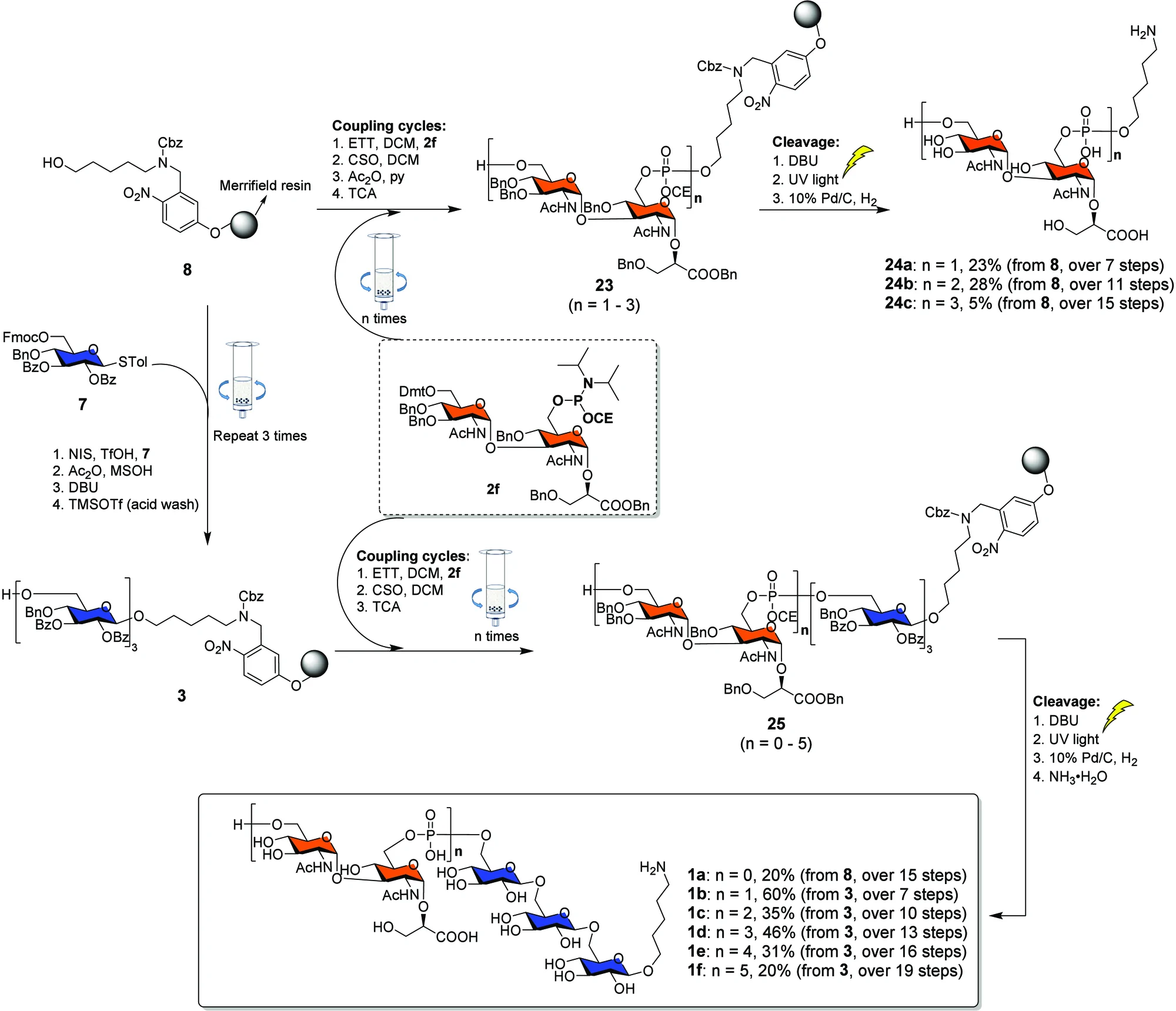
Solid-Phase Synthesis of Well-Defined PS-III Oligosaccharide **24a**–**24c** and **1a**–**1f** ^a^

a Legend: ETT = 5-(ethylthio)-1H-tetrazole; CSO = (1*S*)-(+)-(10-camphorsulfonyl)­oxaziridine; TCA = trichloroacetonitrile; DBU = 1,8-diazabicyclo[5.4.0]­undec-7-ene.

Encouraged by these results, we proceeded to construct the full PS-III antigen featuring a triglucose motif. Thioglycoside donor **7**, bearing a 6-*O*-Fmoc (9-fluorenylmethyloxycarbonyl) group, was reacted with resin **8**. A neighboring 2-*O*-benzoyl group ensured exclusive β-selectivity, consistent with the native configuration. After capping (Ac_2_O) and Fmoc removal (DBU), the 6-OH was exposed for further glycosylation. Repeating this cycle three times yielded triglucose resin **3** with a free 6-OH. Subsequently, the key phosphodisaccharide unit was introduced via the same manual phosphoramidite coupling protocol, iterated up to five times (*n* = 0 to 5) to afford fully protected resin **25**. Final deprotection—DBU treatment, UV cleavage, hydrogenolysis, and alkaline hydrolysis—followed by gel filtration purification, yielded defined-length PS-III oligosaccharides **1a**–**1f** with 0 to 5 phosphodisaccharide repeats. Compared with traditional solution-phase synthesis, our solid-phase approach offers significant advantages in time efficiency, minimal purification, and, most importantly, higher overall yields.

Notably, attempts to automate this process on an oligonucleotide synthesizer resulted in significantly lower yields, likely due to insufficient mixing and the low reactivity of the disaccharide amidite. Nevertheless, our solid-phase approach enabled efficient construction of a comprehensive PS-III glycan library, including the tridecasaccharide **1f**—the largest well-defined PS-III oligomer reported to date—prepared in a time-saving and higher-yielding manner than conventional solution synthesis.^21^ All synthetic PS-III derivatives bearing amino handles are readily applicable to conjugate vaccine preparation and immunological studies.

### PS-III Conjugate Vaccine Preparation

Our synthetic PS-III glycans provide a unique opportunity to design and biologically evaluate a semisynthetic *C. difficile* conjugate vaccine. To systematically investigate structure–immunogenicity relationships, we selected three representative oligosaccharides, the penta-(**1b**), nona-(**1d**), and tridecasaccharide (**1f**)—containing one, three, and five phospho-disaccharide repeats, respectively. Each glycan, equipped with an aminopentyl linker, was activated with bis­(4-nitrophenyl) adipate (PNP) under alkaline condition to afford the corresponding ester intermediates **27b/d/f**. These intermediates were then conjugated to CRM197 in PBS buffer (pH 7.4) at room temperature. CRM197, a nontoxic mutant of diphtheria toxin approved by the U.S. Food and Drug Administration, was selected due to its proven safety and widespread use in licensed conjugate vaccines.^13,32,46^ After purification by centrifugal filtration (10 kDa cut off), the resulting glycoconjugates **1b-CRM197**, **1d-CRM197**, and **1f-CRM197** were obtained (Figure 2a). No free oligosaccharide or endotoxin was detected, and the endotoxin level was found to be below 0.0312 EU/mL by LAL testing. (see the SI, page S38) MALDI-TOF mass spectrometry confirmed glycan loadings (defined as the molar ratio of oligosaccharide to protein) of 5.8, 5.0, and 2.7 for **1b-CRM197**, **1d-CRM197**, and **1f-CRM197**, respectively (see Figure S3).

**Figure 2:**
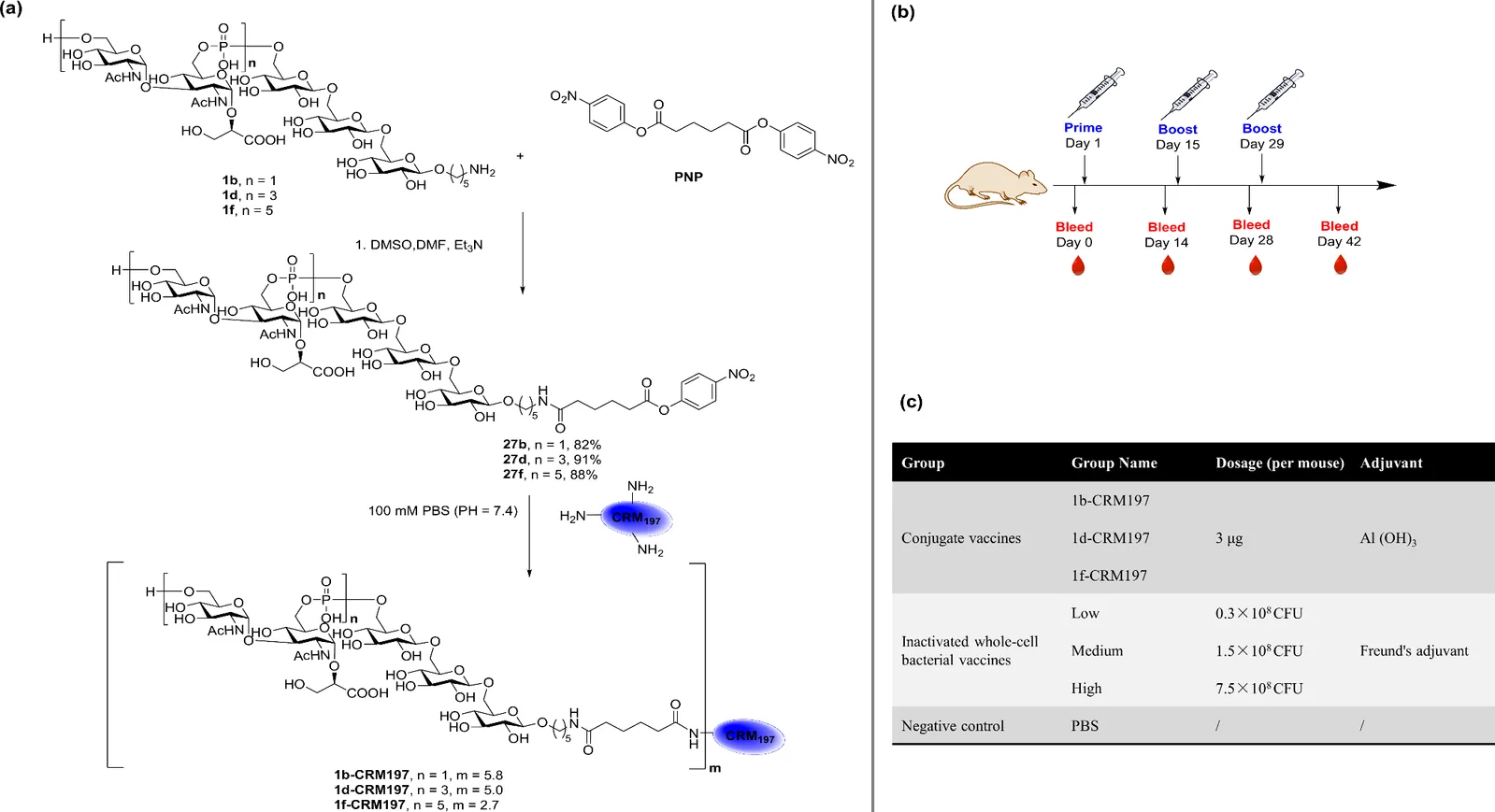
(a) Synthesis of glycoconjugates vaccines **1b/d/f-CRM197**. (b) Immunization schedule. (c) Information of vaccine types, dosage, and adjuvant types. Each group has at least 5 mice.

Mice were immunized with these constructs in a prime–boost regimen (days 1, 15, and 29) and sera were collected on days 0, 14, 28, and 42 (Figure 2b). A formalin-inactivated whole-cell *C. difficile* vaccine was included as a control, administered in low-, medium-, and high-dose regimens (Figure 2c). The immunogenicity of the three glycoconjugates was assessed through in vitro glycan microarray profiling and opsonophagocytic killing assay (OPKA), in vivo immunization, and bacterial challenge study.

### Glycan Microarray Screening of Immunized Animal Sera Reveals a Length-Dependent Binding Pattern

Serum antibody binding to synthetic PS-III glycans (**24a**–**24c** and **1a**–**1f**) was evaluated using a glycan microarray.^47^ Sera collected from all vaccine groups on days 14, 28, and 42 were analyzed, with sera from PBS-treated mice serving as the negative control. Across the different immunization groups, the highest IgG and IgM binding signals were generally observed on day 42, following the final booster immunization (Figure 3a and 3b).

**Figure 3:**
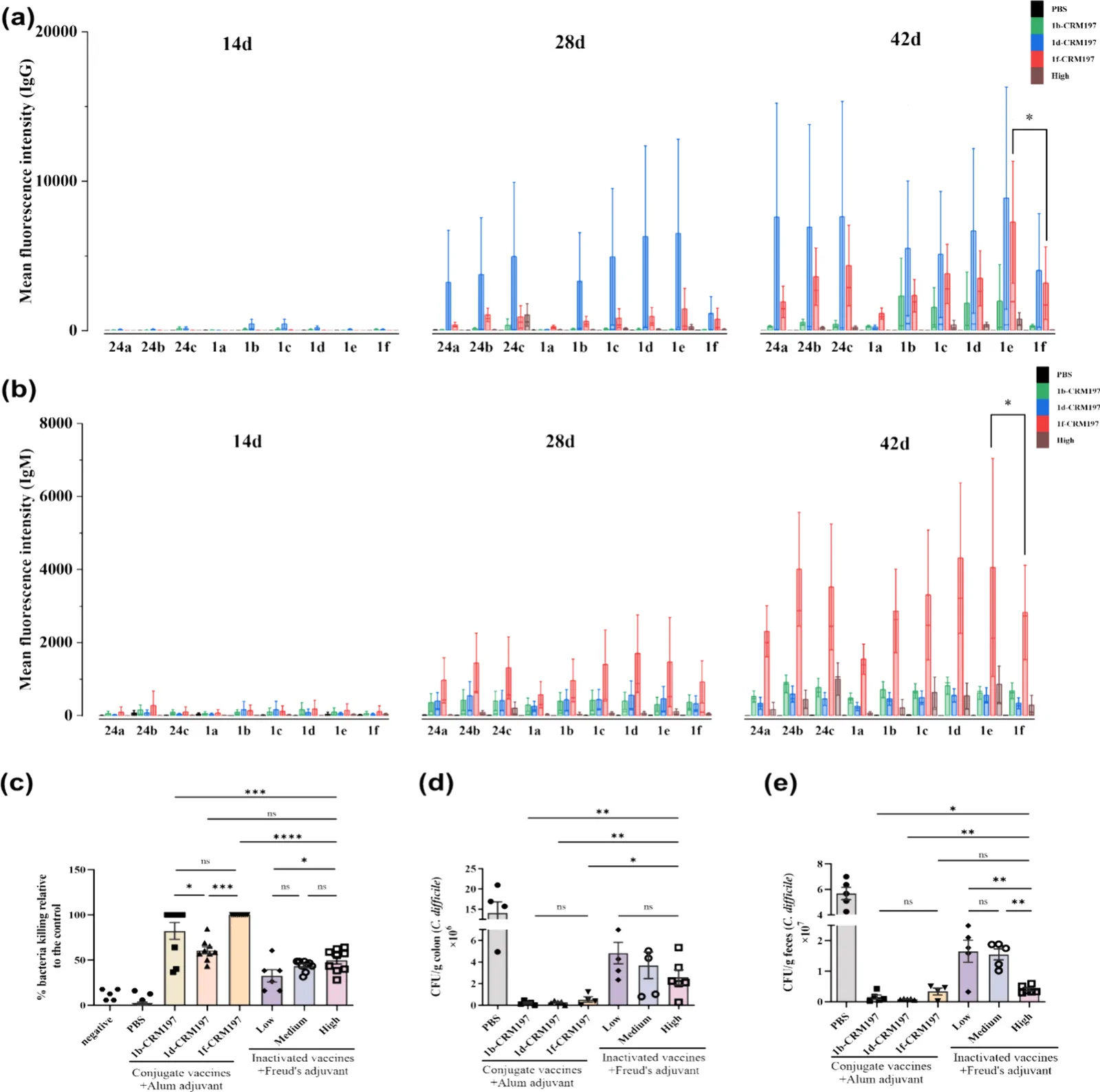
(a) IgG antibody level (serum dilution 1:100) induced by different vaccines or PBS control on day 14, 28, and 42. (b) IgM antibody level (serum dilution 1:100) induced by different vaccines or PBS control on day 14, 28, and 42. (c) In vitro bactericidal experiment by opsonophagocytic killing assays (serum dilution 1:10). Statistical significance was determined by one-way ANOVA with Tukey’s multiple comparisons test. (d) Five days postchallenge colons were analyzed for *C. difficile* by CFU plating assays. (e) Five days post-challenge feces were analyzed for *C. difficile* by CFU plating assay. Note: **PBS** (black); **1b-CRM197** (short-chain glycoconjugate, green); **1d-CRM197** (medium-chain glycoconjugate, blue); **1f-CRM197** (long-chain glycoconjugate, red); **High** (high-dose inactivated whole-cell bacterial vaccine, brown); (*) *p* ≤ 0.05; (**) *p* ≤ 0.01; (***) *p* ≤ 0.001; (****) *p* ≤ 0.0001; ns, not significant.

As shown in Figure 3a, mice immunized with **1d-CRM197** (blue) or **1f-CRM197** (red) developed substantial IgG responses on days 28 and 42, compared to the PBS control. In contrast, **1b-CRM197**, which contained the shortest glycotope (green), and the inactivated whole-cell *C. difficile* vaccine (brown) induced markedly weaker IgG responses. Notably, sera elicited by **1d-CRM197** showed the strongest overall binding to the panel of synthetic PS-III glycans, compared with sera elicited by the shorter glycoconjugates or the whole-cell vaccine. Whereas for IgM responses, **1f-CRM197** elicited the strongest overall IgM binding among the vaccine groups (Figure 3b).

With respect to the immobilized glycan probes, serum antibody binding generally increased with the number of PS-III repeating units, and stronger signals were observed for glycans containing at least one phosphodisaccharide repeating unit, including **24a**–**24c** and **1d**–**1e**, consistent with previous reports.^19,23^ In contrast, all immune sera showed relatively weak binding to triglucose motif **1a**, suggesting that this moiety alone may not constitute the dominant antibody-recognized glycotope. However, additional in vivo studies are required to determine its immunogenic contribution.

A nonmonotonic recognition pattern was observed for the longest glycans. At day 42, sera elicited by **1f-CRM197** showed lower IgG binding to immobilized **1f** than to **1e** (mean MFI 56% lower, *P* < 0.05). This observation suggests that **1e** may already provide an epitope of sufficient length for optimal recognition by antibodies elicited by the long-chain conjugate, whereas the additional phosphodisaccharide repeat in **1f** may not provide further productive antibody contacts and may instead alter the local density, surface orientation, or epitope accessibility of the immobilized glycan. Because microarray MFI is influenced by antigen immobilization and surface presentation and does not directly measure antibody affinity, the molecular basis of this nonmonotonic binding pattern requires further structural and biophysical investigation.

### Glycoconjugate Vaccines Confer Superior Protection Against *C. difficile* Challenge and Enhance Opsonophagocytic Killing of *C. difficile*

Following immunization, mice were subjected to antibiotic-mediated gut microbiota depletion and subsequently challenged orally with 5 × 10^7^ CFU of *C. difficile*. On day 5 post-challenge, colon contents and fecal pellets were collected for bacterial quantification. Compared with whole-cell vaccines, in general glycoconjugate-vaccinated groups conferred superior defense, exhibiting significantly reduced bacterial loads in both colon and fecal samples (Figure 3d and 3e).

To further evaluate the functional activity of vaccine-induced antibodies, an OPKA was performed using differentiated HL-60 cells as standardized neutrophil-like effector cells and sera collected at 42 days (Figure 3c). Differentiated HL-60 cells can acquire neutrophil-like properties to mediate antibody- and complement-dependent opsonophagocytic killing, while reducing the donor-to-donor variability associated with primary neutrophils. *C. difficile* was opsonized with immune sera and cocultured with HL-60 cells to assess phagocytosis-mediated bacterial killing. Sera from the whole-cell vaccine group promoted moderate phagocytosis, whereas sera from all three glycoconjugate groups—**1b-CRM197**, **1d-CRM197**, and **1f-CRM197**—significantly enhanced bacterial clearance. Notably, the **1f-CRM197** group achieved nearly complete (100%) bacterial killing (Figure 3c).

Together, these findings demonstrate that the synthetic glycoconjugates elicit robust functional immune responses, as evidenced by strong in vivo protection and potent opsonophagocytic activity. The relationship between the glycan chain length and immunogenicity appears to be complex rather than simply linear. In general, longer synthetic PS-III fragments may better mimic the native polysaccharide architecture and provide more complete or repetitive epitopes, thereby enhancing B-cell recognition and increasing the overall glycan-binding antibody responses. This is consistent with the glycan microarray results, where sera raised against longer glycotopes in general showed stronger binding to synthetic PS-III glycans (Figure 3a and 3b). The OPKA results also supported the superior functional activity of **1f-CRM197** (Figure 3c). However, the functional activity did not strictly parallel the overall microarray-binding intensity. Notably, sera raised against the shorter **1b-CRM197** conjugate showed relatively weaker microarray binding signals than those of **1d-CRM197** but still displayed strong bactericidal activity (Figure 3c). This apparent discrepancy suggests that the magnitude of glycan-binding antibody responses does not necessarily predict functional opsonophagocytic activity. The glycan microarray detects antibody binding to immobilized synthetic PS-III fragments, whereas OPKA measures the ability of antibodies to recognize native bacterial surface antigens and, more importantly, promote complement-dependent phagocytic killing. Therefore, antibodies elicited by **1b-CRM197** may target a functionally relevant epitope that is accessible on the bacterial surface, despite producing lower overall binding signals on the array. Conversely, longer or intermediate glycotopes may induce measurable binding responses without necessarily generating proportionally stronger bactericidal activity. These findings highlight the importance of functional assays in evaluating carbohydrate-based vaccine candidates and suggest that antibody quality, epitope specificity, and complement-mediated effector function are critical determinants of protection, in addition to antibody titer. Although the inactivated whole-cell vaccine elicited relatively low anti-PS-III antibody responses, it still conferred partial protection and detectable opsonophagocytic activity, likely attributed to immune recognition of additional nonpolysaccharide surface antigens.

## Conclusion

We have established a solid-phase synthesis platform for the concise and modular assembly of well-defined PS-III oligosaccharides. By integrating iterative glycosylation and phosphoramidite-based coupling on a photocleavable Merrifield resin, we successfully assembled the complete set of PS-III glycotopes bearing zero to five phospho-di-GlcNAc repeats in high yield and a time-efficient manner. Conjugation of these glycans to CRM197 enabled a systematic evaluation of structure–immunogenicity relationships in a murine model. Medium- and long-chain glycoconjugates elicited robust humoral immune responses, as demonstrated by glycan microarray analysis showing strong IgG and IgM binding affinity. Most synthetic conjugate vaccines (**1b/d/f–CRM197**) significantly reduced bacterial burdens in colon and fecal samples compared with whole-cell vaccine and PBS controls. Notably, the conjugate with five repeating units (**1f–CRM197**) mediated complete (100%) bacterial killing in opsonophagocytic assays. Our work not only establishes a new synthetic method for accessing PS-III glycans but also highlights the significant clinical potential of a semisynthetic PS-III conjugate vaccine for combating *C. difficile* infection.

## Supplementary Material



## Notes

All data supporting the findings of this study, including detailed experimental section, NMR characterization data, high-resolution mass spectrometric data, and additional figures (PDF)
