# Supplementary material for: Synthetic PS-III Glycoconjugate Vaccines Based on Solid-Phase Assembled Well-Defined Oligosaccharides Confers Protection against Clostridium difficile
Source: ACS Cent Sci. 2026 Aug 15;12(9):1429–38. doi: 10.1021/acscentsci.6c00855 (PMC13614052; doi:10.1021/acscentsci.6c00855)
Supplement: Supplementary file 1 [file oc6c00855_si_001.pdf]

Supplementary Information

Synthetic PS-III Glycoconjugate Vaccines Based on Solid-Phase Assembled Well-defined Oligosaccharides Confers Protection Against *Clostridium difficile*

Yiting Chen[1,2,‡], *Xiao Liu*[3,‡], *Taotao Zhang*[4,2,‡], *Zhen Wang*[3], *Yang Li*[6], *Jing Zeng*[2], *Hanrui Li*[5,2,7], *Chengli Zong*[3,*] *and Qiang Liu*[5,2,1,4,7,*]

Corresponding Authors: Chengli Zong, Email: chengli.zong@hainanu.edu.cn; Qiang Liu, Email: liuqiang@simm.ac.cn

1. Guangzhou University of Chinese Medicine, Guangdong 510006, China

2. Zhongshan Institute for Drug Discovery, Zhongshan 528400, China

3. School of Pharmaceutical Sciences, Key Laboratory of Tropical Biological Resources of Ministry of Education, Hainan University, Haikou 570228, China. Email: chengli.zong@hainanu.edu.cn

4. State Key Laboratory of Discovery and Utilization of Functional Components in Traditional Chinese Medicine, School of Pharmaceutical Sciences, Guizhou Medical University, Guiyang 561113, China

5. Carbohydrate-Based Drug Research Center, Shanghai Institute of Materia Medica, Chinese Academy of Sciences, Shanghai 201203, China. Email: liuqiang@simm.ac.cn

6. Department of Blood Transfusion, Zhongnan Hospital of Wuhan University, Wuhan 430071, China

7. University of Chinese Academy of Sciences, Beijing 100049, China

‡ These authors contributed equally to this work.

Table of Contents

1. General materials and methods....................3

2. Synthesis of building blocks and solid-phase synthesis of PS-III oligosaccharides....................5

3. Comparison of the yield of traditional method and our solid phase method....................35

4. CRM197 conjugation protocols....................35

5. Immunogenicity Evaluation of Fully Synthetic *C. difficile* PS-III Conjugate Vaccines....................39

6. Microarray printing and screening procedure....................41

7. References....................43

1. General materials and methods

Reactions were performed using anhydrous solvents under an atmosphere of Argon unless otherwise noted. Unless stated otherwise, all solvents were removed by rotary evaporation under reduced pressure at 40 °C. Molecular sieves were activated prior to use. Thin layer chromatography (TLC) was purchased from GoldSupplier. TLC plates were detected with UV absorption (254 nm), and sprayed with 10% sulfuric acid in methanol (1:9, v/v) and Ceric ammonium molybdate solution (contains H_2SO_4), followed by heating for visualization. Silica column chromatography was purchased from GoldSupplier (200-300 mesh) Shandong, China. Flash column chromatography was performed on SepaFlash® column. Reversed phase chromatography was performed on C18 columns (40-60 µm, 120Å). Size-exclusion chromatography was performed on a Toyopearl HW-40F, HW-50S column.

^{1}H, ^{13}C, ^{31}P and two-dimensional NMR spectra were recorded on a Bruker Avance 500, a Bruker Avance 600, and a Bruker Avance 800. Chemical shifts (δ) are reported in parts per million (ppm) relative to the respective residual solvent peaks ($CDCl_3$: δ 7.26 in ^{1}H and 77.20 in ^{13}C NMR; D_2O: δ 4.79 in ^{1}H NMR). The following abbreviations are used to indicate peak multiplicities: s singlet; d doublet; dd doublet of doublets; t triplet; dt doublet of triplets; q quartet; m multiplet. Coupling constants (*J*) are reported in Hertz (Hz). LC-MS were performed on Agilent 1260 Infinity II. ESI-HRMS were performed on Exactive Plus 102405P (Thermo Scientific). A sciFLEXARRAYER S3 (Scienion) was used for printing the glycan arrays, and a INNOSCAN 710 (INNPSYS) device was used for the glycan array read-out. CRM197 protein was obtained from Chengli Zong's group. Chemical reagents were purchased from Bide Pharmatech Ltd. and J&K Scientific Ltd. 1,3,4,6-Tetra-*O*-acetyl-2-azido-2-deoxy-β-ᴅ-glucopyranose (compound **9**) was purchased from Jinan Samuel Pharmaceutical Co., Ltd, China. All chemical reagent abbreviations and full name are listed in Table S1.

Table S1. Abbreviations of Chemical Reagents

**Abbreviation**	**Full name**
TolSH	*p*-toluenethiol
DCM	dichloromethane
$BF_3 \cdot Et_2O$	boron trifluoride diethyl etherate
MeOH	methanol
NaOMe	sodium methoxide
ACN	acetonitrile
$PhCH(OMe)_2$	benzaldehyde dimethyl acetal
Et_3N	triethylamine
EA	ethyl acetate
PE	petroleum ether
DMF	*N,N*-dimethylformamide
NaH	sodium hydride
NapBr	2-(bromomethyl)naphthalene
Et_3SiH	triethylsilane
$PhBCl_2$	dichloro(phenyl)borane
LevOH	levulinic acid
EDC	1-ethyl-3-(3-dimethylaminopropyl)carbodiimide
DIPEA	*N,N*-diisopropylethylamine
DMAP	4-dimethylaminopyridine
NBS	*N*-bromosuccinimide
TMSOTf	trimethylsilyl trifluoromethanesulfonate
PBS	phosphate buffer solution
DDQ	2,3-dichloro-5,6-dicyano-1,4-benzoquinone
BnBr	benzyl bromide
FmocCl	9-fluorenylmethyloxycarbonyl chloride
Et_2O	ether
NIS	*N*-iodosuccinimide

Abbreviation	Full name
AgOTf	silver trifluoromethanesulfonate
THF	tetrahydrofuran
AcOH	acetic acid
Ac_2O	acetic anhydride
DmtCl	4,4′-dimethoxytrityl chloride
N_2H_4·AcOH	hydrazine acetate
TBAI	tetrabutylammonium iodide
TfOH	trifluoromethanesulfonic acid
MsOH	methanesulfonic acid
DBU	1,8-diazabicyclo[5.4.0]undec-7-ene
ETT	5-(Ethylthio)-1H-tetrazole
CSO	(1*S*)-(+)-(10-camphorsulfonyl)oxaziridine
TCA	Trichloroacetonitrile
DMSO	dimethyl sulfoxide

2. Synthesis of building blocks and solid-phase synthesis of PS-III oligosaccharides

Synthesis of compound 17

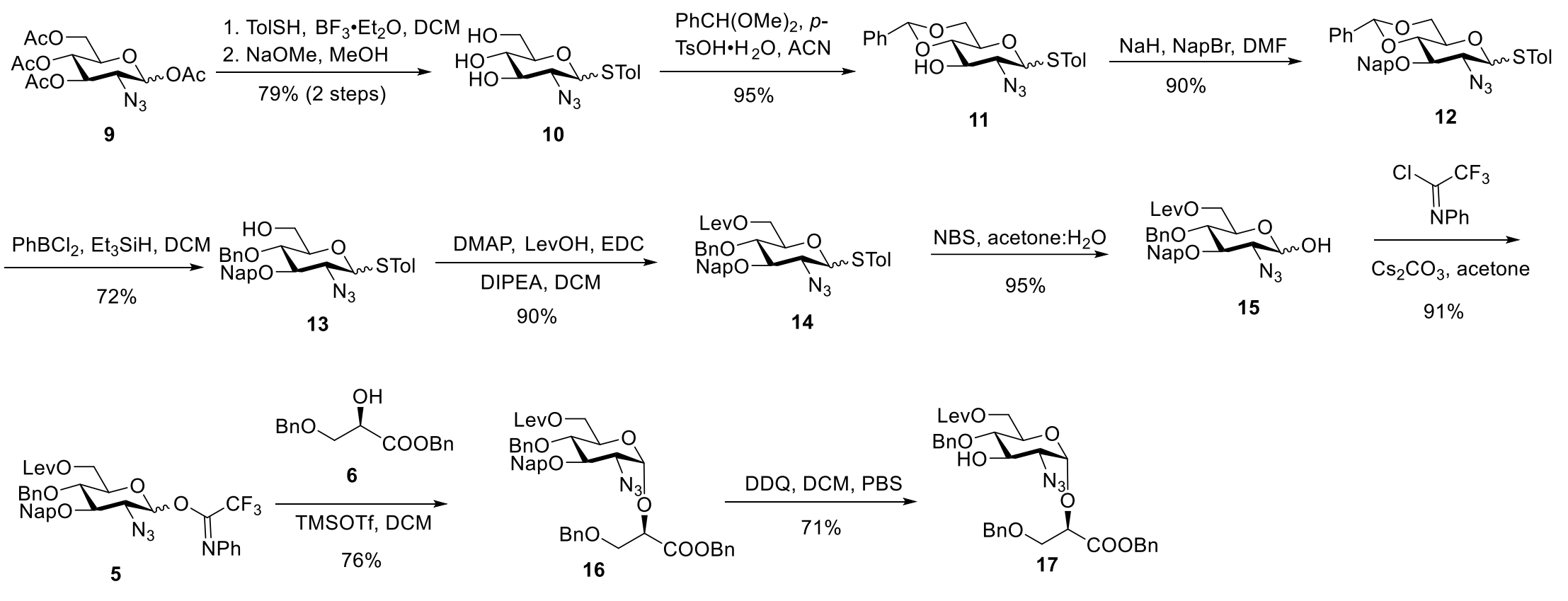

Scheme S1. Synthesis of compound **17**

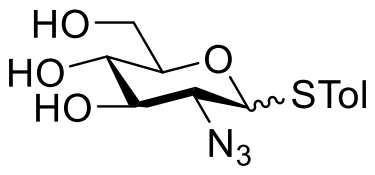

10

***p*-methylphenyl 2-azido-2-deoxy-1-thio-D-glucopyranoside**

The commercially available (purchased from Jinan Samuel Pharmaceutical Co., Ltd, China) compound **9** (30.00 g, 80.36 mmol) and TolSH (14.97 g, 120.54 mmol) were dissolved in 160 mL of anhydrous DCM, and boron trifluoride diethyl etherate ($BF_3 \cdot Et_2O$) (61.93 mL, 482.16 mmol) was slowly added at 0 °C. Stir the mixture overnight in a 40 °C oil bath. After the reaction is completed, the excess $BF_3 \cdot Et_2O$ was quenched with $NaHCO_3$ at 0 °C, diluted with DCM, washed with sat. aq. $NaHCO_3$ 3 times, and then washed with NaCl, and the organic phase was dried with Na_2SO_4, filtered and concentrated. The crude product was then dissolved in 268 mL of MeOH, and NaOMe was slowly added to adjust the pH of the reaction to pH > 10, stirred overnight at room temperature. After the reaction was completed, acid H^+ resin (Amberlite® IR120 hydrogen form, Sigma) was added to adjust the pH < 7 of the reaction system, filtered and concentrated. Column chromatography on silica gel (DCM/MeOH = 40/1→38/1→35/1→30/1) obtained compound **10** (19.77 g, 79% over 2 steps, α/β = 1/0.5) as light yellow oil. TLC: DCM/MeOH = 20/1 R_f = 0.25. ^{1}H NMR (500 MHz, Chloroform-*d*) δ 7.43 (d, 1H, Ar), 7.36 (d, 2H, Ar), 7.12 (dd, *J* = 7.9, 9.8 Hz, 3H, Ar), 5.47 (d, *J* = 4.6 Hz, 1H, H1-α), 4.46 (d, *J* = 10.1 Hz, 0.5H, H1-β), 4.20 (m, 1H, H6a-β, H6b-β), 4.05 (d, *J* = 22.4 Hz, 2H, H6a-α, H6b-α), 3.90 (m, 2H, 2xOH-α), 3.82 (m, 4H, H2-α, H3-α, H4-α, H5-α), 3.68 (t, *J* = 8.2 Hz, 1H, 2xOH-β), 3.58 (t, *J* = 9.4 Hz, 0.5H, H4-β), 3.48 (t, *J* = 8.9 Hz, 0.5H, H3-β), 3.32 (m, 0.5H, H5-β), 3.28 (m, 0.5H, H2-β), 2.80 (s, 0.5H, OH-β), 2.70 (s, 1H, OH-α), 2.34 (s, 1.5H, CH3-β), 2.32 (s, 3H, CH3-α). ^{13}C NMR (126 MHz, Chloroform-*d*) δ 138.8, 138.3, 133.2, 133.1, 130.1 (d, *J* = 4.4 Hz), 129.5, 128.1, 88.0 (C1-α), 87.2 (C1-β), 79.5 (C5-β), 77.00 (C3-β), 73.6 (C5-α), 72.4 (C3-α), 70.1 (C4-α), 69.5 (C4-β), 65.3 (C2-β), 63.6 (C2-α), 61.7 (C6-β), 61.2 (C6-α), 21.3 (CH3-β), 21.2 (CH3-α). HRMS (ESI) *m/z*: calculated for $C_{13}H_{18}O_4S$ $[M+H]^+$: 312.1013, found: 312.1056. The NMR data of **10** is in agreement with published data.[1]

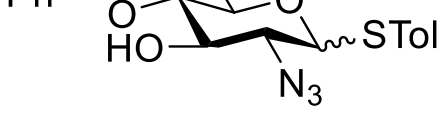

11

***p*-methylphenyl 2-azido-2-deoxy-4,6-*O*-benzylidene-1-thio-D-glucopyranoside**

Similar to a previous report,[2] compound **10** (19.77 g, 63.49 mmol) was dissolved in 352 mL of anhydrous ACN, benzaldehyde dimethyl acetal [$PhCH(OMe)_2$] (19.06 mL, 126.99 mmol) and *p*-toluenesulfonic acid monohydrate (*p*-$TsOH{\cdot}H_2O$) (1.21 g, 6.35 mmol) were added, the mixture was stirred for 3 hours at room temperature. After the reaction of most of the raw materials is completed, it is neutralized with Et_3N and concentrated in vacuum. The crude product was dissolved with EA, washed 3 times with sat. aq. NaCl, and the organic phase was dried with Na_2SO_4, filtered and concentrated. Column chromatography on silica gel (PE/EA = 10/1→8/1→4/1→2/1) obtained compound **11** (24.10 g, 95%. α/β = 1/0.5) as white solid. TLC: PE/EA = 6/1 R_f = 0.3. ^{1}H NMR (500 MHz, Chloroform-*d*) δ 7.51 (m, 2H, Ar), 7.46 (m, 2H, Ar), 7.39 (m, 6H, Ar), 7.16 (dd, *J* = 8.0, 12.0 Hz, 3H, Ar), 5.56 (s, 1H, PhCH-α), 5.52 (s, 0.5H, PhCH-β), 5.50 (d, *J* = 5.6 Hz, 1H, H1-α), 4.48 (d, *J* = 10.1 Hz, 0.5H, H1-β), 4.40 (m, 1.5H, H5-α, H6a-β), 4.24 (dd, *J* = 5.0, 10.3 Hz, 1H, H6a-α), 4.08 (dd, *J* = 2.5, 9.6 Hz, 1H, H2-α), 3.91 (dd, *J* = 5.6, 10.0 Hz, 1H, H6b-α), 3.76 (m, 2H, H3-α, H4-β, H6b-β), 3.58 (t, *J* = 9.4 Hz, 1H, H4-α), 3.44 (m, 1H, H5-β, H3-β), 3.32 (dd, *J* = 9.0, 10.2 Hz, 0.5H, H2-β), 2.75 (d, *J* = 2.7 Hz, 1H, OH-α), 2.72 (d, *J* = 2.8 Hz, 0.5H, OH-β), 2.37 (s, 1.5H, CH3-β), 2.34 (s, 3H, CH3-α). ^{13}C NMR (126 MHz, Chloroform-d) δ 192.6, 139.1, 138.4, 138.1, 136.9, 136.9, 134.5, 134.3, 133.2, 130.1, 130.0, 129.8, 129.5, 129.2, 129.1, 128.5, 128.5, 128.5, 128.3, 126.8, 126.4, 126.3, 103.2, 102.2, 102.0, 88.2, 86.8, 81.8, 74.1, 70.7, 70.3, 68.6, 68.5, 65.1, 63.9, 63.4, 52.8, 21.3, 21.2. HRMS (ESI) *m/z*: calculated for $C_{20}H_{22}N_3O_4S$ $[M+H]^+$: 400.1326, found: 400.1321. The NMR data of **11** is in agreement with published data.[2]

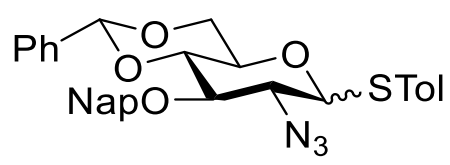

12

p-methylphenyl 2-azido-2-deoxy-4,6-*O*-benzylidene-3-*O*-(naphthylmethyl)-1-thio-ᴅ-glucopyranoside

Similar to a previous report,[3] compound **11** (25.36 g, 63.49 mmol) was dissolved in 141 mL of DMF, 60% NaH (4.06 g, 101.59 mmol) was added at 0 °C, stirred for 10 minutes, then NapBr (21.05 g, 95.24 mmol) was added and stirred for 2 hours at room temperature. After the reaction is completed, quenched with 15 mL of MeOH at 0 °C, the solvent was evaporated, and the crude product was dissolved with EA and washed with H_2O and sat. aq. NaCl. The organic phase was dried with Na_2SO_4, filtered and concentrated. The crude product was recrystallized with PE/DCM,

filtered and dried to obtain compound **12** (30.66 g, 90%, α/β = 1/0.1) as white solid. TLC: PE/EA = 6/1 R_f = 0.8. ^{1}H NMR (500 MHz, Chloroform-d) δ 7.86 – 7.73 (m, 4H, Ar), 7.56 – 7.43 (m, 6H, Ar), 7.40 (m, 5H, Ar), 7.14 (d, *J* = 7.9 Hz, 2H, Ar), 5.61 (s, 1H, PhCH-α), 5.51 (d, *J* = 4.9 Hz, 1H, H1-α), 5.12 (d, *J* = 11.1 Hz, 1H, C*H*H-Nap), 5.00 (d, *J* = 11.2 Hz, 1H, C*H*H-Nap), 4.45 (m, 1H, H5-α), 4.23 (dd, *J* = 10.4, 4.9 Hz, 1H, H6a-α), 4.06 – 3.96 (m, 2H, H2-α, H3-α), 3.80-7.76 (m, 2H, H4-α, H6b-α), 2.34 (s, 3H, CH3-STol). ^{13}C NMR (126 MHz, Chloroform-*d*) δ 138.5, 137.3, 135.3, 133.4, 133.3 (d, *J* = 3.7 Hz), 130.1, 129.3, 129.2, 128.5, 128.3, 128.1, 127.8, 127.2, 126.2 (t, *J* = 4.7 Hz), 126.1, 101.7, 88.3 (C1-α), 82.9 (C4-α), 78.1 (C3-α), 75.3, 68.8 (C6-α), 63.8 (d, *J* = 2.1 Hz) (C2-α, C5-α), 21.3. HRMS (ESI) *m/z*: calculated for $C_{31}H_{30}N_3O_4S$ [M+H]$^+$: 540.1957, found: 540.1945. The NMR data of **12** is in agreement with published data.[3]

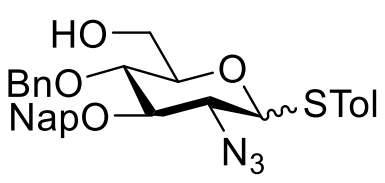

13

***p*-methylphenyl 2-azido-2-deoxy-4-*O*-benzyl-3-*O*-(2-naphthylmethyl)-1-thio-ᴅ-glucopyranoside**

Compound **12** (28.96 g, 53.66 mmol) was dissolved in 273 mL of DCM, and the freshly activated 4 Å molecular sieve was added, stir at room temperature and argon for 30 minutes. The mixture was cooled to -78 °C and Et_3SiH (21.43 mL, 134.15 mmol) and $PhBCl_2$ (13.92 mL, 107.32 mmol) were added. The reaction was then stirred at 0 °C for 30 minutes. Et_3N/MeOH (1/1) was added to quench, diluted with DCM, washed with sat. aq. Sodium bicarbonate$NaHCO_3$ (x2) and sat. aq. NaCl, the organic phase was dried with Na_2SO_4, filtered and concentrated. Column chromatography on silica gel (PE/EA = 12/1→11/1→10/1) to obtained compound **13** (20.74 g, 72%, α/β = 1/0.2) as light yellow oil. TLC: PE/EA = 4/1 R_f = 0.4. ^{1}H NMR (500 MHz, Chloroform-*d*) δ 7.81 (m, 5H, Ar), 7.48 (m, 4H, Ar), 7.33 (m, 9H, Ar), 7.12 (d, *J* = 7.8 Hz, 2H, Ar), 5.48 (d, *J* = 4.6 Hz, 1H, H1-α), 5.05 (q, *J* = 10.8 Hz, 2H, CH2-Nap), 4.89 (dd, *J* = 10.8, 14.3 Hz, 1H, C*H*H-Bn), 4.69 (d, *J* = 11.3 Hz, 1H, C*H*H-Bn), 4.28 (m, 1H, H5-α), 3.90 (m, 2H, H2-α, H3-α), 3.75 (m, 2H, 2xH6-α), 3.66 (t, *J* = 8.6 Hz, 1H, H4-α), 2.32 (s, 3H, CH3-STol). ^{13}C NMR (126 MHz, Chloroform-*d*) δ 138.4, 137.9, 135.3, 133.9, 133.4, 133.3 – 133.1 (m), 130.1, 129.3, 128.7, 128.4, 128.1, 127.9 (d, *J* = 8.6 Hz), 127.8, 127.0, 126.2, 126.1, 87.6 (C1-α), 81.8 (C3-α), 78.1 (C4-α), 75.9, 75.3, 72.5 (C5-α), 64.3 (C2-α), 61.6 (C6-α), 21.3. HRMS (ESI) *m/z*: calculated for $C_{31}H_{31}N_3O_4SNa$ [M+Na]$^+$:

564.1933, found: 564.1923. The NMR data of **13** is in agreement with published data.[3]

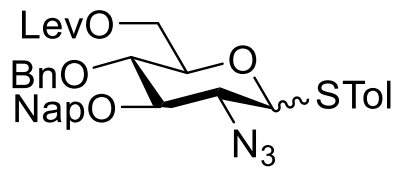

14

_p_-methylphenyl 2-azido-2-deoxy-4-_O_-benzyl-3-_O_-(2-naphthylmethyl)-6-_O_-levulinyl-1-thio-D-glucopyranoside

Compound **13** (25.90 g, 47.87 mmol) was dissolved in 218 mL of DCM, and LevOH (8.37 mL, 96.18 mmol), EDC (11.86 mL, 67.02 mmol), DIPEA (4.17mL, 23.94 mmol) and DMAP (585 mg, 4.79 mmol) were added sequentially. Stir the mixture overnight at room temperature under argon. After the reaction is completed, diluted with EA, washed once each with sat. aq. NH_4Cl, $NaHCO_3$ and NaCl, and the organic phase is dried with anhydrous Na_2SO_4, filtered and concentrated. Column chromatography on silica gel (PE / EA = 12/1→10/1→8/1→6/1) obtained compound **14** (27.60, 90%) as white solid. TLC: PE/EA = 4/1 R_f = 0.35. ^{1}H NMR (500 MHz, Chloroform-*d*) δ 7.84 – 7.73 (m, *J* = 7.5, 9.1 Hz, 4H, Ar), 7.52 – 7.42 (m, 4H, Ar), 7.40 – 7.36 (m, 2H, Ar), 7.35 – 7.25 (m, 5H, Ar), 7.10 (d, *J* = 8.1 Hz, 2H, Ar), 5.51 (d, *J* = 4.9 Hz, 1H, H1-α), 5.08 (d, *J* = 10.8 Hz, 1H, C*H*H-Nap), 5.01 (d, *J* = 10.9 Hz, 1H, C*H*H-Nap), 4.87 (d, *J* = 10.9 Hz, 1H, C*H*H-Bn), 4.61 (d, *J* = 10.9 Hz, 1H, C*H*H-Bn), 4.49-4.45 (m, 1H, H5-α), 4.33 (dd, *J* = 4.9, 12.0 Hz, 1H, H6a-α), 4.24 (dd, *J* = 2.2, 12.0 Hz, 1H, H6b-α), 3.94 – 3.89 (m, 2H, H2-α, H3-α), 3.58 (dd, *J* = 8.1, 10.1 Hz, 1H, H4-α), 2.66 (q, *J* = 6.6 Hz, 2H, CH2-Lev), 2.51 (t, *J* = 6.6 Hz, 2H, CH2-Lev), 2.30 (s, 3H, CH3-Lev), 2.11 (s, 3H, CH3-STol). ^{13}C NMR (126 MHz, Chloroform-*d*) δ 206.4, 172.5, 138.2, 137.5, 135.6, 135.1, 133.3, 133.1, 132.8, 130.0, 129.2, 128.6, 128.3, 128.0 (d, *J* = 10.2 Hz), 127.7, 127.0, 126.1 (m), 87.3 (C1-α), 81.8 (C3-α), 75.8 (C4-α), 75.2, 70.1 (C5-α), 64.1 (C2-α), 63.0 (C6-α), 37.8, 29.8, 27.7, 21.1. HRMS (ESI) *m/z*: calculated for $C_{36}H_{38}N_3O_6S$ $[M+H]^+$: 640.2481, found: 640.2470.

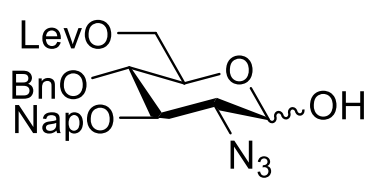

15

2-azido-2-deoxy-4-_O_-benzyl-3-_O_-(2-naphthylmethyl)-6-_O_-levulinyl-D-glucopyranose

Compound **14** (19.31 g, 30.21 mmol) was dissolved in 302 mL of acetone:water (10:1) mixture,

and then NBS (21.51 g, 120.83 mmol) was added at 0 °C. The mixture was stirred at room temperature. After the reaction was completed, it was quenched with saturated $Na_2S_2O_3$, acetone was evaporated, extracted with EA, and the organic phase was washed twice with sat. aq. NaCl, then dried with anhydrous Na_2SO_4, filtered and concentrated. Column chromatography on silica gel (PE/EA = 4/1→3/1→21→1/1) obtained compound **15** (15.15 g, 95%, α/β = 1/0.5) as light yellow oil. TLC: PE/EA = 3/1 R_f = 0.1. ^{1}H NMR (500 MHz, Chloroform-*d*) δ 7.90 – 7.76 (m, 7H, Ar), 7.56 – 7.46 (m, 6H, Ar), 7.40 – 7.24 (m, 10H, Ar), 5.35 (t, *J* = 3.1 Hz, 1H, Ar), 5.32 (d, *J* = 1.6 Hz, 1H, H1-α), 5.13 – 5.05 (m, 2.5H, CH2 Nap-α, C*H*H Nap-β), 5.06 – 4.97 (m, 0.5H, C*H*H Nap-β), 4.91 (dd, *J* = 7.5, 10.9 Hz, 1.5H, C*H*H Bn-α, C*H*H Bn-β), 4.65 (dd, *J* = 7.0, 11.1 Hz, 2H, C*H*H Bn-α, C*H*H Bn-β, H1-β), 4.45 – 4.37 (m, 1.5H, H6a-α, H6a-β), 4.28 – 4.08 (m, 3.5H, H6b-α, H6b-β, H5-α, H3-α), 3.71 – 3.66 (m, 1H, OH-α), 3.61 – 3.52 (m, 3H, H4-α, H3-β, H4-β, H5-β, OH-β), 3.53 – 3.42 (m, 1.5H, H2-α, H2-β), 2.78 (q, *J* = 6.5 Hz, 3H, CH2 Lev-α, CH2 Lev-β), 2.60 (t, *J* = 6.3 Hz, 3H, CH2 Lev-α, CH2 Lev-β), 2.20 (m, 4.5H, CH3 Lev-α, CH3 Lev-β). ^{13}C NMR (126 MHz, Chloroform-*d*) δ 207.4, 207.3, 172.6, 137.7, 137.6, 135.3, 135.3, 133.4, 133.2, 128.7, 128.4, 128.2, 128.1, 128.1, 128.0, 127.8, 127.8, 127.1, 126.3, 126.2, 126.2, 96.3 (C1-β), 92.1 (C1-α), 83.2 (C3-β), 80.4 (C3-α), 78.5 (C4-α), 77.6 (C4-β), 75.8, 75.2, 75.2, 73.4 (C5-β), 69.3 (C5-α), 67.7 (C2-β), 64.2 (C2-α), 63.2 (C6-β), 63.0 (C6-α), 38.2, 38.0, 30.0, 30.0, 28.1, 28.0. HRMS (ESI) *m/z*: calculated for $C_{29}H_{31}N_3O_7Na$ $[M+Na]^+$: 556.2060, found: 556.2045. The NMR data of **14** is in agreement with published data.[4]

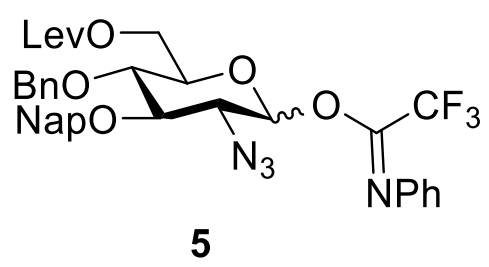

5

2-azido-2-deoxy-4-*O*-benzyl-3-*O*-(2-naphthylmethyl)-6-*O*-levulinyl-D-glucopyranosyl *N*-phenyl-trifluoroacetimidate

Compound **15** (15.15 g, 28.42 mmol) was dissolved in 71 mL of anhydrous acetone, followed by the addition of Cs_2CO_3 (13.89 g, 42.63 mmol) and 2,2,2-trifluoro-*N*-phenethyliminoyl chloride (5.99 mL, 36.95 mmol) at 0 °C. Stir the mixture for 2 hours at room temperature. After the reaction was complete, three drops of Et_3N were added, filtered and concentrated. Column chromatography on silica gel (PE/EA = 5/1→4/1→3/1, moisten the column with 2% Et_3N) obtained compound **5**

(18.02 g, 91%, α/β = 1/0.3) as colorless oil. TLC: PE/EA = 4/1 R_f = 0.5. ^{1}H NMR (500 MHz, Chloroform-*d*) δ 7.9 – 7.7 (m, 4H, Ar), 7.6 – 7.4 (m, 3H, Ar), 7.4 – 7.2 (m, 7H, Ar), 7.1 (t, *J* = 7.5 Hz, 1H, Ar), 6.9 (d, *J* = 7.8 Hz, 2H, Ar), 5.5 (s, 1H, H1-α), 5.1 (d, *J* = 11.1 Hz, 1H, C*H*H Nap-α), 5.0 (d, *J* = 11.0 Hz, 1H, C*H*H Nap-α), 4.9 (d, *J* = 10.8 Hz, 1H, C*H*H Bn-α), 4.6 (d, *J* = 10.8 Hz, 1H, C*H*H Bn-α), 4.4 – 4.2 (m, 2H, 2xH6-α), 3.8 – 3.3 (m, 4H, H2-α, H3-α, H4-α, H5-α), 2.8 – 2.6 (m, 2H, CH2 Lev-α), 2.6 (t, *J* = 6.9 Hz, 2H, CH2 Lev-α), 2.1 (s, 3H, CH3 Lev-α). ^{13}C NMR (126 MHz, Chloroform-*d*) δ 206.4, 172.4, 143.2, 137.4, 135.0, 133.4, 133.2, 128.9, 128.7, 128.4, 128.1 (t, *J* = 6.9 Hz), 127.8, 127.0, 126.3, 126.2, 126.0, 124.7, 119.3, 82.9, 75.8, 75.3, 74.0, 65.4, 62.5, 37.9, 29.8, 27.9.

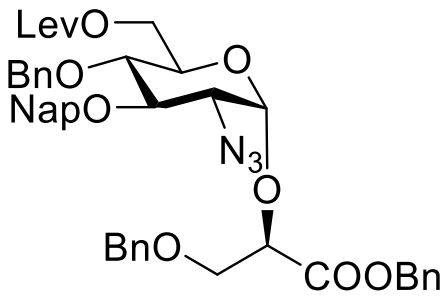

16

Benzyl (2*R*)-3-(benzyloxy)-2-*O*-[2-azido-4-*O*-benzyl-3-*O*-(2-naphthylmethyl)-6-*O*-levulinyl-2-deoxy-α-D-glucopyranosyl]propanoate

A mixture of compounds **5** (8.00 g, 11.36 mmol) and **6**[5] (2.96 g, 10.33 mmol) were evaporated three times with anhydrous toluene and dried in vacuum. Dissolve the mixture in 103 mL of anhydrous DCM, add the freshly activated 4 Å molecular sieve, and stir at room temperature under argon for 30 minutes. TMSOTf (187 µL, 1.03 mmol) was then added at -78 °C and then raised to 0 °C with stirring for 30 minutes. After the reaction was completed, it was quenched with 2 mL of pyridine, diluted with DCM, washed twice with sat. aq. $NaHCO_3$, and the organic layer was dried with Na_2SO_4, filtered, and concentrated. Column chromatography on silica gel (PE/EA = 8/1→6/1 → 4/1 → 2/1) obtained compound **16** (6.27 g, 76%) was purified by silica gel column chromatography with light yellow oil. TLC:PE/EA = 3/1 R_f = 0.4. ^{1}H NMR (500 MHz, Chloroform-d) δ 7.85 – 7.74 (m, 4H, Ar), 7.49 (m, 3H, Ar), 7.37 – 7.22 (m, 15H, Ar), 5.27 – 5.16 (dd, 2H, CH2-Bn), 5.11 (d, *J* = 3.6 Hz, 1H, H1), 5.09 (d, *J* = 10.8 Hz, 1H, C*H*H-Nap), 5.02 (d, *J* = 10.8 Hz, 1H, C*H*H-Nap), 4.88 (d, *J* = 10.9 Hz, 1H, C*H*H-Bn), 4.61 – 4.48 (m, 4H, C*H*H-Bn, CH2-Bn, CH-Gro), 4.21 – 4.08 (m, 4H, 2xH6, H5, H3), 3.88 – 3.77 (m, 2H, CH2-Gro), 3.58 (t, *J* = 9.1 Hz, 1H, H4), 3.44 (dd, *J* = 10.3, 3.6 Hz, 1H, H2), 2.69 (m, 2H, CH2-Lev), 2.59 – 2.47 (m, 2H, CH2-Lev), 2.14

(s, 3H, CH3-Lev)。 ^{13}C NMR (126 MHz, Chloroform-d) δ 206.4, 172.5, 169.2, 137.9, 137.6, 135.4, 135.2, 133.4, 133.2, 128.1, 128.0, 128.0, 127.9, 127.9, 127.8, 127.0, 126.2, 126.1, 126.1, 96.7 (C1), 80.3 (C3), 78.0 (C4), 75.7, 75.2, 74.4, 73.5, 70.2, 69.7 (C5), 67.4, 63.4 (C2), 62.7 (C6), 37.9, 29.9, 27.9. HRMS (ESI) *m/z*: calculated for $C_{46}H_{48}N_3O_{10}$ [M+H]$^+$: 802.3334, found: 802.3394. The NMR data of **16** is in agreement with published data.[4]

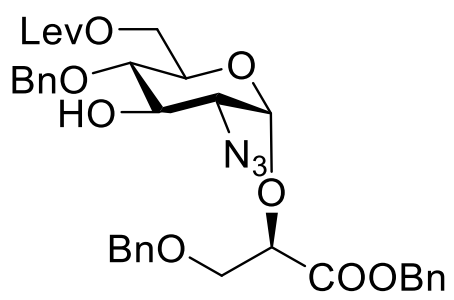

17

Benzyl (2*R*)-3-(benzyloxy)-2-*O*-[2-azido-4-*O*-benzyl-6-*O*-levulinyl-2-deoxy-α-ᴅ-glucopyranosyl]propanoate

Compound **16** (6.13 g, 7.65 mmol) was dissolved in 77 mL of DCM and 7.70 mL of PBS, DDQ (2.60 g, 11.47 mmol) was added at 0 °C and stirred overnight at room temperature. After the reaction was completed, it was quenched with sat. aq. $NaHCO_3$ and $Na_2S_2O_3$ solutions, extracted with DCM, and the organic layer was dried with Na_2SO_4, filtered and concentrated. Column chromatography on silica gel (PE/EA = 7/1→5/1→4/1→3/1→2.5/1→2/1) obtained compound **17** (3.58 g, 71%) as light yellow oil. TLC:PE/EA = 3/1 R_f = 0.25. ^{1}H NMR (500 MHz, Chloroform-*d*) δ 7.38 – 7.28 (m, 10H, Ar), 7.25 (dd, *J* = 3.8, 2.6 Hz, 5H, Ar), 5.26 – 5.14 (m, 2H, CH2-Bn), 5.10 (d, *J* = 3.6 Hz, 1H, H1), 4.78 (d, *J* = 11.3 Hz, 1H, C*H*H-Bn), 4.67 (d, *J* = 11.3 Hz, 1H, C*H*H-Bn), 4.58 – 4.44 (m, 3H, CH2-Bn, CH-Gro), 4.21 – 4.05 (m, 4H, 2xH6, H3, H5), 3.87 – 3.74 (m, 2H, CH2-Gro), 3.44 (dd, *J* = 10.1, 8.6 Hz, 1H, H4), 3.24 (dd, *J* = 10.3, 3.6 Hz, 1H, H2), 2.79 – 2.62 (m, 2H, CH2-Lev), 2.61 – 2.47 (m, 2H, CH2-Lev), 2.15 (s, 3H, CH3-Lev). ^{13}C NMR (126 MHz, Chloroform-d) δ 206.5, 172.4, 169.1, 137.9, 137.5, 135.2, 128.6, 128.4, 128.1, 128.1, 127.8, 127.8, 96.6 (C1), 77.9 (C4), 74.9, 74.4, 73.5, 71.9 (C3), 70.1, 69.2 (C5), 67.2, 62.9 (C2), 62.7 (C6), 37.9, 29.8, 27.8. HRMS (ESI) *m/z*: calculated for $C_{35}H_{40}N_3O_{10}$ [M+H]$^+$: 662.2708, found: 662.2706. The NMR data of **17** is in agreement with published data.[4]

Synthesis of compound 4

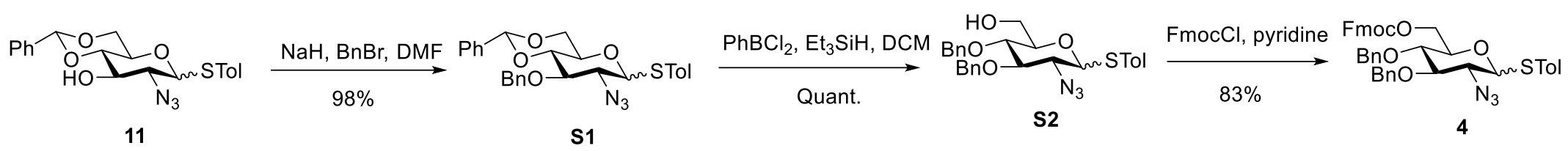

Scheme S2. Synthesis of compound **4**

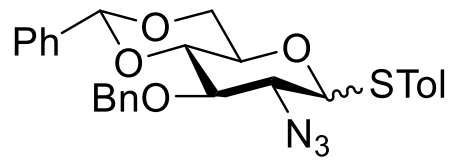

S1

***p*-methylphenyl 2-azido-2-deoxy-4,6-*O*-benzylidene-3-*O*-benzyl-1-thio-D-glucopyranoside**

Similar to a previous report,[6] compound **11** (23.22 g, 58.12 mmol) was dissolved in 129 mL of DMF, 60% NaH (3.72 g, 93.00 mmol) was added at 0 °C, stirred for 10 minutes, then BnBr (10.31 mL, 87.19 mmol) was added and stirred at room temperature for 2 hours. After the reaction is completed, quenched with MeOH, the solvent was evaporated, the crude product was dissolved with EA, washed with H_2O and sat. aq. NaCl, and the organic layer was dried with Na_2SO_4, filtered and concentrated. Column chromatography on silica gel (PE/EA=40/1→35/1→30/1→25/1) obtained compound **S1** (27.89 g, 98%, α/β = 1/0.3) as white solid. TLC:PE/EA = 25/1 R_f = 0.45. ^{1}H NMR (500 MHz, Chloroform-*d*) δ 7.57 – 7.50 (m, 2H, Ar), 7.50 – 7.47 (m, 1H, Ar), 7.46-7.40 (m, 8H, Ar), 7.40 – 7.31 (m, 5H, Ar), 7.14 (dd, *J* = 7.9, 5.7 Hz, 2H, Ar), 5.59 (s, 1H, CHPh-α), 5.56 (s, 0.3H, CHPh-β), 5.52 (d, *J* = 4.9 Hz, 1H, H1-α), 4.97 (d, *J* = 10.8 Hz, 1H, C*H*H Bn-α), 4.90 (d, *J* = 10.9 Hz, 0.3H, C*H*H Bn-β), 4.83 (d, *J* = 10.9 Hz, 1H, C*H*H Bn-α), 4.77 (d, *J* = 10.9 Hz, 0.3H, C*H*H Bn-β), 4.48 – 4.40 (m, 1.3H, H5-α, H5-β), 4.38 (dd, *J* = 10.5, 4.9 Hz, 0.3H, H1-β), 4.23 (dd, *J* = 10.4, 4.9 Hz, 1H, H6a-α), 3.99 – 3.92 (m, 2H, H2-α, H3-α), 3.76 (m, 2.3H, H4-α, H6b-α, H6a-β), 3.62 (m, 0.6H, H3-β, H6b-β), 3.44 (m, 0.3H, H4-β), 3.33 (dd, *J* = 7.5, 2.6 Hz, 0.3H, H2-β), 3.31 – 3.24 (m, 0.3H, H2-β), 2.35 (s, 0.9H, SPhCH3-β), 2.34 (s, 3H, SPhCH3-α). ^{13}C NMR (126 MHz, Chloroform-d) δ 138.5, 137.8, 137.3, 134.7, 133.3, 130.1, 130.1, 129.2, 129.2, 128.6, 128.5, 128.5, 128.5, 128.4, 128.2, 128.1, 126.2, 126.1, 101.6, 101.4, 88.3 (C1-α), 86.7 (C1-β), 82.9 (C4-α), 81.4 (C3-β), 78.0 (C3-α), 75.3, 70.6 (C4-β), 68.8 (C6-α,C6-β), 64.6 (C2-β), 63.8 (C5-α), 63.7 (C2-α), 21.3. HRMS (ESI) *m/z*: calculated for $C_{27}H_{28}N_3O_4S$ $[M+H]^+$: 490.1795, found: 490.1784. The NMR data of **S1** is in agreement with published data.[6]

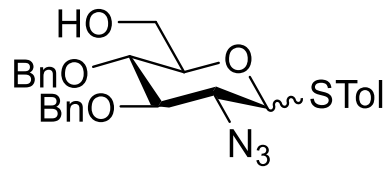

S2

***p*-methylphenyl 2-azido-2-deoxy-3, 4-di-*O*-benzyl--1-thio-D-glucopyranoside**

Similar to a previous report,[6] compound **S1** (12.45 g, 25.42 mmol) was dissolved in 135 mL of anhydrous DCM, then cooled to -78 °C, Et_3SiH (6.09 mL, 38.14 mmol) and $PhBCl_2$ (5.61 mL, 43.22 mmol) were added, and the reaction was stirred at -78 °C for 35 minutes. Quenched with Et_3N and MeOH at 0 °C, the reaction was diluted with DCM, washed twice with sat. aq. $NaHCO_3$, and the organic layer was dried with Na_2SO_4, filtered and concentrated. Column chromatography on silica gel (PE/EA = 12/1→10/1→8/1→6/1→4/1) obtained compound **S2** (12.49 g, Quant.) as light yellow oil. TLC:PE/EA = 10/1 R_f = 0.25. ^{1}H NMR (500 MHz, Chloroform-*d*) δ 7.42 (d, *J* = 8.2 Hz, 1H), 7.39 – 7.23 (m, 18H), 7.09 (t, *J* = 8.4 Hz, 3H), 5.41 (d, *J* = 5.0 Hz, 1H), 4.90 – 4.80 (m, 4H), 4.78 (d, *J* = 11.0 Hz, 1H), 4.66 (d, *J* = 11.1 Hz, 1H), 4.61 (d, *J* = 11.0 Hz, 1H), 4.35 (d, *J* = 10.1 Hz, 1H), 4.24 (dt, *J* = 3.2, 10.0 Hz, 1H), 3.85 – 3.76 (m, 3H), 3.72 (d, *J* = 3.3 Hz, 2H), 3.67 – 3.58 (m, 2H), 3.50 – 3.43 (m, 1H), 3.32 – 3.22 (m, 1H), 2.29 (s, 2H), 2.27 (s, 3H). ^{13}C NMR (126 MHz, Chloroform-*d*) δ 138.8, 138.2, 137.8, 137.7 (d, *J* = 3.3 Hz), 137.5, 133.8, 133.1, 129.9 (d, *J* = 3.9 Hz), 129.2, 128.6 – 128.4 (m), 128.1 (d, *J* = 7.8 Hz), 128.1 – 127.8 (m), 127.2, 79.6, 77.9, 77.4, 75.8, 75.6, 75.0 (d, *J* = 4.7 Hz), 65.1, 64.0, 61.7, 61.3, 21.1 (d, *J* = 3.8 Hz). HRMS (ESI) *m/z*: calculated for $C_{27}H_{29}N_3O_4SNa$ $[M+Na]^+$: 514.1776, found: 514.1768. The NMR data of **S2** is in agreement with published data.[6]

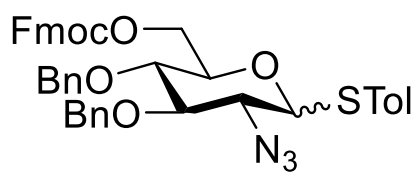

4

***p*-methylphenyl 2-azido-2-*O*-deoxy-3, 4-di-*O*-benzyl-6-*O*-(9-fluorenylmethyloxycarbonyl)- 1-thio-D-glucopyranoside**

Compound **S2** (12.49 g, 25.42 mmol) was dissolved in 254 mL of DCM, followed by the addition of pyridine (20.57 mL, 254.29 mmol) and FmocCl (16.45 g, 63.57 mmol) at 0 °C. Stir the mixture overnight at room temperature. After the reaction was completed, half of the DCM was evaporated, diluted with 300 mL of Et_2O, washed once each with 200 mL of H_2O, 200 mL of hydrogen chloride (HCl) (1 M), 200 mL of sat. aq. $NaHCO_3$ and 200 mL of sat. aq. NaCl, and the organic layer was dried with anhydrous $MgSO_4$, filtered and concentrated. Column chromatography on silica gel (PE/EA = 20/1→19/1→18/1) obtained compound **4** (15.11 g, 83%, α/β = 1/1) as white foamy solid. TLC:PE/EA = 10/1 R_f = 0.40. ^{1}H NMR (500 MHz, Chloroform-*d*) δ 7.76 (t, *J* = 7.5 Hz, 4H, Ar), 7.65 – 7.57 (m, 4H, Ar), 7.49 (d, *J* = 8.0 Hz, 2H, Ar), 7.45 – 7.24 (m, 30H, Ar), 7.09

(d, *J* = 7.9 Hz, 4H, Ar), 5.49 (d, *J* = 5.3 Hz, 1H, H1-α), 4.95 (d, *J* = 10.6 Hz, 1H, C*H*H Bn-α), 4.88 (d, *J* = 10.6 Hz, 3H, CH2 Bn-α, C*H*H Bn-β), 4.83 (dd, 2H, CH2 Bn-β), 4.60 (dd, *J* = 11.0, 15.3 Hz, 2H, C*H*H Bn-α, C*H*H Bn-β), 4.53 – 4.44 (m, 2H, H5-α, H6a-β), 4.43 – 4.33 (m, 7H, CH2 Fmoc-α, H1-β, 2xH6-α, CH2 Fmoc-β), 4.28 (m, 2H, H6b-β, CH Fmoc-α), 4.22 (t, *J* = 7.4 Hz, 1H, CH Fmoc-β), 3.93 (dd, *J* = 5.4, 10.3 Hz, 1H, H2-α), 3.85 (dd, *J* = 8.6, 10.4 Hz, 1H, H3-α), 3.63 (dd, *J* = 8.7, 10.1 Hz, 1H, H4-α), 3.56 – 3.46 (m, 3H, H5-β, H3-β, H4-β), 3.30 (t, *J* = 9.4 Hz, 1H, H2-β), 2.29 (s, 3H, SPhCH3-α), 2.28 (s, 3H, SPhCH3-β). ^{13}C NMR (126 MHz, Chloroform-*d*) δ 155.1, 143.5 (m), 143.3, 141.4 (d, *J* = 2.6 Hz), 139.0, 138.4, 137.6, 137.5 (d, *J* = 4.2 Hz), 134.4, 133.0, 130.1, 129.9, 129.3, 128.7 (d, *J* = 3.6 Hz), 128.4, 128.1 (m), 127.3 (d, *J* = 5.8 Hz), 127.0, 125.3 (m), 120.2 (d, *J* = 1.9 Hz), 87.7 (C1-α), 86.1 (C1-β), 85.2 (C3-β), 81.9 (C3-α), 78.1 (C4-α), 77.3 (C5-β), 77.1, 76.1, 75.9, 75.3 (d, *J* = 9.7 Hz), 70.1 (d, *J* = 7.5 Hz) (C5-α), 66.4 (d, *J* = 13.3 Hz) (C6-α, C6-β), 65.0 (C2-β), 64.2 (C2-α), 46.9 (d, *J* = 3.7 Hz), 21.3 (d, *J* = 6.1 Hz). HRMS (ESI) *m/z*: calculated for $C_{42}H_{40}N_3O_6S$ $[M+H]^+$: 714.2632, found: 714.2608.

Synthesis of phosphoramidite donor 2f

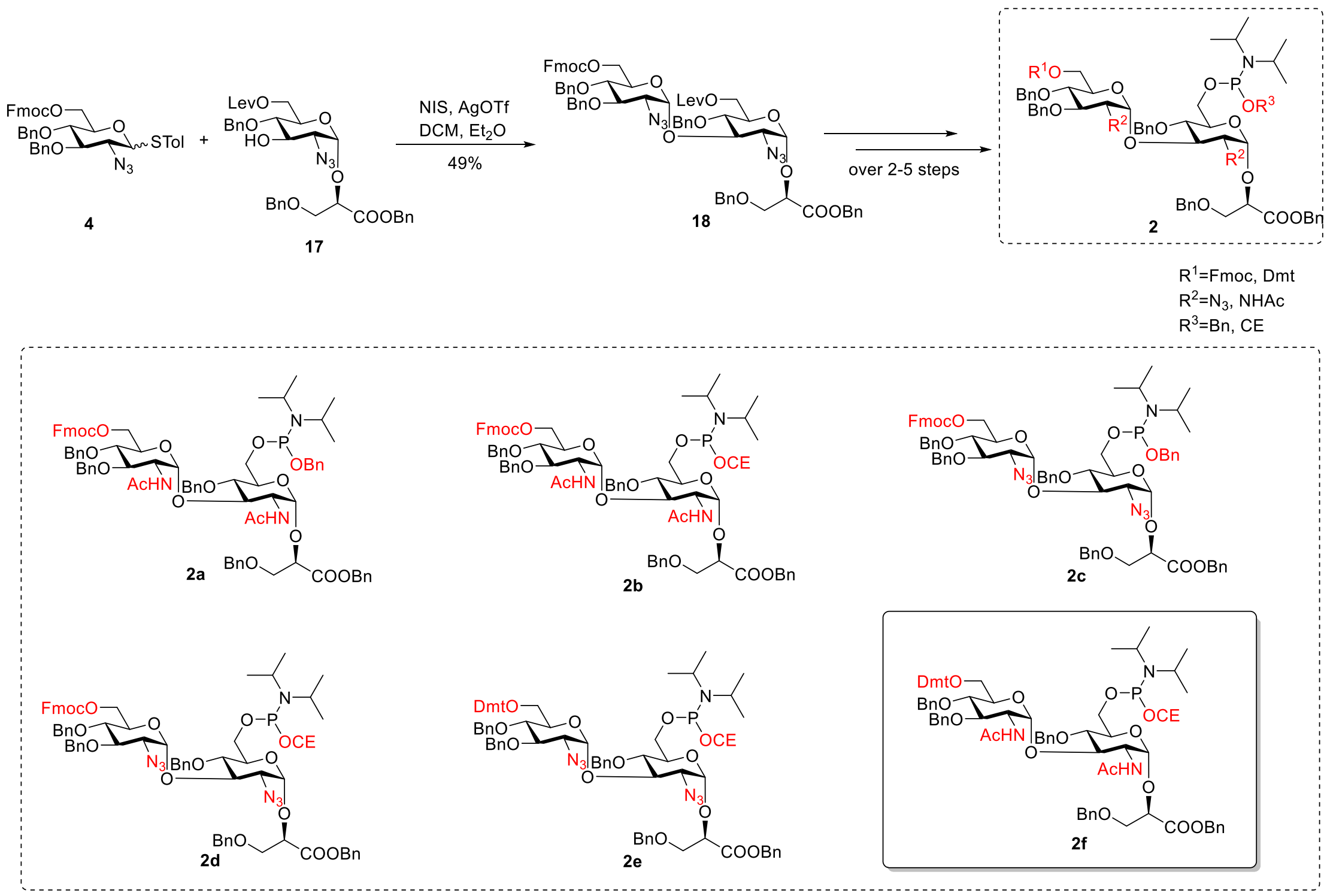

Figure S1. Synthesis of different disaccharide phosphoramidine donors

A series of six disaccharide phosphoramidite donors were synthesized through systematic

variation of the protecting groups at the 6-OH of glucosamine, the substituents at the C2-position of both glycan rings, and the phosphoramidite protecting groups. Initially, donors **2a** and **2b**, featuring a 6-Fmoc (9-fluorenylmethyloxycarbonyl) group, a 2-acetamido group, and Bn (benzyl) or CE (cyanoethyl) protection on the phosphoramidite, were prepared. Starting from compound **18**, **2a** was obtained via a three-step sequence including azide reduction, Lev (levulinyl) removal, and phosphitylation. However, **2a** proved difficult to purify, exhibited poor stability, and showed low coupling efficiency with a solid-phase trisaccharide acceptor. Under similar conditions, **2b** (with CE protection) was synthesized and demonstrated improved stability, yet it failed to couple effectively with the trisaccharide acceptor. Donors **2c** and **2d**, bearing a 6-Fmoc group, two 2-azido groups, and Bn or CE phosphoramidite protection, were subsequently prepared from **18** in two steps (Lev removal and phosphitylation). Both donors were highly unstable and underwent rapid hydrolysis upon concentration, likely due to the strong electron-donating effect of the azido group enhancing their reactivity. The initial use of the base-labile Fmoc group at the 6-position—intended to facilitate coupling efficiency monitoring during solid-phase synthesis—proved incompatible with the labile phosphoramidite group. Standard silica gel purification using triethylamine-containing eluents led to partial Fmoc deprotection during concentration, indicating a lack of orthogonality. Drawing from solid-phase oligonucleotide synthesis strategies,[7] an acid-labile Dmt (4,4′-dimethoxytrityl) group was adopted as the 6-OH protector, paired with the orthogonal CE-protected phosphoramidite. This approach afforded **2e** (2-azido) and **2f** (2-acetamido). Synthesis of **2e** from **18** involved four steps: Fmoc removal, Dmt protection, Lev cleavage, and phosphitylation. Although **2e** was unstable upon concentration and required immediate use in super-dry acetonitrile, it coupled efficiently with the trisaccharide acceptor; however, subsequent azide reduction was problematic. Donor **2f**, prepared from **18** in five steps (including azide reduction and Dmt installation), exhibited improved stability for short-term storage at low temperature and also coupled efficiently with the trisaccharide acceptor.

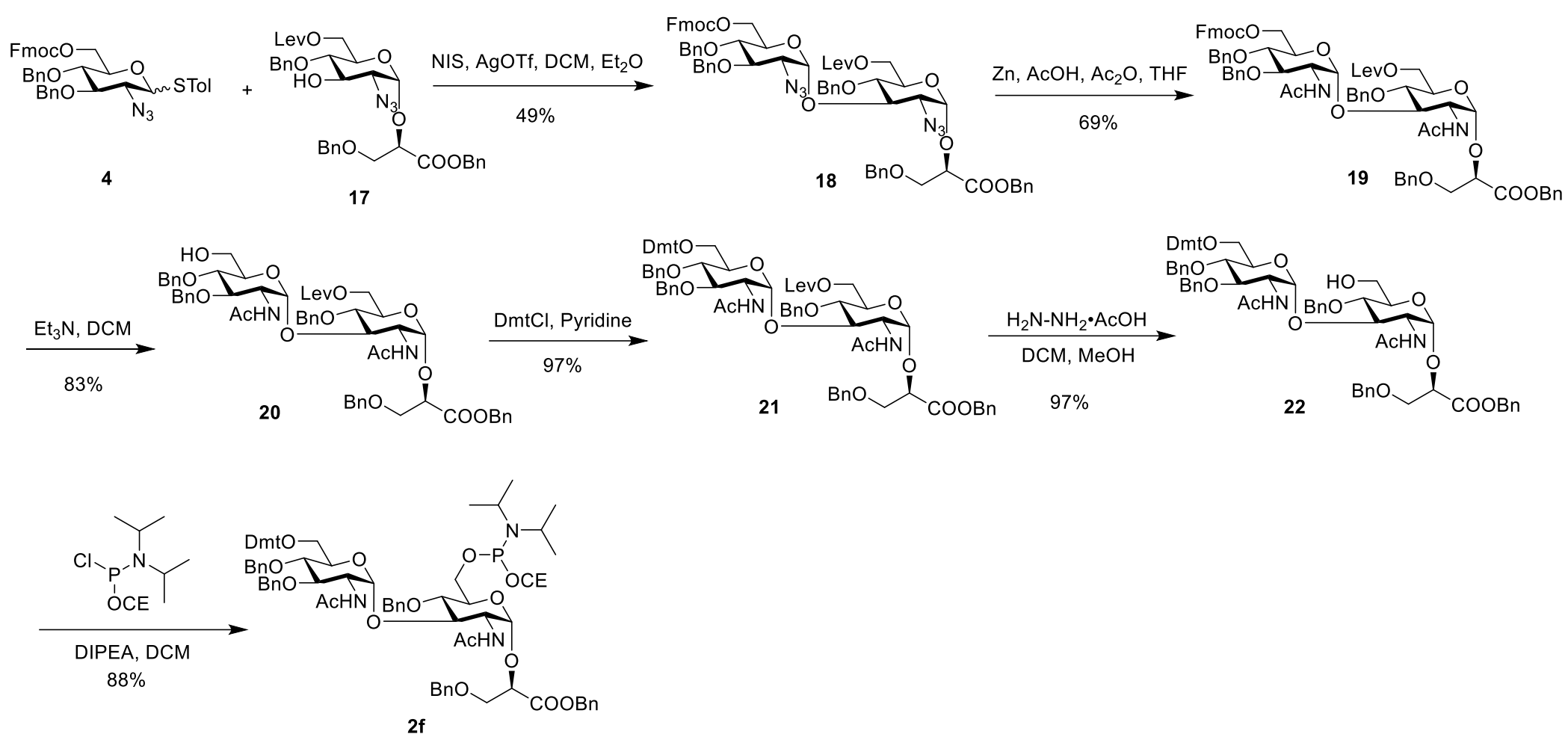

Scheme S3. Synthesis of phosphoramidite donor **2f**

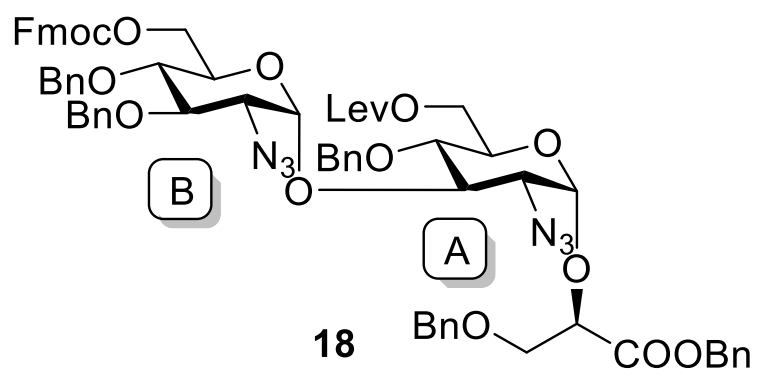

Benzyl (2*R*)-3-(benzyloxy)-2-*O*-[2-azido-4-*O*-benzyl-3-*O*-(2-azido-3,4-di-*O*-benzyl-6-*O*-(9-fluorenylmethoxycarbonyl)-2-deoxy-α-D-glucopyranosyl)-6-*O*-levulinyl-2-deoxy-α-D-glucopyranosyl]propanoate

Compounds **4** (3.23 g, 4.53 mmol) and **17** (2.00 g, 3.02 mmol) were evaporated together with toluene for 3 times and dried in *vacuo*. Dissolve the mixture in 60 mL of anhydrous DCM/Et_2O (1/1) mixture, add the freshly activated 4 Å molecular sieves, and stir at room temperature argon for 30 minutes. Cool to -40 °C and add NIS (1.16 g, 5.14 mmol) and AgOTf (466 mg, 1.81 mmol). Then raise to 0 °C and stir for 3 hours. After the acceptor reaction was completed, quenched with Pyridine, filtered, diluted with EA, washed once each with sat. aq. $Na_2S_2O_3$, H_2O, sat. aq. $NaHCO_3$ and NaCl, and the organic layer was dried with Na_2SO_4, filtered and concentrated. Column chromatography on silica gel (PE/EA = 6/1→5/1→4/1→3/1) obtained compound **18** (1.85 g, 49%) as light yellow foamy solid. TLC:PE/EA = 3/1 R_f = 0.2. ^{1}H NMR (500 MHz, Chloroform-d) δ 7.75 (dd, *J* = 7.6, 3.2 Hz, 3H, Ar), 7.60 (dd, *J* = 14.0, 7.5 Hz, 2H, Ar), 7.43-7.17 (m, 37H, Ar), 5.54 (d, *J* = 3.8 Hz, 1H, H1-B), 5.21 (d, *J* = 12.1 Hz, 1H, C*H*H-Bn), 5.19 (d, *J* = 3.8 Hz, 1H, H1-A), 5.16 (d, *J* = 12.1 Hz, 1H, C*H*H-Bn)5.03 (d, *J* = 10.8 Hz, 1H, C*H*H-Bn), 4.93 (s, 2H, CH2-Bn), 4.88 (d, *J* = 10.9 Hz, 1H, C*H*H-Bn), 4.63 (d, *J* = 10.9 Hz, 1H, C*H*H-Bn), 4.57–4.49 (m, 5H, CH2-Bn, C*H*H-Bn, CH-Gro), 4.48–4.33 (m, 4H, CH2-Fmoc, 2xH6-B), 4.31–4.19 (m, 4H, H5-B, H3-A, H6a-A, CH-Fmoc), 4.16

(m, 1H, H5-A), 4.11–4.00 (m, 2H, H3-B, H6b-A), 3.86 (dd, *J* = 10.4, 2.8 Hz, 1H, C*H*H-Gro), 3.80–3.70 (m, 2H, C*H*H-Gro, H4-B), 3.66 (dd, *J* = 10.1, 8.7 Hz, 1H, H4-A), 3.45 (dd, *J* = 10.4, 3.8 Hz, 1H, H2-B), 3.16 (dd, *J* = 10.5, 3.7 Hz, 1H, H2-A), 2.82–2.47 (m, 4H, 2xCH2-Lev), 2.16 (s, 3H, CH3-Lev). ^{13}C NMR (126 MHz, Chloroform-d) δ 206.2, 172.4, 168.9, 154.9, 143.53, 143.3, 141.3, 141.3, 137.8, 137.7, 137.5, 135.1, 128.7, 128.6, 128.6, 128.5, 128.5, 128.5, 128.4, 128.4, 128.1, 128.0, 128.0, 127.9, 127.8, 127.8, 127.8, 127.2, 127.2, 127.2, 125.3, 125.2, 120.0, 98.5 (C1-B), 96.8 (C1-A), 80.2 (C3-B), 79.1 (C4-A), 77.7 (C4-B), 75.4 (C3-A), 75.2, 74.2, 74.2, 73.5, 70.1 (C5-B), 70.0 (C6-B), 69.1 (C5-A), 67.3, 66.1, 63.5 (C2-B), 62.3 (C6-A), 61.4 (C2-A), 46.7, 37.9, 29.8, 29.7, 27.8. HRMS (ESI) *m/z*: calculated for $C_{70}H_{71}N_6O_{16}$ $[M+H]^+$: 1251.4921, found: 1251.4920.

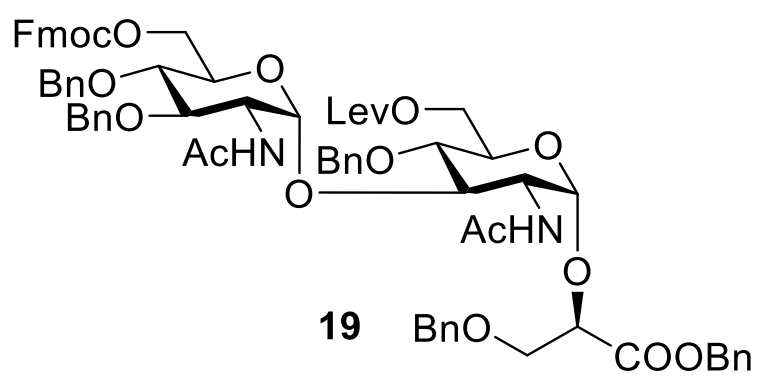

19

Benzyl (2*R*)-3-(benzyloxy)-2-*O*-[2-acetamido-4-*O*-benzyl-3-*O*-(2-acetamido-3,4-di-*O*-benzyl-6-*O*-(9-fluorenylmethoxycarbonyl)-2-deoxy-α-ᴅ-glucopyranosyl)-6-*O*-levulinyl-2-deoxy-α-ᴅ-glucopyranosyl]propanoate

Compound **18** (2.25 g, 1.80 mmol) was dissolved in a mixture of 45 mL of THF/ AcOH/ Ac_2O (4/1/2), activated zinc powder (4.68 g, 71.97 mmol) was added, and stirred vigorously overnight at room temperature. After the reaction was complete, it was filtered, diluted with DCM, washed twice with sat. aq. $NaHCO_3$, and the organic layer was dried with Na_2SO_4, filtered and concentrated. Column chromatography on silica gel (PE/EA = 2/1→1/1→1/2) obtained compound **19** (1.59 g, 69%) as white foamy solid. TLC: PE/EA = 1/2 R_f = 0.35. ^{1}H NMR (500 MHz, Chloroform-d) δ 7.78 (dd, J = 7.5, 2.3 Hz, 2H, Ar), 7.64 (dd, J = 7.6, 3.1 Hz, 2H, Ar), 7.46–7.20 (m, 34H, Ar), 6.73 (d, J = 9.3 Hz, 1H, NH-A), 6.44 (d, J = 9.9 Hz, 1H, NH-B), 5.34 (d, J = 3.6 Hz, 1H, H1-B), 5.13 (q, J = 12.1 Hz, 2H, CH2-Bn), 4.89 (dd, J = 14.5, 11.3 Hz, 2H, CH2-Bn), 4.77 (d, J = 3.5 Hz, 1H, H1-A), 4.71 (d, J = 11.5 Hz, 1H, C*H*H-Bn), 4.64 (dd, J = 10.8, 2.9 Hz, 2H, CH2-Bn), 4.54–4.42 (m, 6H, C*H*H-Bn, CH2-Bn, CH-Gro, H6a-B, H2-B), 4.41–4.34 (m, 3H, CH2-Fmoc, H6b-B), 4.30–4.22 (m, 2H, CH-Fmoc, H2-A), 4.21–3.98 (m, 5H, 2xH6-A, H3-A, H5-B, H5-A), 3.84–3.63 (m, 5H, H3-B, CH2-Gro, H4-B, H4-A), 2.77 (m, 2H, CH2-Lev), 2.67–2.55 (m, 2H, CH2-Lev), 2.19 (s, 3H, CH3-

Lev), 2.04 (s, 3H, CH3-Ac), 1.78 (s, 3H, CH3-Ac). ^{13}C NMR (126 MHz, Chloroform-d) δ 172.3, 171.4, 170.2, 170.0, 155.1, 143.4, 143.4, 141.3, 138.6, 138.1, 137.2, 136.7, 134.8, 134.1, 128.7, 128.7, 128.5, 128.4, 128.4, 128.3, 128.3, 128.1, 128.0, 127.9, 127.9, 127.8, 127.7, 127.6, 127.3, 126.4, 126.2, 125.3, 125.3, 120.0, 98.1 (C1-A, C1-B), 80.7 (C3-B), 79.0 (C4-A), 77.5 (C4-B), 75.4, 75.2, 75.1 (C3-A), 74.8, 74.6, 73.3, 70.1, 70.1, 70.0 (C5-A), 69.8 (C5-B), 67.4, 66.5 (C6-B), 62.1 (C6-A), 52.6 (C2-B), 52.0 (C2-A), 46.7, 37.9, 31.5, 31.3, 31.2, 30.2, 30.2, 29.8, 29.7, 29.7, 27.9, 23.3, 23.1, 14.1. HRMS (ESI) *m/z*: calculated for $C_{74}H_{79}N_2O_{18}$ $[M+H]^+$: 1283.5322, found: 1283.5328.

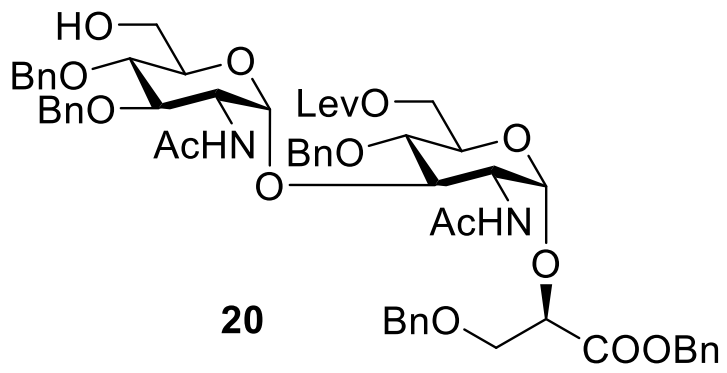

20

Benzyl (2*R*)-3-(benzyloxy)-2-*O*-[2-acetamido-4-*O*-benzyl-3-*O*-(2-acetamido-3,4-di-*O*-benzyl-6-*O*-levulinyl-2-deoxy-α-ᴅ-glucopyranosyl)-2-deoxy-α-ᴅ-glucopyranosyl]propanoate

Compound **19** (4.38 g, 3.41 mmol) was dissolved in 68 mL of DCM/Et_3N (1/1) mixed solution, stirred at room temperature for an hour, and then concentrated in *vacuo* after the reaction was complete. Column chromatography on silica gel (DCM/MeOH = 100/1→95/1→90/1→80/1) obtained compound **20** (3.00 g, 83%) as white foamy solid. TLC: PE/EA = 1/2 R_f = 0.3. ^{1}H NMR (600 MHz, Chloroform-*d*) δ 7.29 (m, *J* = 34.1, 27.3, 14.0, 6.8 Hz, 27H, Ar), 6.97 (d, *J* = 9.5 Hz, 1H, NH-A), 6.50 (d, *J* = 9.7 Hz, 1H, NH-B), 5.22 (d, *J* = 3.7 Hz, 1H, H1-B), 5.17 (d, *J* = 3.6 Hz, 2H, CH2-Bn), 4.85 – 4.74 (m, 3H, CH2-Bn, H1-A), 4.66 – 4.56 (m, 3H, CH2-Bn, C*H*H-Bn), 4.52 (d, *J* = 11.6 Hz, 2H, CH2-Bn), 4.45 (d, *J* = 11.6 Hz, 2H, C*H*H-Bn, CH-Gro), 4.31 (q, *J* = 9.7, 9.3 Hz, 2H, H2-A, H2-B), 4.22 (d, *J* = 12.3 Hz, 1H, H6a-A), 4.13 (dd, *J* = 12.3, 3.3 Hz, 1H, H6b-B), 4.02 – 3.89 (m, 3H, H5-A, H3-A, H5-B), 3.85 – 3.73 (m, 3H, H6a-B, CH2-Gro), 3.70 (t, *J* = 9.6 Hz, 1H, H3-B), 3.63 (t, *J* = 9.5 Hz, 1H, H4-A), 3.55 (dd, *J* = 11.7, 6.8 Hz, 1H, H6b-B), 3.39 (t, *J* = 9.5 Hz, 1H, H4-B), 2.87 (s, 1H, OH-B), 2.81 – 2.66 (m, 2H, CH2-Lev), 2.59 (m, 2H, CH2-Lev), 2.16 (s, 3H, , CH3-Lev), 1.99 (s, 3H, CH3-Ac), 1.69 (s, 3H, CH3-Ac). ^{13}C NMR (126 MHz, Chloroform-d) δ 206.5, 172.3, 171.8, 170.2, 169.8, 138.5, 138.1, 137.2, 136.6, 134.9, 128.8, 128.7, 128.6, 128.5, 128.4, 128.4, 128.4, 128.2, 128.0, 127.8, 127.8, 127.8, 127.7, 127.6, 98.2 (C1-B), 98.0 (C1-A), 80.9

(C3-B), 78.5 (C4-B), 78.5 (C4-A), 76.2 (C3-A), 75.2, 75.0, 75.0, 74.8, 73.4, 73.0 (C5-B), 70.1 (C5-A), 70.1, 67.5, 62.6 (C2-B), 62.2 (C2-A), 53.0, 52.2, 37.9, 29.8, 27.9, 23.3, 23.0. HRMS (ESI) *m/z*: calculated for $C_{59}H_{69}N_2O_{16}$ $[M+H]^+$: 1061.4642, found: 1061.4648.

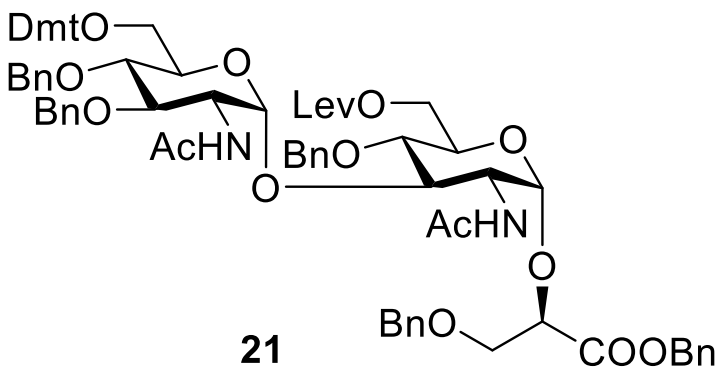

21

Benzyl (2*R*)-3-(benzyloxy)-2-*O*-[2-acetamido-4-*O*-benzyl-6-*O*-levulinyl-3-*O*-(2-acetamido-3,4-di-*O*-benzyl-6-*O*-(4,4′-dimethoxytrityl)-2-deoxy-α-ᴅ-glucopyranosyl)-2-deoxy-α-ᴅ-glucopyranosyl]propanoate

Compound **20** (2.93 g, 2.76 mmol) was dissolved in 7 mL of Pyridine, and DmtCl (3.74 g, 11.05 mmol) was added at room temperature. Stir the mixture at room temperature for 3 hours. After the reaction was complete, it was diluted with EA, washed twice with sat. aq. NaCl, and the organic layer was dried with $MgSO_4$, filtered and concentrated. Column chromatography on silica gel (DCM/MeOH = 100/1→90/1→80/1→60/1) obtained compound **21** (3.62 g, 97%) as yellow foamy solid. TLC: PE/EA = 1/2 R_f = 0.3. ^{1}H NMR (500 MHz, Chloroform-d) δ 7.50–7.45 (m, 2H), 7.40–7.09 (m, 33H), 6.90 (dd, *J* = 6.7, 2.7 Hz, 2H), 6.76–6.68 (m, 4H), 6.55 (d, *J* = 9.3 Hz, 1H), 6.46 (d, *J* = 9.8 Hz, 1H), 5.43 (d, *J* = 3.5 Hz, 1H), 5.20–5.11 (m, 2H), 4.80 (d, *J* = 11.6 Hz, 1H), 4.76 (d, *J* = 3.5 Hz, 1H), 4.69–4.59 (m, 3H), 4.50–4.40 (m, 3H), 4.37 (s, 2H), 4.30 (d, *J* = 10.9 Hz, 1H), 4.25–4.06 (m, 5H), 3.98 (m, 1H), 3.92–3.84 (m, 2H), 3.75–3.61 (m, 10H), 3.49 (dd, *J* = 10.2, 1.5 Hz, 1H), 3.25 (dd, *J* = 10.2, 3.6 Hz, 1H), 2.81–2.67 (m, 2H), 2.64–2.51 (m, 2H), 2.16 (s, 3H), 1.79 (d, *J* = 10.2 Hz, 11H). HRMS (ESI) *m/z*: calculated for $C_{80}H_{87}N_2O_{18}$ $[M+H]^+$: 1363.5948, found: 1363.5948.

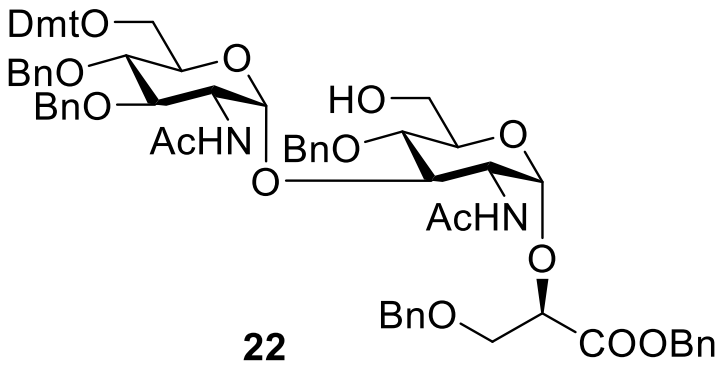

22

Benzyl (2*R*)-3-(benzyloxy)-2-*O*-[2-acetamido-4-*O*-benzyl-3-*O*-(2-acetamido-3,4-di-*O*-benzyl-6-*O*-(4,4′-dimethoxytrityl)-2-deoxy-α-ᴅ-glucopyranosyl)-2-deoxy-α-ᴅ-

glucopyranosyl]propanoate

Compound **21** (3.62 g, 2.65 mmol) was dissolved in 177 mL of DCM/MeOH(3/1) mixture, and N_2H_4·AcOH (1.05 g, 11.42 mmol), stir the mixture overnight at room temperature. After the reaction was completed, it was quenched with acetone, diluted with DCM, washed twice with sat. aq. NaCl, and the organic layer was dried with Na_2SO_4, filtered and concentrated. Column chromatography on silica gel (DCM/MeOH = 100/1→80/1→60/1→50/1) obtained compound **22** (3.23 g, 97%) as white foamy solid. TLC:PE/EA = 1/2 R_f = 0.3. ^{1}H NMR (500 MHz, Chloroform-d) δ 7.49–7.43 (m, 2H), 7.42–7.06 (m, 31H), 6.96 (d, *J* = 10.0 Hz, 1H), 6.91–6.85 (m, 2H), 6.75–6.65 (m, 5H), 5.46 (d, *J* = 3.4 Hz, 1H), 5.24–5.06 (m, 2H), 4.82–4.57 (m, 6H), 4.48–4.33 (m, 4H), 4.31–4.20 (m, 2H), 4.12 (dd, *J* = 10.5, 8.9 Hz, 1H), 3.91–3.60 (m, 15H), 3.48 (d, *J* = 10.2 Hz, 1H), 3.37 (d, *J* = 8.9 Hz, 1H), 3.23 (dd, *J* = 10.1, 2.7 Hz, 1H), 1.81 (s, 3H), 1.69 (s, 3H). ^{13}C NMR (126 MHz, Chloroform-*d*) δ 170.4, 170.1, 158.3, 138.5, 137.2, 136.3, 134.9, 130.3, 130.1, 128.8, 128.7, 128.7, 128.5, 128.3, 128.2, 128.2, 127.9, 127.9, 127.8, 127.7, 127.6, 127.5, 127.4, 127.2, 113.1, 113.1, 98.1, 85.8, 80.9, 78.0, 75.4, 75.2, 74.4, 73.4, 73.2, 72.4, 71.9, 69.9, 67.4, 55.1, 52.6, 52.3, 26.0, 23.3, 23.2. HRMS (ESI) *m/z*: calculated for $C_{75}H_{81}N_2O_{16}$ $[M+H]^+$: 1265.5581, found: 1265.5579. The NMR data of **22** is in agreement with published data.[8]

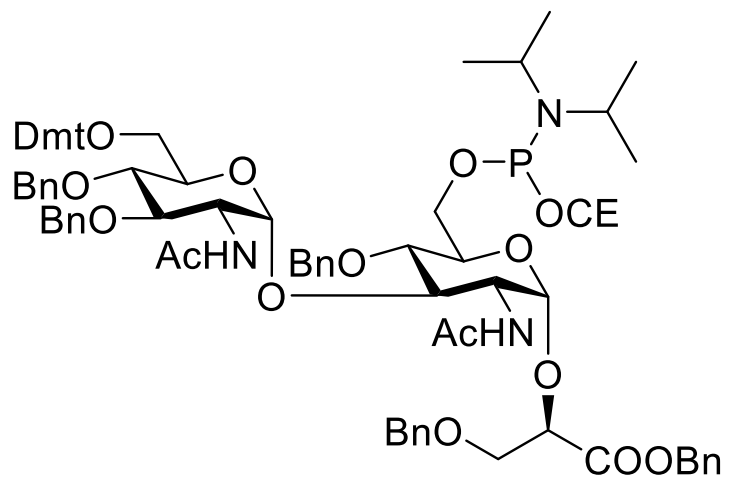

2f

Benzyl (2*R*)-3-(benzyloxy)-2-*O*-[2-acetamido-4-*O*-benzyl-6-*O*-(*N*,*N*-diisopropylaminophosphanyl)-3-*O*-(2-acetamido-3,4-di-*O*-benzyl-6-*O* -(4,4′-dimethoxytrityl)-2-deoxy-α-D-glucopyranosyl)-2-deoxy-α-D-glucopyranosyl]propanoate

Compound **22** (1.50 g, 1.18 mmol) was dissolved in 3 mL of anhydrous DCM, DIPEA (617 µL, 3.55 mmol) and 2-cyanoethyl *N*,*N*-diisopropyl chlorophosphoramidite (530 µL, 2.37 mmol) were added, and the mixture was stirred for an hour at room temperature. After the reaction was completed, column chromatography on silica gel (PE (containing 5% Et_3N)/EA = 1/0→1/1) obtained compound **2f** (1.53 g, 88%) as a white foamy solid. TLC (moistened plate with 5%

Et$_3$N):PE/EA = 1/1 R_f = 0.6. ^{1}H NMR (500 MHz, Chloroform-d) δ 7.47 (d, *J* = 7.8 Hz, 2H), 7.40–7.07 (m, 29H), 6.94-6.89 (m, 2H), 6.77–6.67 (m, 4H), 6.61 (d, *J* = 9.9 Hz, 1H), 6.54–6.45 (m, 1H), 5.42 (dd, *J* = 13.5, 3.5 Hz, 1H), 5.21–5.10 (m, 2H), 4.87–4.58 (m, 6H), 4.50–4.36 (m, 4H), 4.29 (t, *J* = 10.6 Hz, 1H), 4.19 (m, 1H), 4.15–4.02 (m, 2H), 3.96–3.52 (m, 19H), 3.47 (dd, *J* = 9.9, 6.5 Hz, 1H), 3.24 (dd, *J* = 10.3, 3.7 Hz, 1H), 2.77–2.49 (m, 3H), 1.80–1.72 (m, 6H), 1.20–1.10 (m, 12H). ^{31}P NMR (202 MHz, Chloroform-*d*) δ 149.37, 148.69. The NMR data of **2f** is in agreement with published data.[8]

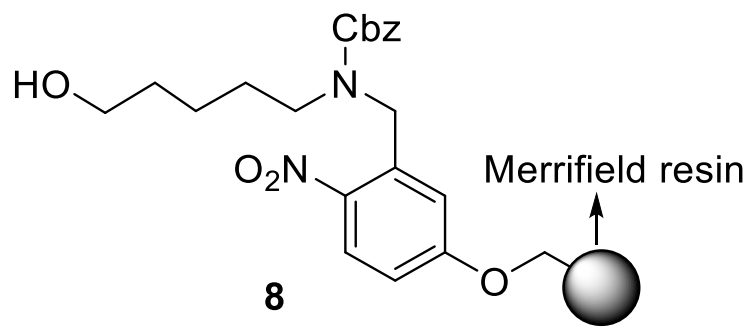

8

Similar to a previous report,[9] Merrifield resin (6.00 g, 100-200 mesh, initial loading 1.0 mmol/g) was pre-washed three times with anhydrous DCM and was swollen in 60 mL anhydrous DMF before benzyl (5-hydroxy-2-nitrobenzyl)(5-hydroxypentyl)carbamate (4.70 g, 12.00 mmol), Cs_2CO_3 (3.91 g, 12.00 mmol) and TBAI (4.43 g, 12.00 mmol) were added. The suspension was gently shaken on a rotavapor at 55 °C overnight at 650 mmbar. The mixture was filtered and the resin was washed successively with 20 mL each of THF, H_2O, THF, DMF, MeOH, DCM. The resin was swollen in anhydrous DCM for 2 hours, then DCM was concentrated. DMF (60 mL) and CsOAc (1.80 g, 9.36 mmol) were added. The suspension was gently shaken on a rotavapor at 60 °C overnight at 800 mmbar. The mixture was filtered and the resin was washed with 20 mL each of THF, H_2O, THF, DMF, MeOH, DCM. The resin **8** was dried under high *vacuo* overnight and protected from light with aluminum foil. The Loading of resin **8** (8.38 g, 92%) was 0.66 mmol/g.

Loading determination:

Similar to a previous report,[10] dry resin **8** (51 mg, theoretical loading: 1.00 mmol/g, 0.0510 mmol) was placed in a syringe (5 mL) equipped with a frit. DCM (2 - 3 mL) was added to swell the resin for an hour. The DCM was drained and a solution of FmocCl (100 mg, 0.39 mmol) and pyridine (0.10 mL, 1.24 mmol) in DCM (1 mL) was added to the resin. The reaction mixture was shaken for 6 hours, the solution was drained and the resin was washed with DCM, MeOH and DCM (six times

each). A DBU solution (2% in DMF, v/v; 2 mL) was added and the resin was shaken for an hour. The solution was drained in a vial. An aliquot of this solution (160 μL) was diluted with acetonitrile to a total volume of 10 mL and the UV absorption of this solution was measured at 294 and 304 nm. The loading of the resin was calculated as an average of the values resulting from the formulas (2): $Absorption_{304\ nm} \times 16.4$/mass of resin used in mg (for 304 nm) and $Absorption_{294\ nm} \times 14.2$/mass of resin used in mg (for 294 nm). Loading of **8**: 0.66 mmol/g.

Solid-phase synthesis of oligosaccharide 1a

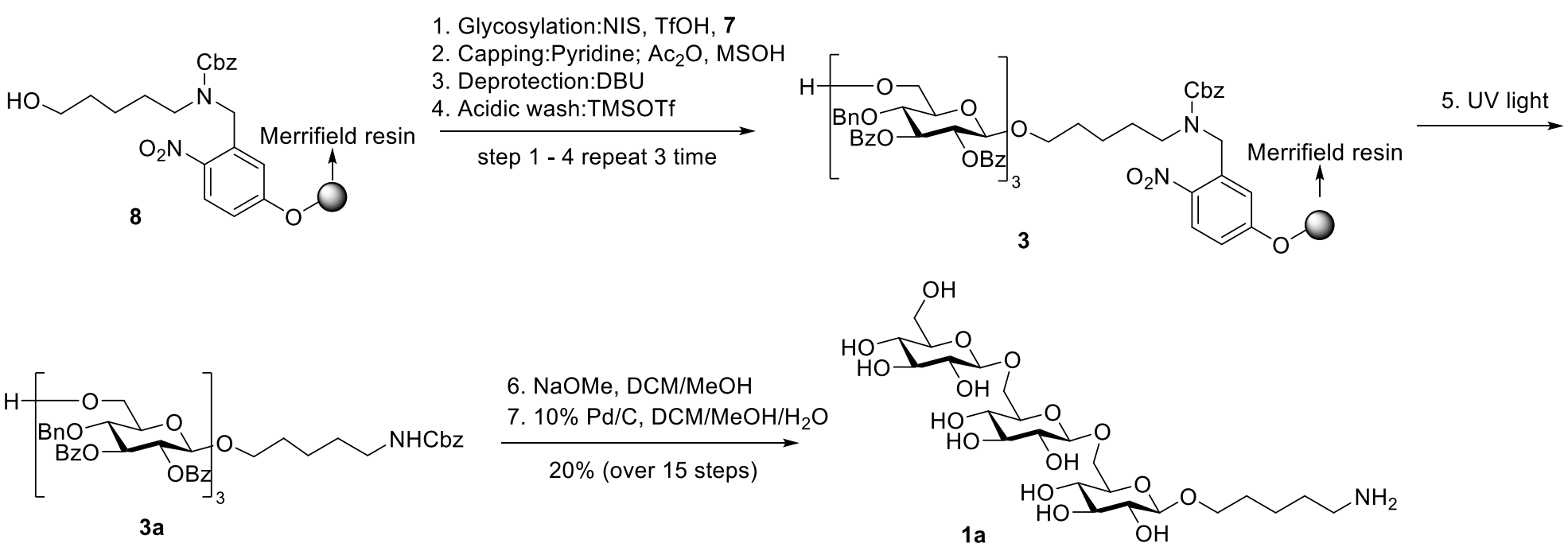

Scheme S4. Solid-phase synthesis of oligosaccharide **1a**

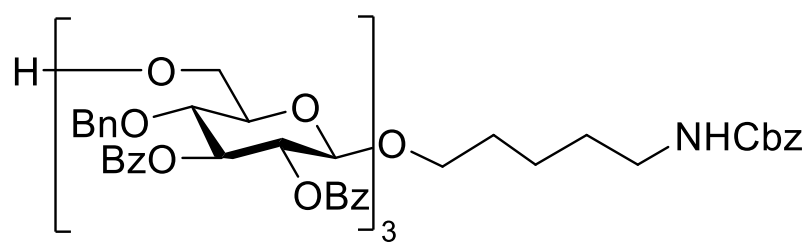

3a

5-Aminopentyl 2,3-di-*O*-benzoyl-4-*O*-benzyl-6-*O*-[2,3-di-*O*-benzoyl-4-*O*-benzyl-β-ᴅ-glucopyranosyl-(1→6)-2,3-di-*O*-benzoyl-4-*O*-benzyl-β-ᴅ-glucopyranosyl] β-ᴅ-glucopyranoside

Similar to a previous report,[10] resin **8** (2.00 g, 1.32 mmol) swelled in DMF and DCM for 20 minutes each. Repeat **Cycle A** 3 times, and the Loading of Resin **3** (4.21 g, 99%) is 0.24 mmol/g. Resin **3** (100 mg, 0.024 mmol) was placed in a reaction syringe with a sand core frit plate, irradiated by UV light at 365 nm in DCM for 24 hours, in which the filtered solution was collected at an interval of 20 minutes - 1 hour, and the resulting crude product was separated by column chromatography gel LH-20 (DCM/MeOH = 1/1) to obtain compound **3a** (27 mg, 70%). TLC:PE/EA = 2/1, R_f = 0.40. ^{1}H NMR (500 MHz, Chloroform-d) δ 8.03 – 7.80 (m, 12H), 7.56 – 7.42 (m, 6H), 7.42 – 7.27 (m, 17H), 7.18 – 7.07 (m, 12H), 6.97 – 6.88 (m, 4H), 5.75 (t, *J* = 9.6 Hz, 1H), 5.64 (t, *J*

= 9.5 Hz, 2H), 5.47 (dd, *J* = 9.9, 7.8 Hz, 1H), 5.39 (dd, *J* = 9.8, 7.8 Hz, 1H), 5.29 (dd, *J* = 9.9, 7.9 Hz, 1H), 5.06 (s, 2H), 4.80 (d, *J* = 7.8 Hz, 1H), 4.71 – 4.56 (m, 4H), 4.35 – 4.23 (m, 4H), 4.17 (dd, *J* = 11.5, 1.8 Hz, 1H), 4.12 (dd, *J* = 11.3, 1.7 Hz, 1H), 4.05 – 3.97 (m, 2H), 3.92 – 3.71 (m, 7H), 3.67 – 3.58 (m, 3H), 3.38 (q, *J* = 7.0 Hz, 1H), 2.91 (q, *J* = 6.6 Hz, 2H), 1.96 (s, 4H), 1.43 (s, 2H), 1.37 (s, 2H), 1.34 (s, 3H). HRMS (ESI) *m/z*: calculated for $C_{94}H_{92}NO_{24}$ $[M+H]^+$: 1618.6003, found: 1618.6009. The NMR data of **3a** is in agreement with published data.[10]

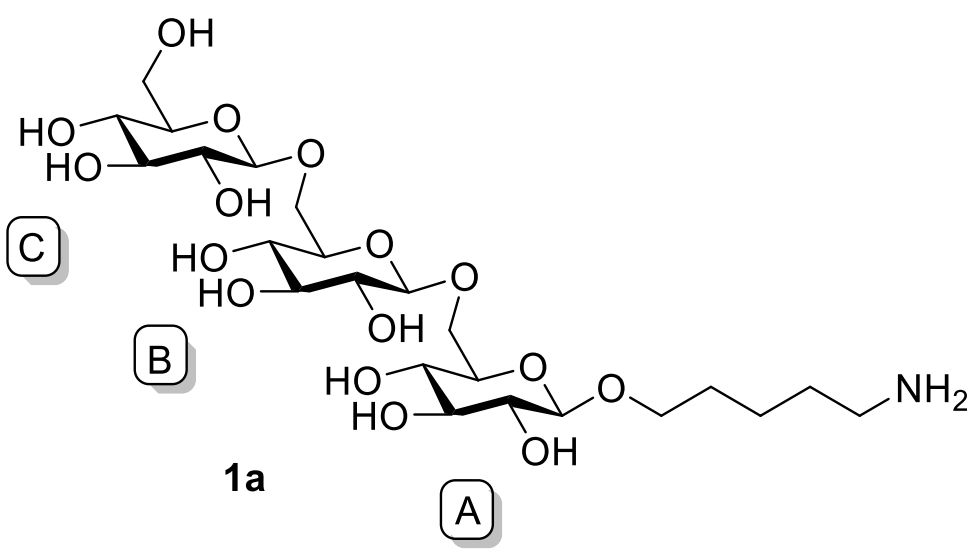

1a

Compound **3a** was dissolved in DCM/MeOH (1/1) solution, and NaOMe was added. Stir the mixture overnight at room temperature. Acidic H^+ resin was added to adjust pH = 7, filtered and concentrated. The crude product was dissolved in DCM/MeOH/H_2O (10/10/1) solution, 10% Pd/C was added under argon, and the mixture was then stirred at room temperature at H_2 for 24 hours. After filtration and concentration, the crude product was purified by a gel column HW-40F (0.1 M $NH_3{\cdot}HCO_3$ in water) to obtain compound **1a** (3.90 mg, 20% over 15 steps), which was a white solid. ^1H NMR (600 MHz, Deuterium Oxide) δ 4.44 (t, *J* = 7.8 Hz, 2H, H1-B, H1-C), 4.39 (d, *J* = 8.0 Hz, 1H, H1-A), 4.13 (dd, *J* = 2.3, 11.7 Hz, 2H, H6a-A, H6a-B), 3.89 – 3.81 (m, 2H, H6a-C, C*H*H-OCH_2), 3.82-3.74 (m, 2H, H6a-A, H6a-B), 3.63 (m, 2H, H6a-C, C*H*H-OCH_2), 3.54 (m, 2H, H5-B, H5-C), 3.40 (m, 6H, H5-A, H3-A, H3-B, H3-C, H4-A, H4-B), 3.31 (t, *J* = 9.4 Hz, 1H, H4-C), 3.24 (m, 2H, H2-B, H2-C), 3.18 (t, *J* = 8.5 Hz, 1H, H2-A), 2.92 (m, 2H, CH_2NH_2), 1.60 (q, *J* = 7.4 Hz, 4H, $OCH_2CH_2CH_2CH_2$), 1.37 (q, *J* = 7.5 Hz, 2H, $OCH_2CH_2CH_2$). HRMS (ESI) *m/z*: calculated for $C_{23}H_{44}NO_{16}$ $[M+H]^+$: 590.2655, found: 590.2652. The NMR data of **1a** is in agreement with published data.[10]

Cycle A:

(1) **Glycosylation:** Resin **8** and compound **7**[11-13] (5.30 g, 6.58 mmol) were placed in a flask, 26 mL

of anhydrous DCM was added, freshly activated 4 Å molecular sieve was added, and stirred at room temperature under argon for 20 minutes. The mixture was cooled to 0 °C and NIS (1.78 g, 7.90 mmol) and TfOH (76 μL, 0.85 mmol) were added. Stir the mixture at 0 °C for 2 hours. After the reaction was completed, the reaction suspension was filtered through a syringe with a sand core, and the resin is washed 3 times each with DCM (20 mL), DCM/dioxane (1/2, 20 mL,) and DCM (20 mL), and vacuum dried.

(2) **Capping:** Place the resin in a reaction syringe with a frit plate, add DMF solution containing 10% Pyridine after swelling, filter out the reaction solution after shaking for 1 minute, and wash the resin 3 times (20 mL, 25 seconds) with DMF and DCM. Capping solution (DCM solution with 10% Ac_2O and 2% MsOH) was added,[12] and after shaking for 20 minutes, the reaction solution was drained, and the resin was washed 3 times each with DCM and DMF (20 mL, 25 seconds).

(3) **Deprotection:** Place the resin in a reaction syringe with a frit plate, add DMF solution with 2% DBU, shake for 2 hours, then filter out the reaction and wash 3 times (20 mL, 25 seconds) with DMF and DCM.

(4) **Acidic wash:** Place the resin in a reaction syringe with a frit plate, add a DCM solution containing 1% TMSOTf, shake for 3 minutes, drain the acidic solution, and wash the resin 3 times (20 mL, 25 seconds) with DCM. Vacuum drying.

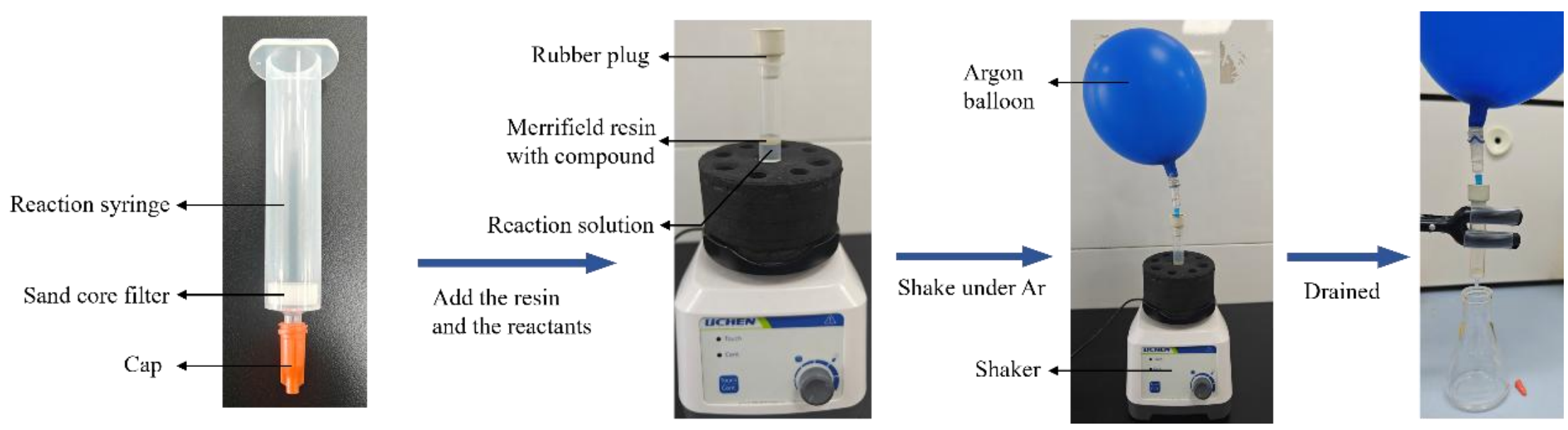

Figure S2. Solid phase synthesis device and operation

Solid-phase synthesis of oligosaccharide 24a-c

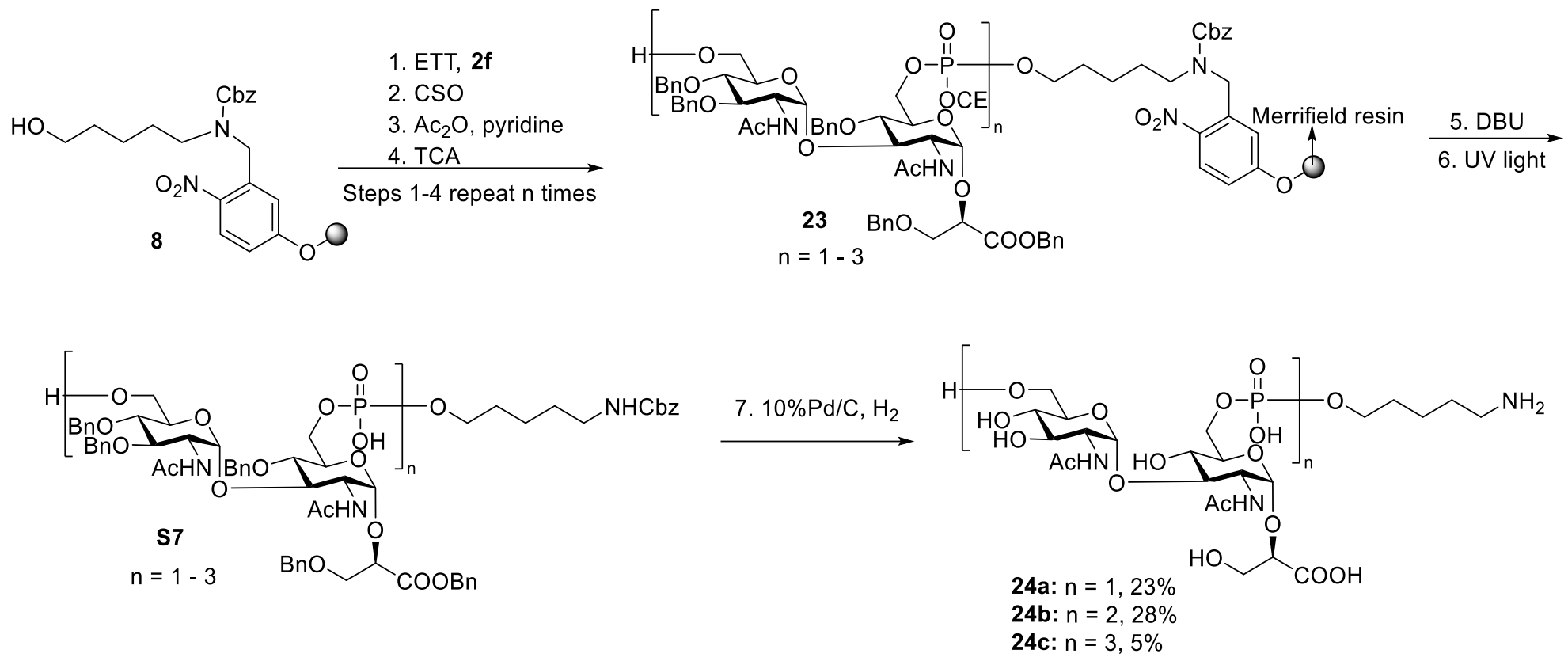

Scheme S5. Solid-phase synthesis of oligosaccharides **24a-c**

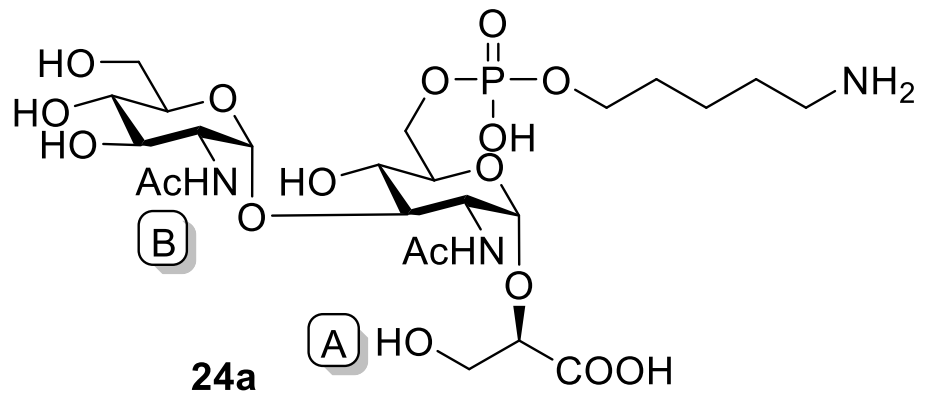

24a

Based on the solid-phase phosphoramidite method for DNA synthesis,[7] resin **8** (25 mg, 0.0170 mmol) was placed in a reaction syringe with a sand core, added to anhydrous DCM for 20 minutes to swell, and the solution was filtered out. Repeat **Cycle B** 1 time. Subsequently, ACN solution containing 5% DBU was added to the resin-containing reaction syringe, shaken twice for 15 minutes each, and the reaction solution was filtered out and washed with ACN and DCM 3 times each. Subsequently, the filtered lysate was collected at an interval of 20 minutes to 1 hour by UV light at 365 nm in DCM, and the crude product was simply separated by silica gel chromatography column method (DCM/MeOH = 1/0→4/1). Crude was dissolved in 2 mL DCM/MeOH/H_2O (10/10/1) solution and 10% Pd/C (5 M) was added under argon. Stir the mixture H_2 at room temperature for 2 days. The crude product was filtered and concentrated, and then purified by a gel column HW-40F (0.1 M NH_3·HCO_3 in water) to obtain compound **24a** (5.10 mg, 23% over 7 steps), which was a white solid. ^{1}H NMR (600 MHz, Deuterium Oxide) δ 5.38 (d, J = 3.7 Hz, 1H, H1-B), 4.92 (d, J = 3.5 Hz, 1H, H1-A), 4.26 (s, 1H, CH-Gro), 4.15 – 4.06 (m, 3H, 2xH6-A, H2-A), 4.03 – 3.98 (m, 1H, H3-A), 3.94 – 3.88 (m, 6H, H5-A, H2-B, OCH_2CH$_2$, CH2-Gro), 3.83 (m, 3H, 2xH6-B, H4-A), 3.64 (m, 2H, H5-B, H3-B), 3.54 (m, 1H, H4-B), 3.02 (m, 2H, CH_2NH$_2$), 2.06 (m, 6H, 2xCH3-Ac), 1.70 (m, 4H, OCH$_2$CH_2CH$_2$CH_2), 1.48 (m, 2H, OCH$_2$CH$_2$CH_2). ^{13}C NMR (151 MHz, Deuterium Oxide) δ 174.4 (d, J = 9.2 Hz), 174.1, 97.7 (C1-B), 96.5 (C1-A), 76.1 (C3-A), 72.2 (C3-B), 71.4 (C5-A),

70.7 (C5-B), 70.5 (C4-A), 69.3 (C4-B), 65.9 (d, J = 5.9 Hz), 63.8 (C6-A), 62.8, 60.0 (C6-B), 53.6 (C2-B), 52.0 (C2-A), 39.4, 29.1, 26.3, 22.2, 22.0 (d, J = 4.1 Hz). ^{31}P NMR (203 MHz, Deuterium Oxide) δ 1.18. HRMS (ESI) *m/z*: calculated for $C_{24}H_{43}N_3O_{17}P$ $[M-H]^-$: 676.2330, found: 676.2320. The NMR data of **24a** is in agreement with published data.[4]

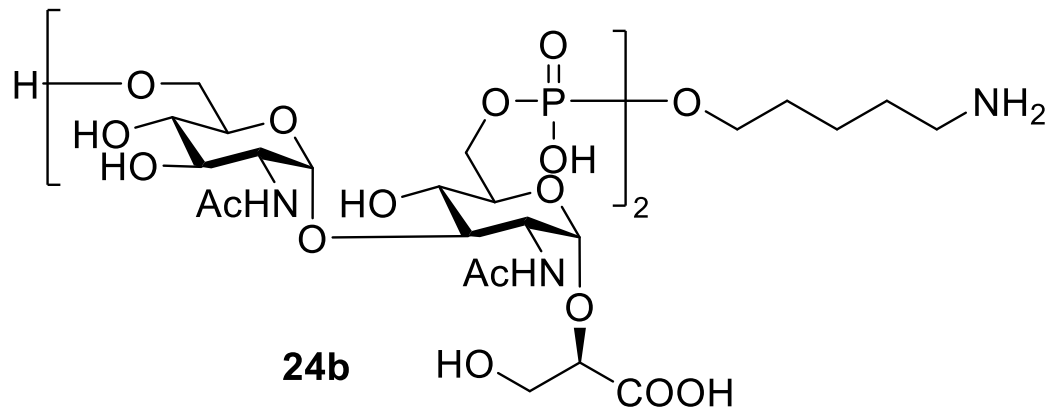

24b

Resin **8** (25 mg, 0.0170 mmol) was placed in a reaction syringe with a sand core, added to anhydrous DCM for 20 minutes to swell, and the solution was filtered out. Repeat **Cycle B** 2 times below. Subsequently, ACN solution containing 5% DBU was added to the resin-containing reaction syringe, shaken twice for 15 minutes each, and the reaction solution was filtered out and washed with ACN and DCM 3 times each. Subsequently, the filtered lysate was collected at an interval of 20 minutes to 1 hour by UV light at 365 nm in DCM, and the crude product was simply separated by silica gel chromatography column method (DCM/MeOH = 1/0→4/1). Crude was dissolved in 2 mL DCM/MeOH/H_2O (10/10/1) solution and 10% Pd/C (5 M) was added under argon. Stir the mixture under H_2 at room temperature for 2 days. The crude product was filtered and concentrated, and then purified by a gel column HW-40F (0.1 M $NH_3{\cdot}HCO_3$ in water) to obtain compound **24b** (5.70 mg, 28% over 11 steps), which was a white solid. ^{1}H NMR (800 MHz, Deuterium Oxide) δ 5.36 (d, J = 3.4 Hz, 2H, H1-B, B′), 4.95 (s, 2H, H1-A, A′), 4.27 (s, 2H, 2xCH-Gro), 4.21 – 4.05 (m, 9H), 4.02 – 3.88 (m, 11H), 3.87 – 3.82 (m, 3H), 3.80 (d, J = 9.0 Hz, 1H), 3.71 (d, J = 8.5 Hz, 1H), 3.68 – 3.60 (m, 4H), 3.53 (t, J = 9.4 Hz, 1H), 3.01 (t, J = 7.4 Hz, 2H, CH_2NH$_2$), 2.15 – 2.01 (m, 12H, 4xCH3-Ac), 1.73-1.66 (m, 4H OCH$_2$CH_2CH$_2$CH_2), 1.50-1.45 (m, 2H, OCH$_2$CH$_2$CH_2). ^{13}C NMR (201 MHz, Deuterium Oxide) δ 174.3 (d, J = 11.2 Hz), 97.9 (C1-B), 97.8 (C1-B′), 96.6 (C1-A), 96.3 (C1-A′), 76.6, 76.3, 72.2, 71.4, 70.7, 70.5 (d, J = 23.4 Hz), 70.3, 69.4, 69.0, 65.9, 63.9 (d, J = 33.1 Hz), 62.4, 60.1, 53.6 (d, J = 11.8 Hz), 52.0, 39.4, 29.1, 26.3, 22.3 (d, J = 14.9 Hz), 22.0 (d, J = 5.1 Hz). ^{31}P NMR (203 MHz, Deuterium Oxide) δ 2.54. HRMS (ESI) *m/z*: calculated for $C_{43}H_{76}N_5O_{33}P_2$ $[M+H]^+$: 1252.3892, found: 1252.3896. The NMR data of **24b** is in agreement with

published data.[4]

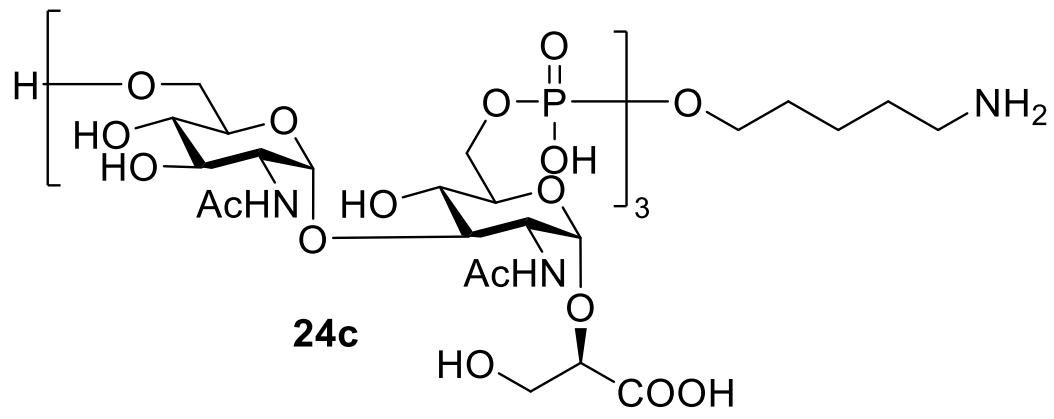

24c

Resin **8** (25 mg, 0.0170 mmol) was placed in a reaction syringe with a sand core, added to anhydrous DCM for 20 minutes to swell, and the solution was filtered out. Repeat **Cycle B** 3 times below. Subsequently, ACN solution containing 5% DBU was added to the resin-containing reaction syringe, shaken twice for 15 minutes each, and the reaction solution was filtered out and washed with ACN and DCM 3 times each. Subsequently, the filtered lysate was collected at an interval of 20 minutes to 1 hour by UV light at 365 nm in DCM, and the crude product was simply separated by silica gel chromatography column method (DCM/MeOH = 1/0→4/1). Crude was dissolved in 2 mL DCM/MeOH/H_2O (10/10/1) solution and 10% Pd/C (5 M) was added under argon. Stir the mixture under H_2 at room temperature for 2 days. The crude product was filtered and concentrated, and then purified by a gel column HW-40F (0.1 M $NH_3 \cdot HCO_3$ in water) to obtain compound **24c** (3.10 mg, 5% over 15 steps), which was a white solid. ^{1}H NMR (800 MHz, Deuterium Oxide) δ 5.40 – 5.30 (m, 3H, H1-B, B′, B′′), 5.02 – 4.93 (m, 3H, H1-A, A′, A′′), 4.35 (s, 3H, 3xCH-Gro), 4.24 – 4.06 (m, 12H), 4.04 – 3.88 (m, 15H), 3.87 – 3.82 (m, 4H), 3.82 – 3.77 (m, 2H), 3.71 (d, *J* = 9.2 Hz, 2H), 3.69 – 3.58 (m, 6H), 3.53 (t, *J* = 9.5 Hz, 1H), 3.01 (t, *J* = 7.5 Hz, 2H, CH_2NH_2), 2.25 – 1.95 (m, 18H, 6xCH3-Ac), 1.74 – 1.66 (m, 4H $OCH_2CH_2CH_2CH_2$), 1.50 – 1.45 (m, 2H, $OCH_2CH_2CH_2$). ^{13}C NMR (201 MHz, Deuterium Oxide) δ 174.3, 98.2 (C1-B), 97.9 (C1-B′), 97.8 (C1-B′′), 96.6 (C1-A), 96.4 (C1-A′), 96.2 (C1-A′′), 76.6, 76.3, 72.2, 71.3, 70.6 (m), 70.3, 69.4, 69.6, 65.9, 63.9, 62.6, 60.1, 53.6 (d, *J* = 12.4 Hz), 52.0, 39.4, 29.1 (d, *J* = 6.7 Hz), 26.3, 22.2 (d, *J* = 15.0 Hz), 22.0 (d, *J* = 6.8 Hz). ^{31}P NMR (203 MHz, Deuterium Oxide) δ 1.48. HRMS (ESI) *m/z*: calculated for $C_{62}H_{107}N_7O_{49}P_3$ $[M+H]^+$: 1826.5303, found: 1826.5347.

Cycle B:

(1) **Coupling:** ETT (9 mg, 0.0330 mmol) and reactant **2f** (48 mg, 0.0330 mmol) solution (0.08 M

in anhydrous DCM) were added to the resin, and the mixture was shaken under argon gas at room temperature for an hour, and the reaction solution was filtered out; The coupling step was repeated one more time, filtered out the reaction solution; Wash the resin 3 times with anhydrous DCM under N_2.

(2) **Oxidation:** CSO solution (0.5 M in DCM) (1.20 mL, 1.2000 mmol) was added to the reaction syringe and shaken at argon at room temperature for 15 minutes to filter out the reaction solution; The oxidate step was repeated one more time, filter out the reaction solution; Wash the resin 3 times with anhydrous DCM and ACN under N_2.

(3) **Capping:** Cap A solution (AC_2O) and Cap B solution (acetonitrile/*N*-methylimidazole/pyridine) were added to the reaction syringe in a 1:1 ratio (2 mL), and after shaking for 2 hours, the solution was filtered out and washed 3 times each with ACN and DCM.

(4) **Dmt-Deprotection:** TCA solution (3% in DCM) was added to the reaction syringe, shaken for 25 seconds, and the reaction solution was filtered; The deprotection step was repeated 3 times, and the reaction solution was filtered out and washed 6 times each with DCM, ACN and DCM, and vacuum dried.

Solid-phase synthesis of oligosaccharide 1b-f

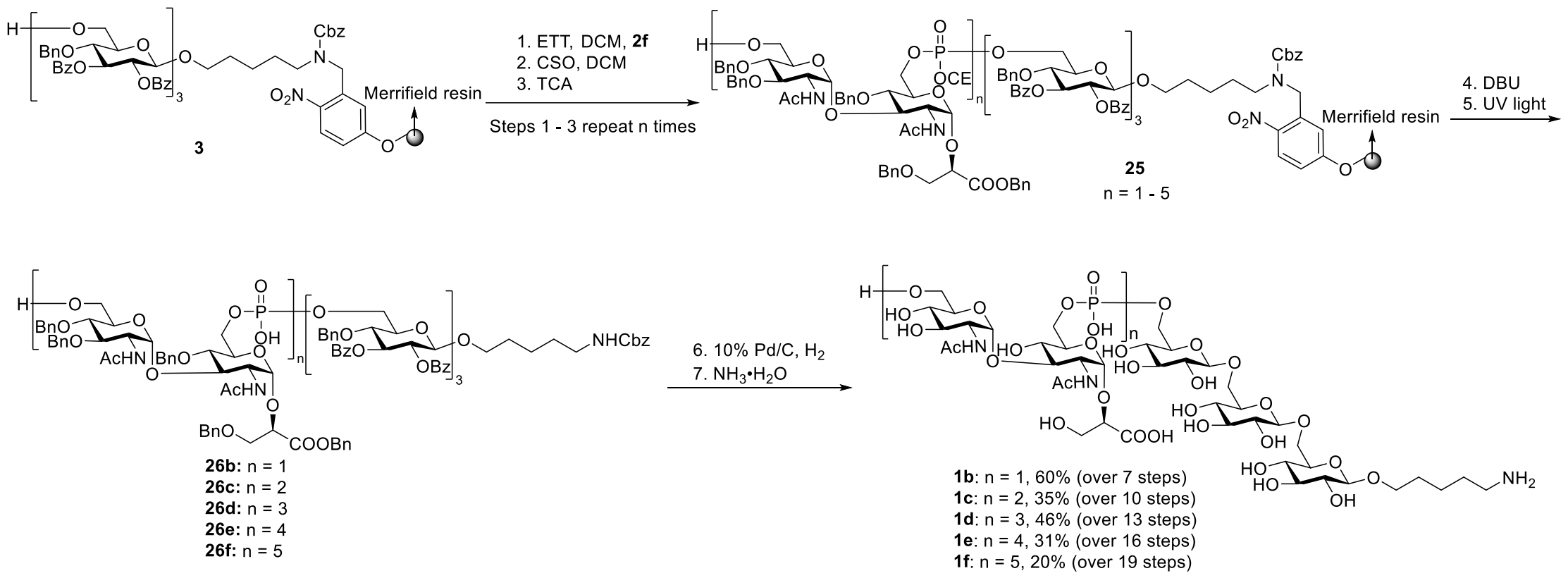

Scheme S6. Solid-phase synthesis of oligosaccharide **1b-1f**

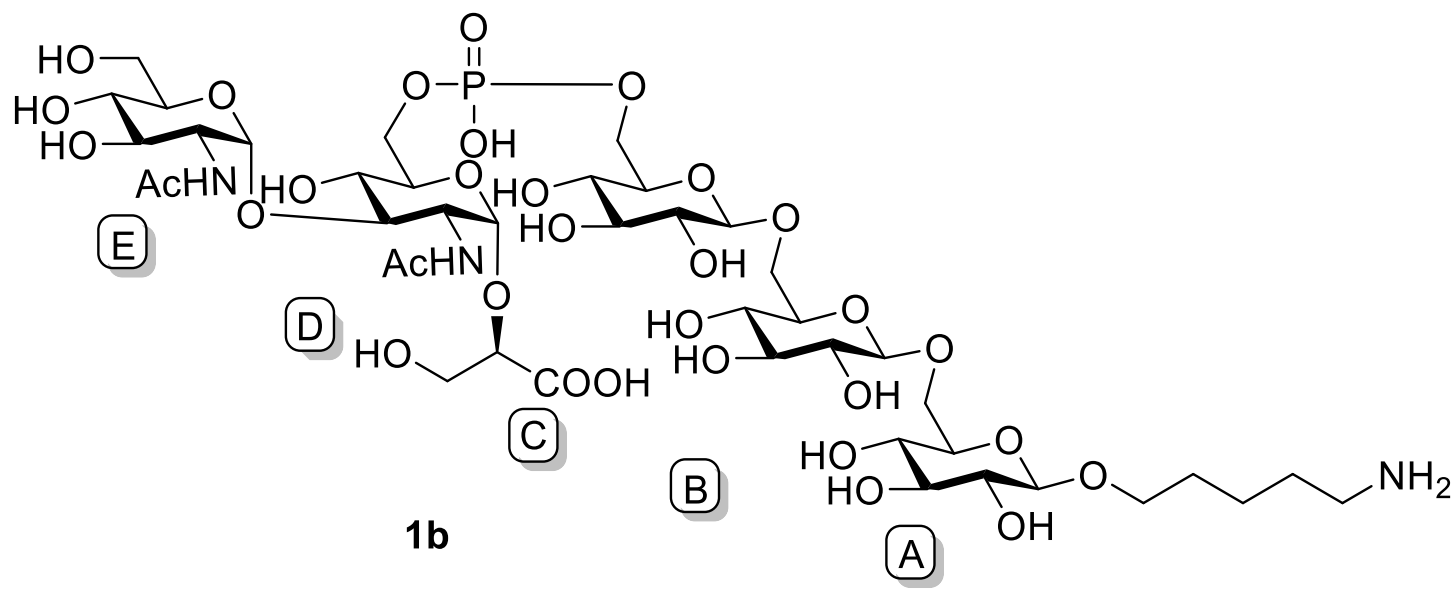

1b

Resin **3** (50 mg, 0.0120 mmol) was placed in a reaction syringe with a sand core, and the solution was filtered out by adding anhydrous DCM to swell for 20 minutes. Repeat the following **Cycle C** 1 time. Subsequently, ACN solution containing 5% DBU was added to the resin, shaken twice for 15 minutes each, and the reaction solution was filtered out and washed with ACN and DCM 3 times each. Subsequently, the filtered lysate was collected at intervals of 20 minutes to 1 hour by UV light at 365 nm in DCM, and the resulting crude product **26b** was dissolved in 2 mL DCM/MeOH/H_2O (10/10/1) solution and 10% Pd/C (5 M) was added under argon. Stir the mixture under H_2 at room temperature for 2 days. The mixture was filtered and concentrated, then the crude product was stirred in ammonium hydroxide ($NH_3{\cdot}H_2O$) overnight. Concentrated and purified by a gel column HW-40F (0.1 M $NH_3{\cdot}HCO_3$ in water) to obtain compound **1b** (8.42 mg, 60% over 7 steps), which was a white solid. ^{1}H NMR (500 MHz, Deuterium Oxide) δ 5.40 (d, *J* = 3.6 Hz, 1H, H1-E), 4.88 (d, *J* = 3.2 Hz, 1H, H1-D), 4.53 (dd, *J* = 3.3, 8.0 Hz, 2H, 2H, H1-B, H1-C), 4.47 (d, *J* = 8.0 Hz, 1H, H1-A), 4.26 – 3.77 (m, 19H, H2-D, 2xH6-D, H3-D, H4-D, CH-Gro, H6a-B, H6a-A, OC*H*HCH_2, H2-E, H4-D, H6b-A, H6b-B, H6a-C, 2xH6-E, CH2-Gro, H5-E), 3.74 – 3.44 (m, 13H, H3-E, OC*H*HCH_2, H5-B, H5-C, H6b-C, H4-E, H3-A, H4-A, H5-A, H3-B, H4-B, H3-C, H4-C), 3.36 – 3.24 (m, 3H, H2-B, H2-C, H2-A), 3.02 (t, *J* = 7.6 Hz, 2H, CH_2NH_2), 2.07 (s, 6H, 2xCH3-Ac), 1.68 (m, *J* = 7.1, 14.1 Hz, 4H, OCH_2CH_2CH_2CH_2), 1.47 (q, *J* = 7.8 Hz, 2H, OCH_2CH_2CH_2). ^{13}C NMR (151 MHz, Deuterium Oxide) δ 174.4, 174.3, 103.1 (C1-B), 102.8 (C1-C), 102.2 (C1-A), 97.6 (C1-E), 96.7 (C1-D), 76.0, 75.7, 75.6, 75.4, 75.0, 74.8, 74.7, 73.1, 72.2, 71.3, 70.8, 70.4, 70.2, 69.5, 69.4, 69.3, 69.2, 68.8, 68.7, 64.3, 63.8, 63.1, 60.0, 53.6, 52.0, 39.4, 28.2, 26.4, 22.3, 22.1, 22.0. ^{31}P NMR (202 MHz, Deuterium Oxide) δ 1.09. HRMS (ESI) *m/z*: calculated for $C_{42}H_{73}N_3O_{32}P$ [M-H]$^-$: 1162.3909, found: 1162.3917, [M-2H/2]$^-$: 580.6913, found: 580.6921.

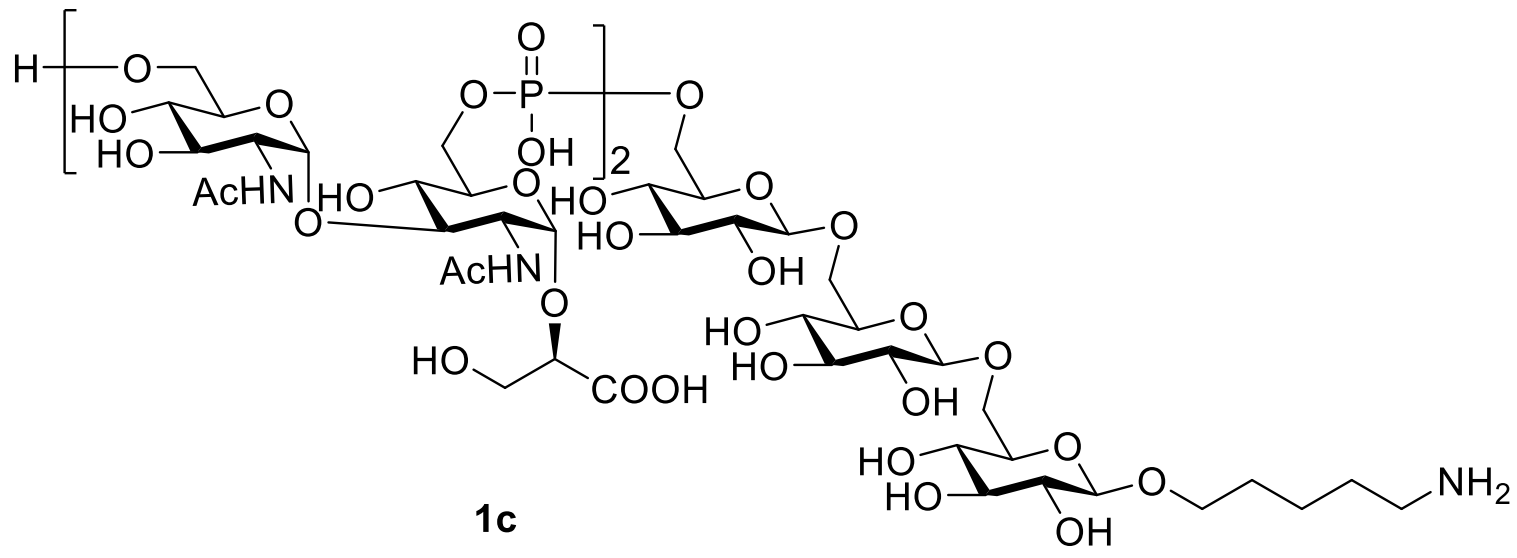

1c

Resin **3** (50 mg, 0.0120 mmol) was placed in a reaction syringe with a sand core, and the solution was filtered out by adding anhydrous DCM to swell for 20 minutes. Repeat the following **Cycle C** 2 times. Subsequently, ACN solution containing 5% DBU was added to the resin, shaken twice for 15 minutes each, and the reaction solution was filtered out and washed with ACN and DCM 3 times each. Subsequently, the filtered lysate was collected at intervals of 20 minutes to 1 hour by UV light at 365 nm in DCM, and the resulting crude product **26c** was dissolved in 2 mL DCM/MeOH/H_2O (10/10/1) solution and 10% Pd/C (5 M) was added under argon. Stir the mixture under H_2 at room temperature for 2 days. The mixture was filtered and concentrated, then the crude product was stirred in $NH_3{\cdot}H_2O$ overnight. Concentrated and purified by a gel column HW-40F (0.1 M $NH_3{\cdot}HCO_3$ in water) to obtain compound **1c** (7.28 mg, 35% over 10 steps), which was a white solid. ^{1}H NMR (500 MHz, Deuterium Oxide) δ 5.39 (d, J = 3.4 Hz, 2H, H1-E, E′), 4.91 (d, J = 23.9 Hz, 2H, H1-D, D′), 4.54 (d, J = 8.0 Hz, 2H, H1-B, H1-C), 4.48 (d, J = 8.2 Hz, 1H, H1-A), 4.32 – 3.76 (m, 30H), 3.76 – 3.41 (m, 17H), 3.38 – 3.23 (m, 3H), 3.02 (t, J = 7.7 Hz, 2H, 2H, CH_2NH$_2$), 2.25 – 1.90 (m, 12H, 4xCH3-Ac), 1.69 (m, J = 7.2, 13.3 Hz, 4H, OCH$_2$CH_2CH$_2$CH_2), 1.47 (q, J = 7.9 Hz, 2H, OCH$_2$CH$_2$CH_2).^{13}C NMR (151 MHz, Deuterium Oxide) δ 174.3, 103.1 (C1-B), 102.8 (C1-C), 102.2 (C1-A), 97.7 (C1-E, E′), 96.9 (C1-D), 96.4 (C1-D′), 75.7, 75.6, 75.4, 74.9, 74.8, 73.1, 72.2, 71.3, 70.8, 70.5, 70.2, 69.5, 69.3, 69.2, 68.7, 63.9, 63.1, 62.4, 60.0, 53.6, 52.1, 39.4, 28.2, 26.4, 22.1. ^{31}P NMR (202 MHz, Deuterium Oxide) δ 1.16. HRMS (ESI) *m/z*: calculated for $C_{61}H_{104}N_5O_{48}P_2$ [M-H]$^-$: 1736.5320, found: 1736.5347, [M-2/2]$^-$: 867.7618, found: 867.7657.

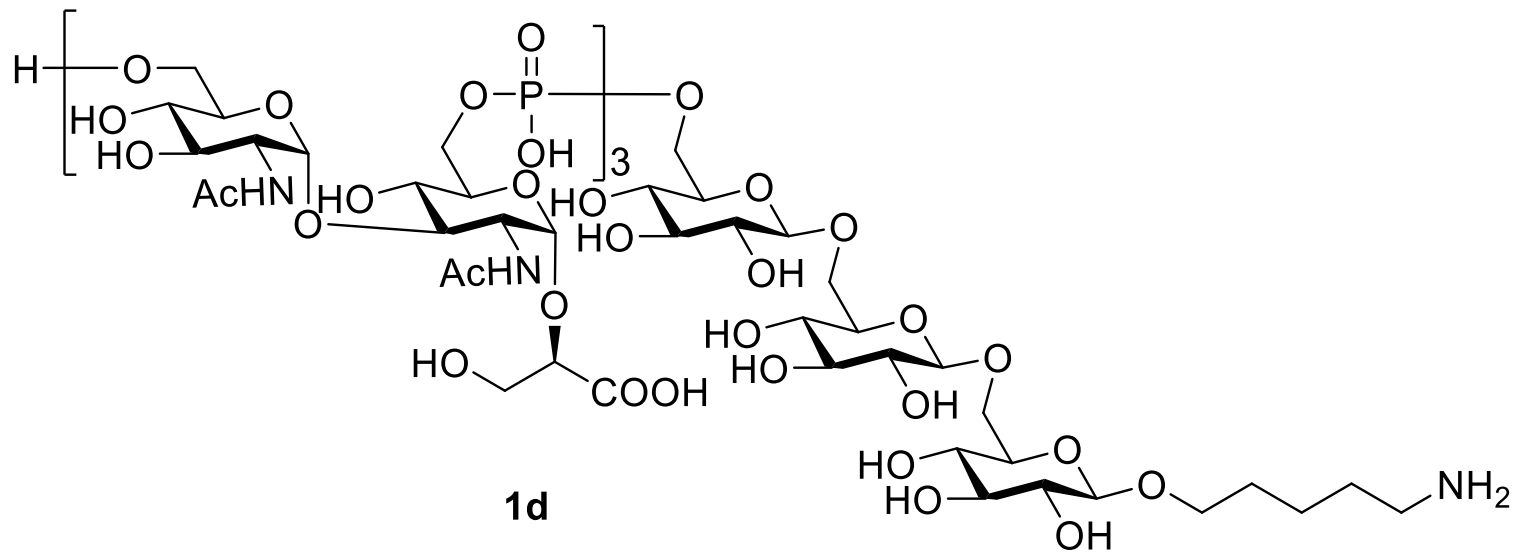

1d

1d

Resin **3** (50 mg, 0.0120 mmol) was placed in a reaction syringe with a sand core, and the solution was filtered out by adding anhydrous DCM to swell for 20 minutes. Repeat the following **Cycle C** 3 times. Subsequently, ACN solution containing 5% DBU was added to the resin, shaken twice for 15 minutes each, and the reaction solution was filtered out and washed with ACN and DCM 3 times each. Subsequently, the filtered lysate was collected at intervals of 20 minutes to 1 hour by UV light at 365 nm in DCM, and the resulting crude product **26d** was dissolved in 2 mL DCM/MeOH/H_2O (10/10/1) solution and 10% Pd/C (5 M) was added under argon. Stir the mixture under H_2 at room temperature for 2 days. The mixture was filtered and concentrated, then the crude product was stirred in $NH_3{\cdot}H_2O$ overnight. Concentrated and purified by a gel column HW-40F (0.1 M $NH_3{\cdot}HCO_3$ in water) to obtain compound **1d** (12.80 mg, 46% over 13 steps), which was a white solid. ^{1}H NMR (500 MHz, Deuterium Oxide) δ 5.45 – 5.30 (m, 3H, H1-E, E′, E′′), 4.92 (d, *J* = 14.9 Hz, 3H, H1-D, D′, D′′), 4.56 – 4.50 (m, 2H, H1-B, H1-C), 4.47 (d, *J* = 7.8 Hz, 1H, H1-A), 4.33 – 3.77 (m, 42H), 3.77 – 3.41 (m, 20H), 3.37 – 3.24 (m, 3H), 3.02 (t, *J* = 7.5 Hz, 2H, CH_2NH_2), 2.07 (d, *J* = 8.1 Hz, 18H, 6xCH3-Ac), 1.68 (m, *J* = 7.0, 13.0, 13.7 Hz, 4H, $OCH_2CH_2CH_2CH_2$), 1.52 – 1.40 (m, 2H, $OCH_2CH_2CH_2$). ^{13}C NMR (151 MHz, Deuterium Oxide) δ 174.3 (d, *J* = 10.8 Hz), 103.1 (C1-B), 102.8 (C1-C), 102.2 (C1-A), 99.6 – 95.7 (m, C1-E, E′, E′′, C1-D, D′, D′′), 76.2, 75.9 – 75.2 (m), 75.2 – 74.1 (m), 73.1 (d, *J* = 5.3 Hz), 72.2, 72.0 – 69.8 (m), 69.7 – 68.1 (m), 64.1 (d, *J* = 62.4 Hz), 62.6 (d, *J* = 75.2 Hz), 60.0, 53.6, 52.0 (d, *J* = 16.4 Hz), 39.3, 28.2, 26.4, 22.5 – 21.9 (m). ^{31}P NMR (202 MHz, Deuterium Oxide) δ 1.12. HRMS (ESI) *m/z*: calculated for $C_{80}H_{136}N_7O_{67}P_3$ $[M-2/2]^-$: 1154.8324, found: 1154.8384, $[M-3/3]^-$: 769.5521, found: 769.5599.

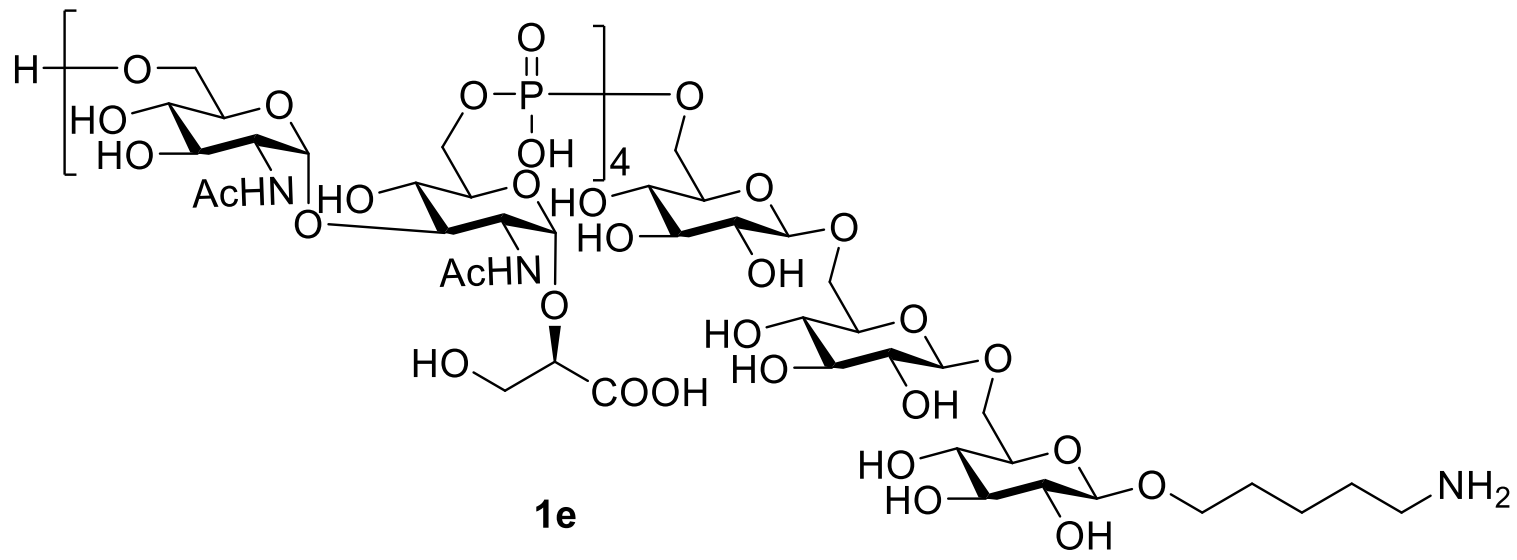

1e

Resin **3** (50 mg, 0.0120 mmol) was placed in a reaction syringe with a sand core, and the solution was filtered out by adding anhydrous DCM to swell for 20 minutes. Repeat the following **Cycle C** 4 times. Subsequently, ACN solution containing 5% DBU was added to the resin, shaken twice for 15 minutes each, and the reaction solution was filtered out and washed with ACN and DCM 3 times each. Subsequently, the filtered lysate was collected at intervals of 20 minutes to 1 hour by UV light at 365 nm in DCM, and the resulting crude product **26e** was dissolved in 2 mL DCM/MeOH/H_2O (10/10/1) solution and 10% Pd/C (5 M) was added under argon. Stir the mixture under H_2 at room temperature for 2 days. The mixture was filtered and concentrated, then the crude product was stirred in $NH_3{\cdot}H_2O$ overnight. Concentrated and purified by a gel column HW-40F (0.1 M $NH_3{\cdot}HCO_3$ in water) to obtain compound **1e** (10.60 mg, 31% over 16 steps), which was a white solid. ^{1}H NMR (500 MHz, Deuterium Oxide) δ 5.45 – 5.30 (m, 4H, H1-E, E′, E′′, E′′′), 4.93 (d, *J* = 3.7 Hz, 4H, H1-D, D′, D′′, D′′′), 4.53 (dd, *J* = 1.8, 8.0 Hz, 2H, H1-B, H1-C), 4.47 (d, *J* = 8.0 Hz, 1H, H1-A), 4.31 – 3.80 (m, 61H), 3.76 – 3.44 (m, 26H), 3.33 (q, *J* = 7.8, 8.4 Hz, 2H), 3.27 (t, *J* = 8.5 Hz, 1H, 2H, C*H*$_2$NH$_2$), 3.02 (t, *J* = 7.5 Hz, 2H), 2.08 (d, *J* = 10.1 Hz, 24H, 8xCH3-Ac), 1.75 – 1.62 (m, 4H, OCH$_2$C*H*$_2$CH$_2$C*H*$_2$), 1.47 (m, *J* = 6.7, 11.0 Hz, 2H, OCH$_2$CH$_2$C*H*$_2$). ^{13}C NMR (151 MHz, Deuterium Oxide) δ 175.2 – 173.8 (m), 103.1 (C1-B), 102.8 (C1-C), 102.2 (C1-A), 98.0 – 96.4 (d, *J* = 146.2 Hz, C1-E, E′, E′′, E′′′, C1-D, D′, D′′, D′′′), 78.6 – 74.1 (m), 73.1 (d, *J* = 5.3 Hz), 72.2, 72.1 – 69.7 (m), 69.7 – 68.1 (m), 64.1 (d, *J* = 58.1 Hz), 62.6 (d, *J* = 57.2 Hz), 60.0, 53.6, 52.0 (d, *J* = 11.1 Hz), 39.3, 28.2, 26.4, 22.5 – 21.9 (m). ^{31}P NMR (202 MHz, Deuterium Oxide) δ 1.14. HRMS (ESI) *m/z*: calculated for $C_{99}H_{167}N_9O_{80}P_4$ $[M+2/2]^+$: 1443.9191, found:1443.9288.

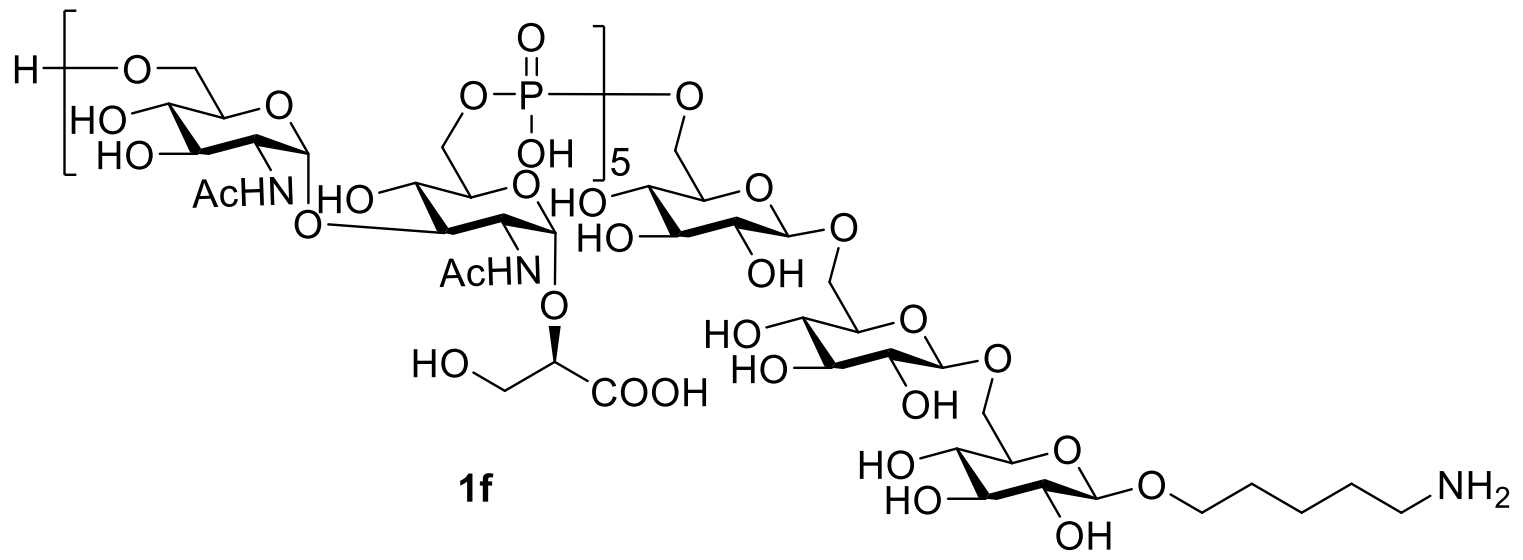

1f

1f

Resin **3** (100 mg, 0.0240 mmol) was placed in a reaction syringe with a sand core, and the solution was filtered out by adding anhydrous DCM to swell for 20 minutes. Repeat the following **Cycle C** 5 times. Subsequently, ACN solution containing 5% DBU was added to the resin, shaken twice for 15 minutes each, and the reaction solution was filtered out and washed with ACN and DCM 3 times each. Subsequently, the filtered lysate was collected at intervals of 20 minutes to 1 hour by UV light at 365 nm in DCM, and the resulting crude product **26f** was dissolved in 2 mL DCM/MeOH/H_2O (10/10/1) solution and 10% Pd/C (5 M) was added under argon. Stir the mixture under H_2 at room temperature for 2 days. The mixture was filtered and concentrated, then the crude product was stirred in $NH_3{\cdot}H_2O$ overnight. Concentrated and purified by a gel column HW-40F (0.1 M $NH_3{\cdot}HCO_3$ in water) to obtain compound **1f** (16.40 mg, 20% over 19 steps), which was a white solid. ^{1}H NMR (500 MHz, Deuterium Oxide) δ 5.47 – 5.27 (m, 5H, H1-E, E′, E″, E‴, E⁗), 5.00 – 4.91 (m, 5H, H1-D, D′, D″, D‴, D⁗), 4.57 – 4.52 (m, 2H, H1-B, H1-C), 4.48 (s, 1H, H1-A), 4.32 – 3.79 (m, 65H), 3.75 – 3.44 (m, 27H), 3.33 (q, *J* = 7.9, 8.4 Hz, 2H), 3.27 (t, *J* = 8.2 Hz, 1H), 3.02 (t, *J* = 7.5 Hz, 2H, C*H*$_2$NH$_2$), 2.09 (d, *J* = 20.0 Hz, 30H, 10xCH3-Ac), 1.74 – 1.64 (m, 4H, OCH$_2$C*H*$_2$CH$_2$C*H*$_2$), 1.47 (q, *J* = 6.8, 7.4 Hz, 2H, OCH$_2$CH$_2$C*H*$_2$). ^{13}C NMR (151 MHz, Deuterium Oxide) δ 174.4, 103.1 (C1-B), 102.8 (C1-C), 102.2 (C1-A), 98.5 – 96.5 (m, , C1-E, E′, E″, E‴, E⁗, C1-D, D′, D″, D‴, D⁗), 76.7 (d, *J* = 43.3 Hz), 75.9 – 75.3 (m), 75.1 – 74.4 (m), 73.1, 72.2, 72.0 – 69.6 (m), 69.6 – 68.1 (m), 64.1 (d, *J* = 58.0 Hz), 62.4, 53.6, 52.0, 39.4, 28.2, 26.4, 22.2 (d, *J* = 42.0 Hz). ^{31}P NMR (202 MHz, Deuterium Oxide) δ 1.18. HRMS (ESI) *m/z*: calculated for $C_{118}H_{198}N_{11}O_{96}P_5$ [M-2/2]$^-$: 1728.9741, found: 1728.9954.

Cycle C:

(1) **Coupling:** ETT (13 mg, 0.096 mmol) and **2f** (71 mg, 0.0480 mmol) solution (0.08 M in DCM) were added to the resin, the mixture was shaken under argon at room temperature for 30 minutes

to 1 hour, and the reaction solution was filtered, the coupling step was repeated once more to filter out the reaction solution, and the resin was washed 3 times with anhydrous DCM under N_2.

(2) **Oxidation:** CSO solution (0.5 M in DCM) (1.20 mL, 1.2000 mmol) was added to the reaction syringe and shaken at argon at room temperature for 20 minutes to filter out the reaction solution; The coupling step was repeated 1 more time to filter out the reaction solution; Wash the resin 3 times with anhydrous DCM under N_2.

(3) **Dmt-Deprotection:** TCA solution (3% in DCM) was added to the reaction syringe, shaken for 25 seconds, and the reaction solution was filtered; The deprotection step was repeated 3 times, and the reaction solution was filtered out and washed 6 times each with DCM, ACN and DCM, and vacuum dried.

3. Comparison of the yield of traditional method and our solid phase method

In 2014, Pedersen and co-workers[14] attempted the synthesis of similar PS-III pentamers using solution-phase chemistry, but they did not obtain pure tetramer or pentamer due to the limitation of solution-phase synthesis of these phosphoglycans. Benefiting from the high coupling efficiency of Merrifield resin, purification-free nature (between each coupling cycle) and mild photocleavable linker used in this manuscript, our solid-phase method enabled the rapid assembly and purification of PS-III oligosaccharides containing one to five phosphodisaccharide repeats (**1b**–**1f**) within few weeks. We compared the solid phase synthesis work with that of solution phase synthesis by Pedersen[8], and the results are shown in the table (the yield is calculated from the first coupling):

Table S2. Comparison of the yield of solution phase and our solid phase method

	**monomer**	**dimer**	**trimer**	**tetramer**	**pentamer**
solution phase	29%	18%	12%	4% (no NMR data)	9% (with dimer, trimer and tetramer, no NMR data)
solid phase (this work)	**60%**	**35%**	**46%**	**31%**	**20%**

4. CRM197 conjugation protocols

Oligosaccharide modification

To a solution of Oligosaccharide (1.00 mg, 1 eq) and 1,6-bis(4-nitrophenyl) hexanedioate (PNP)[15] (12.00 mg,) in DMF/ DMSO (1.50 mL, 2/1) was added triethylamine (6 μL). The reaction mixture was stirred in dark at room temperature for 6 hours to 24 hours. After LC-MS analysis showed the complete conversion of the starting material to a major product, the reaction mixture was freeze-dried then purified by HW-50S (H_2O) to afford the derivatized oligosaccharides as a yellow solid. (Quantitative yields, about 1 mg product after modification).

General procedure for conjugation

To a solution of CRM197 (0.50 mg) in 100 mM PBS buffer pH 7.4 (100 μL) was added derivatized oligosaccharides (about 1.00 mg). The mixture was stirred gently for 48 hours at room temperature. The mixture was then diluted with deionized water and washed six times by centrifugation (12,000 rpm, 10 minutes, 10 °C) with deionized water using a 10 kDa molecular weight cutoff ultrafiltration filter (Amicon® Ultra-0.5, Millipore, Billerica, MA) and then kept in PBS buffer (pH 7.4, 10 mM) at 2 mg/mL for further biological evaluation. The loading of the conjugate was measured by SDS-PAGE and MALDI-TOF mass spectrometry.

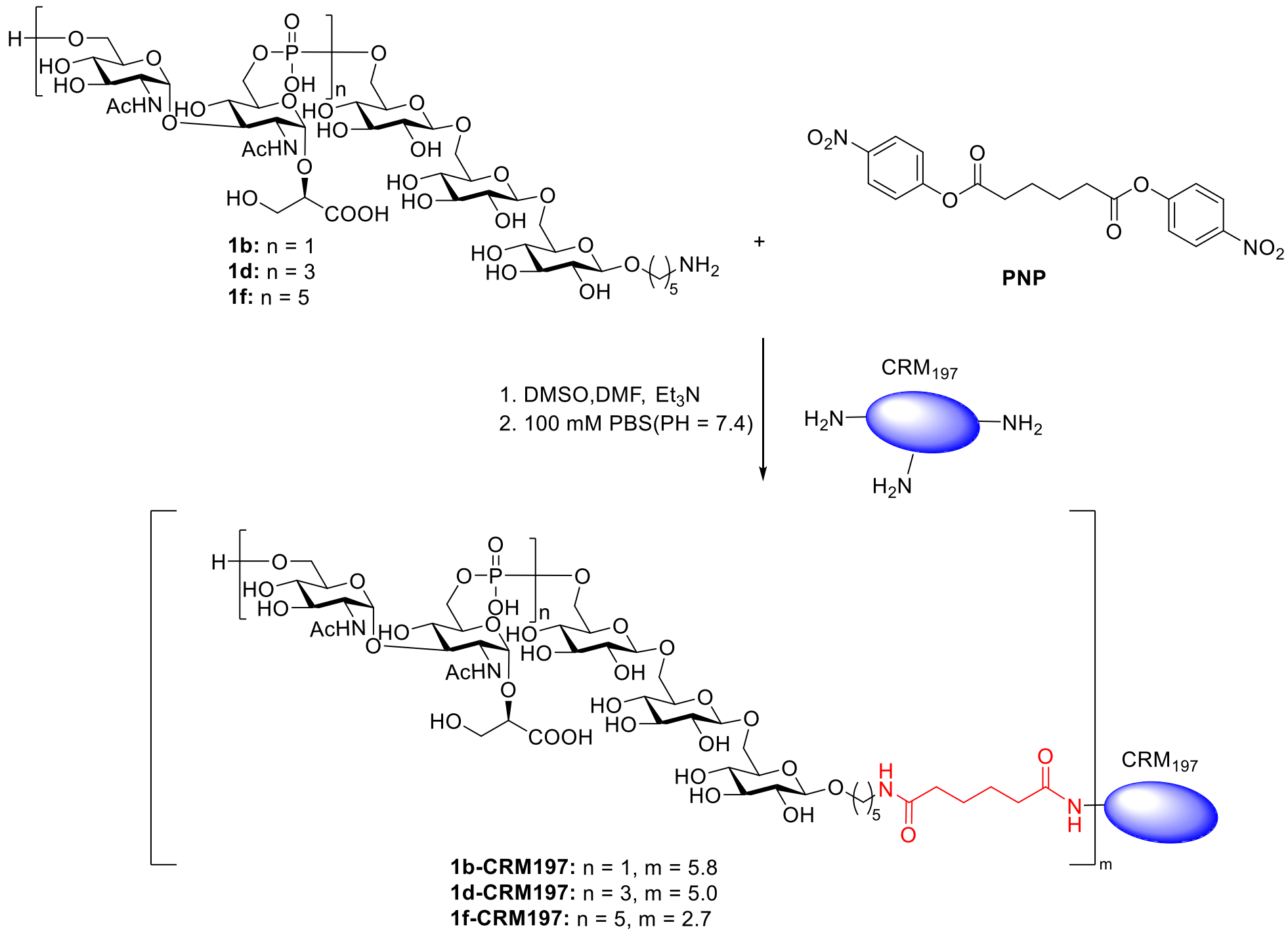

Scheme S7. Synthesis of glycoconjugates **1b/d/f-CRM197**

SDS-PAGE analysis of conjugates: The samples were diluted to a concentration of approximately 0.2 mg/mL using ultrapure water. A 12 μL aliquot of the samples were mixed with 4 μL of 5×loading buffer and loaded onto the gel. Samples were concentrated using a 5% stacking gel and separated on a 12% resolving gel. Electrophoresis was performed at 80 V during the stacking phase and 120 V during the separation phase. After electrophoresis, silver staining was performed using a commercial kit (Beyotime, P0017S) according to the manufacturer's instructions. Briefly, the gel was fixed overnight in a prefabricated fixing solution (50% ethanol and 10% acetic acid in water). The gel was then sequentially washed with 30% ethanol and ultrapure water for 10 minutes each. After sensitization with the kit-provided sensitizing solution for 2 minutes, the gel was washed twice with ultrapure water for 1 minute each. Silver staining was carried out by incubating the gel in silver solution for 10 minutes, followed by a 1minute ultrapure water rinse. Protein bands were developed using the kit-supplied developing solution until optimal band intensity was achieved. The reaction was terminated by incubating the gel in stop solution for 10 minutes. Finally, the gel was rinsed with ultrapure water and photographed for documentation.

MALDI-TOF analysis of conjugates: The glycoconjugate vaccines were diluted to approximately 0.20 mg/mL with ultrapure water. Subsequently, 2 μL aliquots were mixed with 2 μL of saturated 2,5-dihydroxybenzoic acid (DHB) matrix solution (30% acetonitrile, 0.1% trifluoroacetic acid) and spotted onto a stainless steel target plate. The molecular weights of three glycoconjugate vaccines were then tested via MALDI-TOF MS using the Bruker ultrafleXtreme. Bovine serum albumin (BSA) was employed as the calibration standard prior to analysis. The glycan loadings were calculated following the formula: [M(conjugate)-M(CRM197)]/M(oligosaccharide). For example: Loading of **1b-CRM197** = 65519-58080/1291 = 5.8

SDS-PAGE and MALDI-TOF data of conjugates

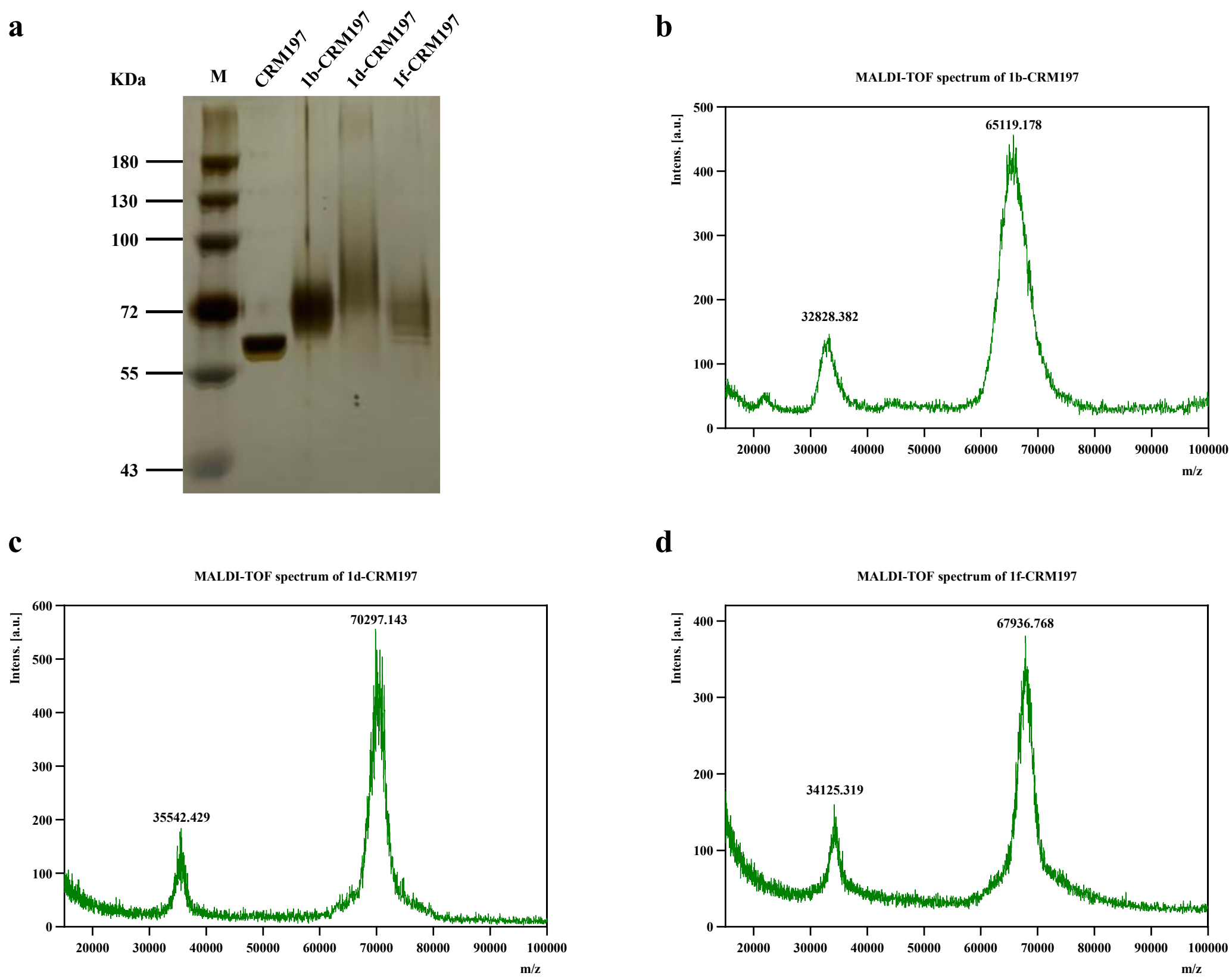

Figure S3. SDS-PAGE and MALDI-TOF analysis of three glycoconjugate vaccines. (a) SDS-PAGE gel images of three glycoconjugate vaccines. (b) MALDI-TOF spectrum of **1b-CRM197**. (c) MALDI-TOF spectrum of **1d-CRM197**. (d) MALDI-TOF spectrum of **1f-CRM197**.

Endotoxin Testing

To ensure the absence of endotoxin in the glycoconjugate vaccines, the endotoxin levels were tested using the Limulus Amebocyte Lysate (LAL) assay kit (Zhanjiang Bokang Marine Biological Co., Ltd., BKT-01). Briefly, the bacterial endotoxin standard provided with the kit was diluted to concentrations of 0.25 EU/mL, 0.125 EU/mL, 0.0625 EU/mL, and 0.0312 EU/mL to serve as the positive control; water for injection was used as the negative control. Following the instructions provided in the kit, the endotoxin content of the three glycoconjugate vaccines was tested. The test results revealed that the endotoxin levels in all three glycoconjugate vaccines were below the detection limit of 0.0312 EU/mL.

5. Immunogenicity Evaluation of Fully Synthetic *C. difficile* PS-III Conjugate Vaccines

Vaccine Formulation, Murine Immunization and Serum Collection

Fully synthetic Clostridium difficile capsular oligosaccharide conjugate vaccines (**1b-CRM197**, **1d-7-CRM197**, and **1f-CRM197**) were adjuvanted with aluminum hydroxide and administered at 3 μg glycan antigen per mouse (3 injections).[16] The control group received formalin-inactivated whole-cell *C. difficile* vaccine with Freund's adjuvant at low (0.3×10^8 cells), medium (1.5×10^8 cells), and high (7.5×10^8 cells) doses per mouse. Phosphate-buffered saline (PBS) served as a negative control (n = 5). Female C57BL/6J mice (6-8 weeks old, n ≥ 6 per group) were immunized subcutaneously with 100 μL of vaccine formulations. Prime immunization was performed on day 1, followed by booster immunizations on days 15 and 29. Blood samples were collected via the retro-orbital plexus on days 0, 14, 28, and 42. Serum were separated by centrifugation at 3,000 × g for 15 minutes and stored at -20 °C for subsequent analysis.

Ethics Statement:

Animal Studies: All animal experiments described in this study were conducted in strict accordance with the guidelines for the care and use of laboratory animals and were approved by the Animal Ethics Committee of Hainan University (Approval No. HPIACUC2025061). Throughout the study, we adhered rigorously to the principles of animal welfare, implementing measures such as euthanasia according to AVMA guidelines to minimize animal suffering and distress.

Human-Derived Cell Line: The HL60 human promyelocytic leukemia cell line, used in the OPKA assay was obtained commercially from Procell system Biotechnology Co., Ltd (Catalog No. CL-0110). As HL60 is an immortalized, commercially available cell line and not primary cells derived directly from human subjects for this specific study, its use does not require separate institutional ethics approval or informed consent under standard ethical guidelines governing established cell lines. We confirm that no primary human samples or participant data were utilized in this work.

C. difficile Challenge in Immunized Mice

Following immunization, the gut microbiota was depleted by antibiotic treatment. An antibiotic cocktail containing kanamycin (0.400 mg/mL), gentamicin (0.035 mg/mL), colistin (0.035 mg/mL),

metronidazole (0.215 mg/mL), and vancomycin (0.045 mg/mL) was administered in drinking water for 5 days. On day 5, clindamycin was intraperitoneally administered at 10 mg/kg body weight. Twenty-four hours post-depletion, mice were orally gavage with 5×10^7 *C. difficile* spores. On day 5 post-infection, mice were euthanized for fecal pellets and cecal contents collection. For bacterial load analysis, samples were homogenized in PBS, serially diluted 10-fold, and concentration of *C. difficile* were enumerated via drop-plate assay and expressed as CFU per gram.

C. difficile spores were purified through the following procedure prior to experimental use: The *C. difficile strains* were inoculated onto brain heart infusion (BHI) agar supplemented with 0.05% L-cysteine, followed by anaerobic incubation at 37 °C for 48 hours. Colonies were transferred to BHI broth with 0.05% L-cysteine and cultured anaerobically for 5 days. Bacterial pellets were collected by centrifugation (3,000 × g, 10 minutes, 4 °C), washed three times with PBS, and resuspended in PBS. The suspension was subjected to heat shock at 70 °C for 10 minutes to eliminate vegetative cells. Spore concentration was then determined by plating on taurocholate-supplemented BHI agar and calculating CFU/mL after 48 hours incubation.

Opsonophagocytic Killing Assay (OPKA)

C. difficile stocks preserved in cryoprotectant were rapidly thawed in a 37 °C-metal bath. Bacterial cells were pelleted by centrifugation (12,000 × g, 2 minutes, 4 °C), washed twice with OBB buffer, and resuspended to 1×10^6 CFU/mL. Differentiated HL-60 cells were harvested by centrifugation (350 × g, 5 minutes), washed in Ca^{2+}/Mg^{2+}-free HBSS, followed by washing in complete HBSS (with Ca^{2+}/Mg^{2+}). Cells were finally resuspended in freshly prepared OBB buffer at 5×10^6 cell/mL. In U-bottom 96-well plates, 20 μL test serum collected on day 42 post-immunization and 10 μL bacterial suspension were added per well. Negative controls received 20 μL test serum collected on day 0 (pre-immunization) plus 10 μL bacterial suspension, and blank control received 20 μL OBB buffer plus 10 μL bacterial suspension. After 30 minutes incubation at 37 °C with 700 rpm shaking, heat-inactivated complement was mixed with dHL-60 cells (1:9 v/v), and 50 μL added to wells. Plates were incubated for 45 minutes under identical conditions. Reactions were terminated on ice for 20 minutes. Ten microliters from each well were plated on taurocholate-supplemented BHI agar. Bacterial colonies were enumerated after 24 hours anaerobic incubation at 37 °C. The bactericidal activity (%) was calculated using the formula: [1 - CFU(experimental) /

CFU(blank control)] × 100%, where CFU represents colony-forming units.[17]

6. Microarray printing and screening procedure

Preparation of Glycan Arrays

Amine-terminated oligosaccharides were immobilized on commercial *N*-hydroxysuccinimide (NHS) ester-activated microarray slides (CodeLink Activated Slides; SurModics) using a piezoelectric microarray spotting device (S3; Scienion). Each slide contained 64 identical subarrays. Before the spotting, samples (100 and 500 µM) were diluted in 10 mM sodium phosphate buffer, pH 8.5. Slides were incubated in a humid chamber for 24 hours at room temperature to complete coupling reactions. The next day, slides were quenched in 5 µM ethanolamine in 50 µM Tris-HCl buffer, pH 9, for 1 hour at 50 °C. Then, slides were washed three times with deionized water, dried by centrifugation (4000 rpm, 5 minutes) and stored in a desiccator at room temperature in a black box until use.

Glycan Microarray Screening

The slides were blocked with a blocking buffer (1% BSA in PBS (w/v)) and wash 3 times with washing buffer (0.1% Tween-20 in PBS, PBST) and PBS. A FlexWell 64 grid (Grace BioLabs) was attached, and the slides were incubated with 40 µL mouse sera diluted 1:100 in sample buffer (0.01% Tween-20 and 1% BSA in PBS) in a humidification chamber for 2 hours at room temperature. After washing 3 times with PBST and PBS, slides were incubated with fluorescently labeled secondary antibodies [Multi-rAb CoraLite® Plus 647-Goat Anti-Mouse Recombinant Secondary Antibody (H+L), RGAM005; Goat Anti-Mouse IgM (Heavy chain) Secondary Antibody, Alexa Fluor™ 647, A-21238] each diluted 1:400 in blocking buffer. Slides were incubated for 1 hour at room temperature in a humidification chamber, washed three times with PBST, PBS and rinsed with deionized water, dried by centrifugation. Fluorescence signals were measured with a INNOSCAN 710 (INNPSYS) device. The photomultiplier tube (PMT) voltage was adjusted to avoid oversaturation of the signals and the wavelength was chosen based on the secondary antibody used. Data were analyzed using Origin 2022 software for statistical analysis.

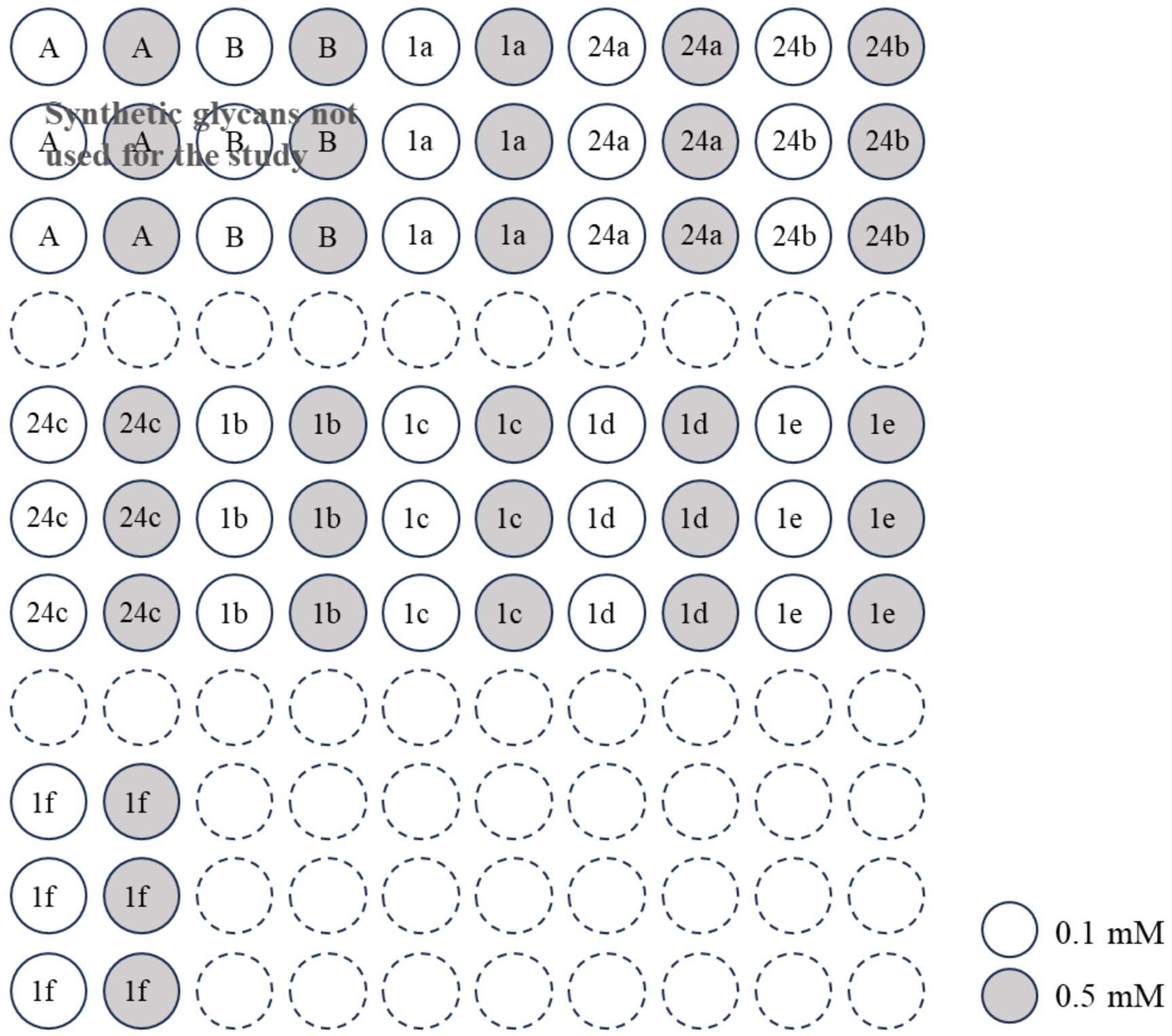

Figure S4. Microarray printing pattern of *Clostridium difficile* oligosaccharides

Synthetic glycans used for the study

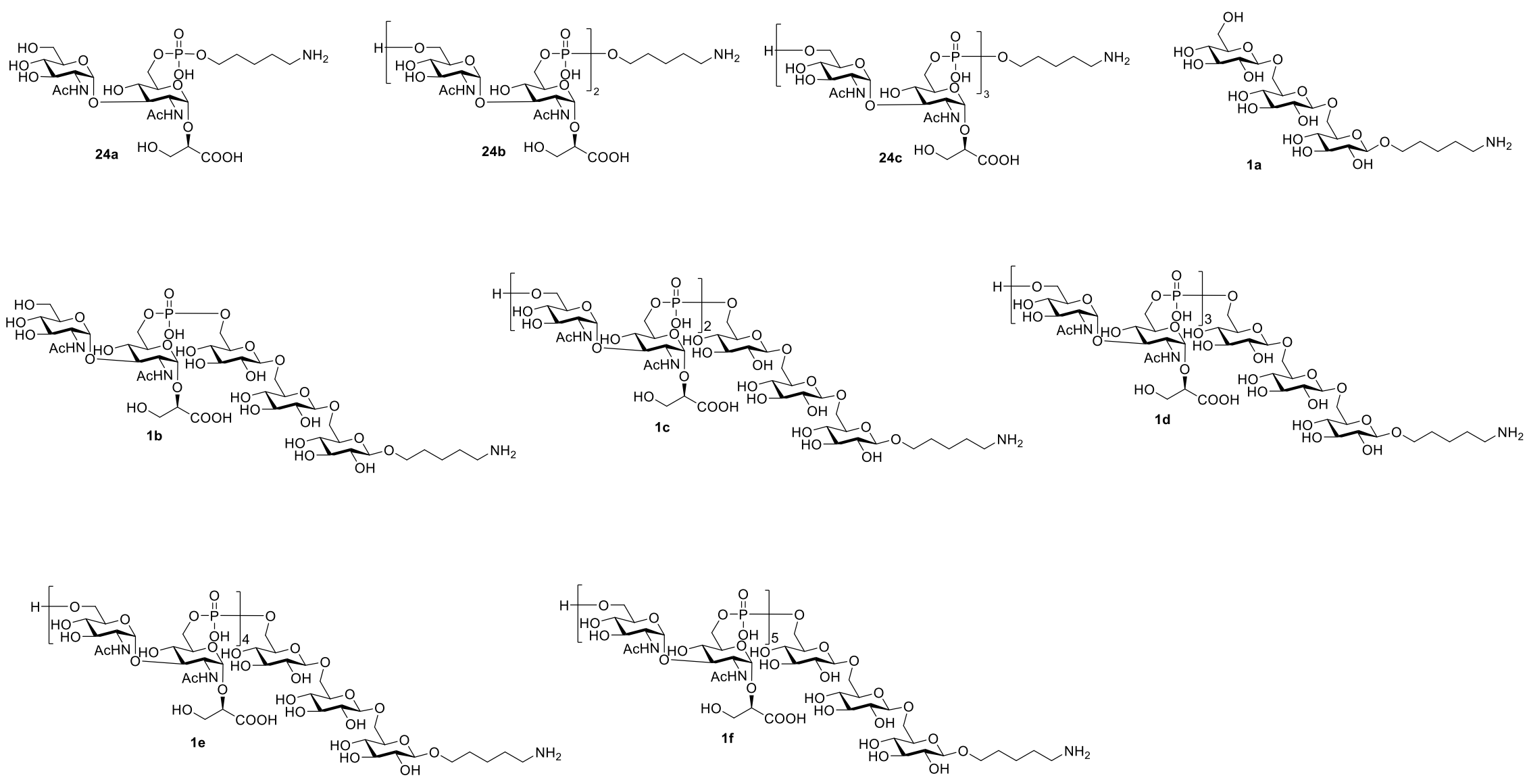

7. References

1. Chang, C. H.; Lico, L. S.; Huang, T. Y.; Lin, S. Y.; Chang, C. L.; Arco, S. D.; Hung, S. C., Synthesis of the heparin-based anticoagulant drug fondaparinux. *Angew Chem Int Ed Engl* **2014,** *53* (37), 9876-9.

2. Mong, T. K. K.; Huang, C.-Y.; Wong, C.-H., A New Reactivity-Based One-Pot Synthesis of N-Acetyllactosamine Oligomers. *J Org Chem* **2003,** *68* (6), 2135-2142.

3. Hu, Y. P.; Zhong, Y. Q.; Chen, Z. G.; Chen, C. Y.; Shi, Z.; Zulueta, M. M.; Ku, C. C.; Lee, P. Y.; Wang, C. C.; Hung, S. C., Divergent synthesis of 48 heparan sulfate-based disaccharides and probing the specific sugar-fibroblast growth factor-1 interaction. *J Am Chem Soc* **2012,** *134* (51), 20722-7.

4. Martin, C. E.; Broecker, F.; Eller, S.; Oberli, M. A.; Anish, C.; Pereira, C. L.; Seeberger, P. H., Glycan arrays containing synthetic Clostridium difficile lipoteichoic acid oligomers as tools toward a carbohydrate vaccine. *Chem Commun (Camb)* **2013,** *49* (64), 7159-61.

5. Hu, D. X.; O'Brien, M.; Ley, S. V., Continuous multiple liquid-liquid separation: diazotization of amino acids in flow. *Org Lett* **2012,** *14* (16), 4246-9.

6. Chen, D.; Srivastava, A. K.; Dubrochowska, J.; Liu, L.; Li, T.; Hoffmann, J. P.; Kolls, J. K.; Boons, G. J., A Bioactive Synthetic Outer-Core Oligosaccharide Derived from a Klebsiella pneumonia Lipopolysaccharide for Bacteria Recognition. *Chemistry* **2023,** *29* (25), e202203408.

7. Kosuri, S.; Church, G. M., Large-scale de novo DNA synthesis: technologies and applications. *Nature Methods* **2014,** *11* (5), 499-507.

8. Hogendorf, W. F.; Gisch, N.; Schwudke, D.; Heine, H.; Bols, M.; Pedersen, C. M., Total Synthesis of Five Lipoteichoic acids of Clostridium difficile. *Chemistry* **2014,** *20* (42), 13511-6.

9. Eller, S.; Collot, M.; Yin, J.; Hahm, H. S.; Seeberger, P. H., Automated solid-phase synthesis of chondroitin sulfate glycosaminoglycans. *Angew Chem Int Ed Engl* **2013,** *52* (22), 5858-61.

10. Chaube, M. A.; Trattnig, N.; Lee, D. H.; Belkhadir, Y.; Pfrengle, F., Synthesis of Fungal Cell Wall Oligosaccharides and Their Ability to Trigger Plant Immune Responses. *European J Org Chem* **2022,** *2022* (27), e202200313.

11. Liao, G.; Burgula, S.; Zhou, Z.; Guo, Z., A Convergent Synthesis of 6-O-Branched β-Glucan Oligosaccharides. *European J Org Chem* **2015,** *2015* (13), 2942-2951.

12. Chen, T. Y.; Chao, C. S.; Mong, K. K.; Chen, Y. C., Ultrasonication-assisted spray ionization mass spectrometry for on-line monitoring of organic reactions. *Chem Commun (Camb)* **2010,** *46* (44), 8347-9.

13. Delbianco, M.; Kononov, A.; Poveda, A.; Yu, Y.; Diercks, T.; Jiménez-Barbero, J.; Seeberger, P. H., Well-Defined Oligo- and Polysaccharides as Ideal Probes for Structural Studies. *J Am Chem Soc* **2018,** *140* (16), 5421-5426.

14. Pedersen, C. M.; Figueroa-Perez, I.; Lindner, B.; Ulmer, A. J.; Zähringer, U.; Schmidt, R. R., Total synthesis of lipoteichoic acid of Streptococcus pneumoniae. *Angew Chem Int Ed Engl* **2010,** *49* (14), 2585-90.

15. Wu, X.; Ling, C.-C.; Bundle, D. R., A New Homobifunctional p-Nitro Phenyl Ester Coupling Reagent for the Preparation of Neoglycoproteins. *Org Lett* **2004,** *6* (24), 4407-4410.

16. Broecker, F.; Wegner, E.; Seco, B. M. S.; Kaplonek, P.; Bräutigam, M.; Ensser, A.; Pfister, F.; Daniel, C.; Martin, C. E.; Mattner, J.; Seeberger, P. H., Synthetic Oligosaccharide-Based Vaccines Protect Mice from Clostridioides difficile Infections. *ACS Chem Biol* **2019,** *14* (12), 2720-2728.

17. Paschall, A. V.; Middleton, D. R.; Avci, F. Y., Opsonophagocytic Killing Assay to Assess Immunological Responses Against Bacterial Pathogens. *JoVE* **2019,** (146), e59400.

NMR and HR-MS Spectrum

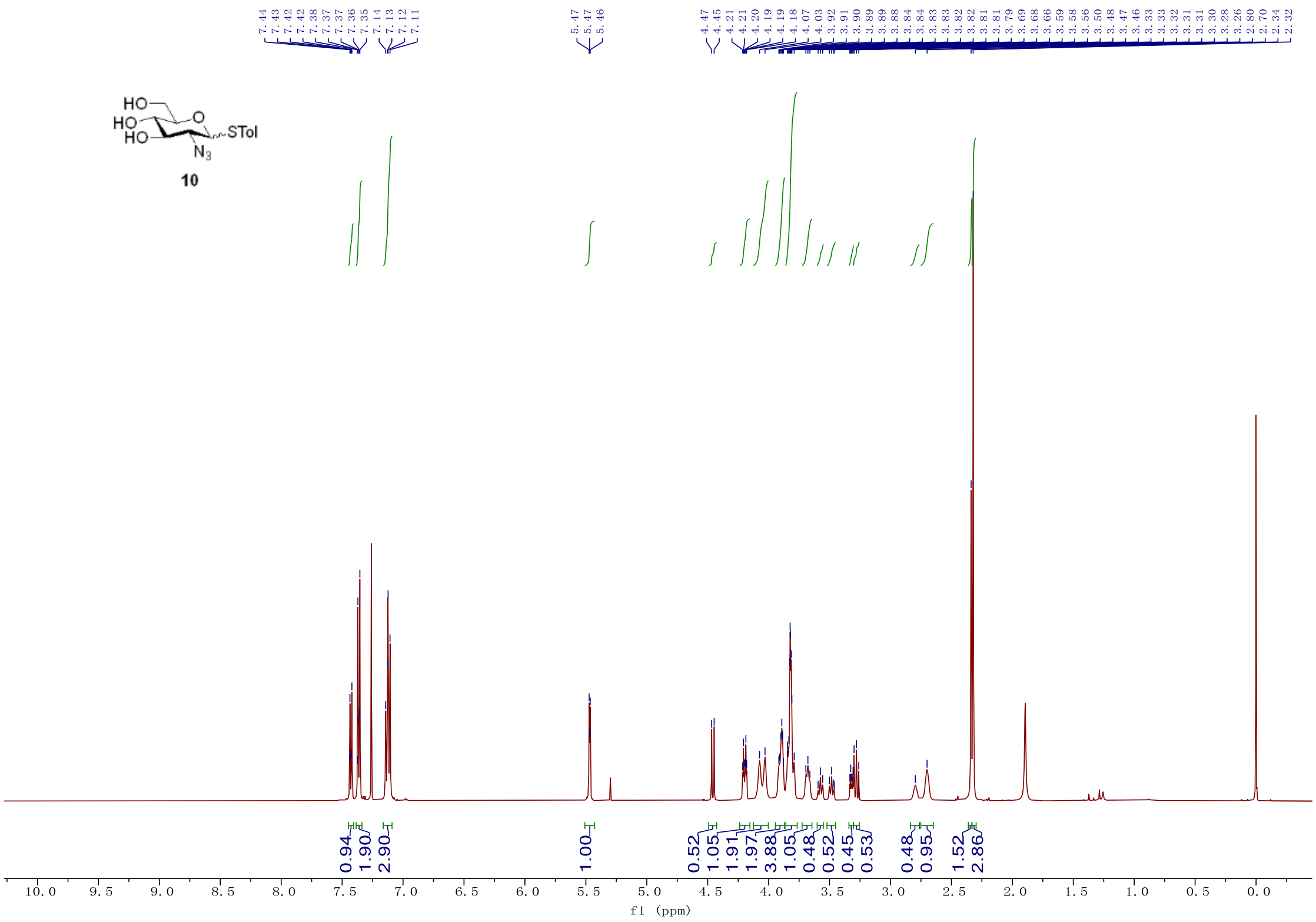

Figure S5. Compound 10 ^{1}H NMR ($CDCl_3$, 500 MHz)

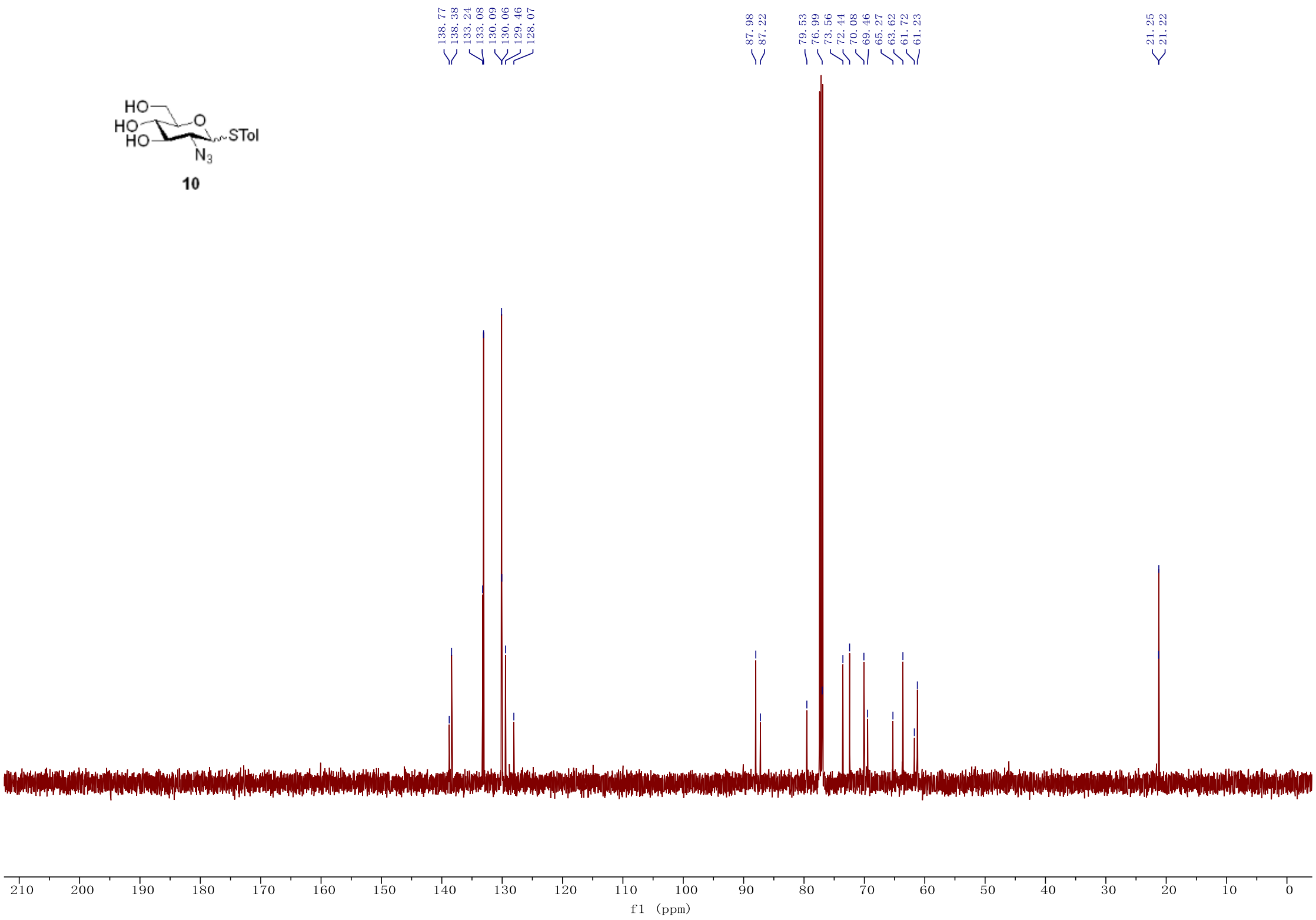

Figure S6. Compound 10 ^{13}C NMR ($CDCl_3$, 126 MHz)

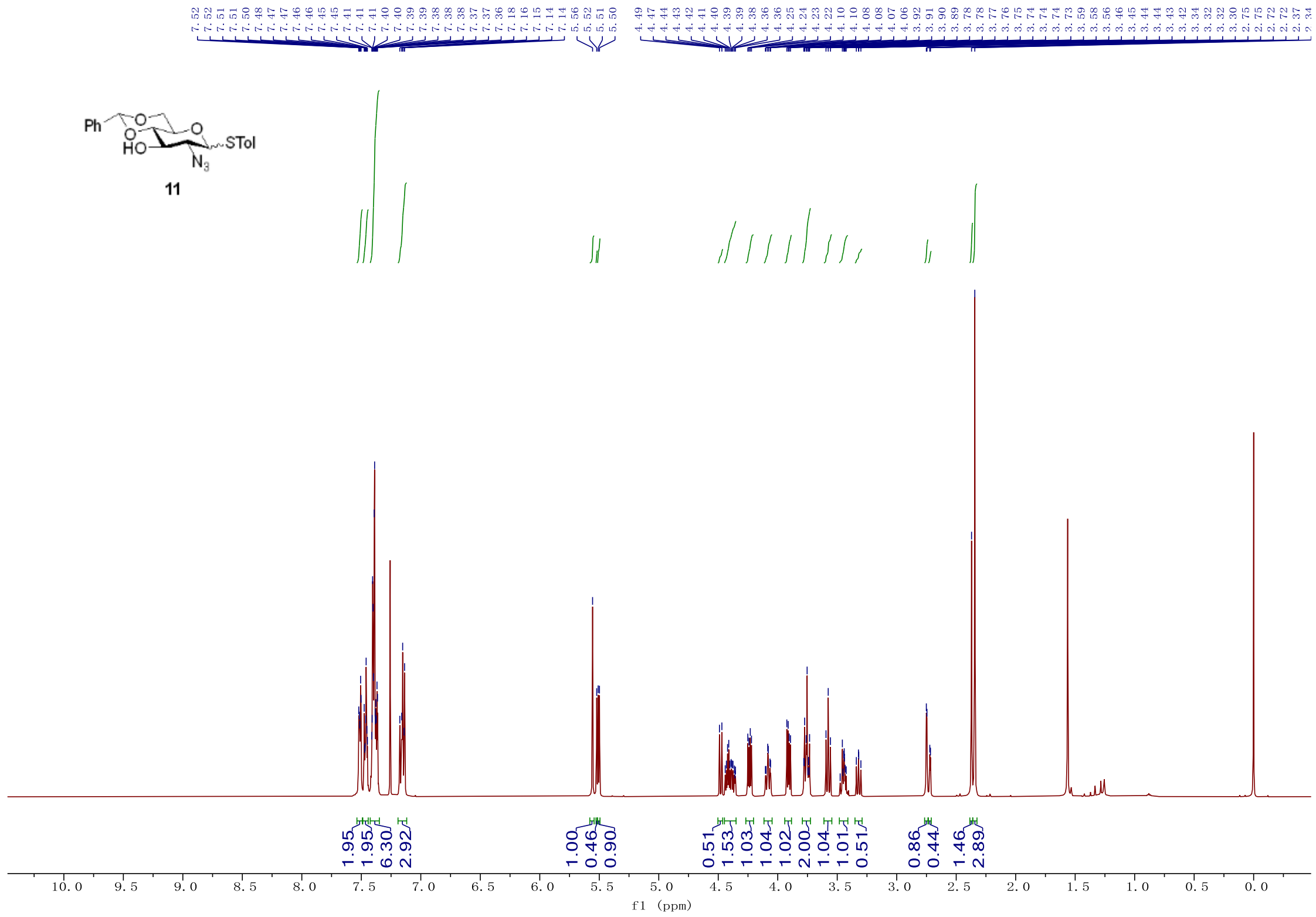

Figure S7. Compound **11** ^{1}H NMR ($CDCl_3$, 500 MHz)

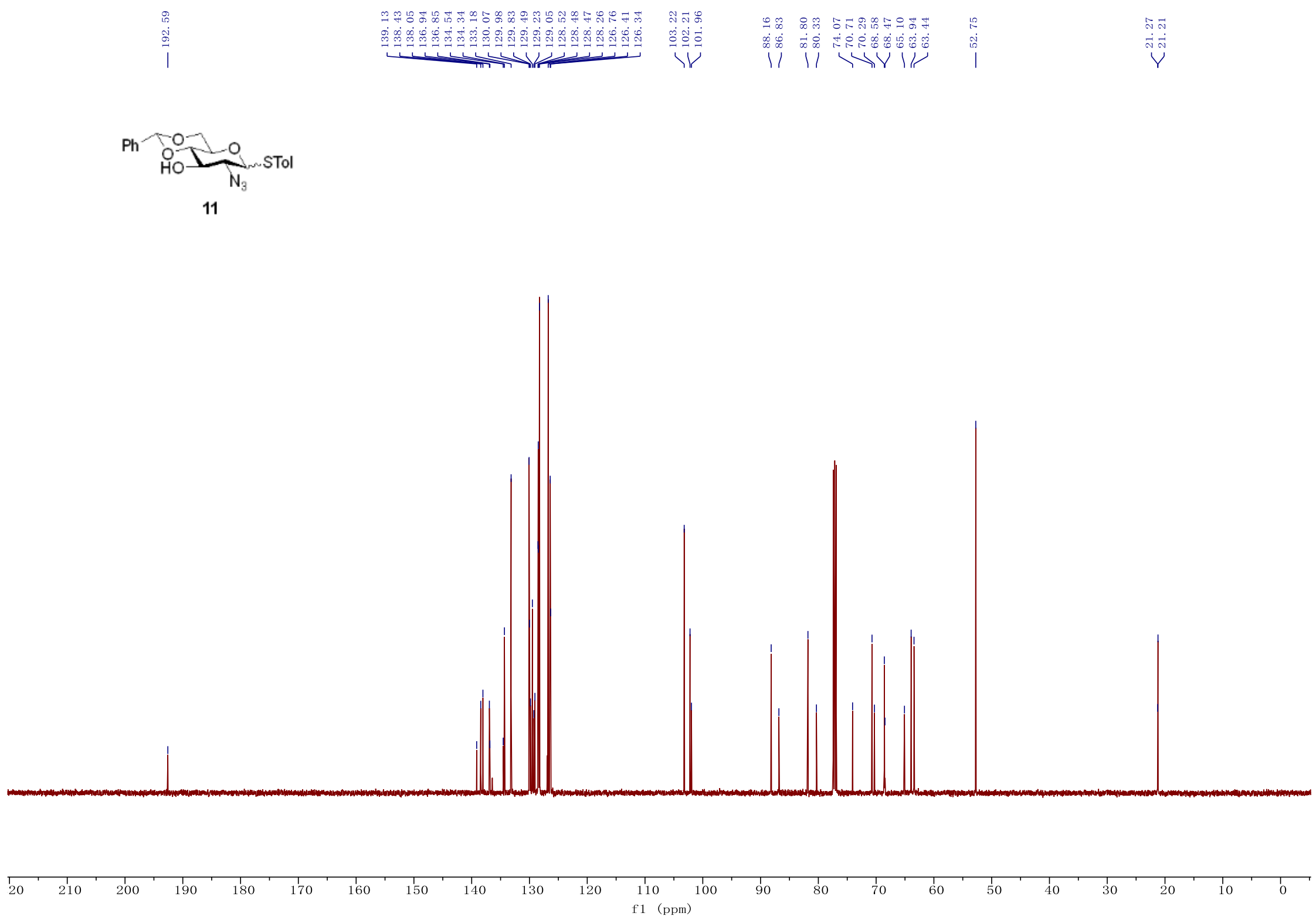

Figure S8. Compound **11** ^{13}C NMR ($CDCl_3$, 126 MHz)

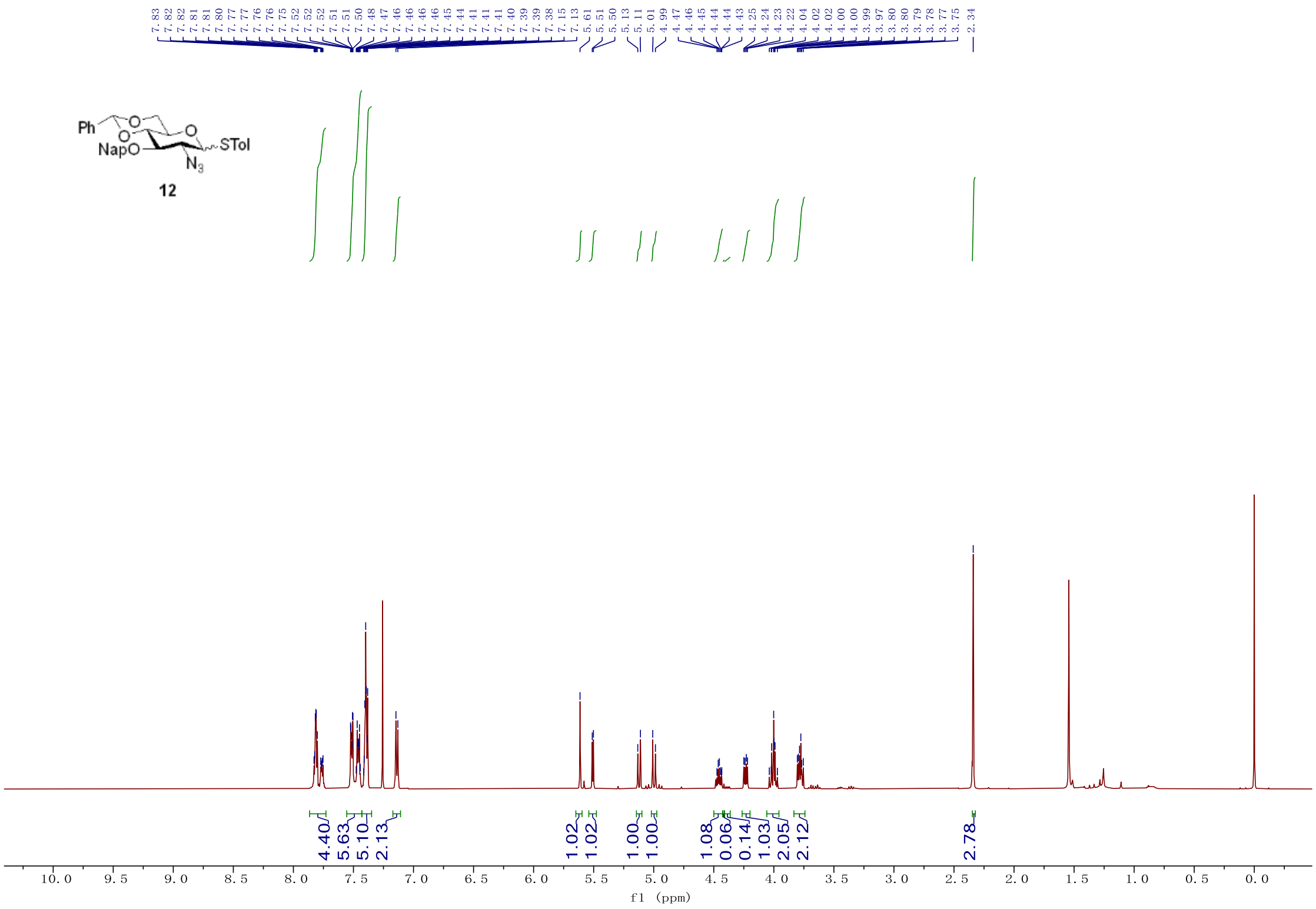

Figure S9. Compound **12** ^{1}H NMR ($CDCl_3$, 500 MHz)

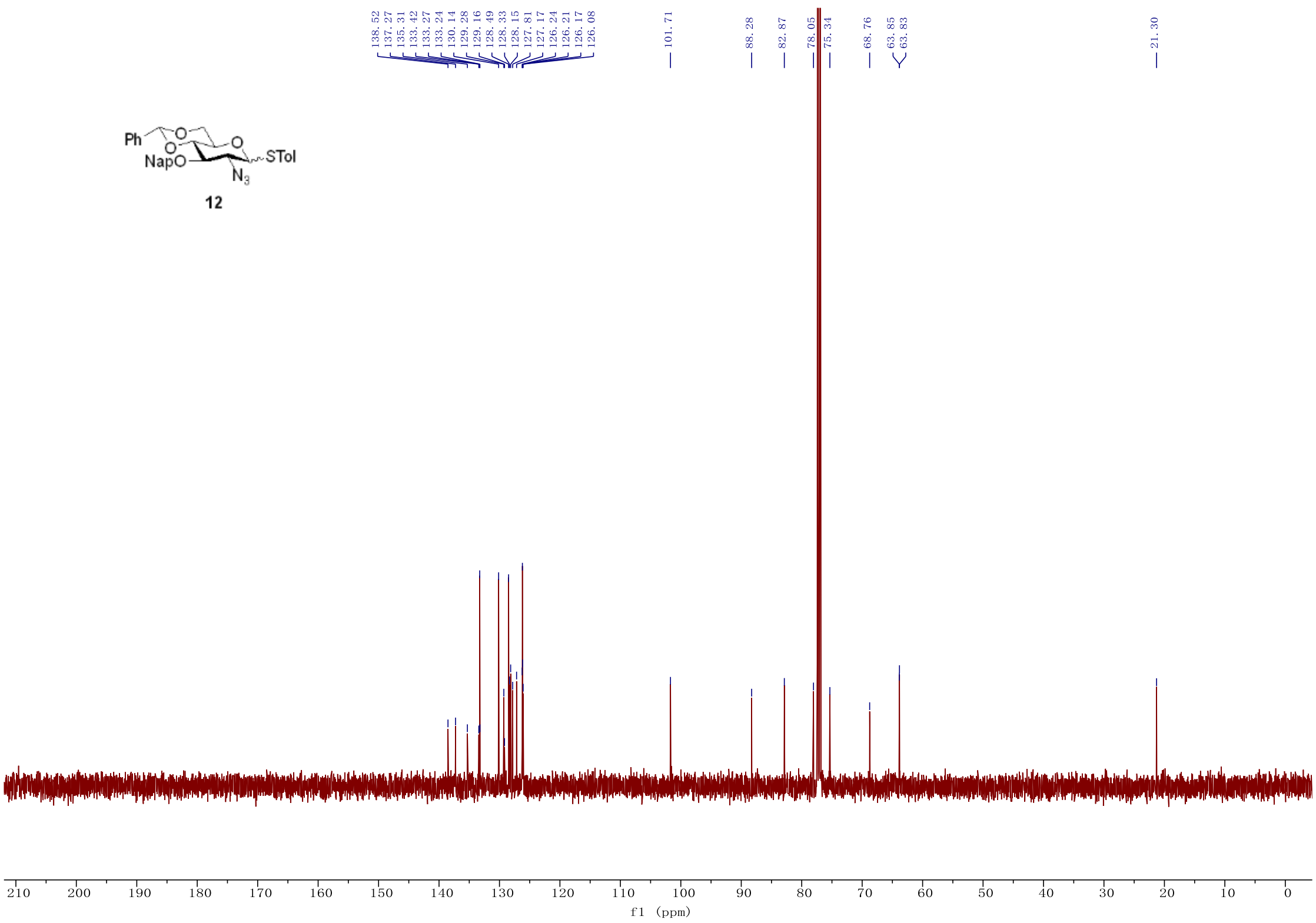

Figure S10. Compound **12** ^{13}C NMR ($CDCl_3$, 126 MHz)

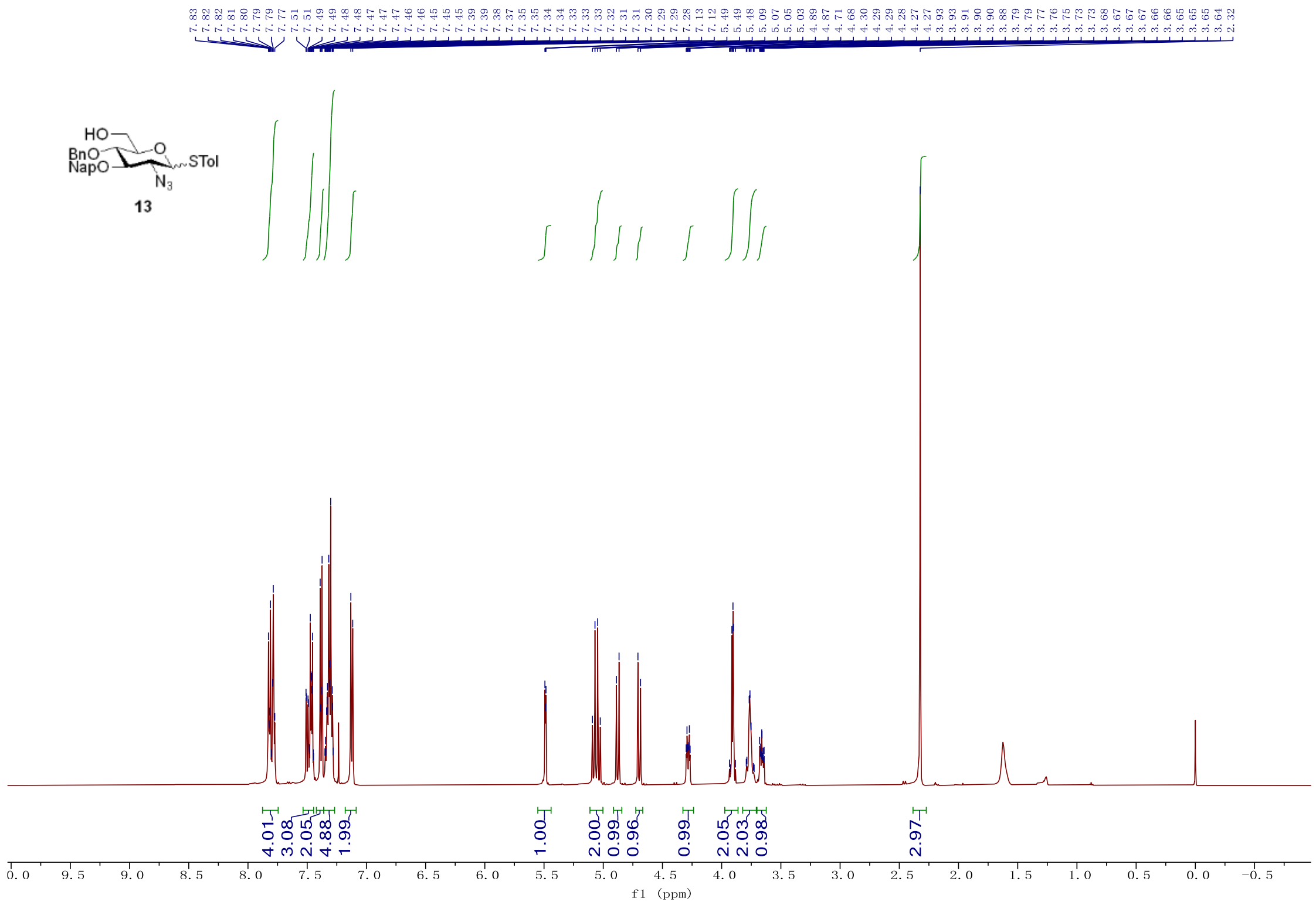

Figure S11. Compound **13** ^{1}H NMR ($CDCl_3$, 500 MHz)

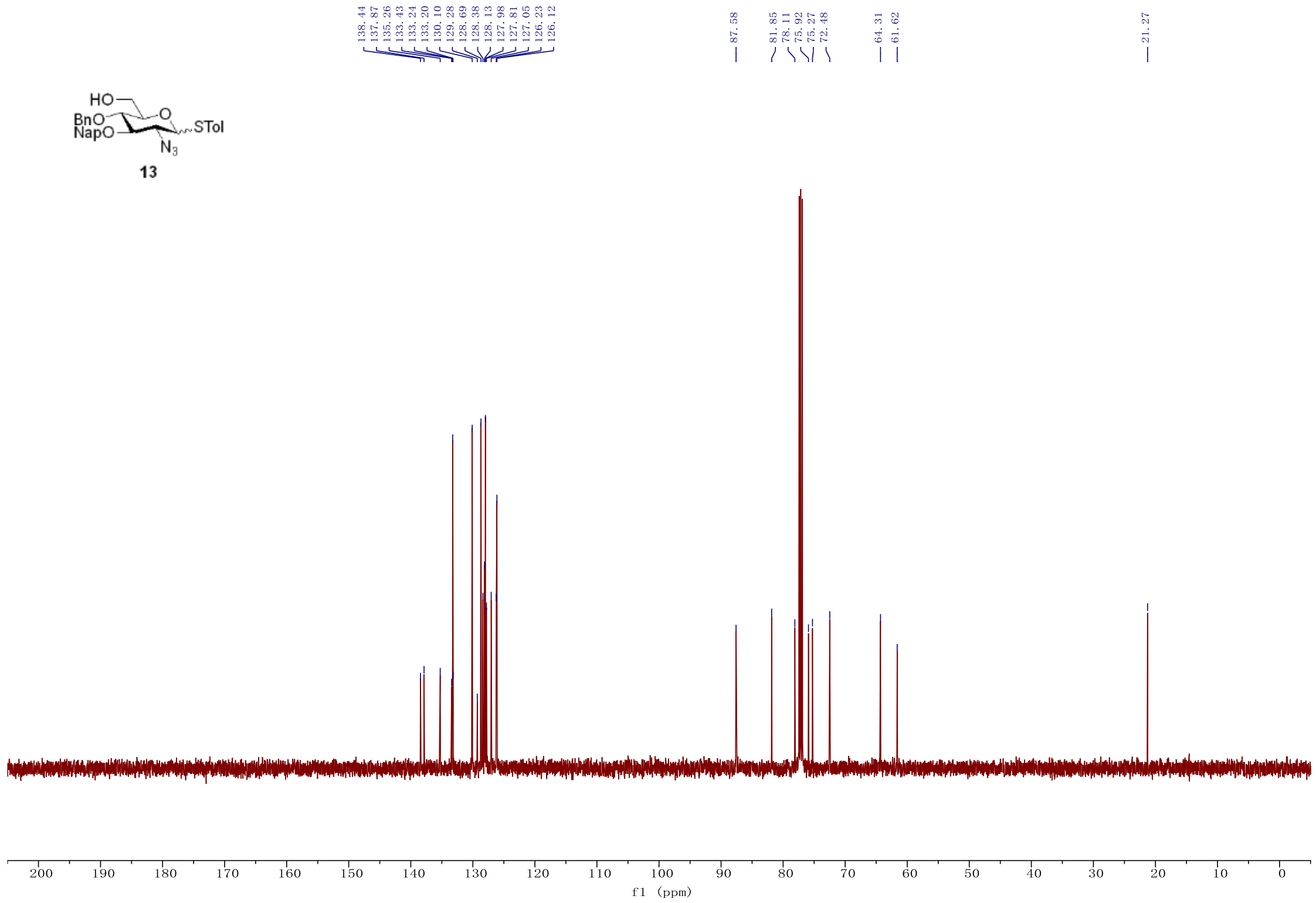

Figure S12. Compound **13** ^{13}C NMR ($CDCl_3$, 126 MHz)

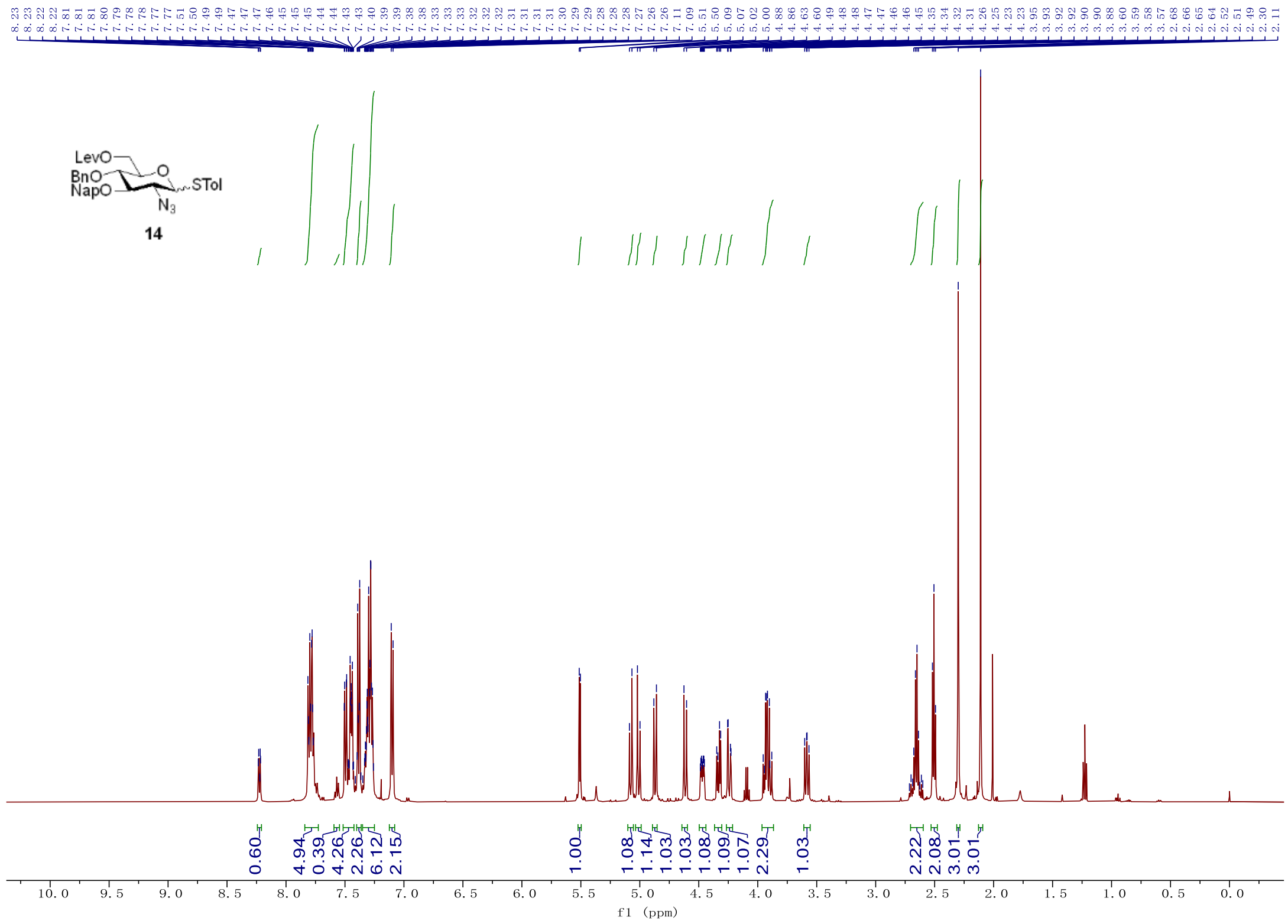

Figure S13. Compound **14** ^{1}H NMR ($CDCl_3$, 500 MHz)

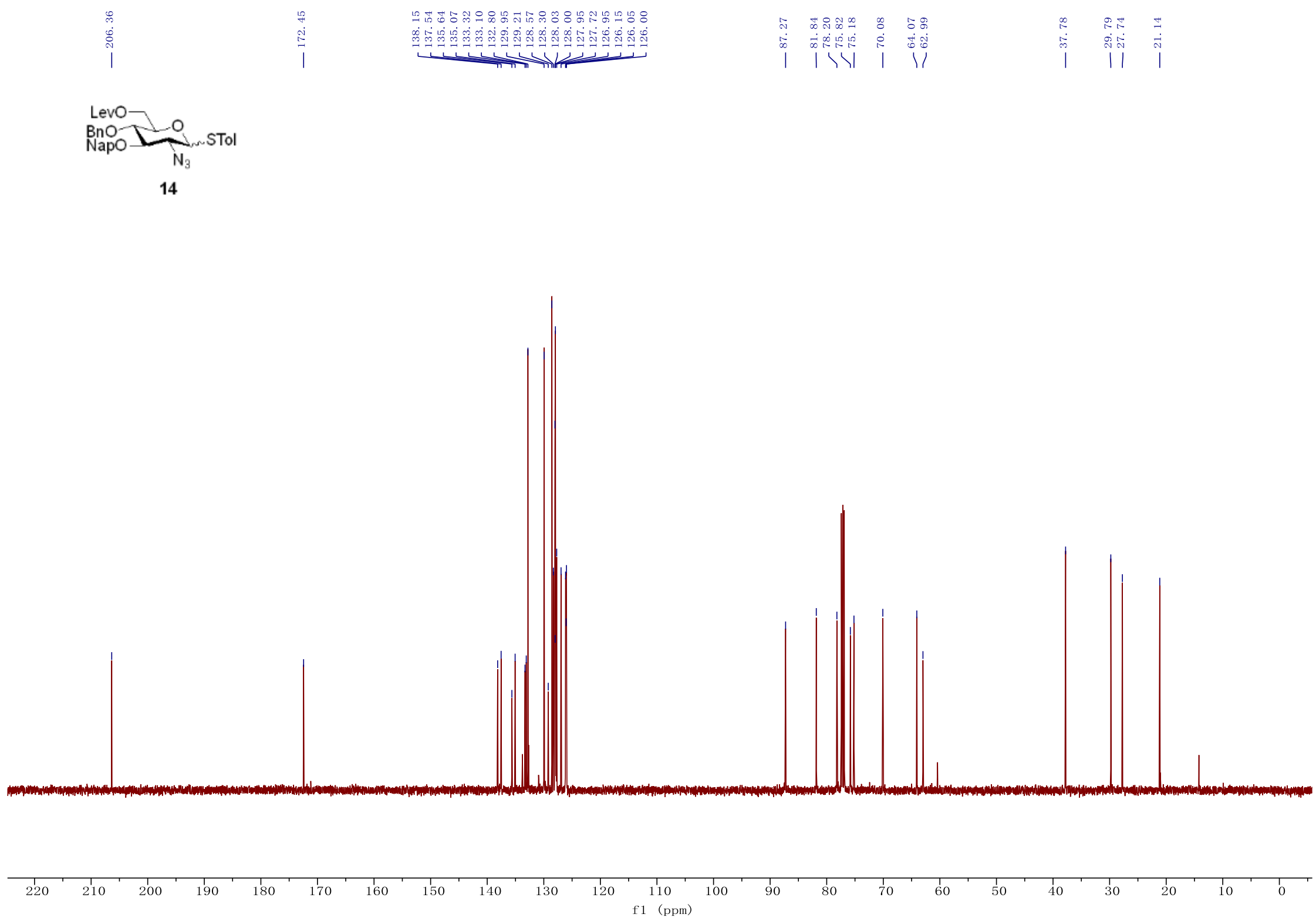

Figure S14. Compound **14** ^{13}C NMR ($CDCl_3$, 126 MHz)

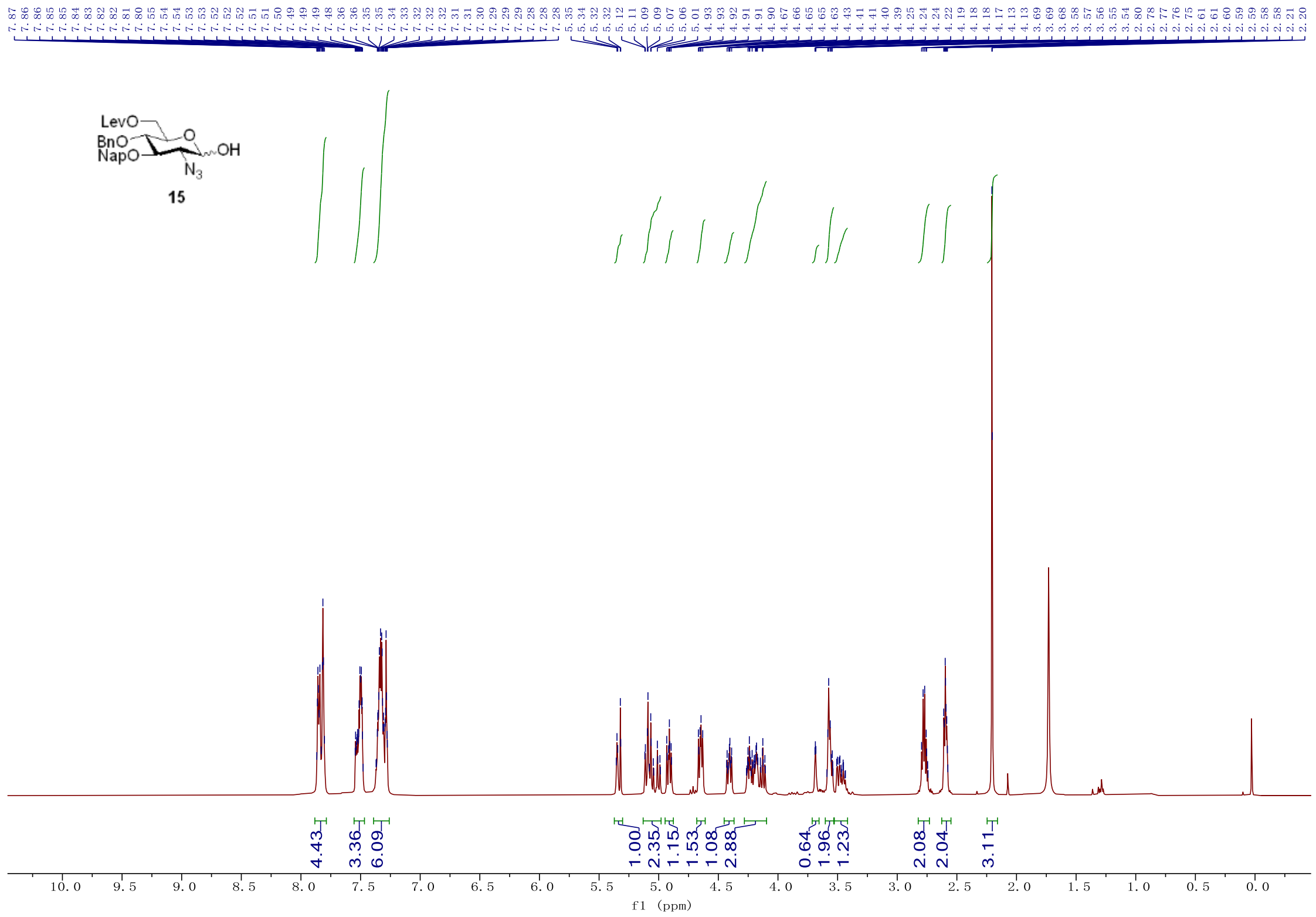

Figure S15. Compound **15** ^{1}H NMR ($CDCl_3$, 500 MHz)

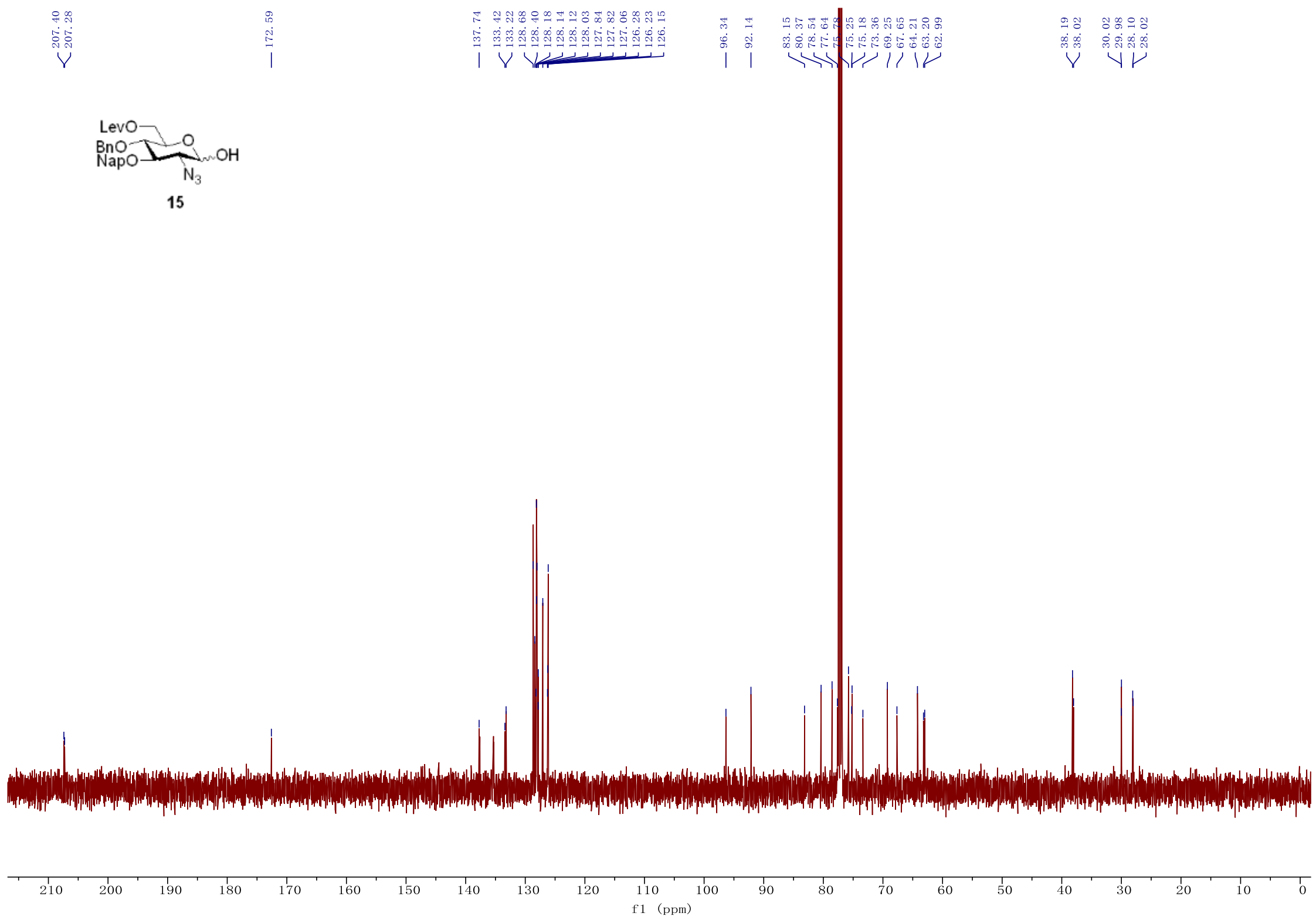

Figure S16. Compound **15** ^{13}C NMR ($CDCl_3$, 126 MHz)

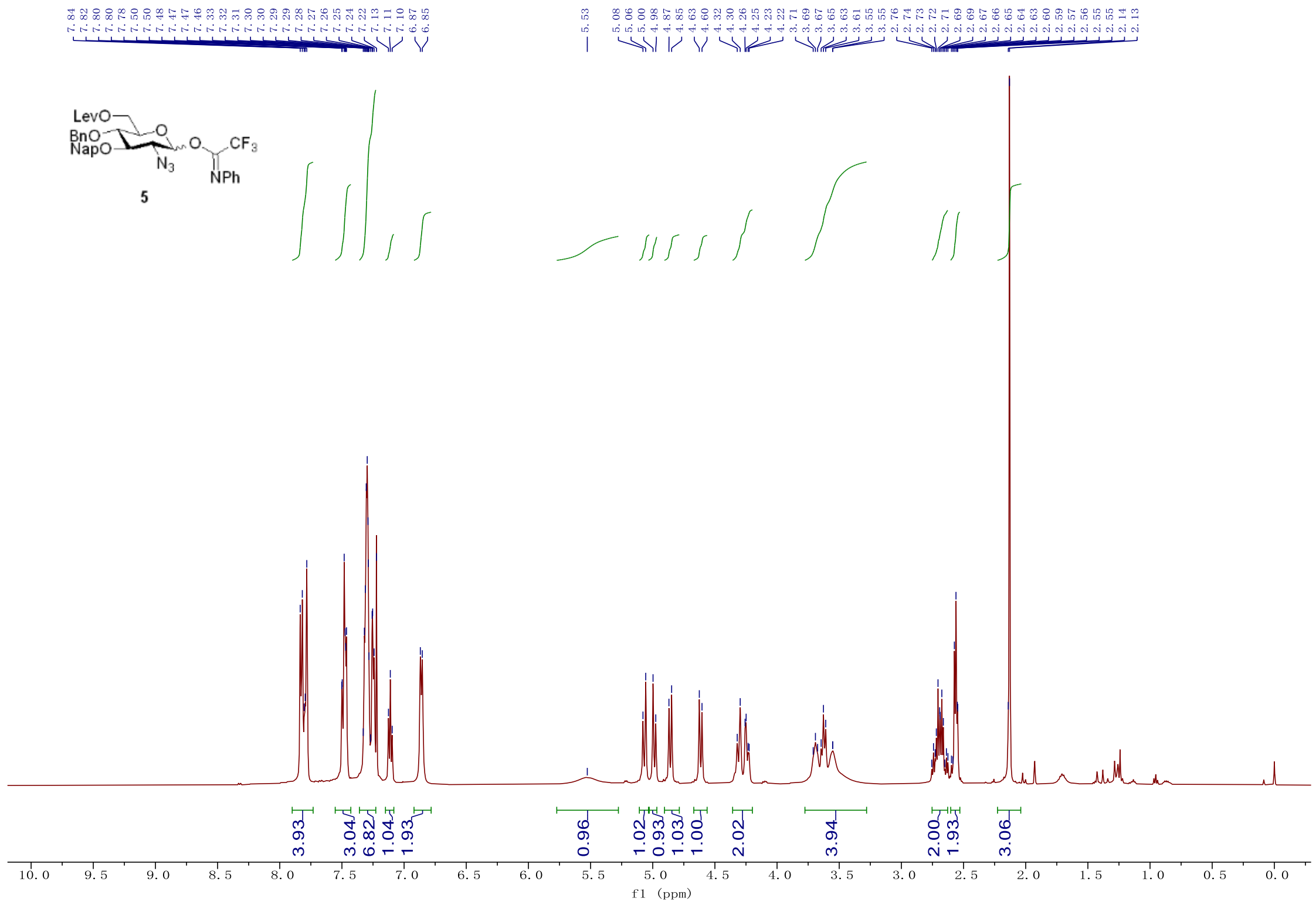

Figure S17. Compound **5** ^{1}H NMR ($CDCl_3$, 500 MHz)

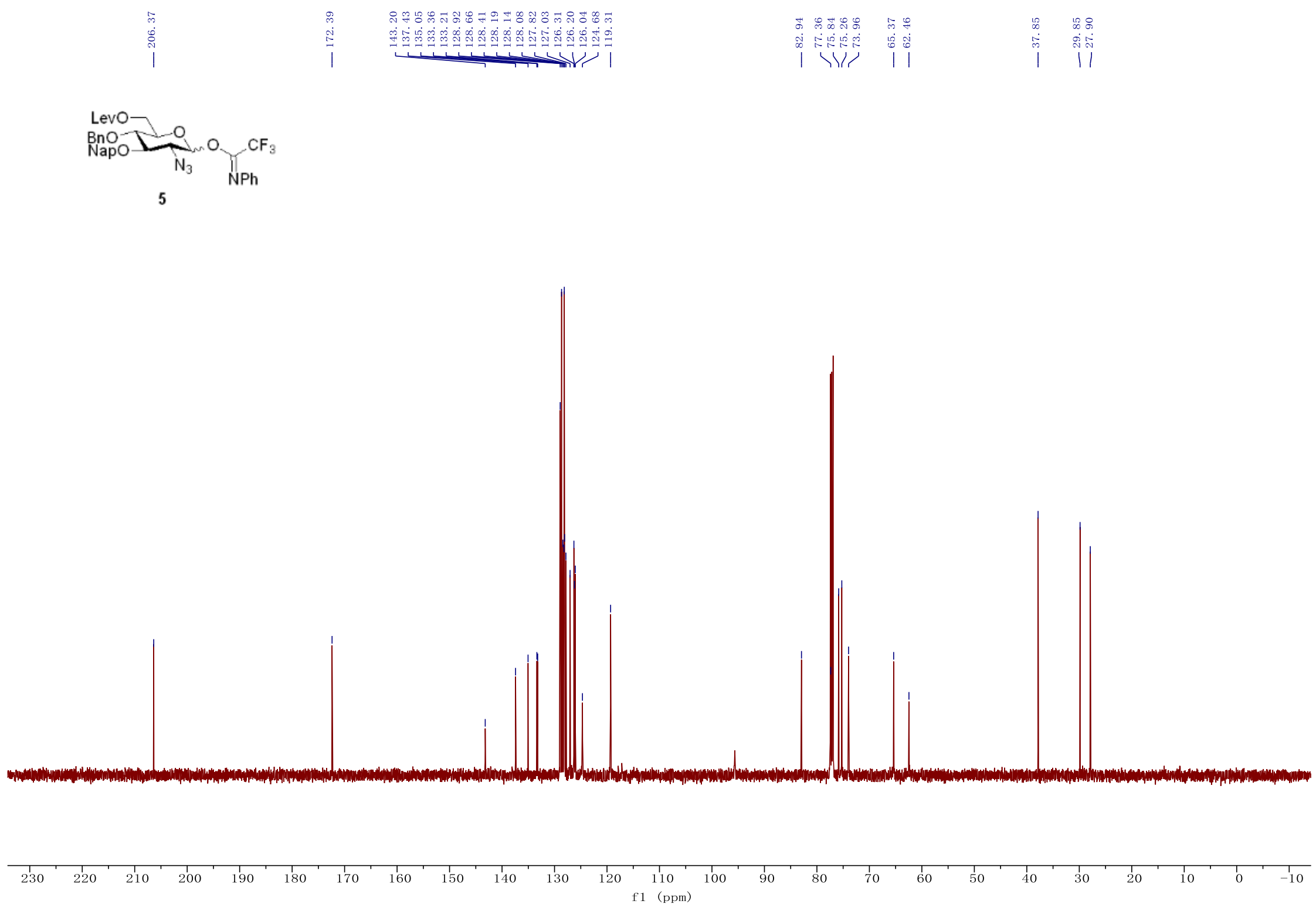

Figure S18. Compound **5** ^{13}C NMR ($CDCl_3$, 126 MHz)

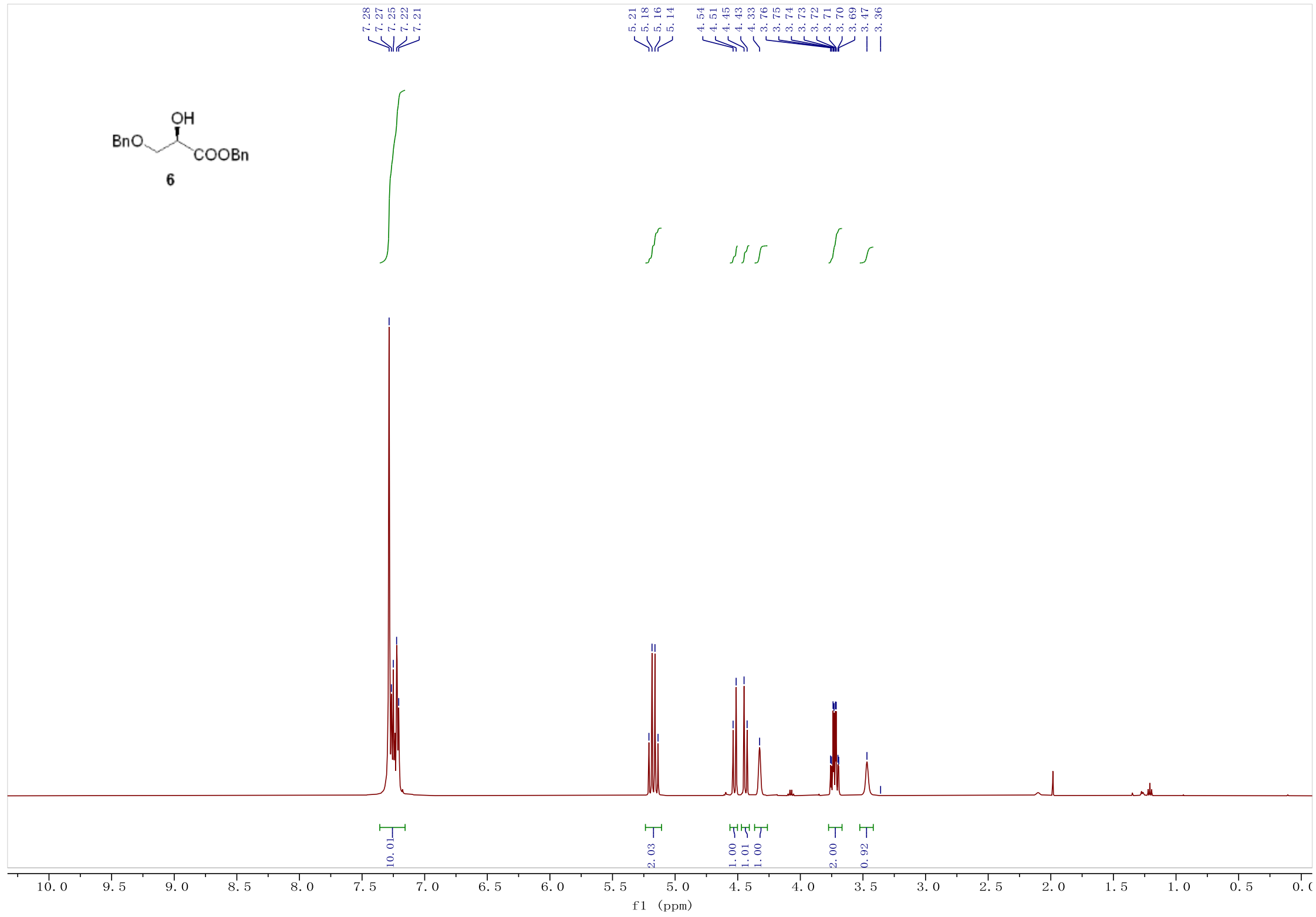

Figure S19. Compound **6** ^{1}H NMR ($CDCl_3$, 500 MHz)

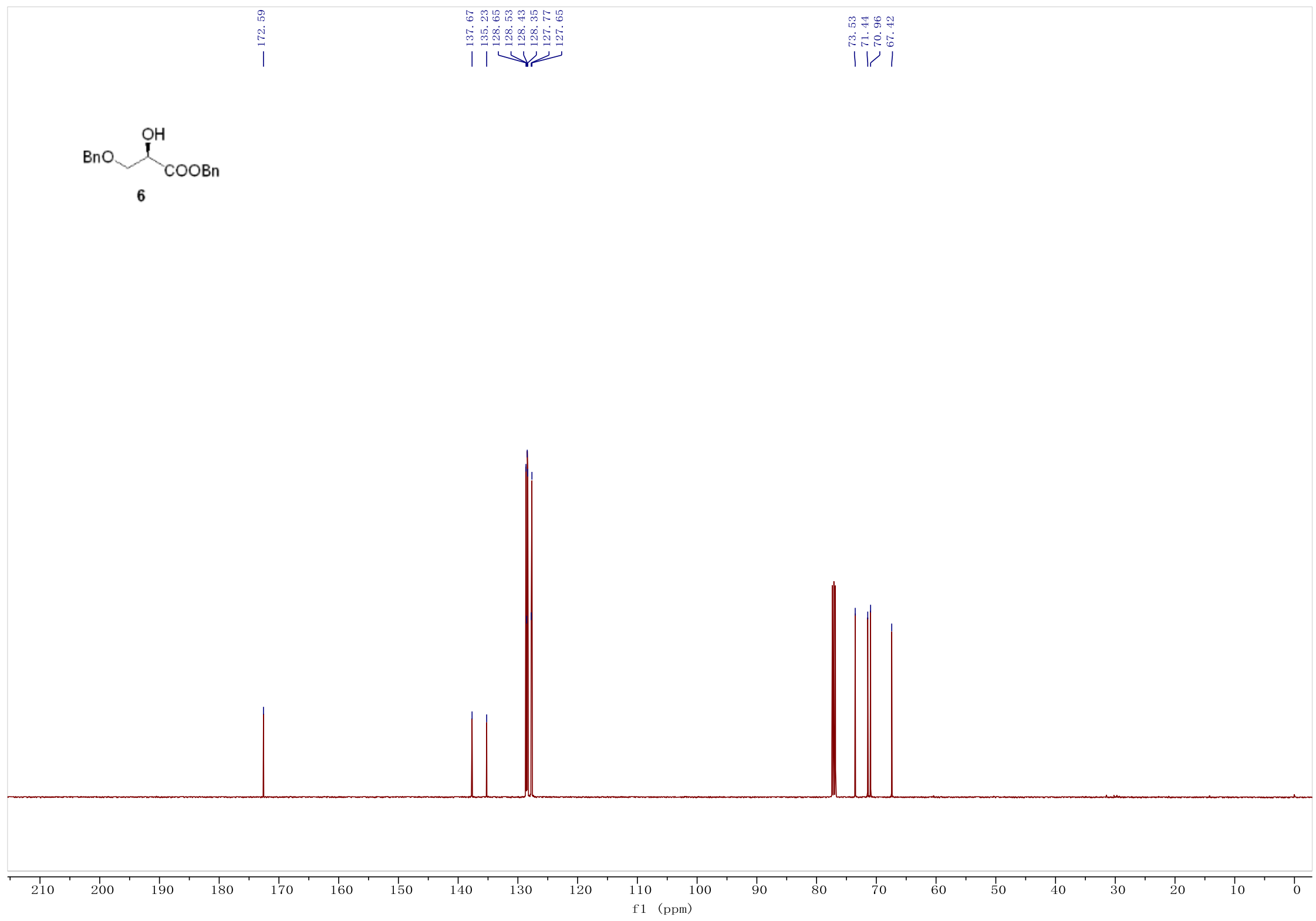

Figure S20. Compound **6** ^{13}C NMR ($CDCl_3$, 126 MHz)

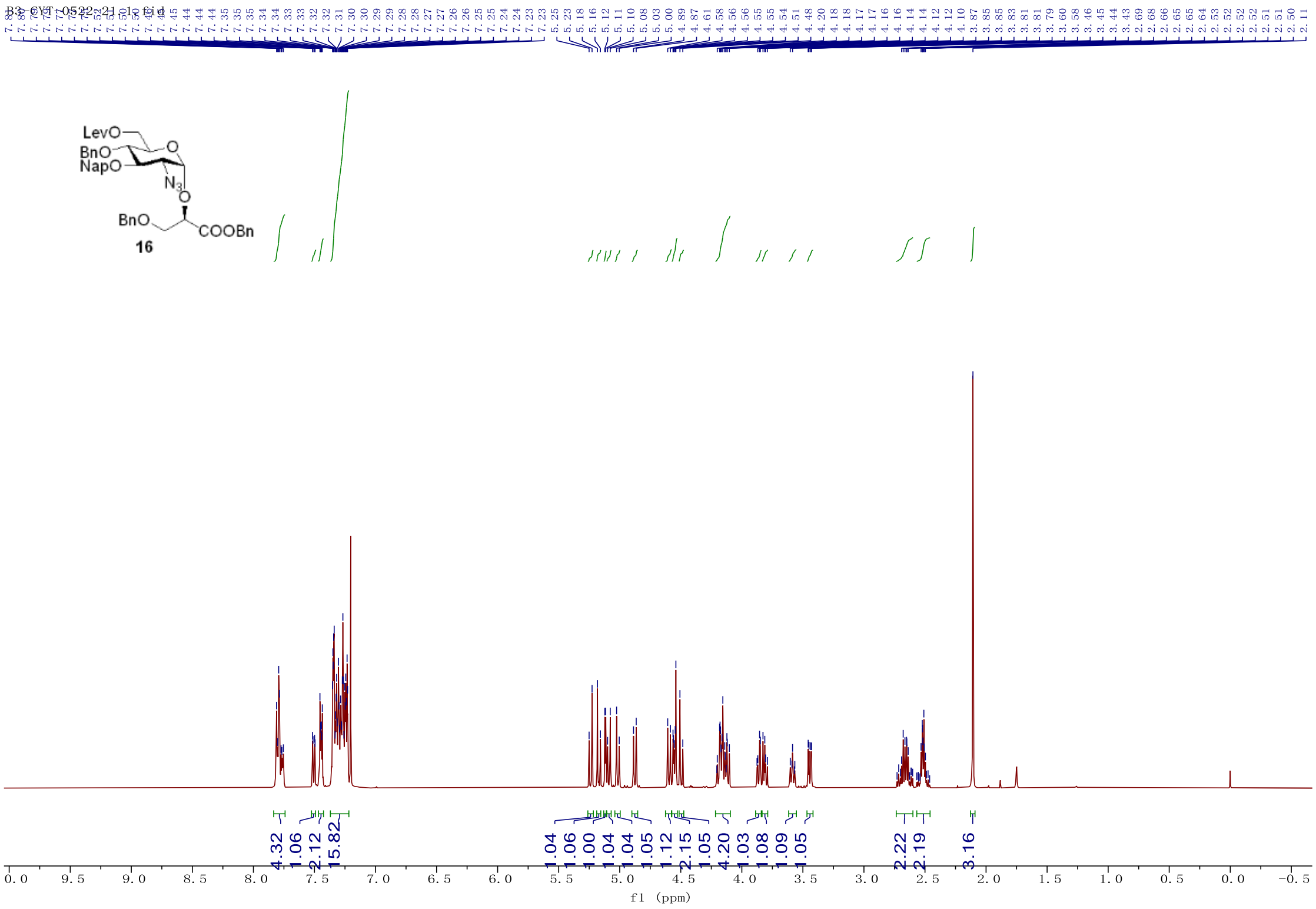

Figure S21. Compound **16** ^{1}H NMR ($CDCl_3$, 500 MHz)

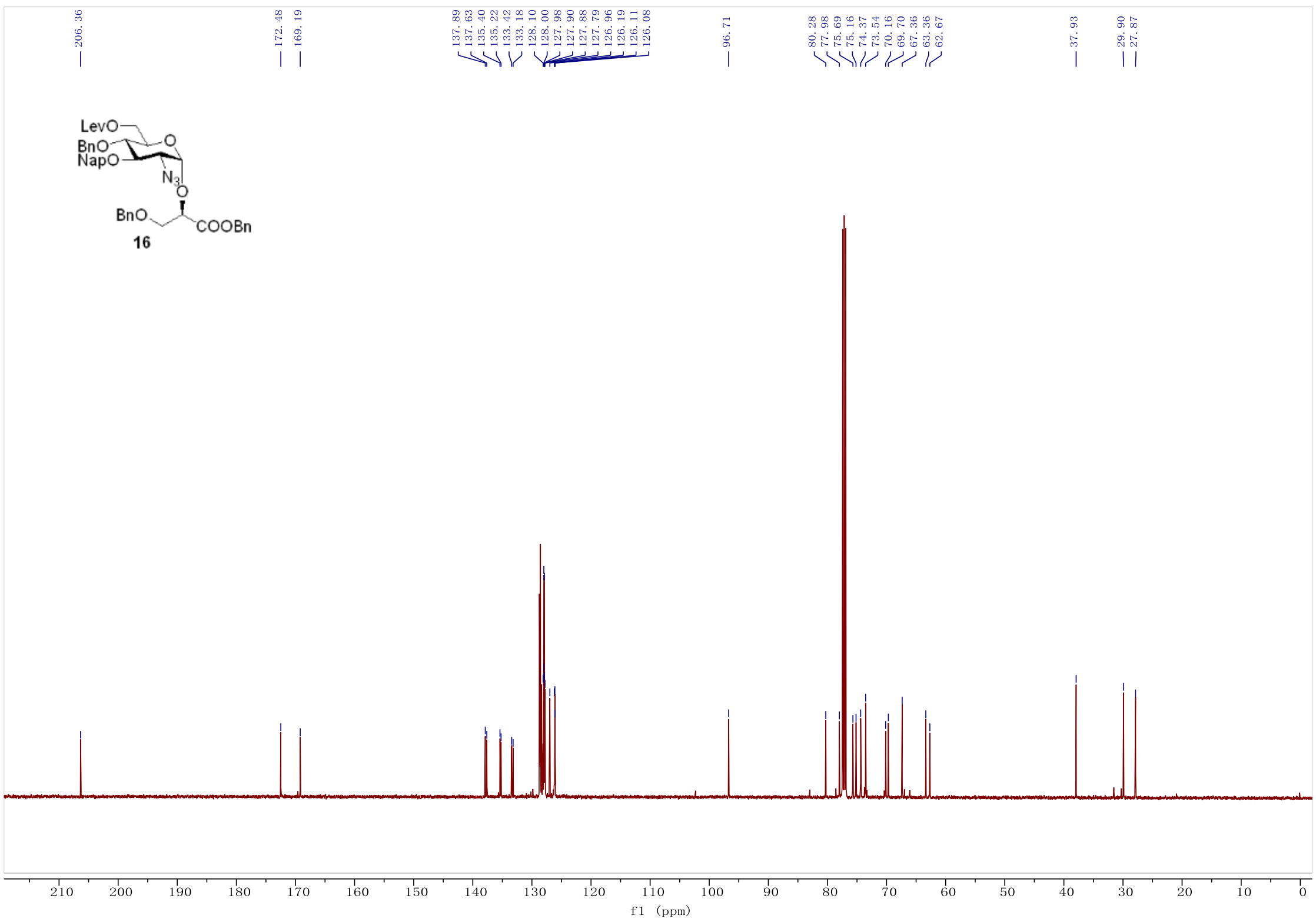

Figure S22. Compound **16** ^{13}C NMR ($CDCl_3$, 126 MHz)

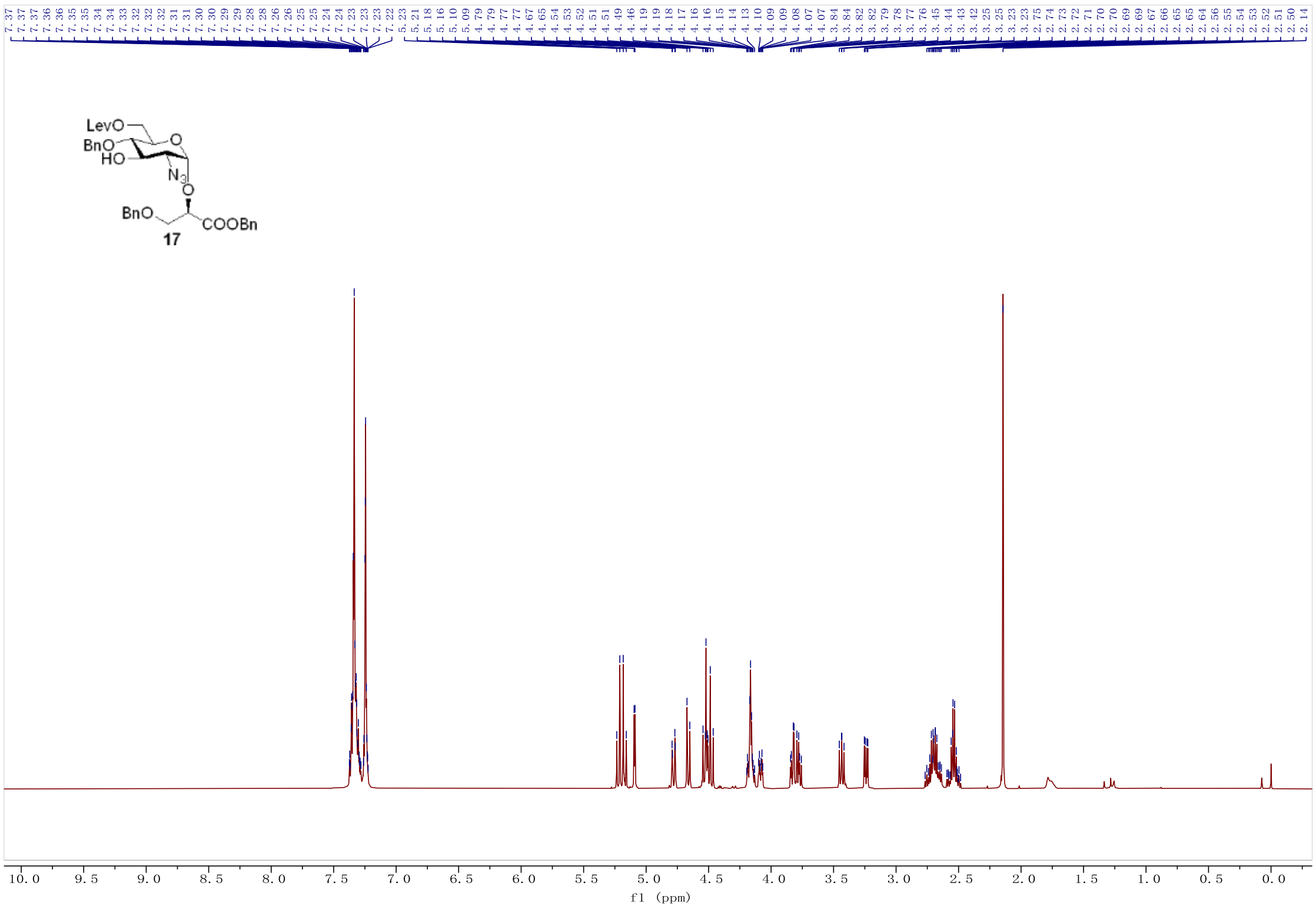

Figure S23. Compound **17** ^{1}H NMR ($CDCl_3$, 500 MHz)

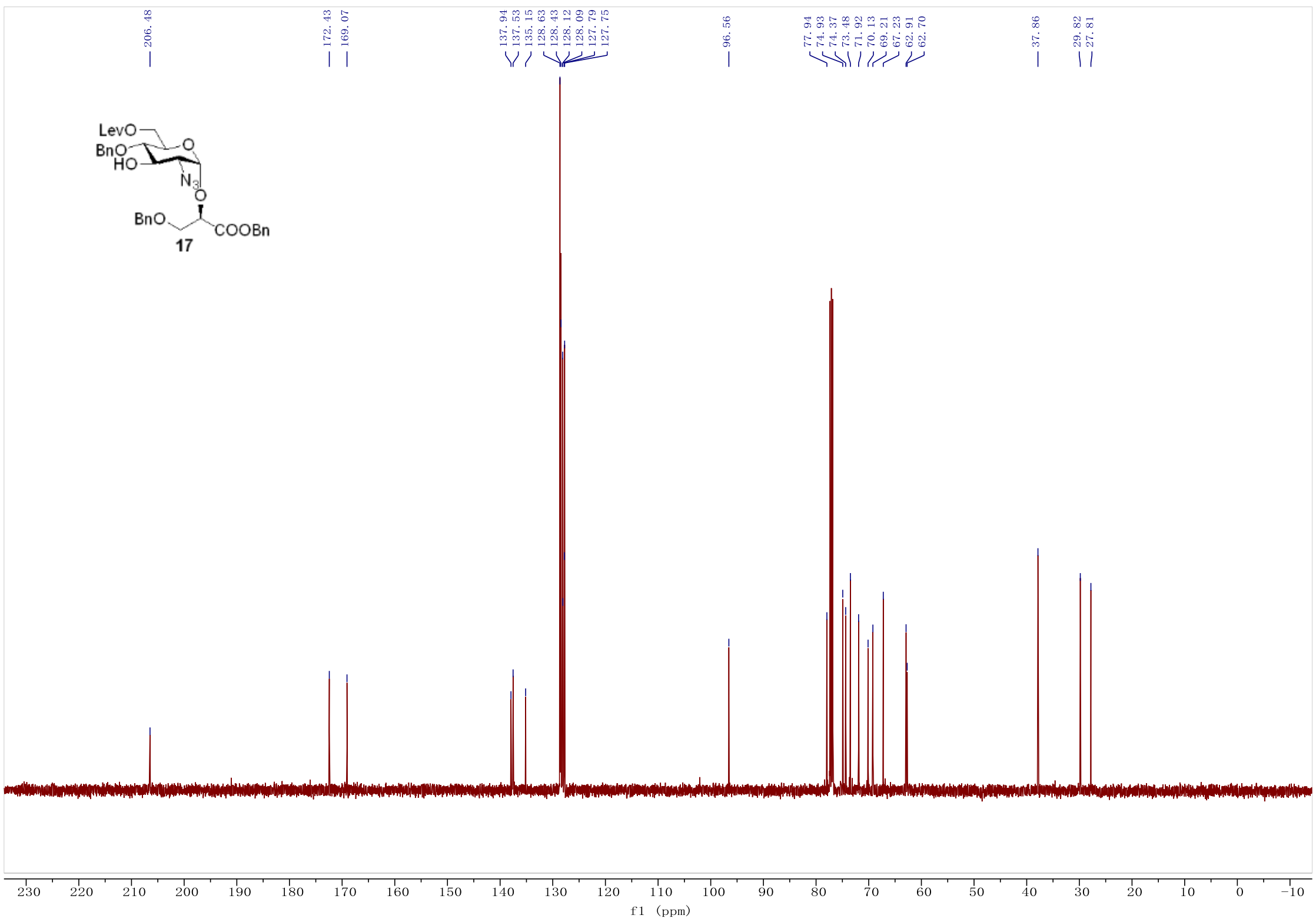

Figure S24. Compound **17** ^{13}C NMR ($CDCl_3$, 126 MHz)

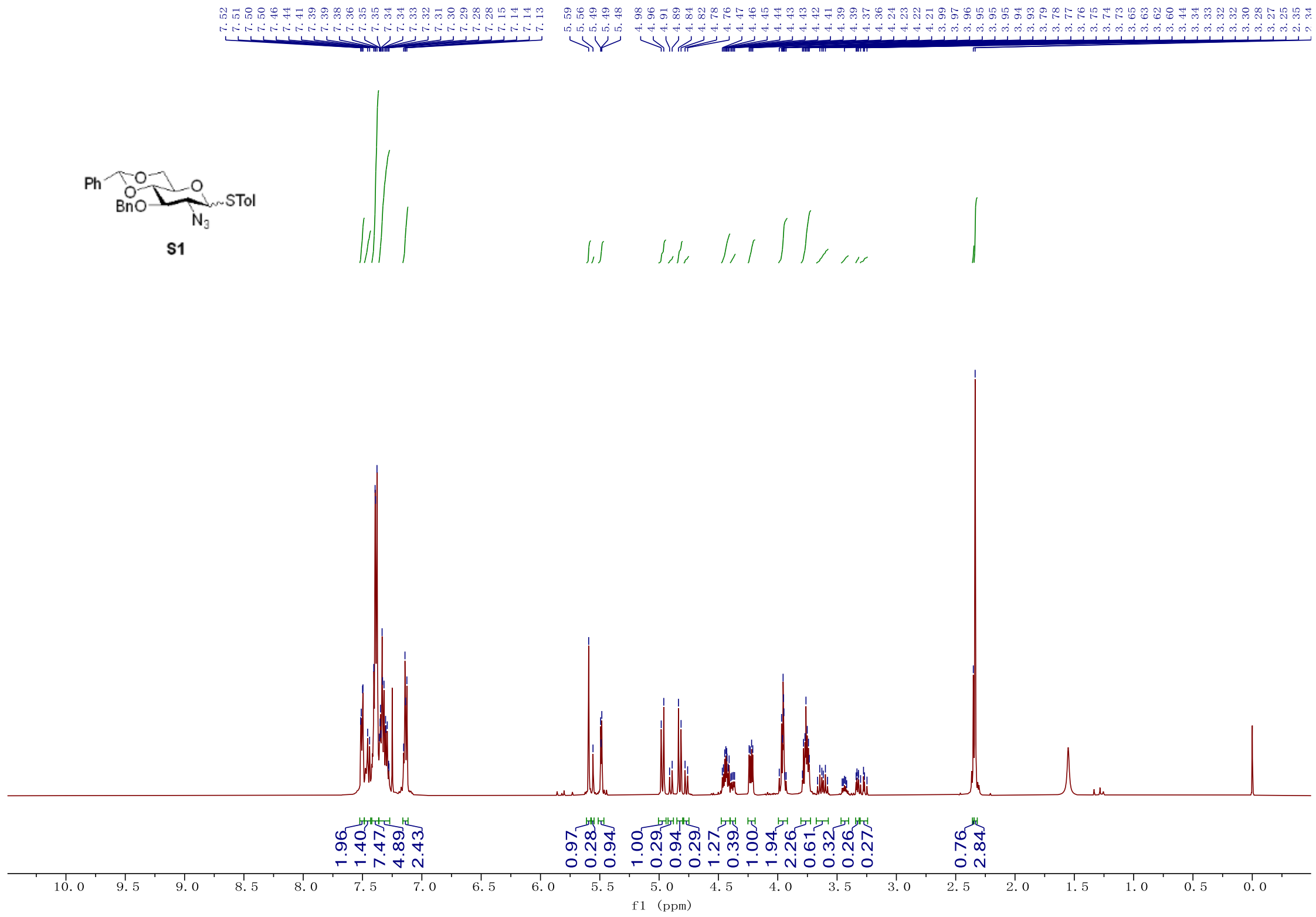

Figure S25. Compound **S1** ^{1}H NMR ($CDCl_3$, 500 MHz)

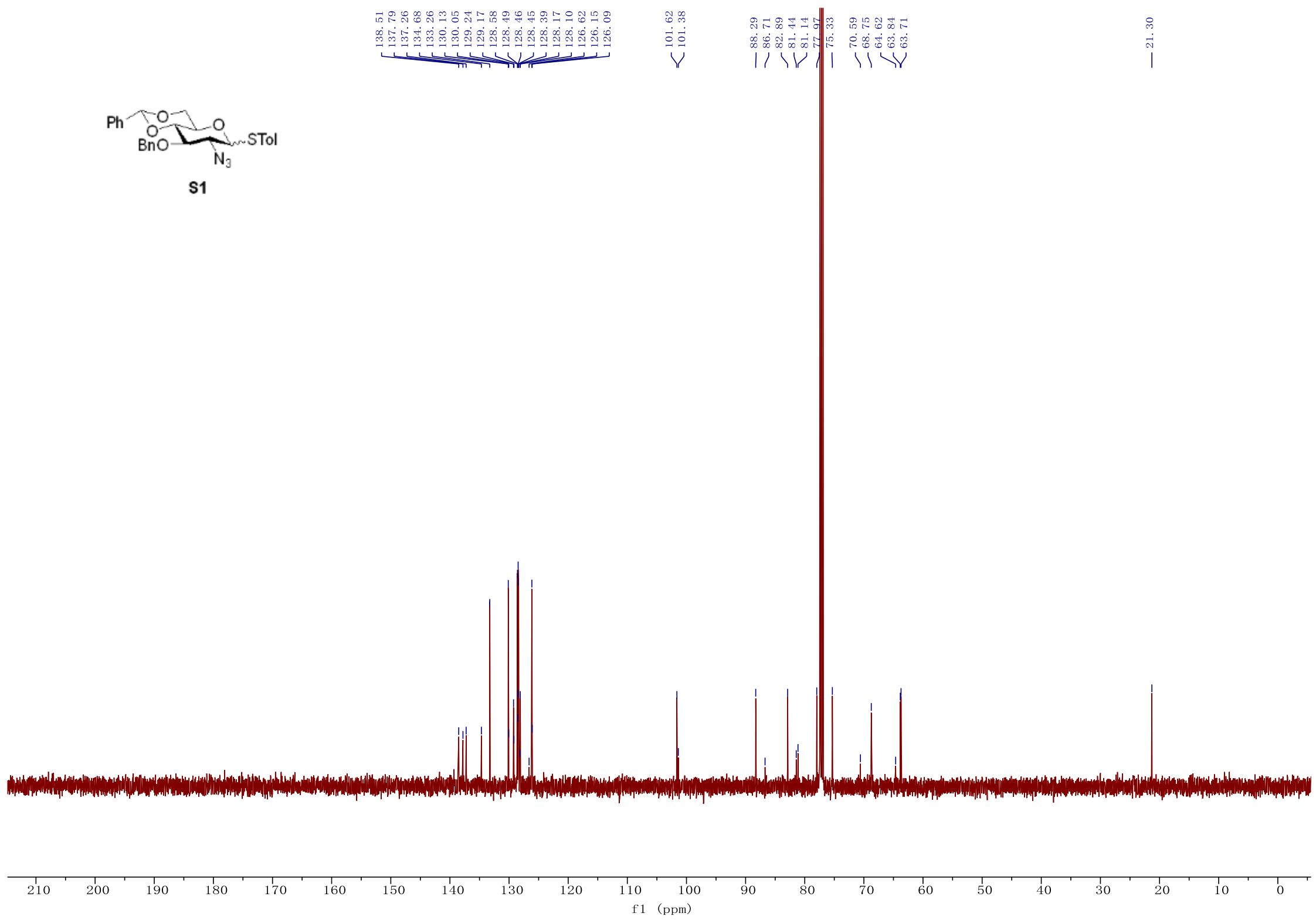

Figure S26. Compound **S1** ^{13}C NMR ($CDCl_3$, 126 MHz)

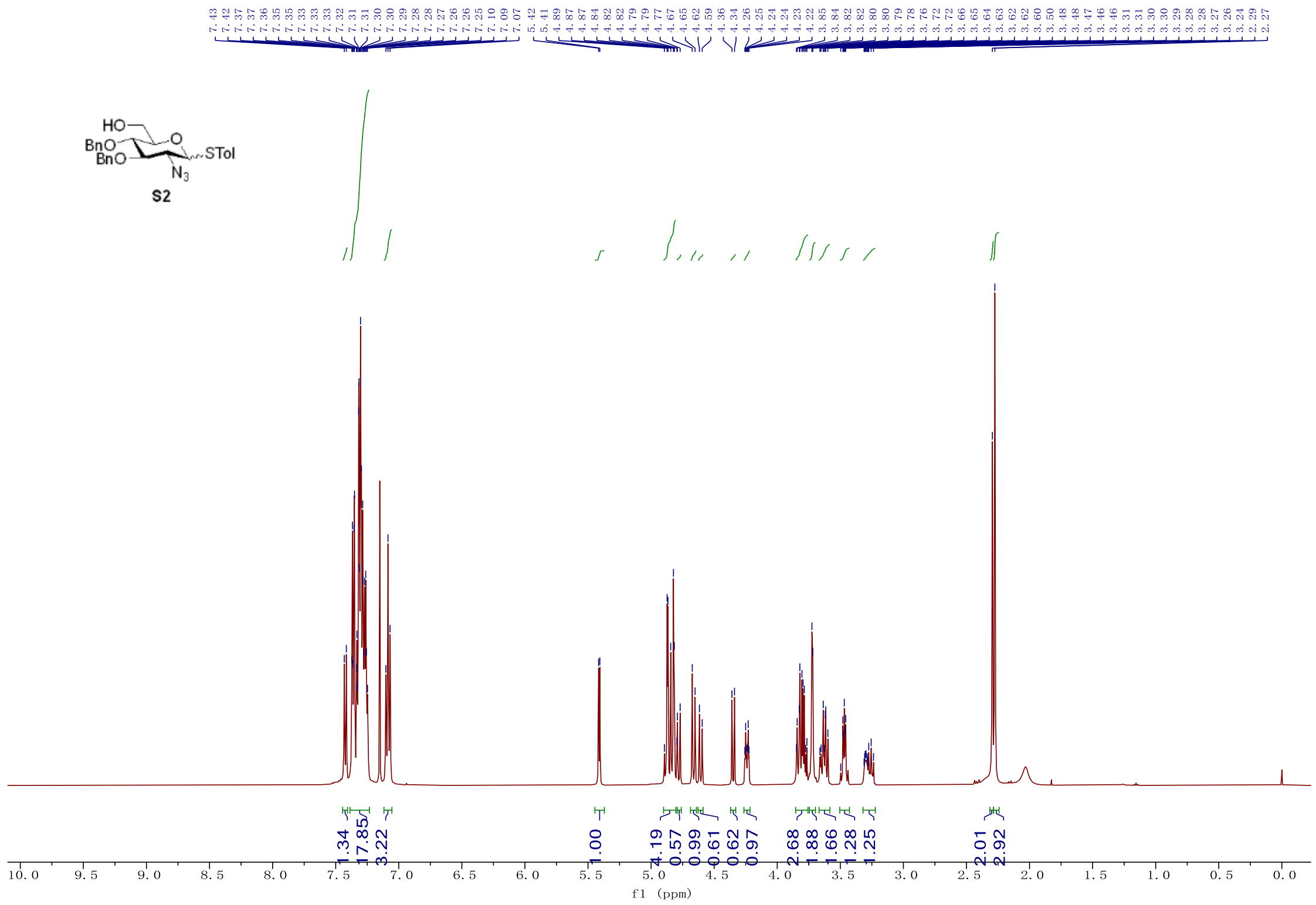

Figure S27. Compound **S2** ^{1}H NMR ($CDCl_3$, 500 MHz)

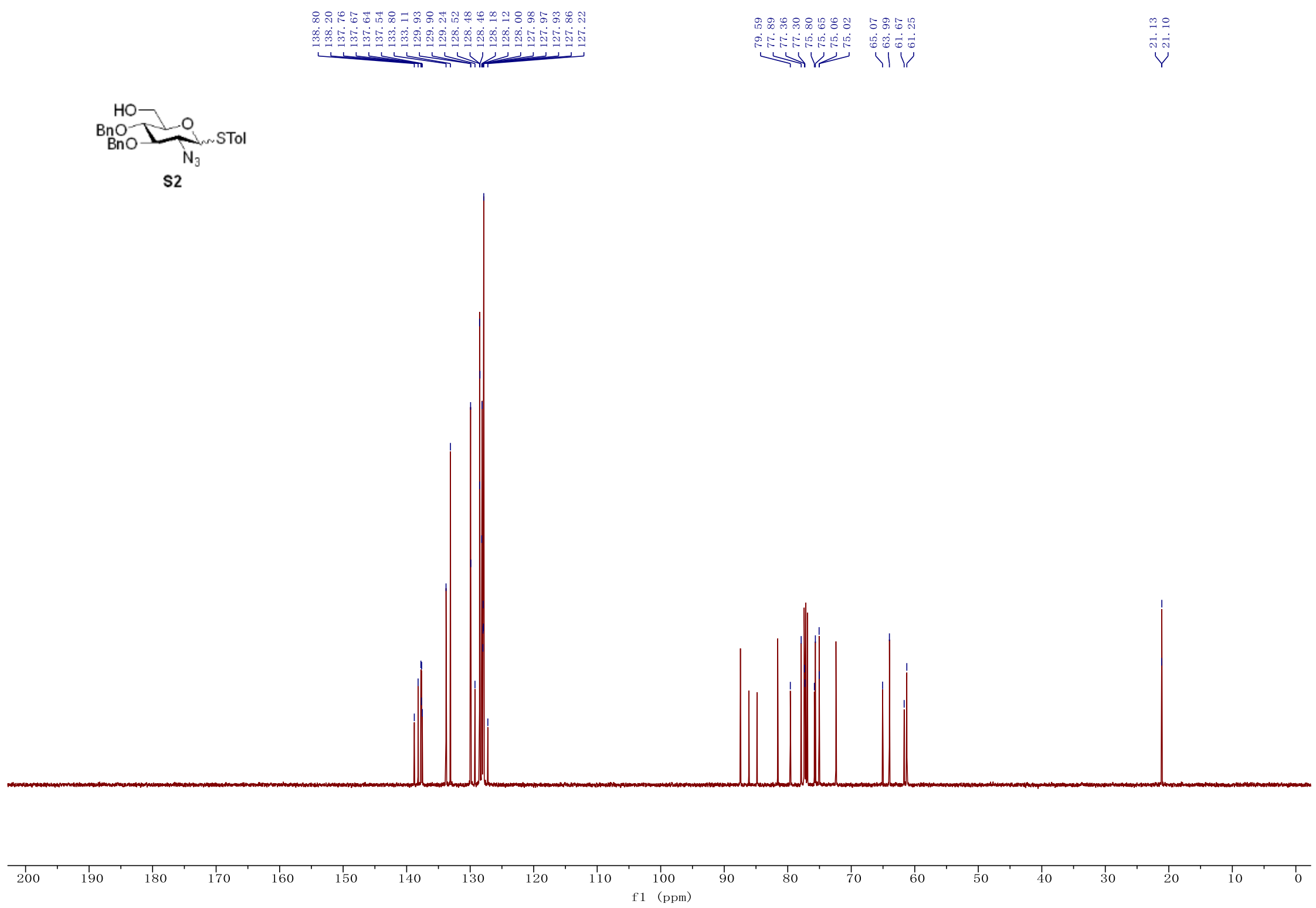

Figure S28. Compound **S2** ^{13}C NMR ($CDCl_3$, 126 MHz)

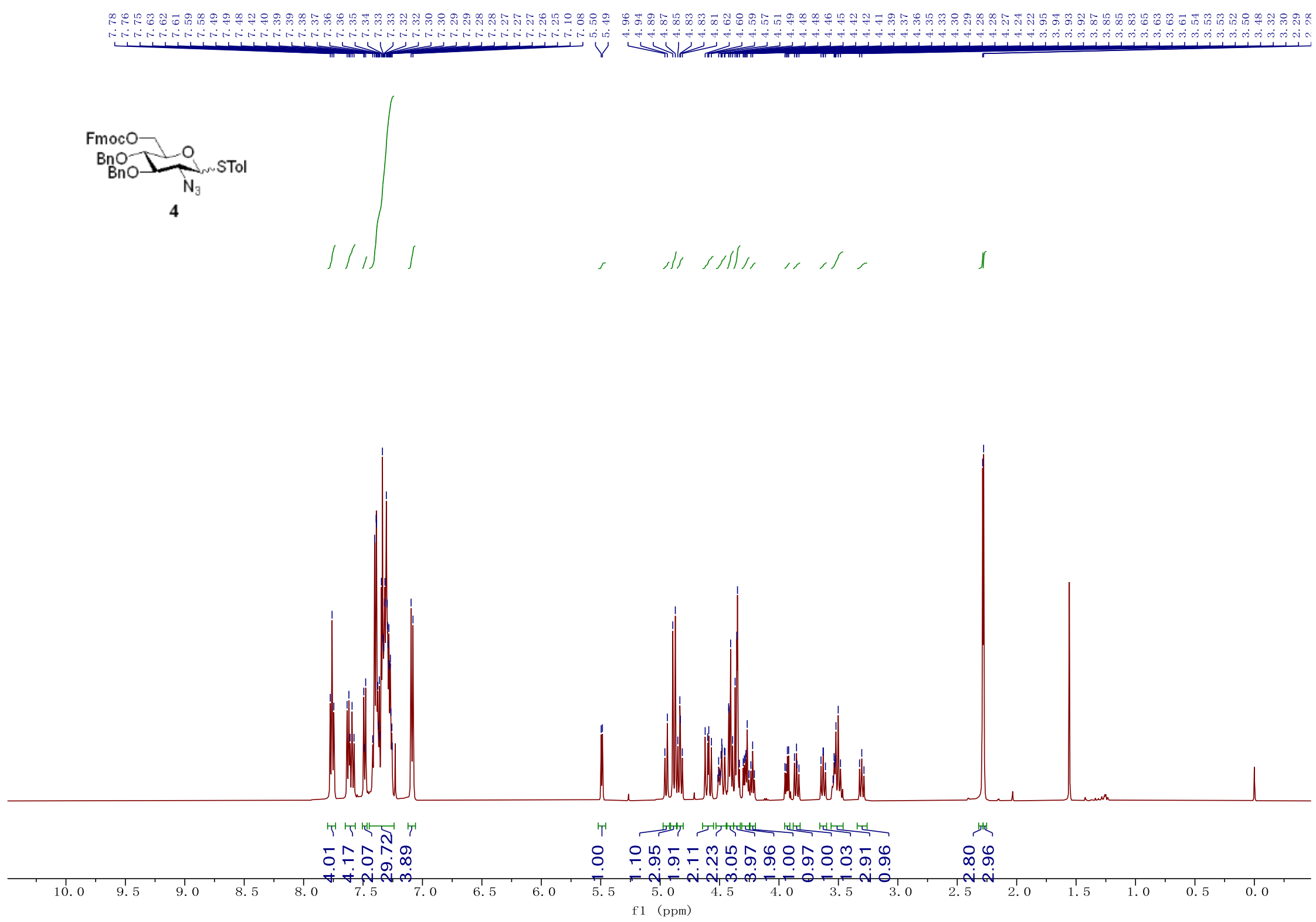

Figure S29. Compound **4** ^{1}H NMR ($CDCl_3$, 500 MHz)

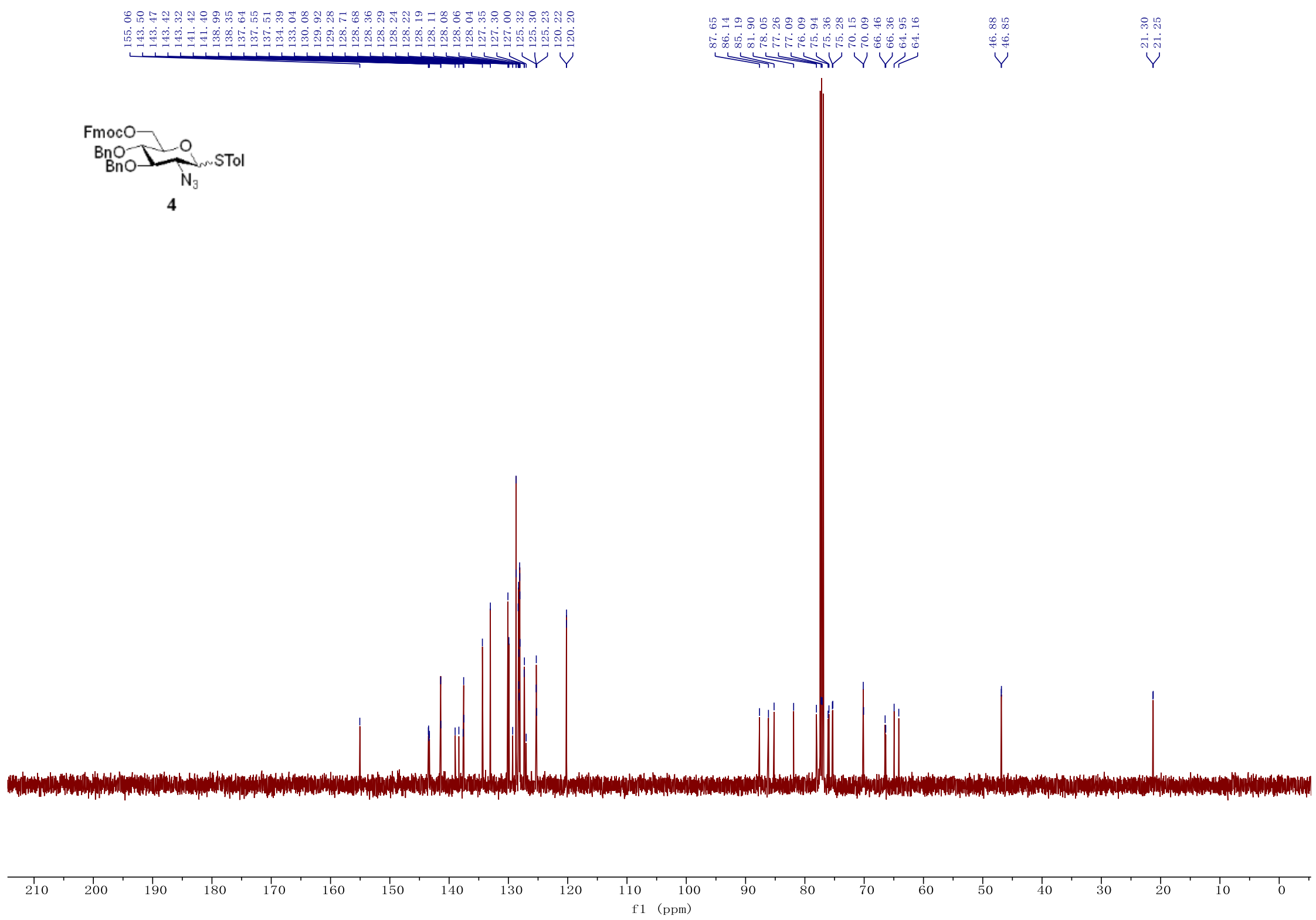

Figure S30. Compound **4** ^{13}C NMR ($CDCl_3$, 126 MHz)

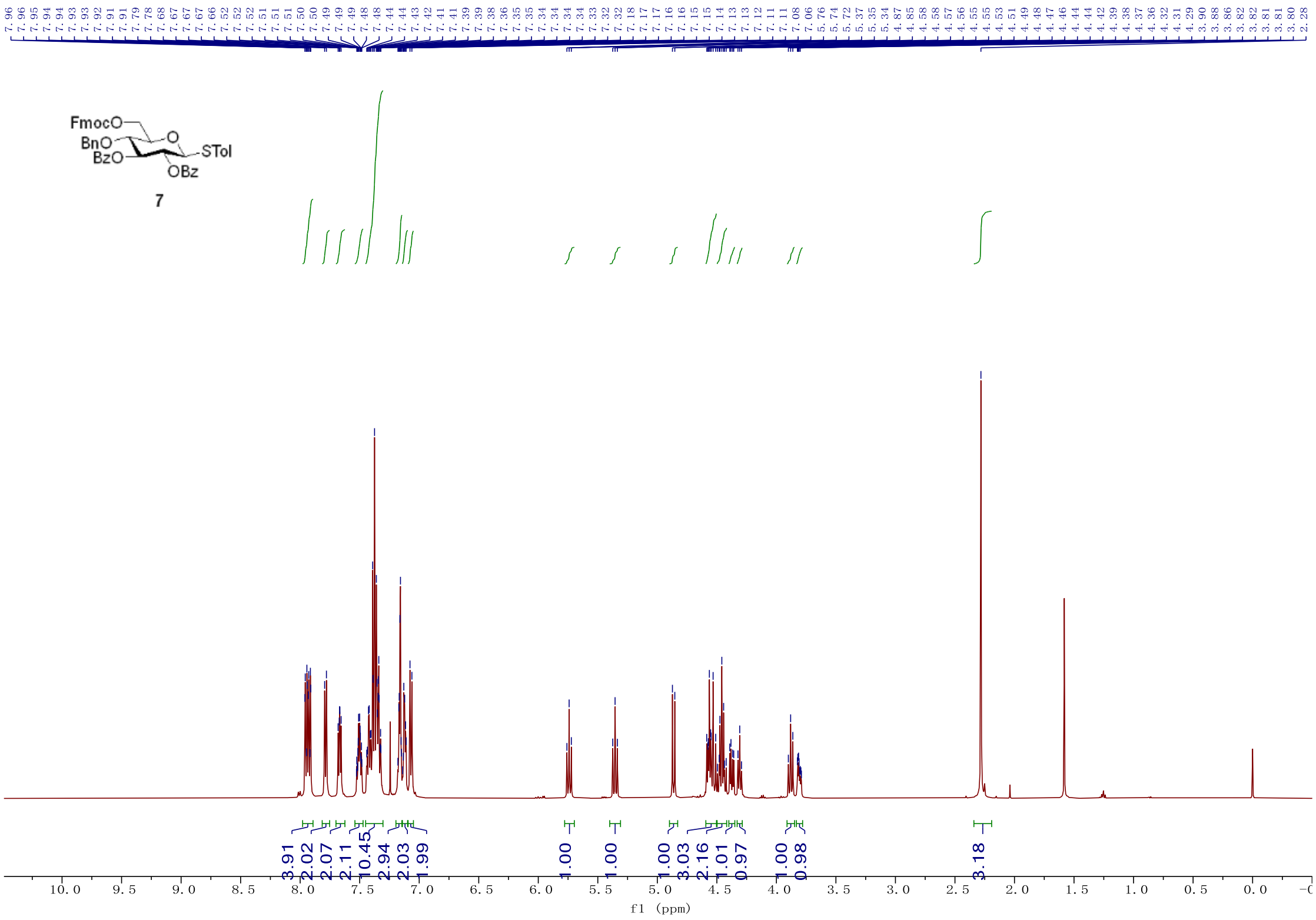

Figure S31. Compound **7** ^{1}H NMR ($CDCl_3$, 500 MHz)

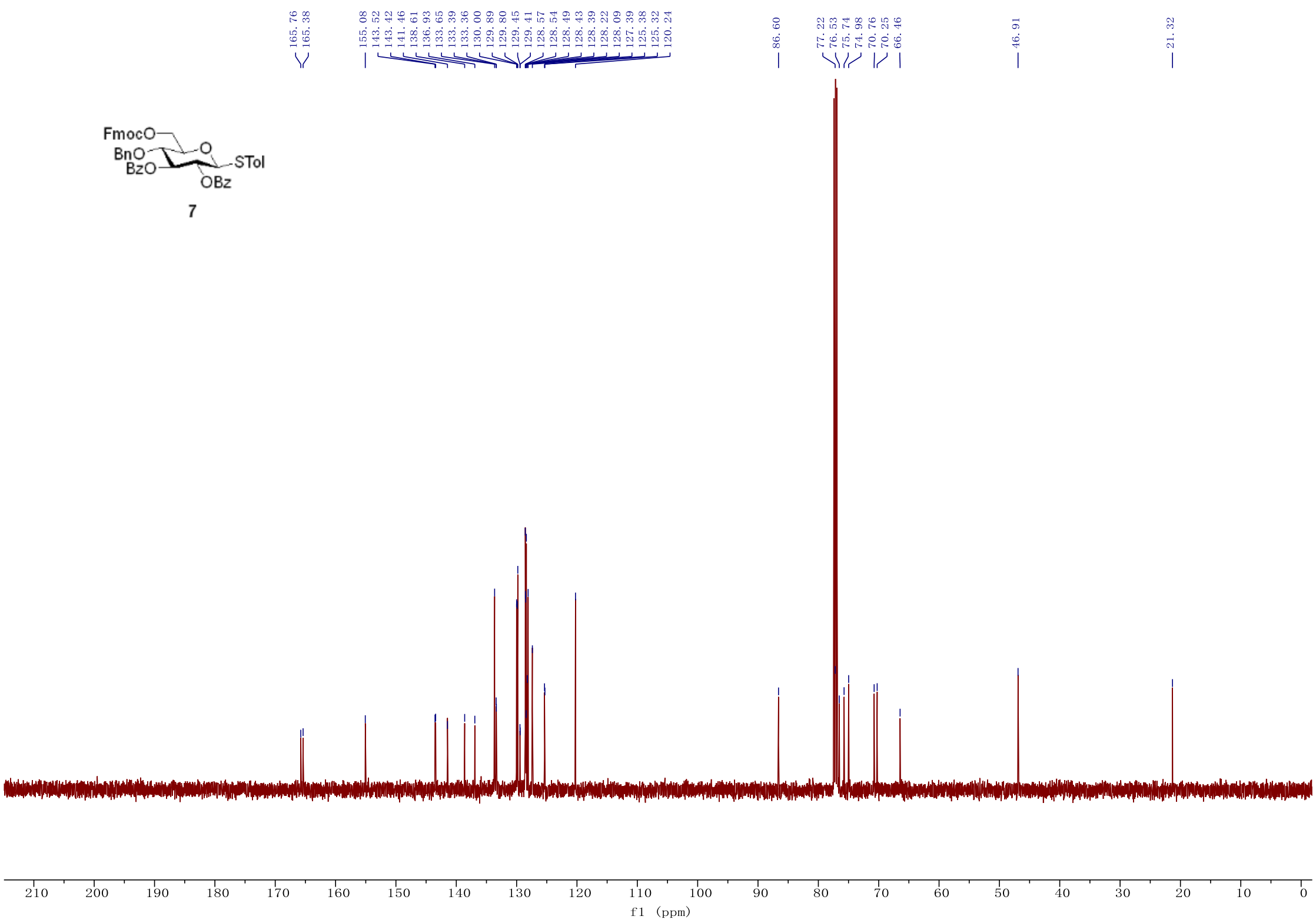

Figure S32. Compound **7** ^{13}C NMR ($CDCl_3$, 126 MHz)

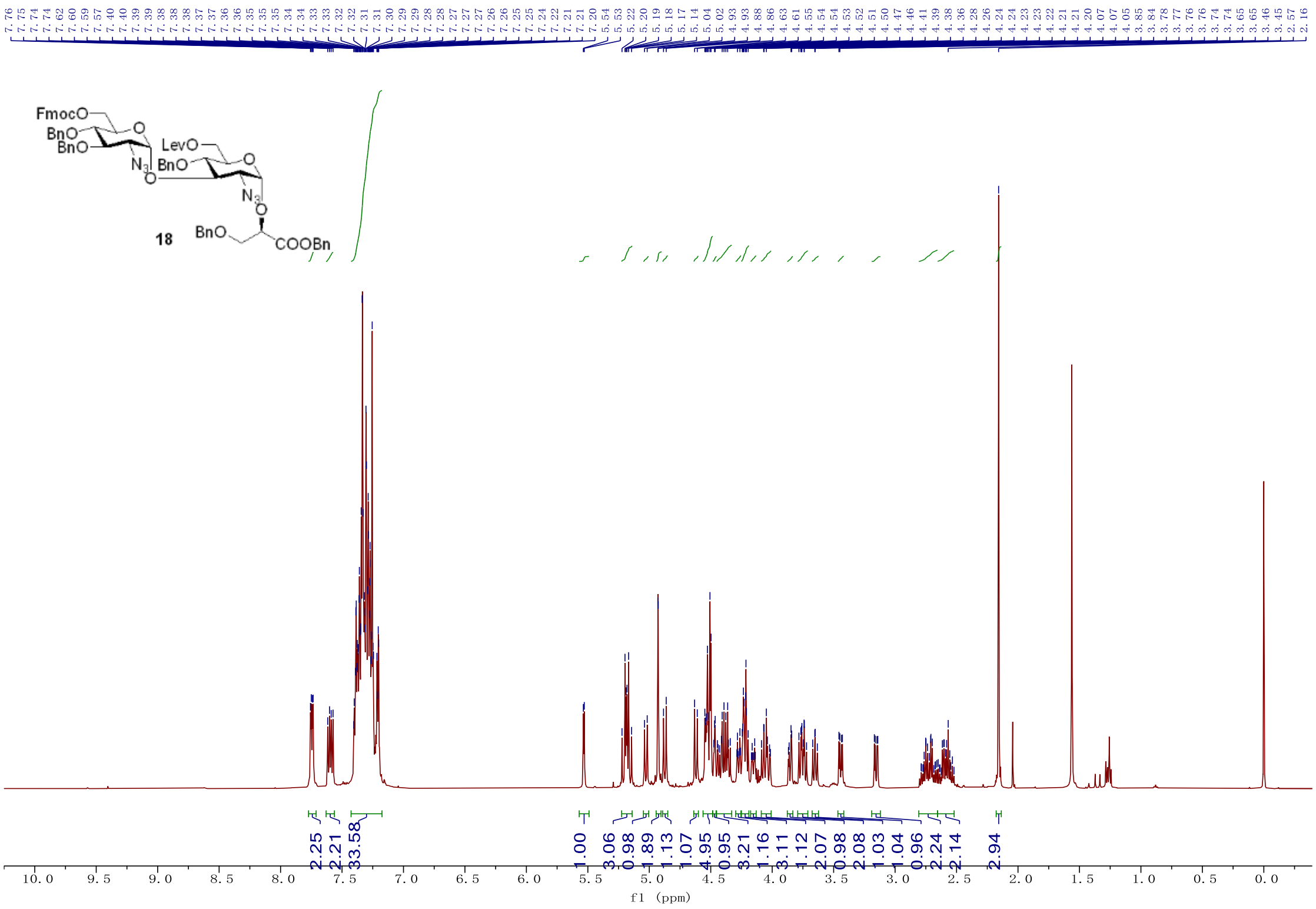

Figure S33. Compound **18** ^{1}H NMR ($CDCl_3$, 500 MHz)

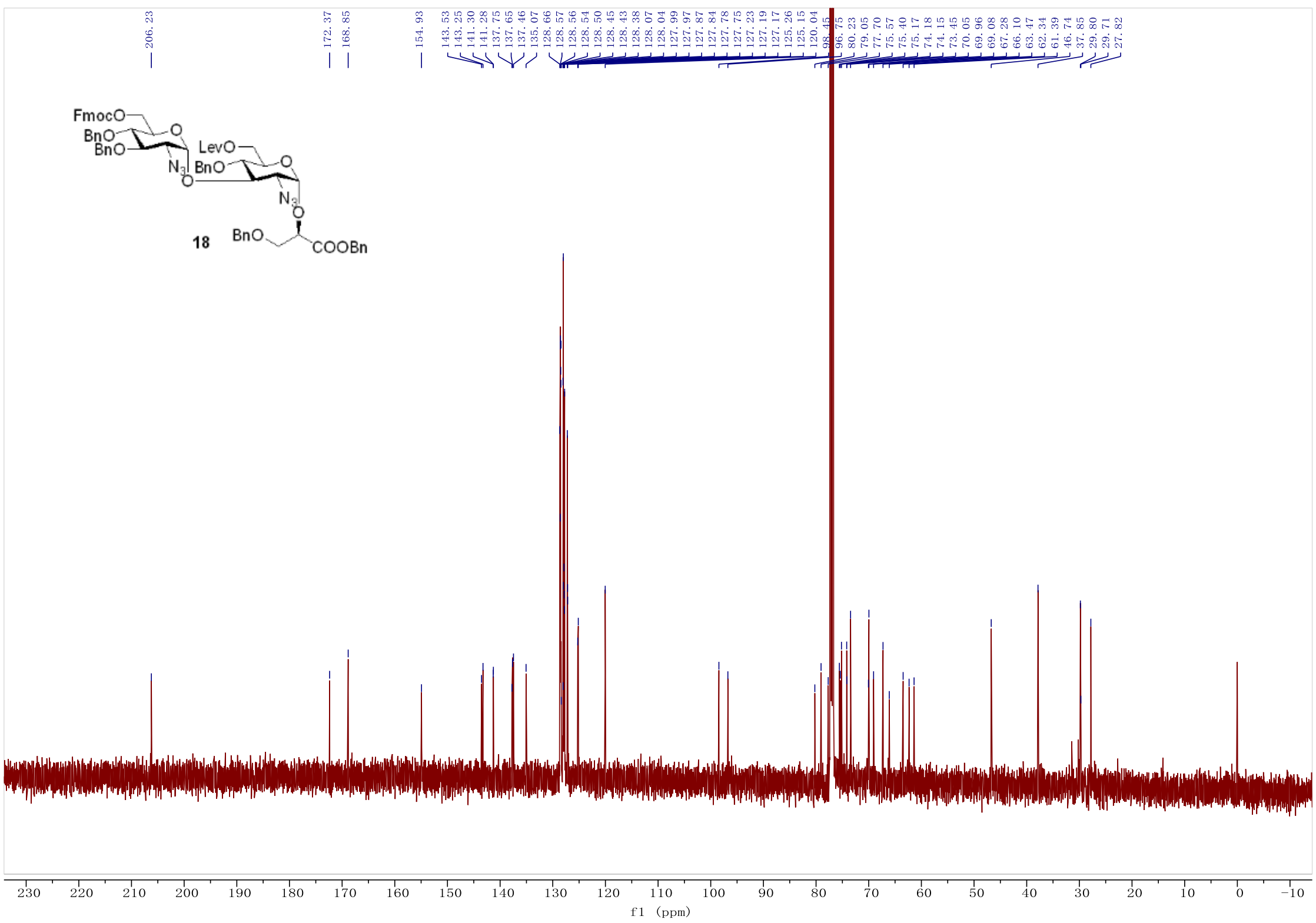

Figure S34. Compound **18** ^{13}C NMR ($CDCl_3$, 126 MHz)

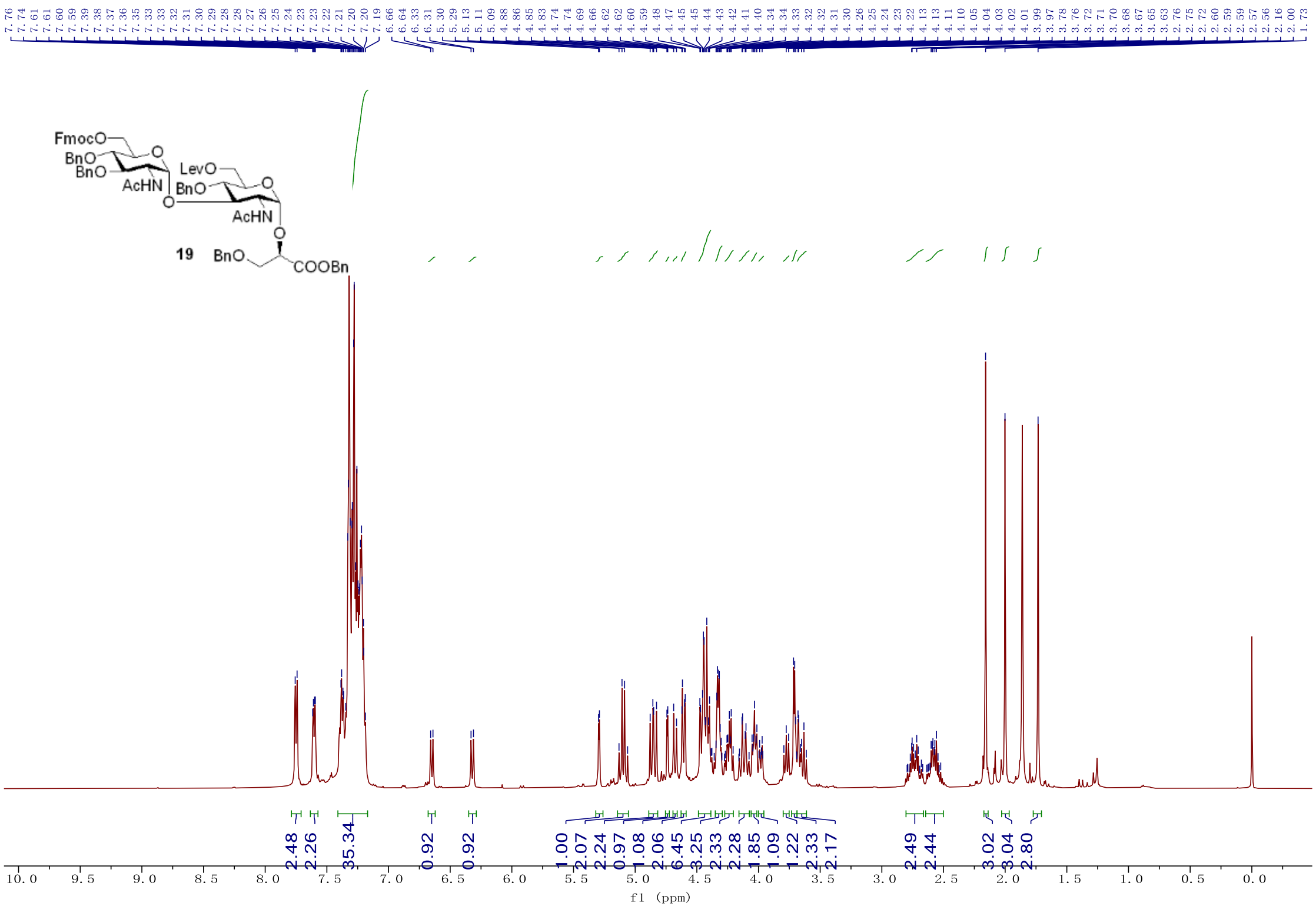

Figure S35. Compound **19** ^{1}H NMR ($CDCl_3$, 500 MHz)

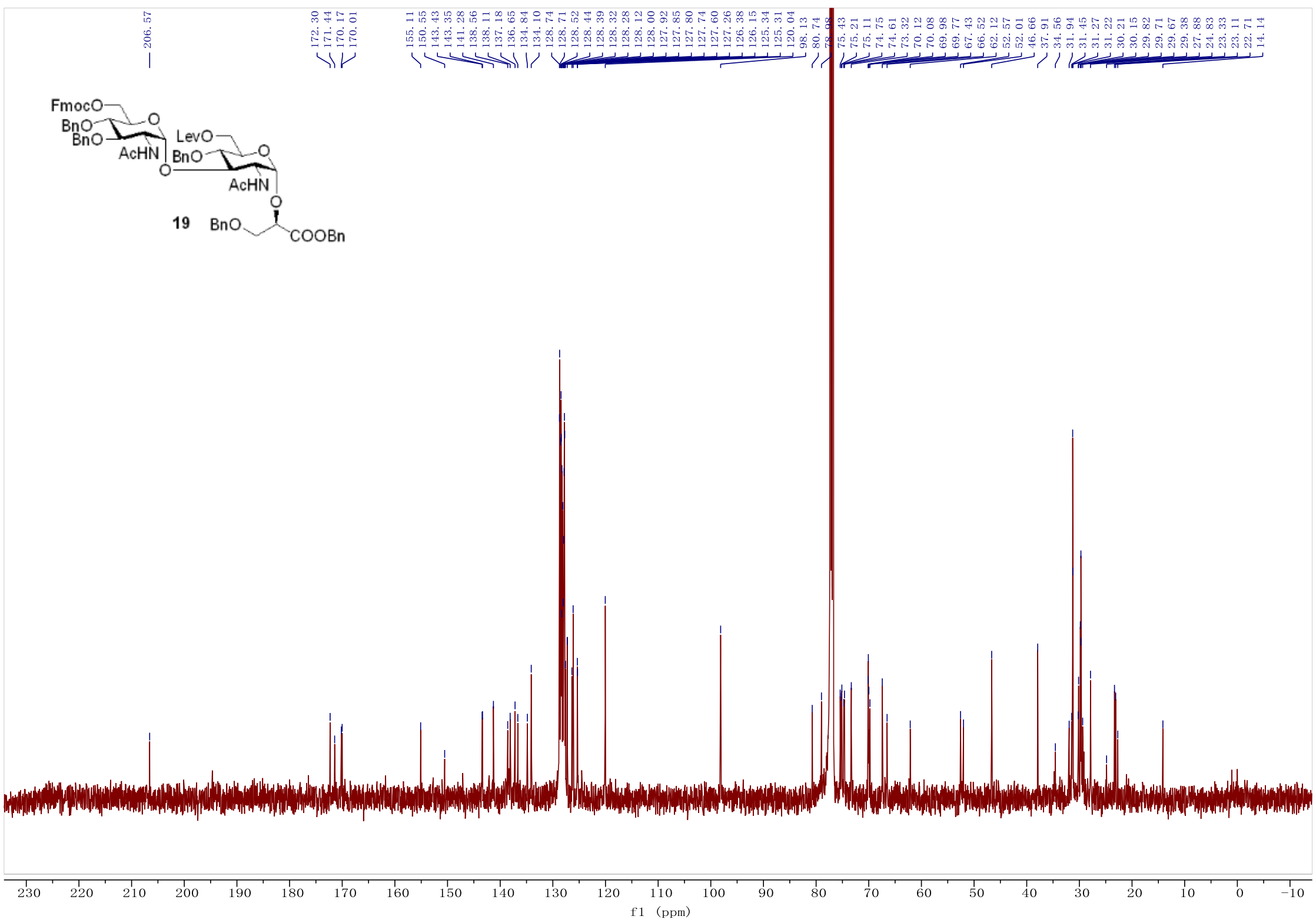

Figure S36. Compound **19** ^{13}C NMR ($CDCl_3$, 126 MHz)

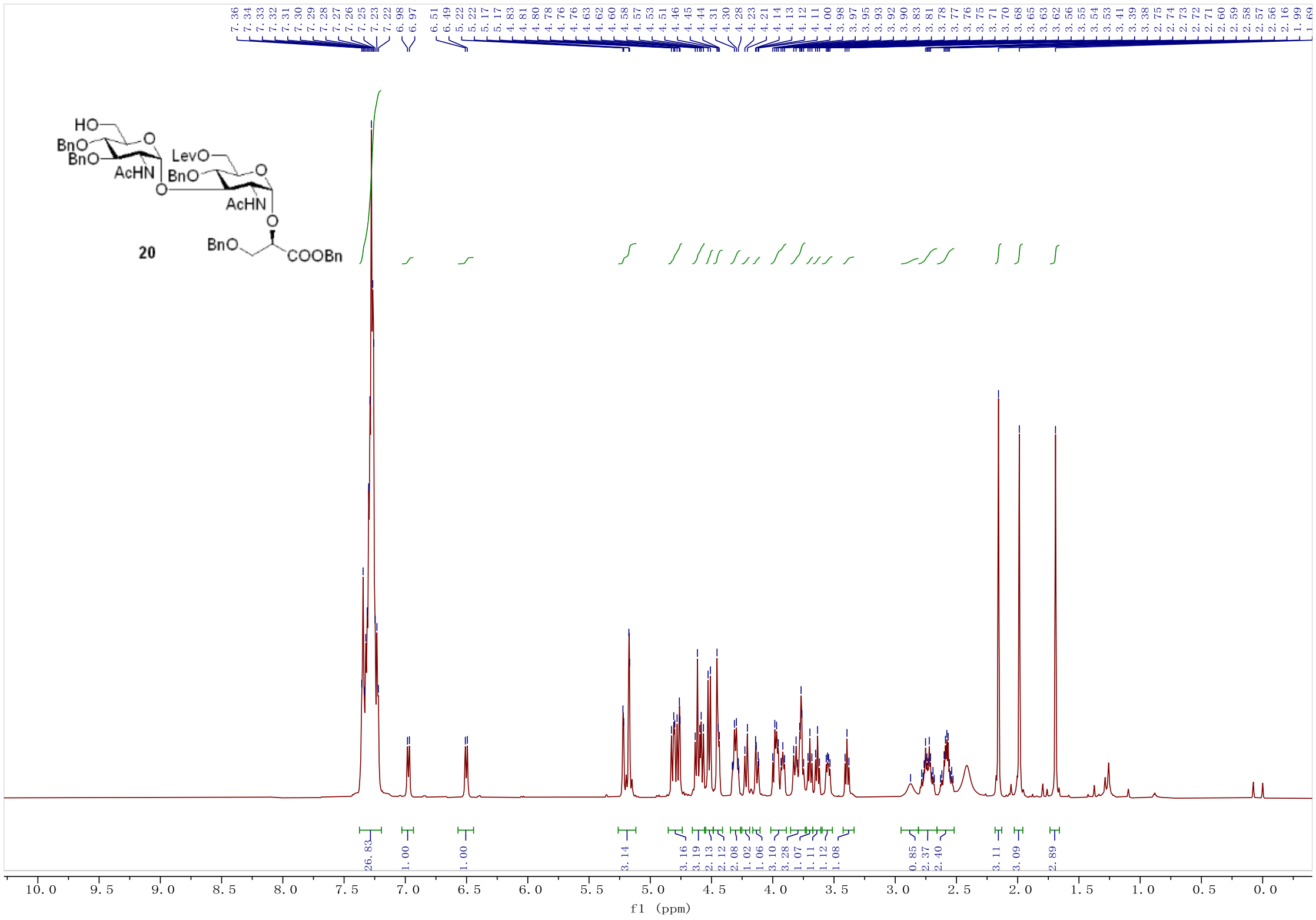

Figure S37. Compound **20** ^{1}H NMR ($CDCl_3$, 500 MHz)

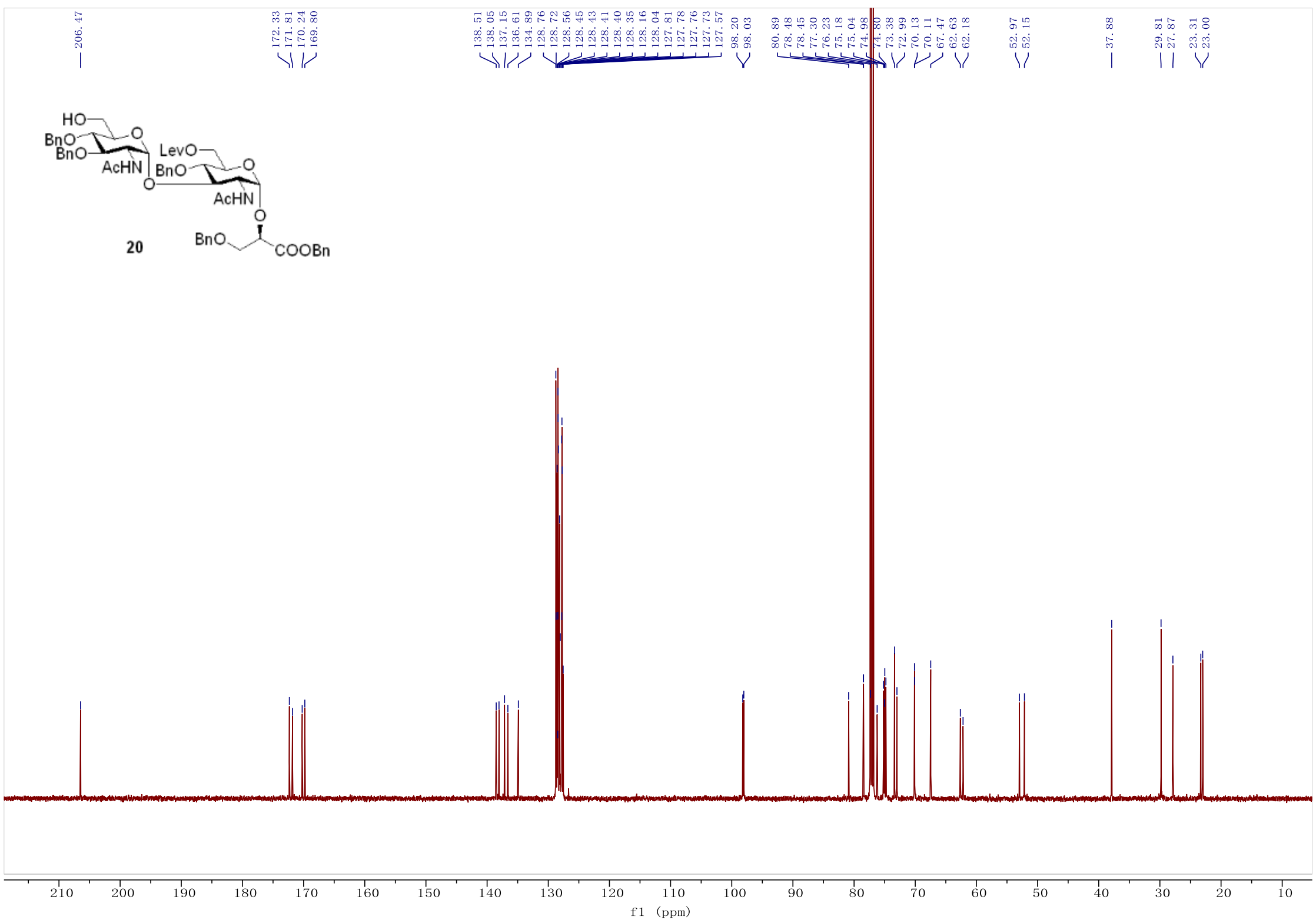

Figure S38. Compound **20** ^{13}C NMR ($CDCl_3$, 126 MHz)

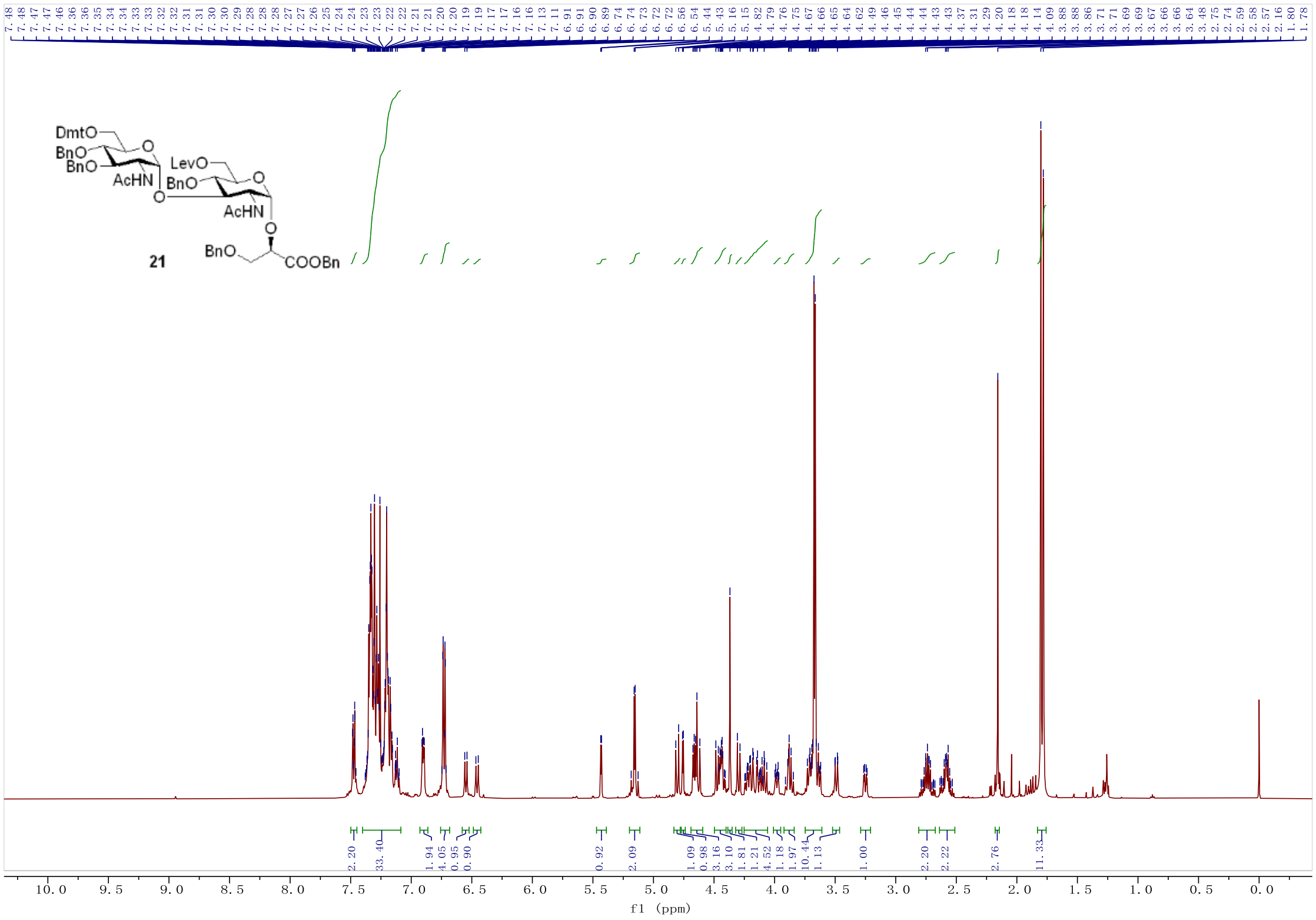

Figure S39. Compound **21** ^{1}H NMR ($CDCl_3$, 500 MHz)

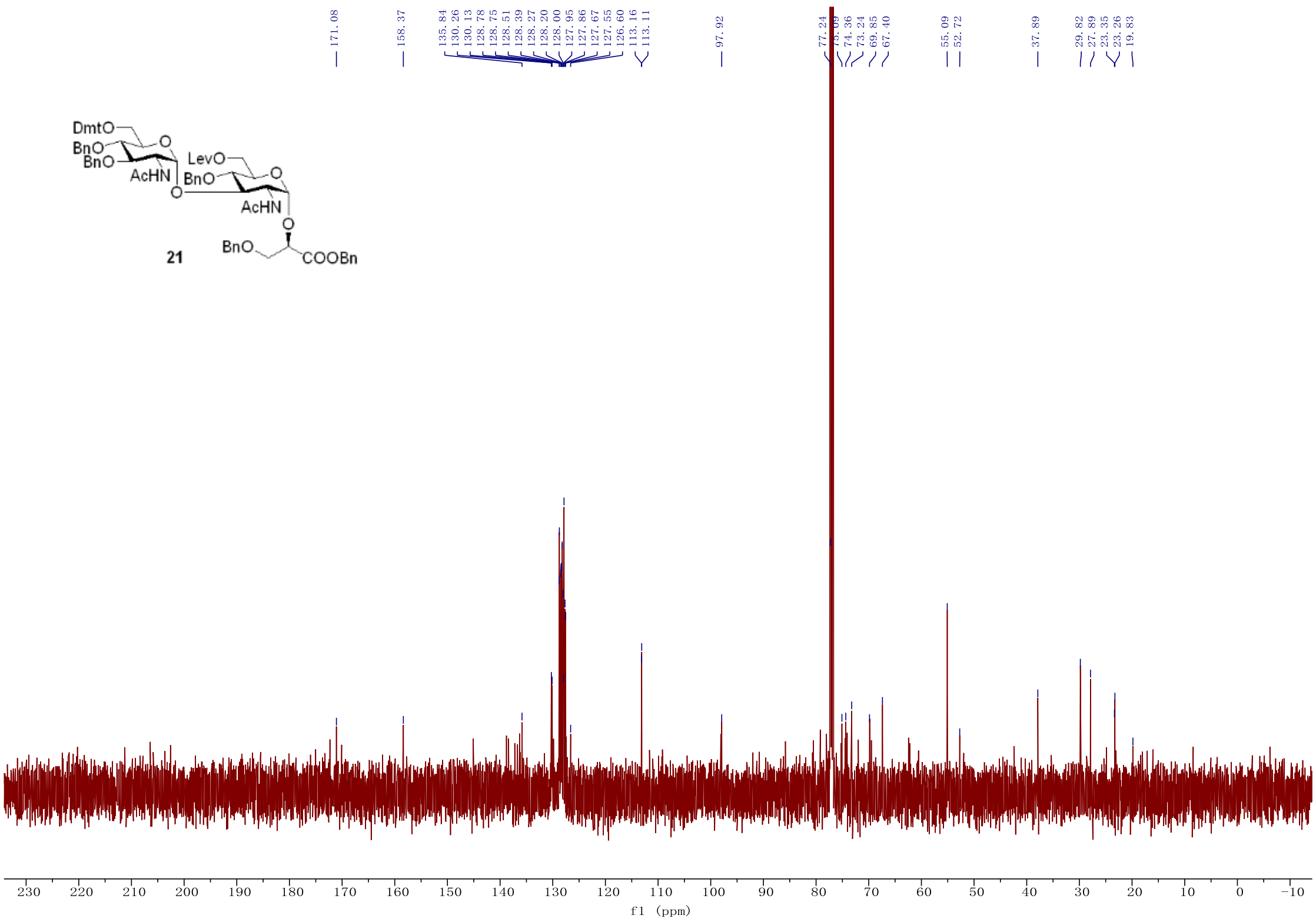

Figure S40. Compound **21** ^{13}C NMR ($CDCl_3$, 126 MHz)

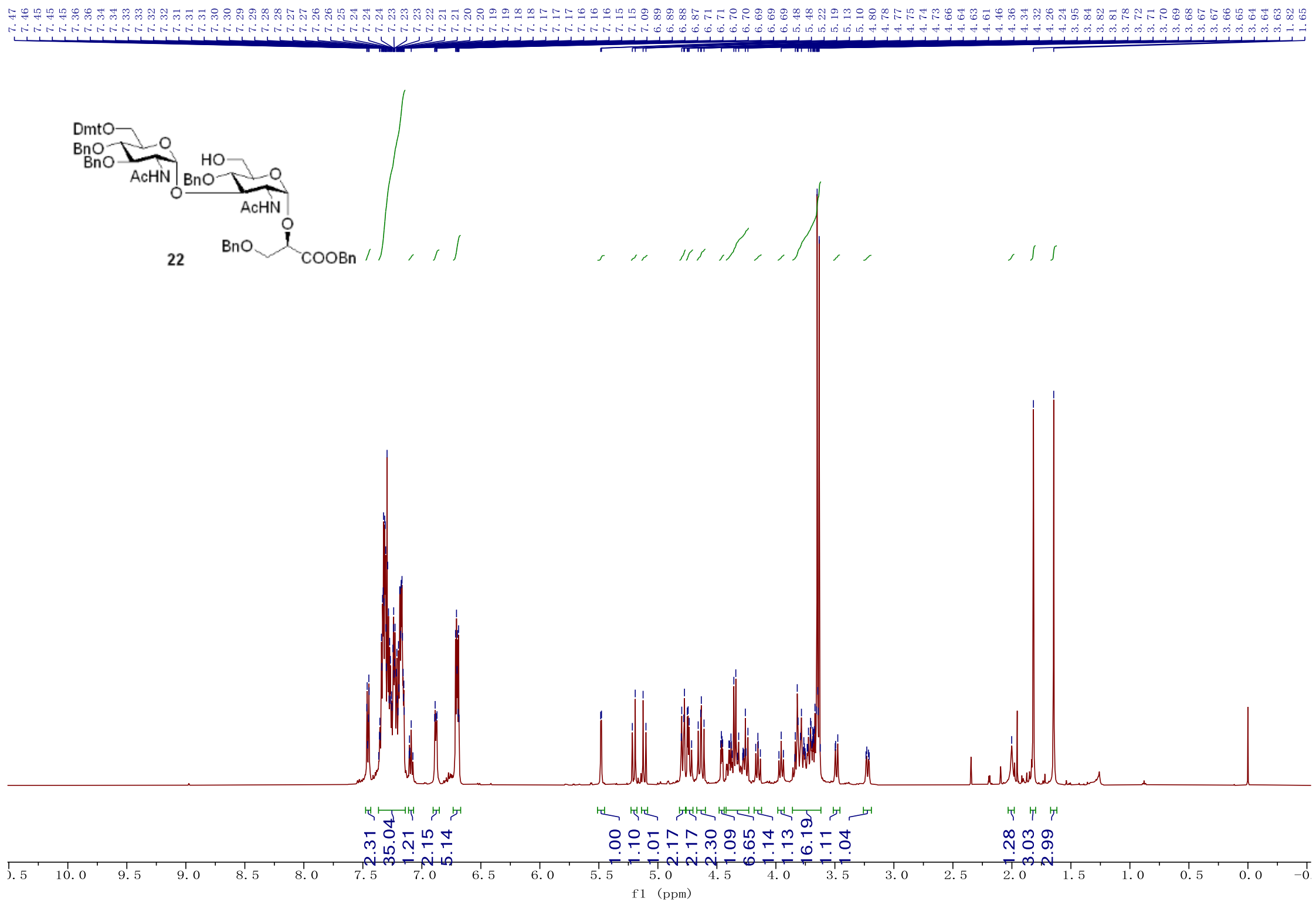

Figure S41. Compound **22** ^{1}H NMR ($CDCl_3$, 500 MHz)

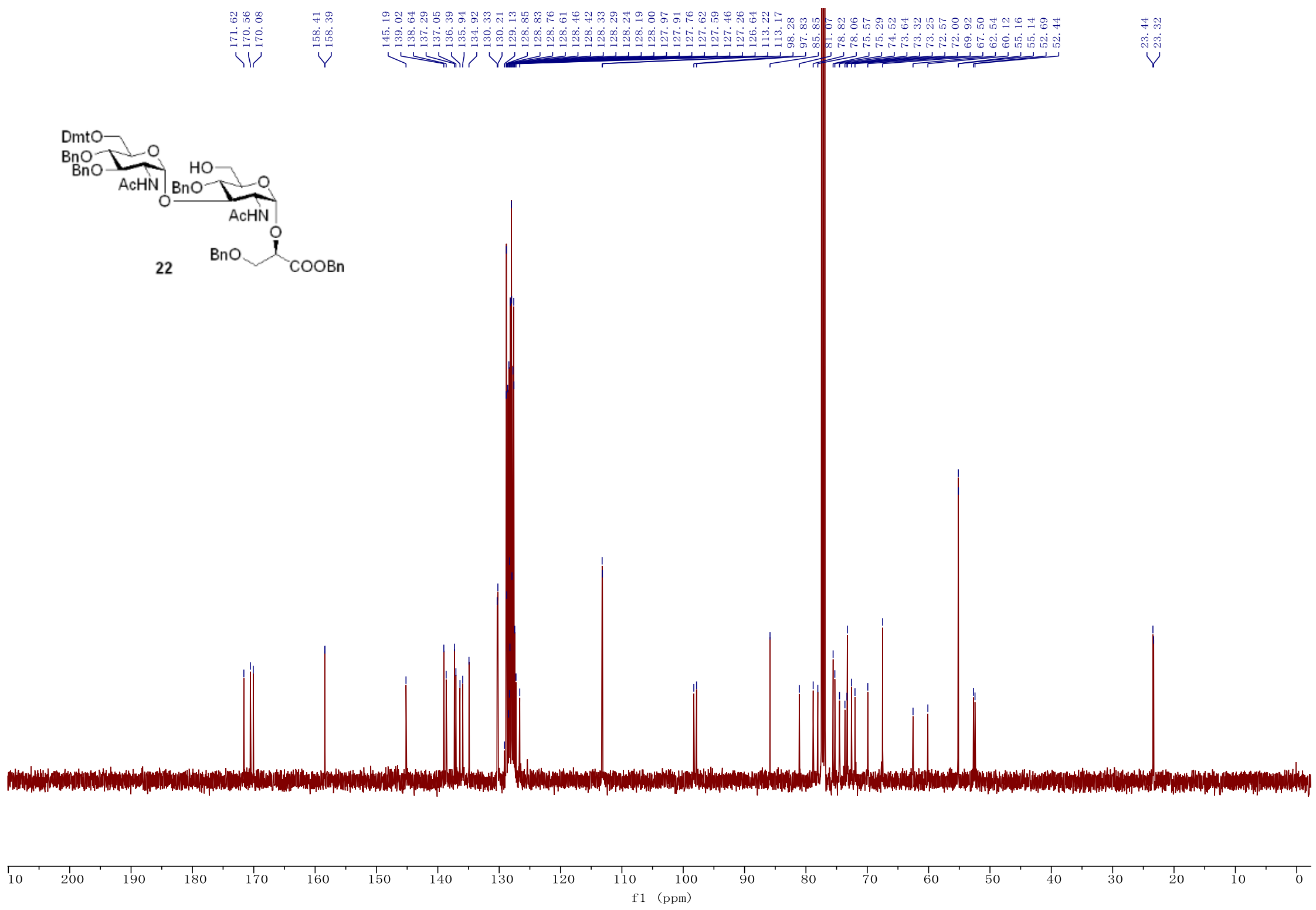

Figure S42. Compound **22** ^{13}C NMR ($CDCl_3$, 126 MHz)

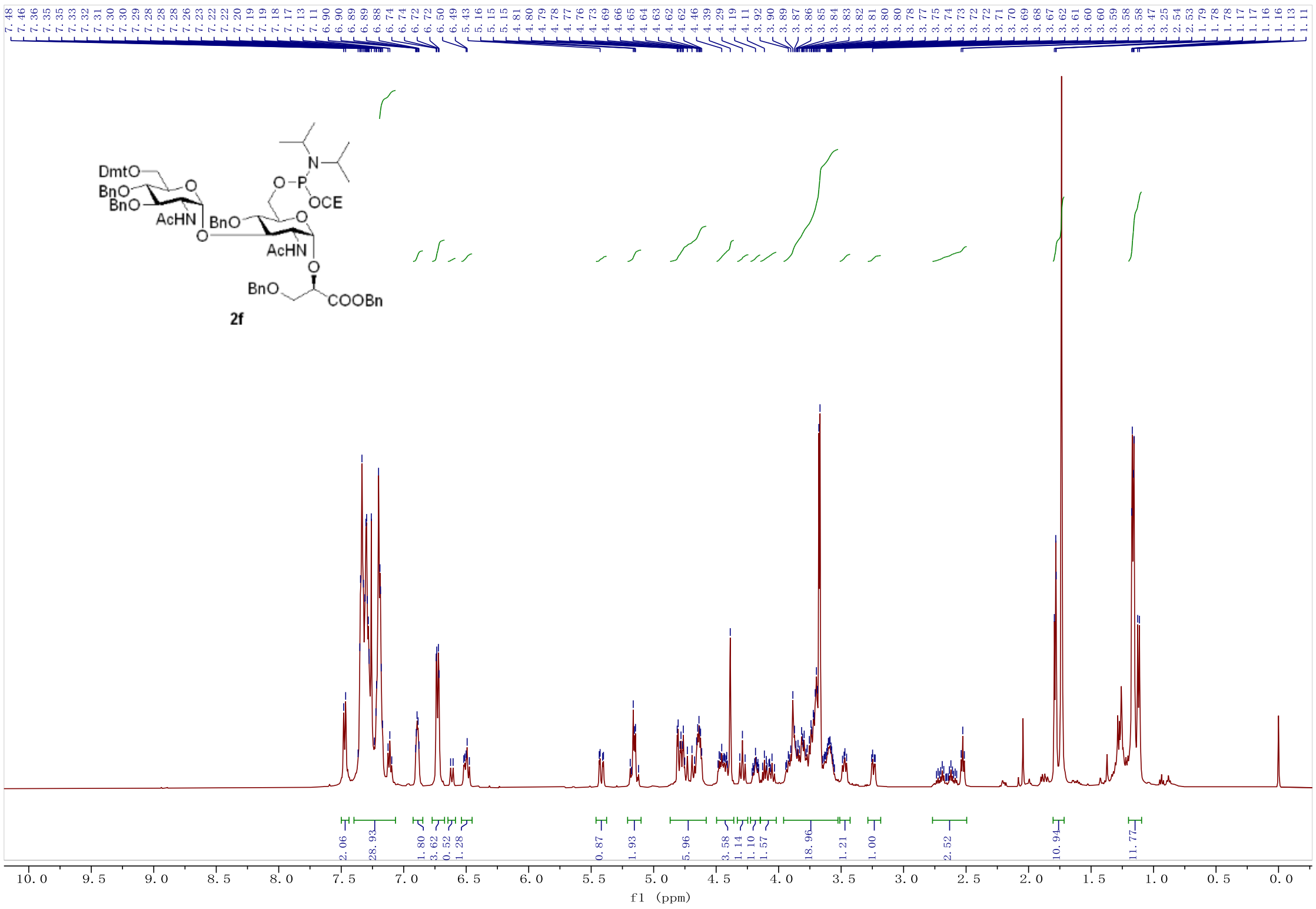

Figure S43. Compound **2f** ^{1}H NMR ($CDCl_3$, 500 MHz)

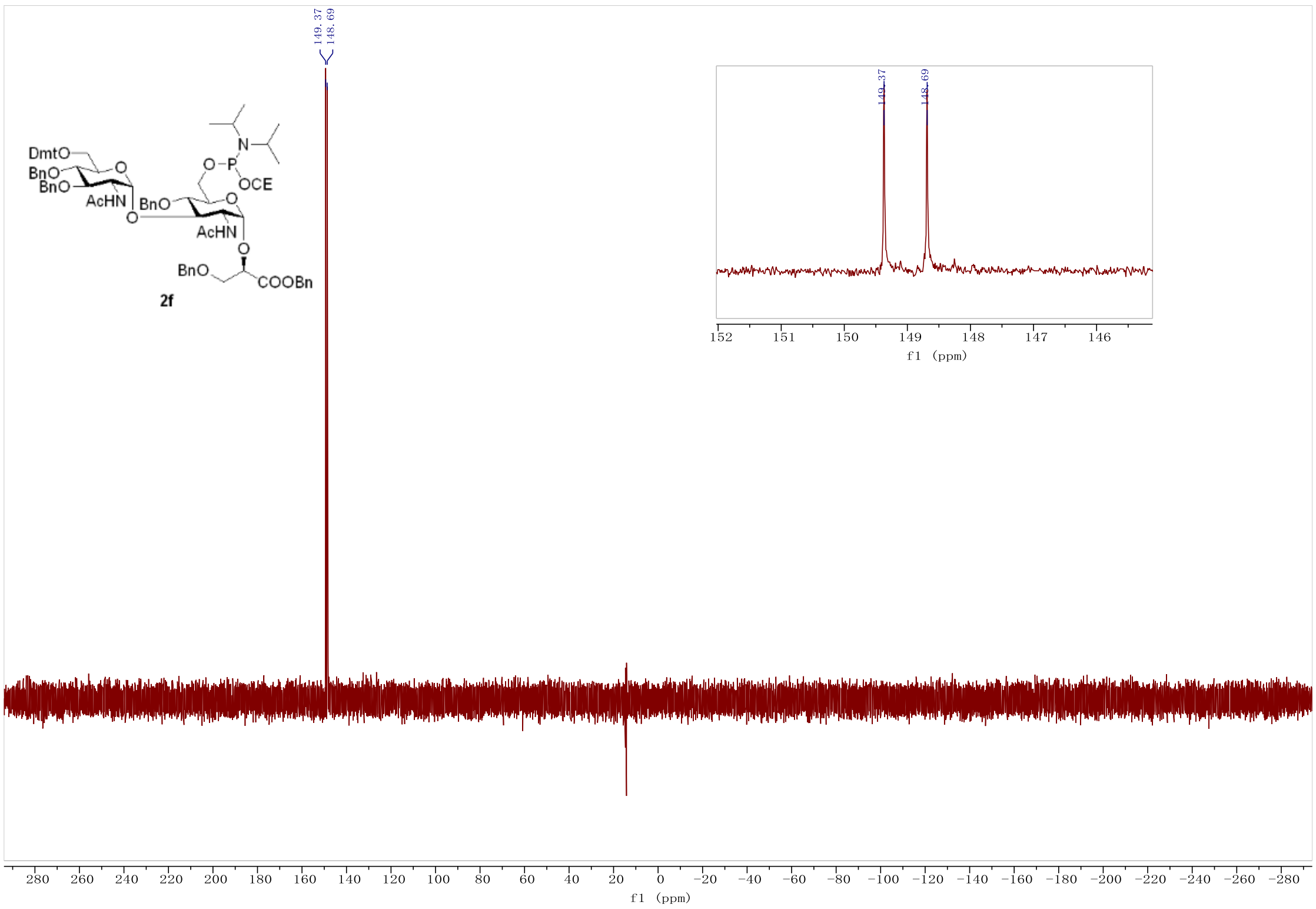

Figure S44. Compound **2f** ^{31}P NMR ($CDCl_3$, 202 MHz)

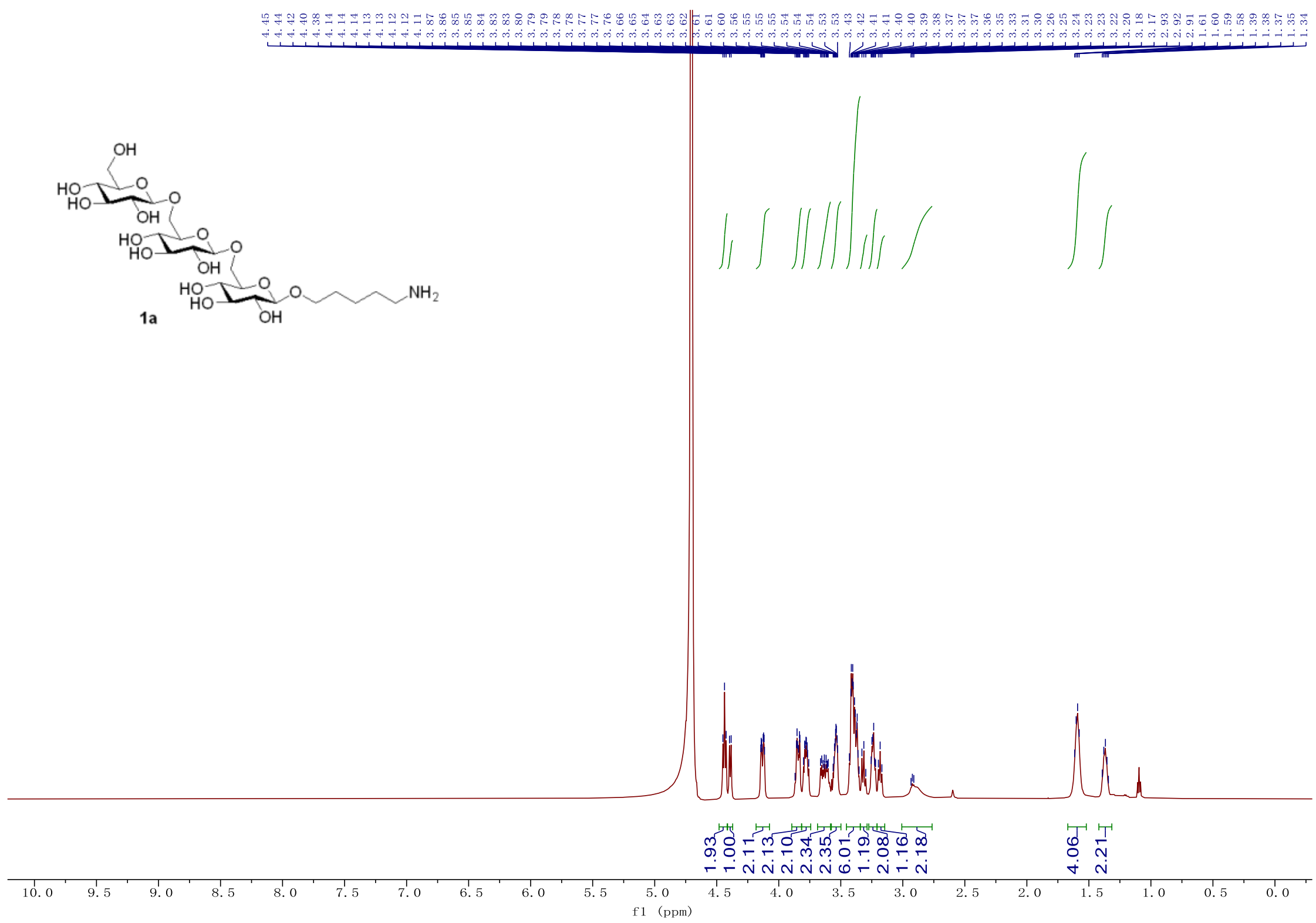

Figure S45. Compound **1a** ^{1}H NMR (D_2O, 600 MHz)

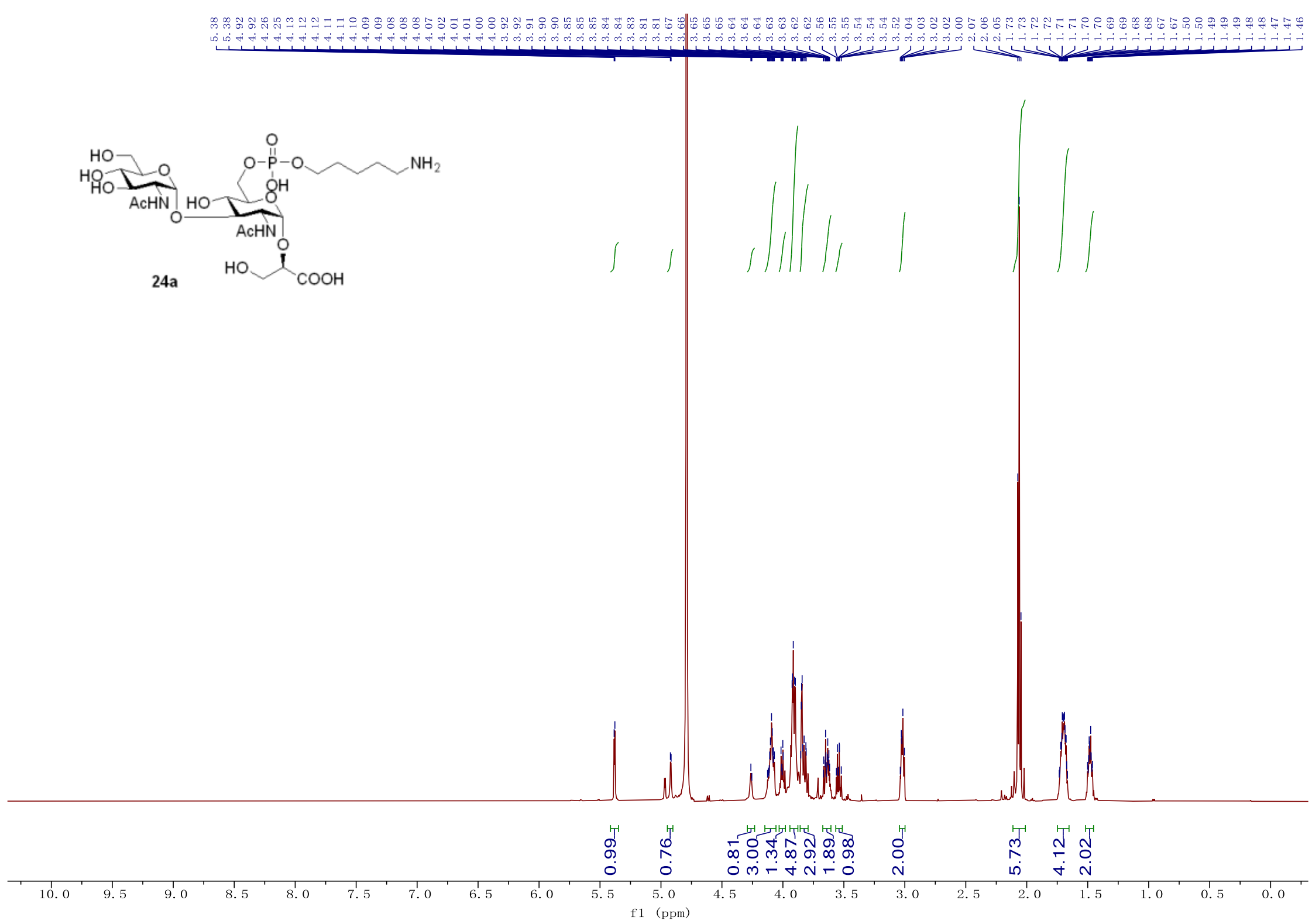

Figure S46. Compound **24a** ^{1}H NMR (D_2O, 600 MHz)

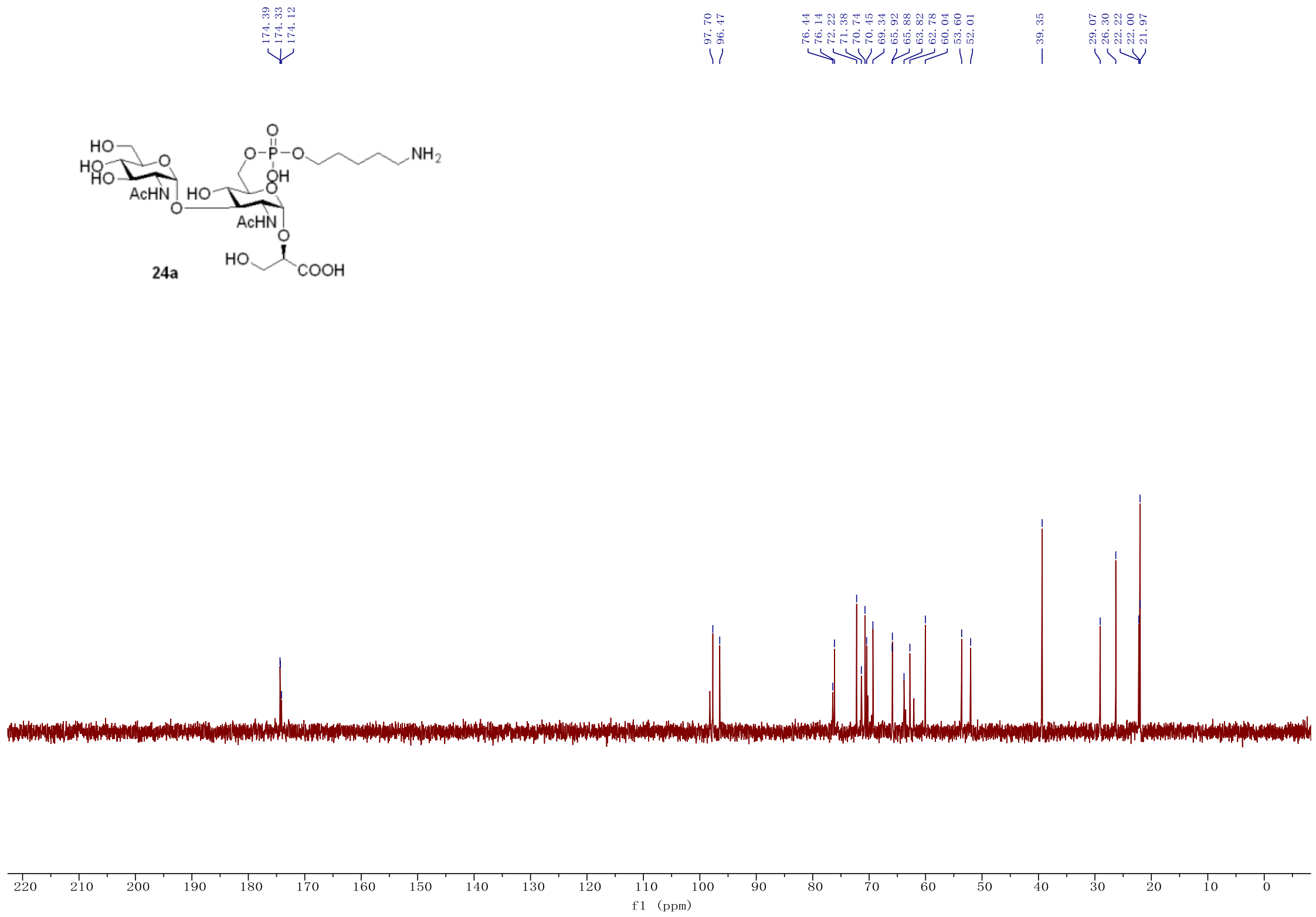

Figure S47. Compound **24a** ^{13}C NMR (D_2O, 151 MHz)

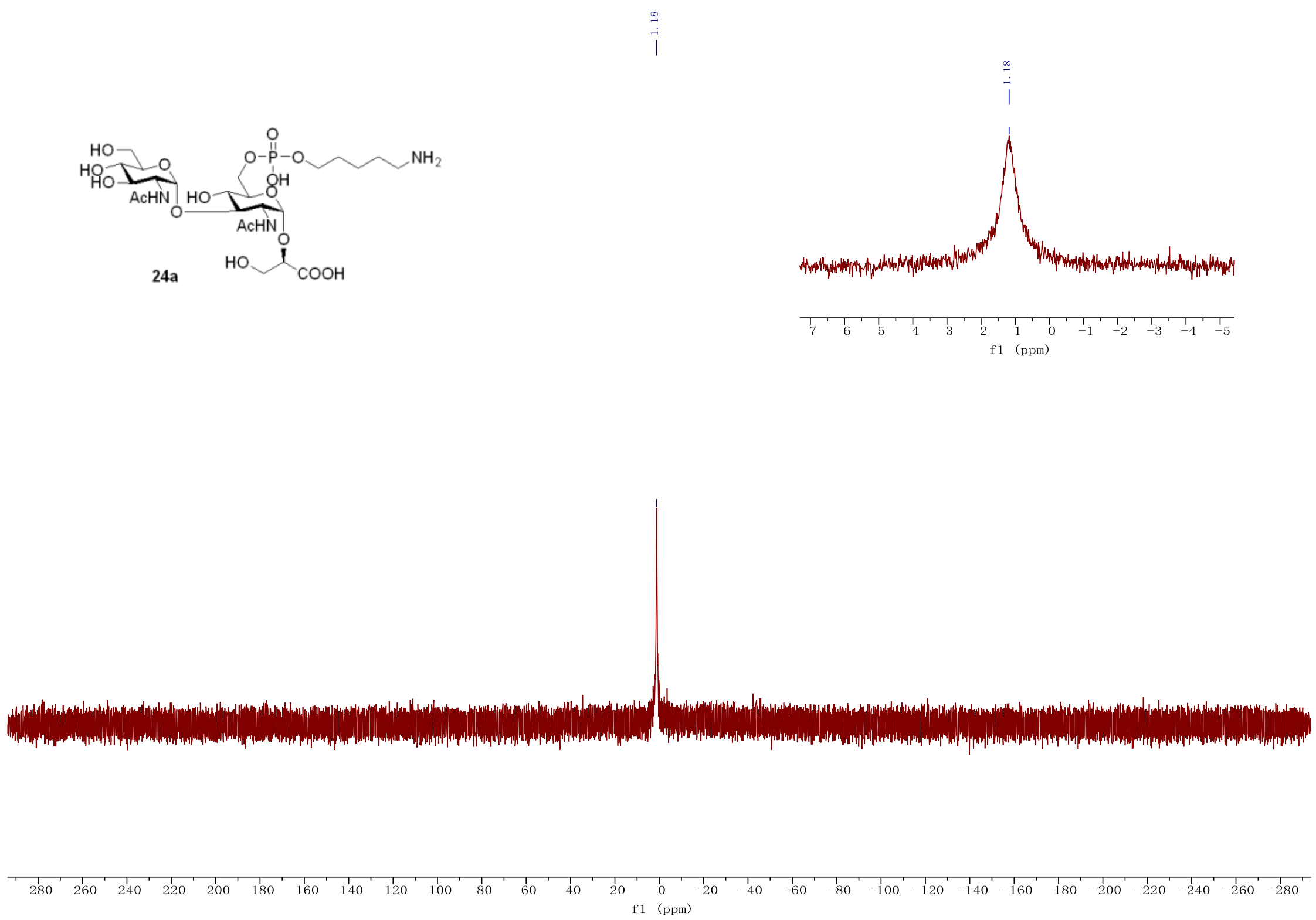

Figure S48. Compound **24a** ^{31}P NMR (D_2O, 203 MHz)

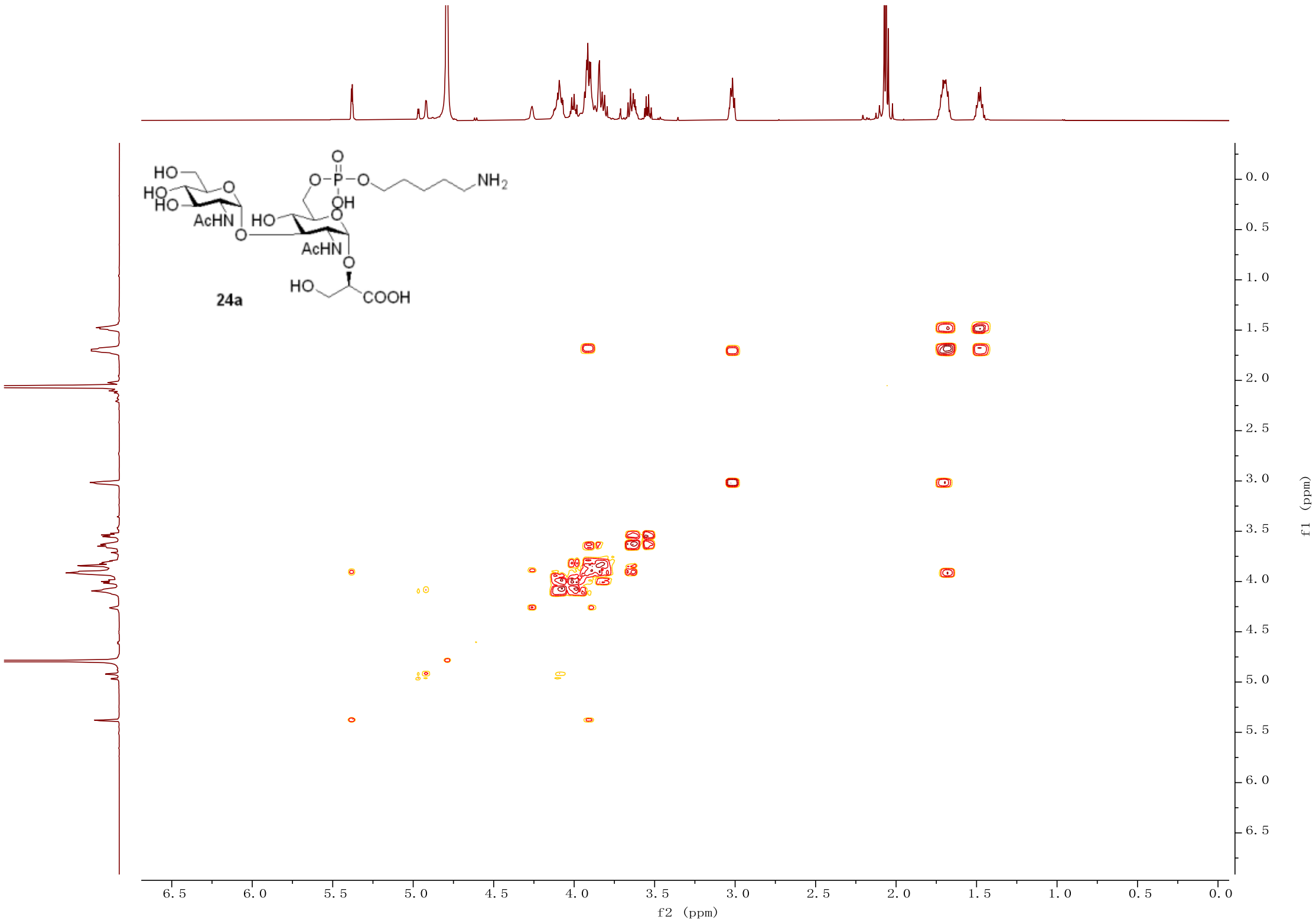

Figure S49. Compound **24a** COSY NMR (D_2O, 800 MHz)

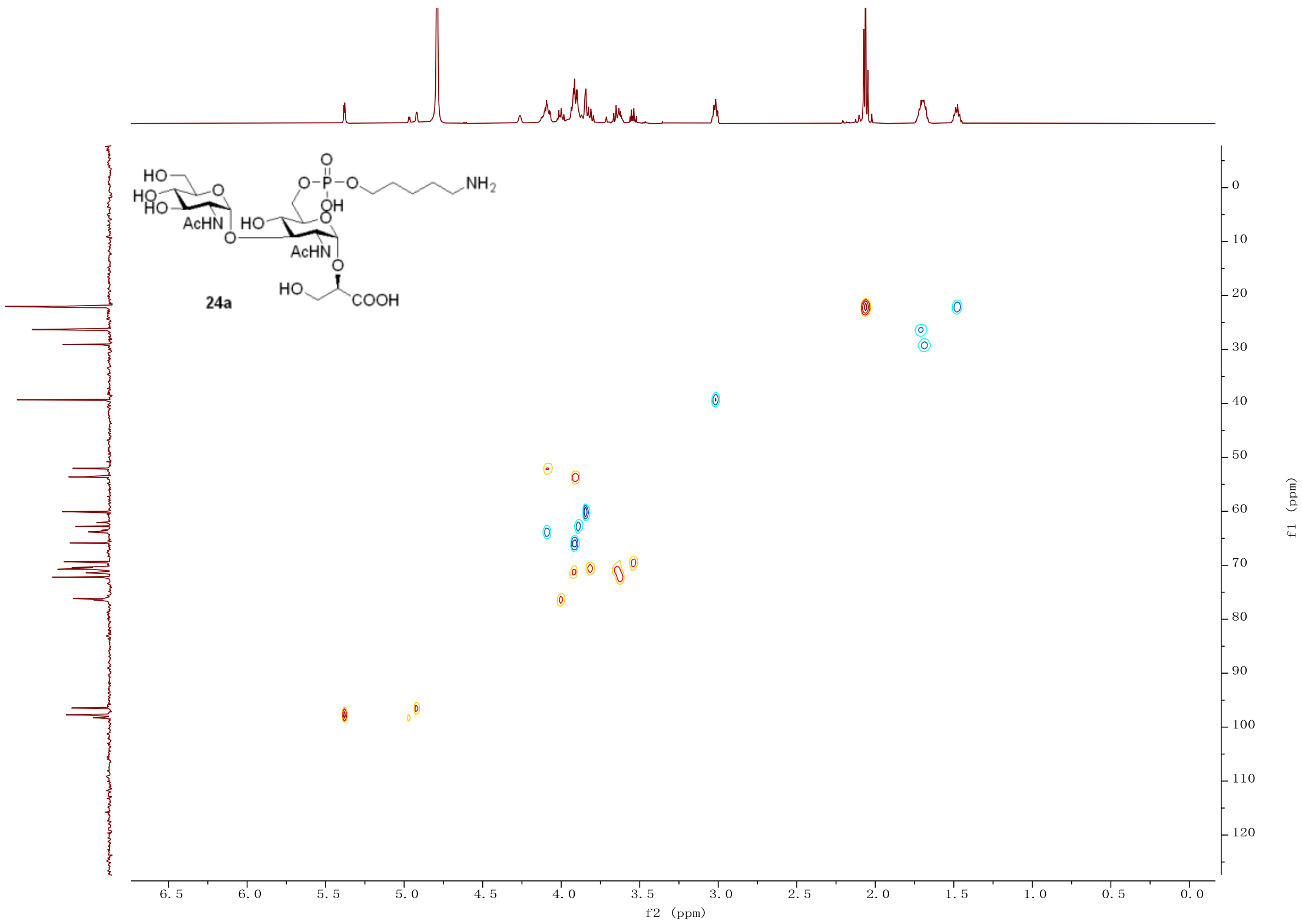

Figure S50. Compound **24a** HSQC NMR (D_2O, 800 MHz)

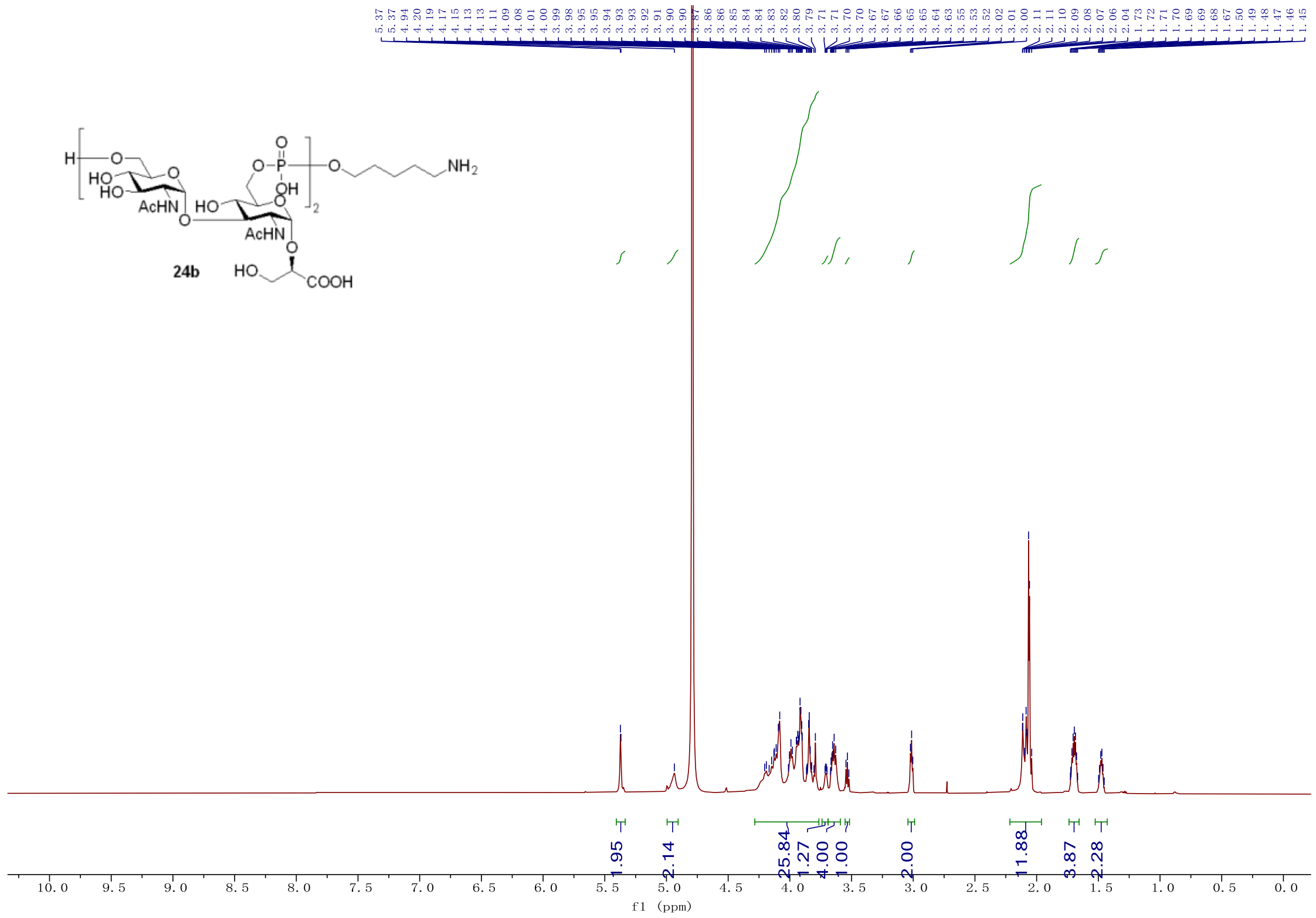

Figure S51. Compound **24b** ^{1}H NMR (D_2O, 800 MHz)

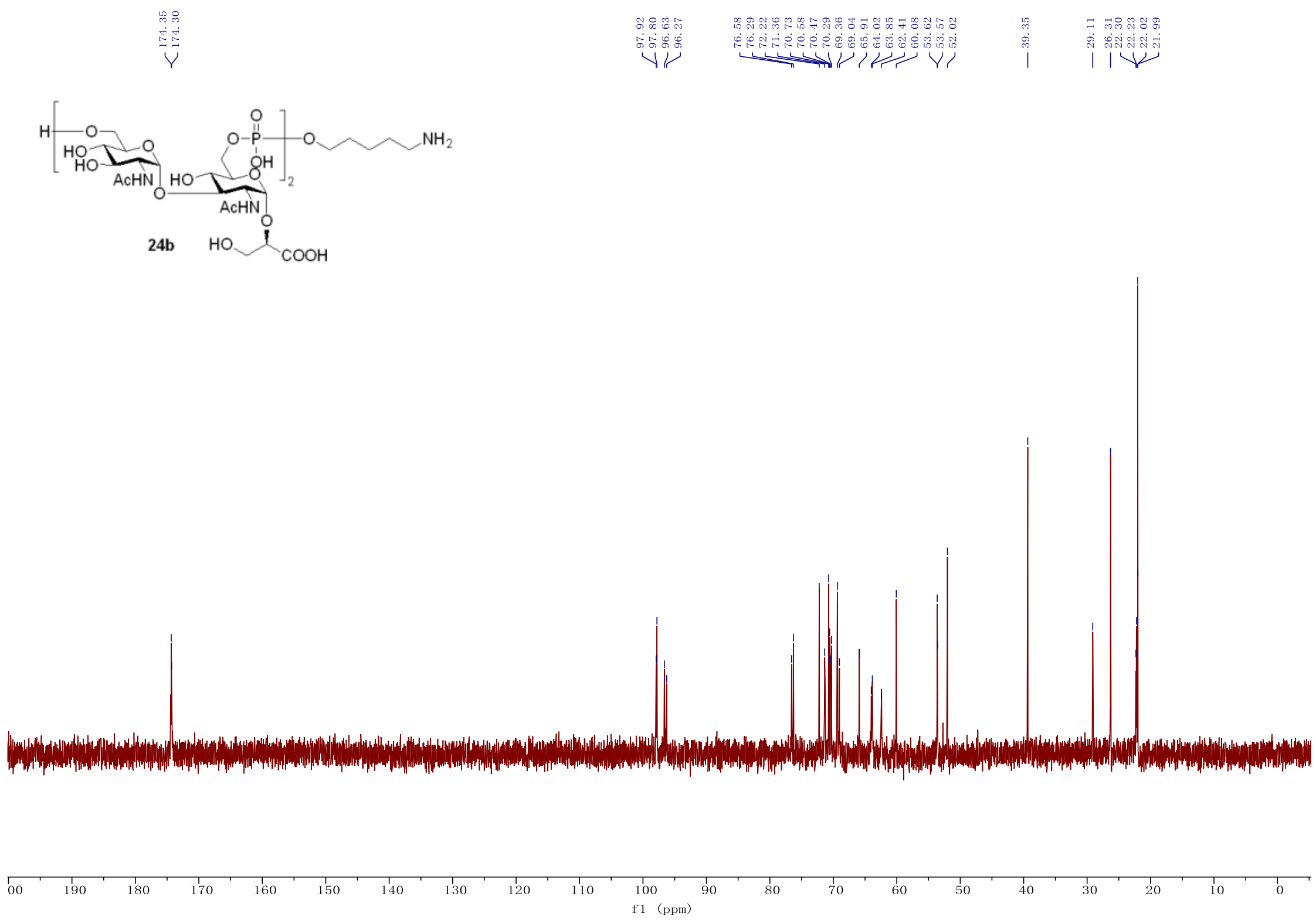

Figure S52. Compound **24b** ^{13}C NMR (D_2O, 201 MHz)

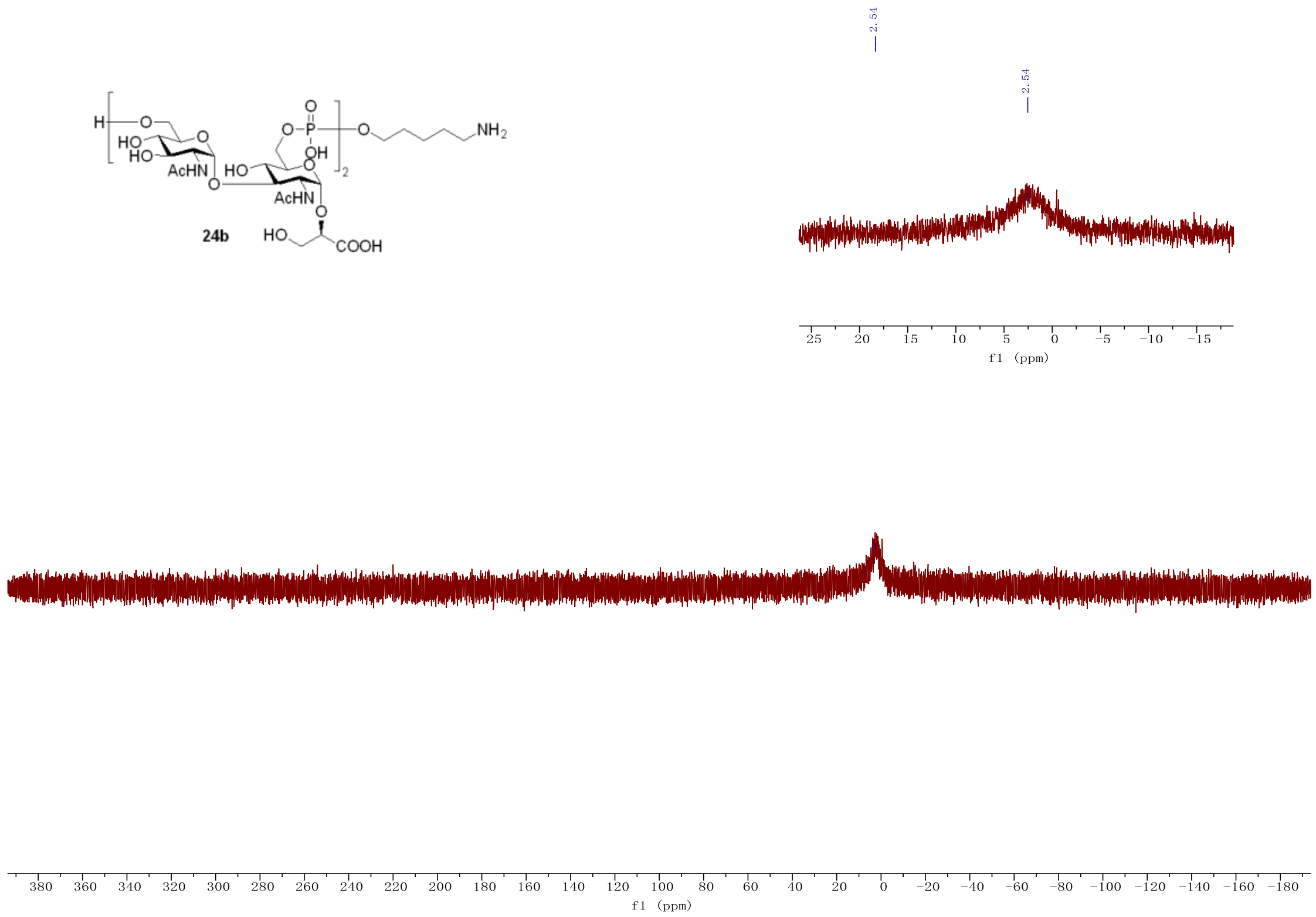

Figure S53. Compound **24b** ^{31}P NMR (D_2O, 203 MHz)

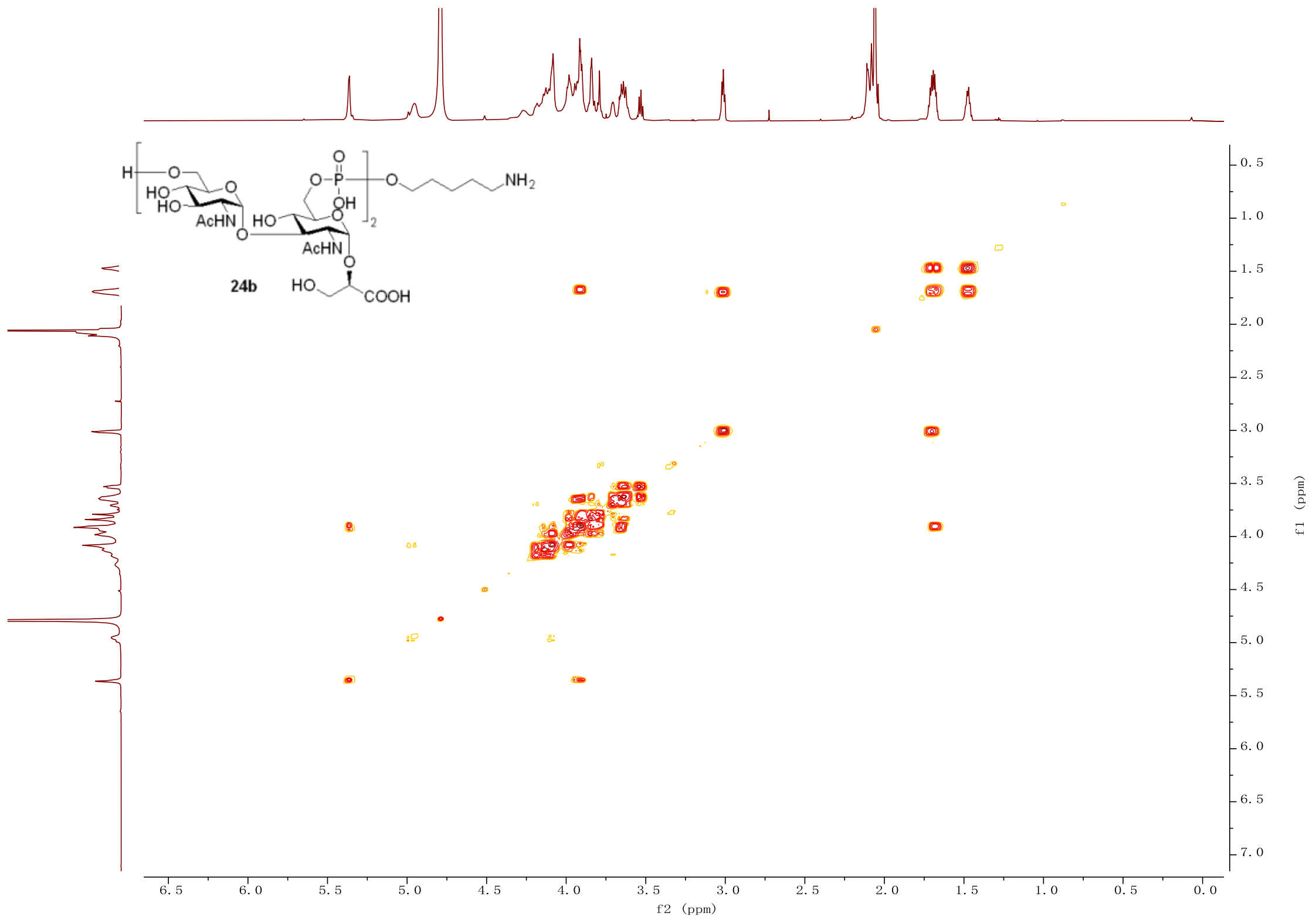

Figure S54. Compound **24b** COSY NMR (D_2O, 800 MHz)

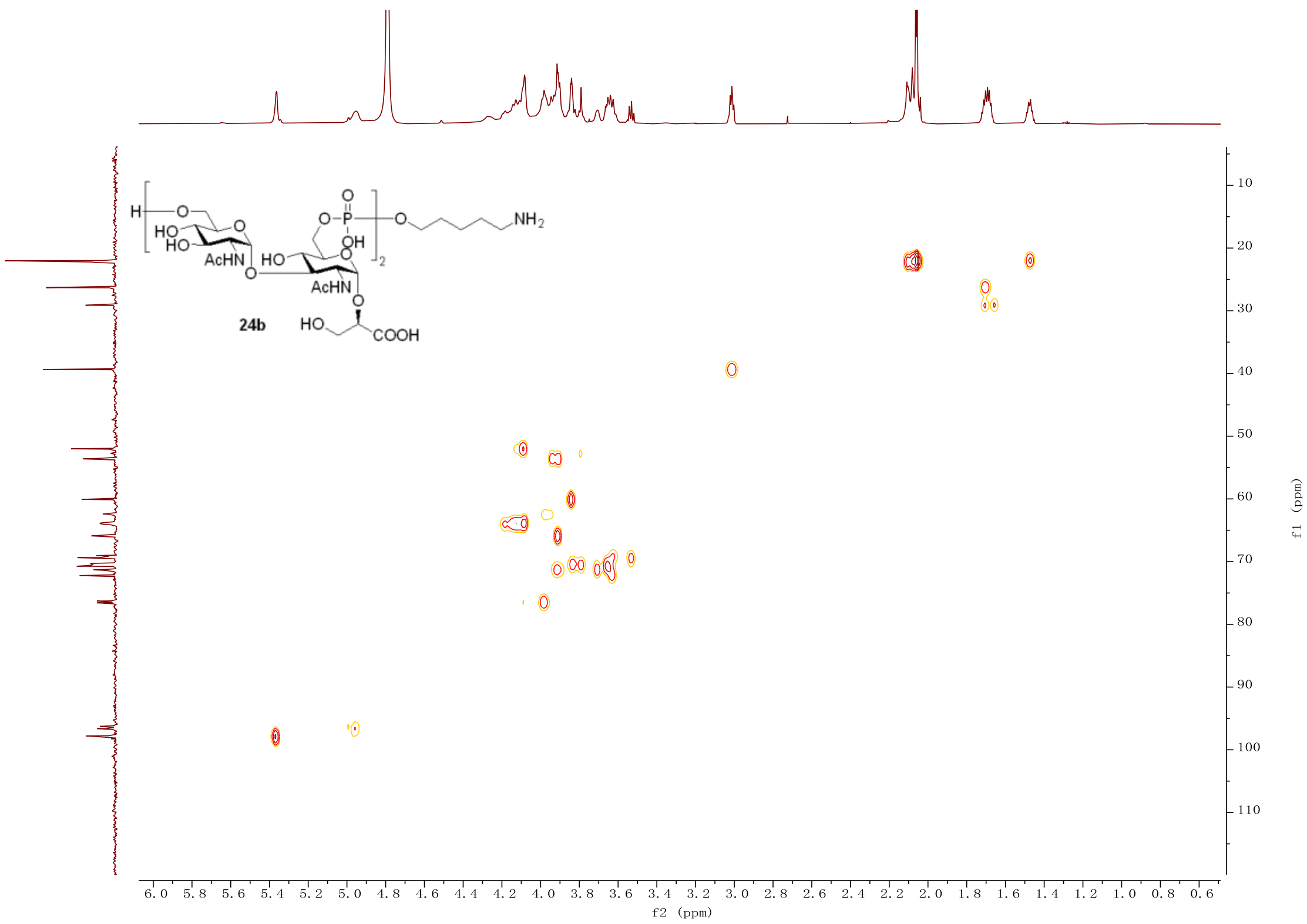

Figure S55. Compound **24b** HSQC NMR (D_2O, 800 MHz)

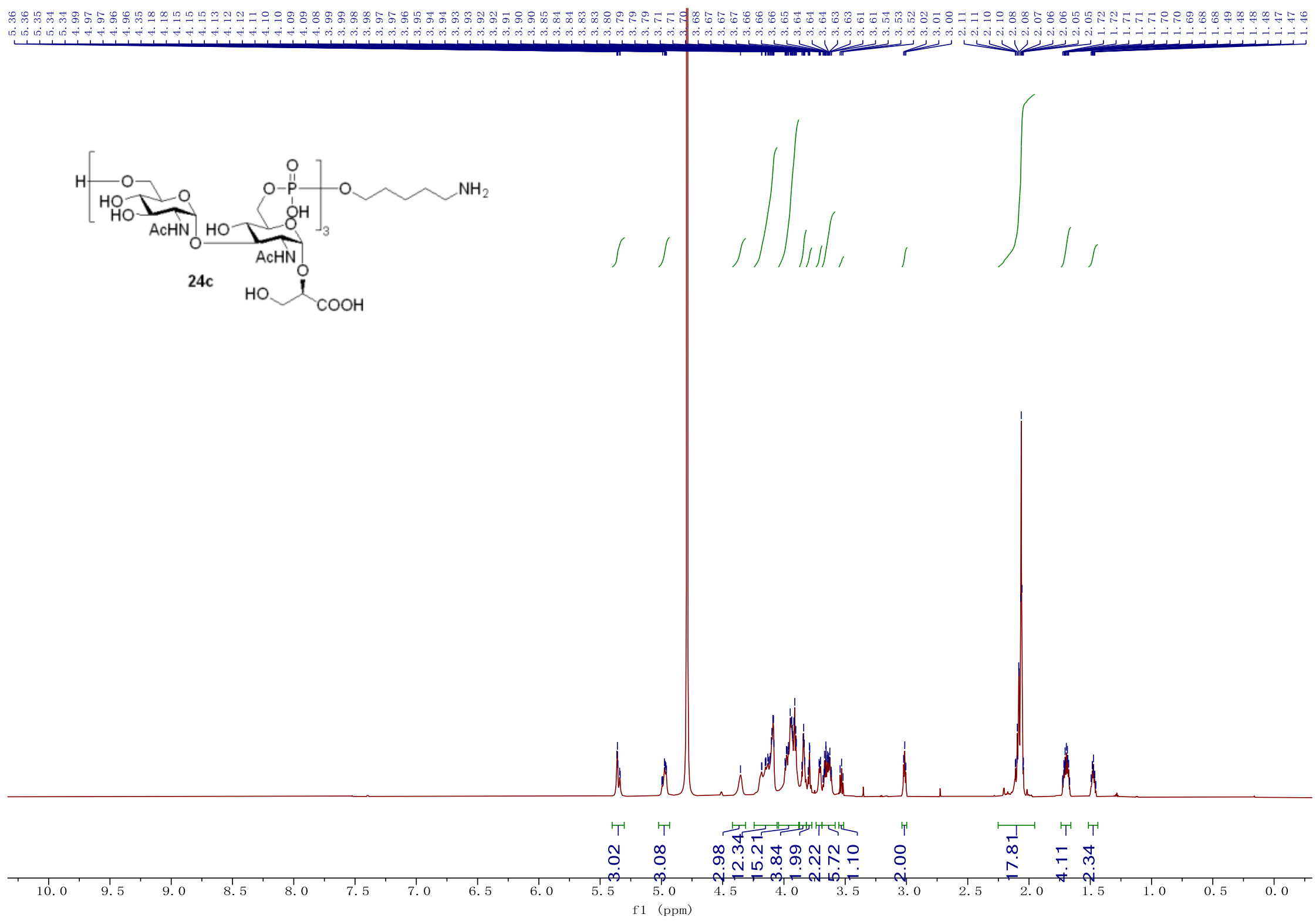

Figure S56. Compound **24c** ^{1}H NMR (D_2O, 800 MHz)

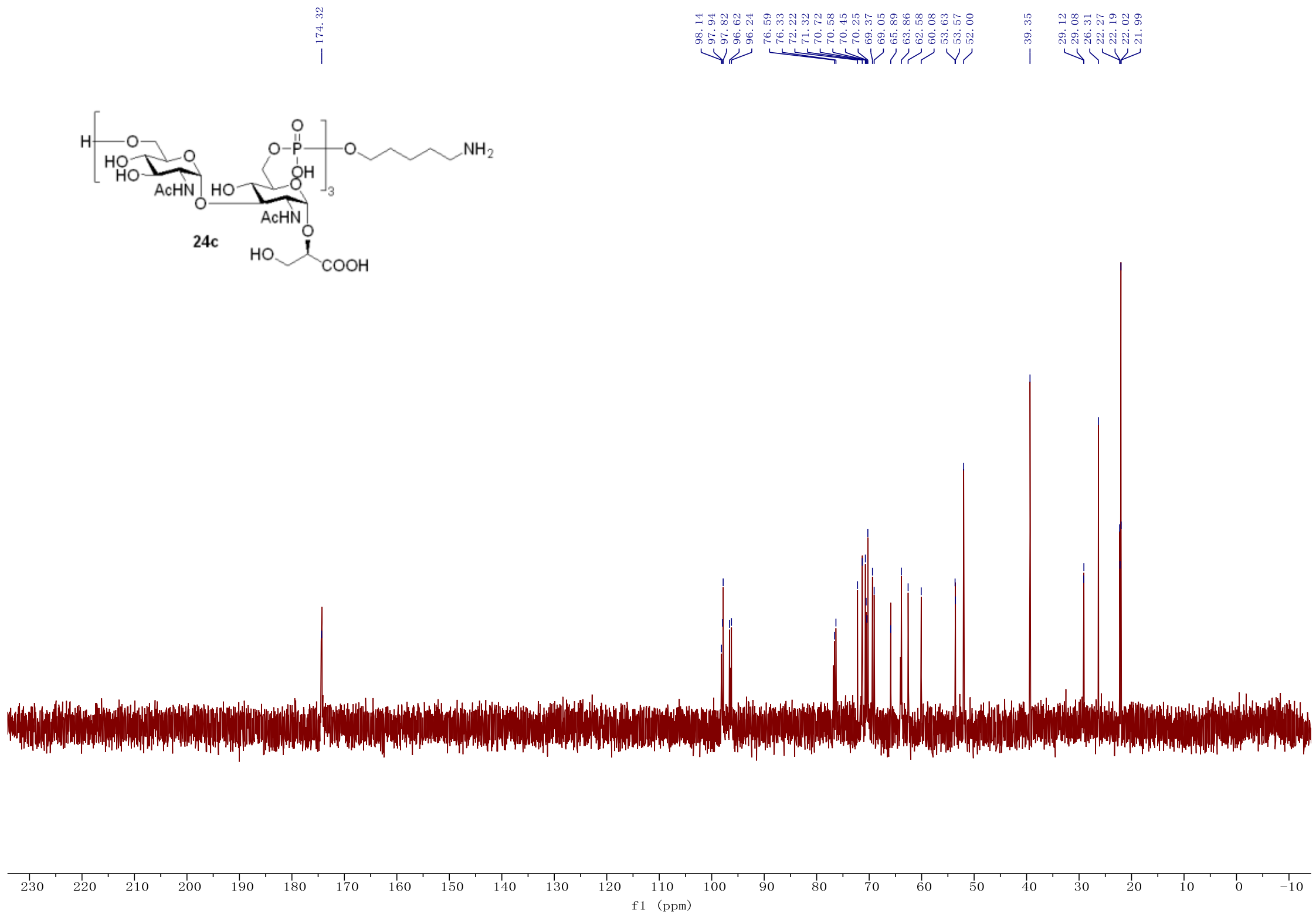

Figure S57. Compound **24c** ^{13}C NMR (D_2O, 201 MHz)

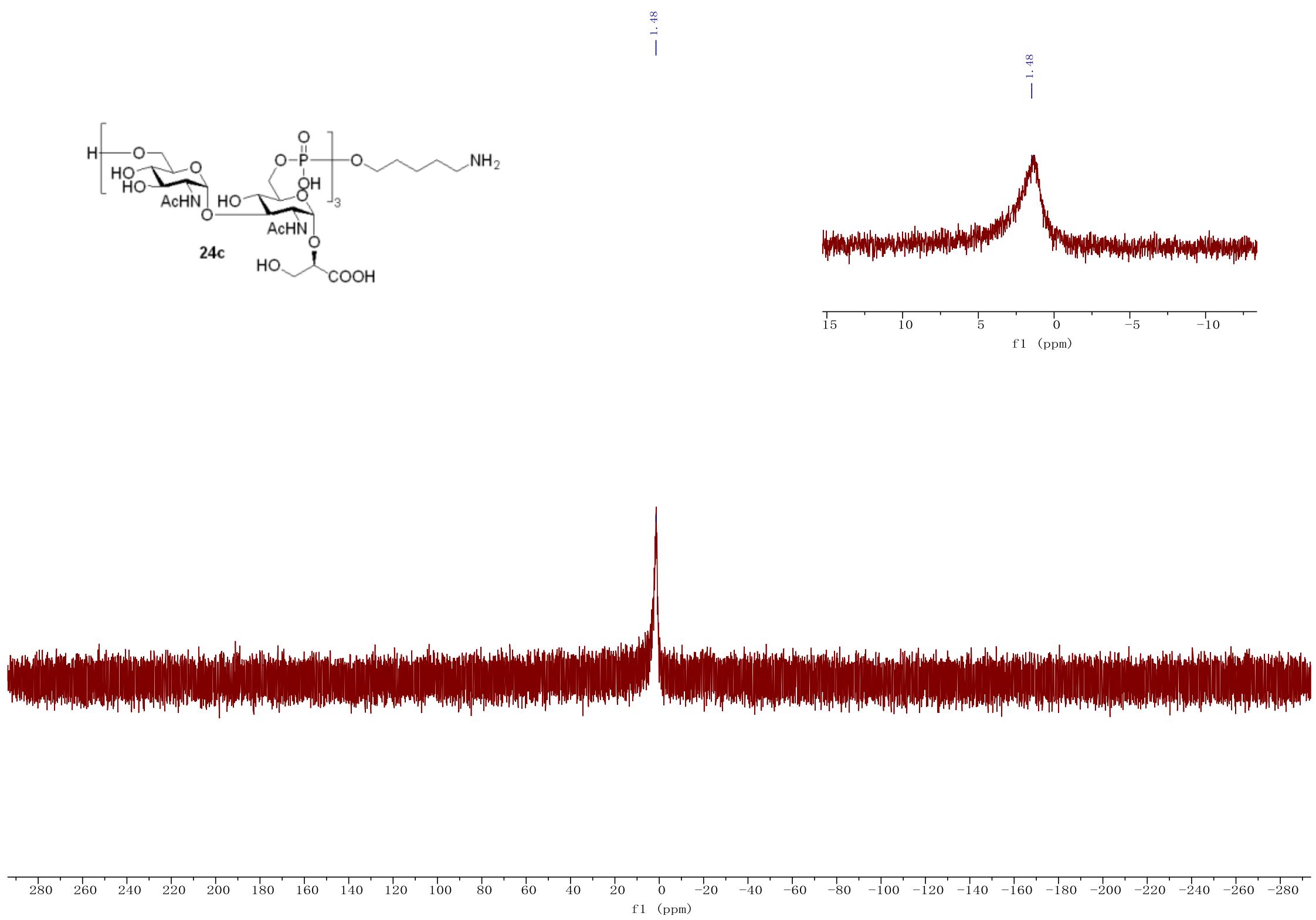

Figure S58. Compound **24c** ^{31}P NMR (D_2O, 203 MHz)

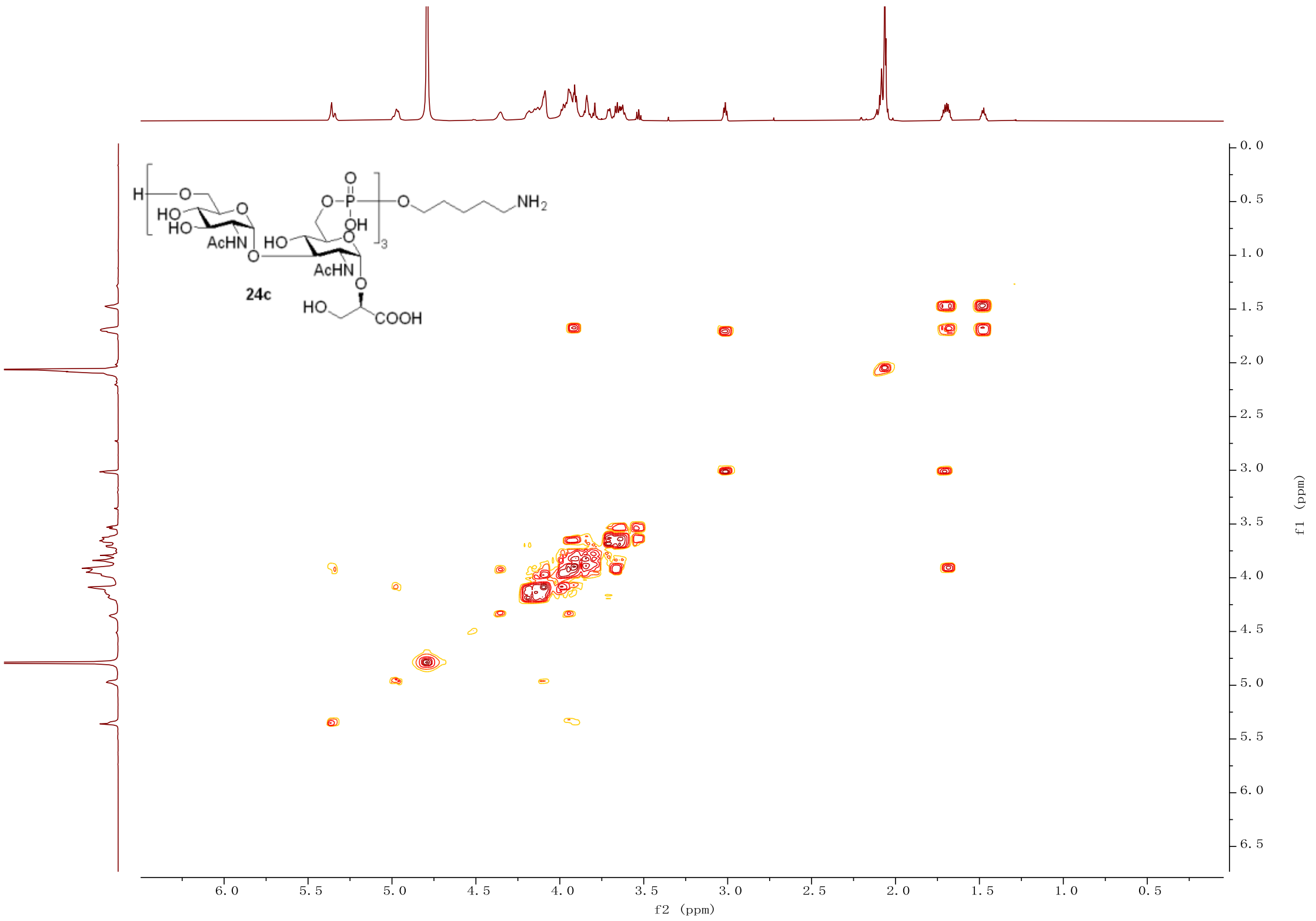

Figure S59. Compound **24c** COSY NMR (D_2O, 800 MHz)

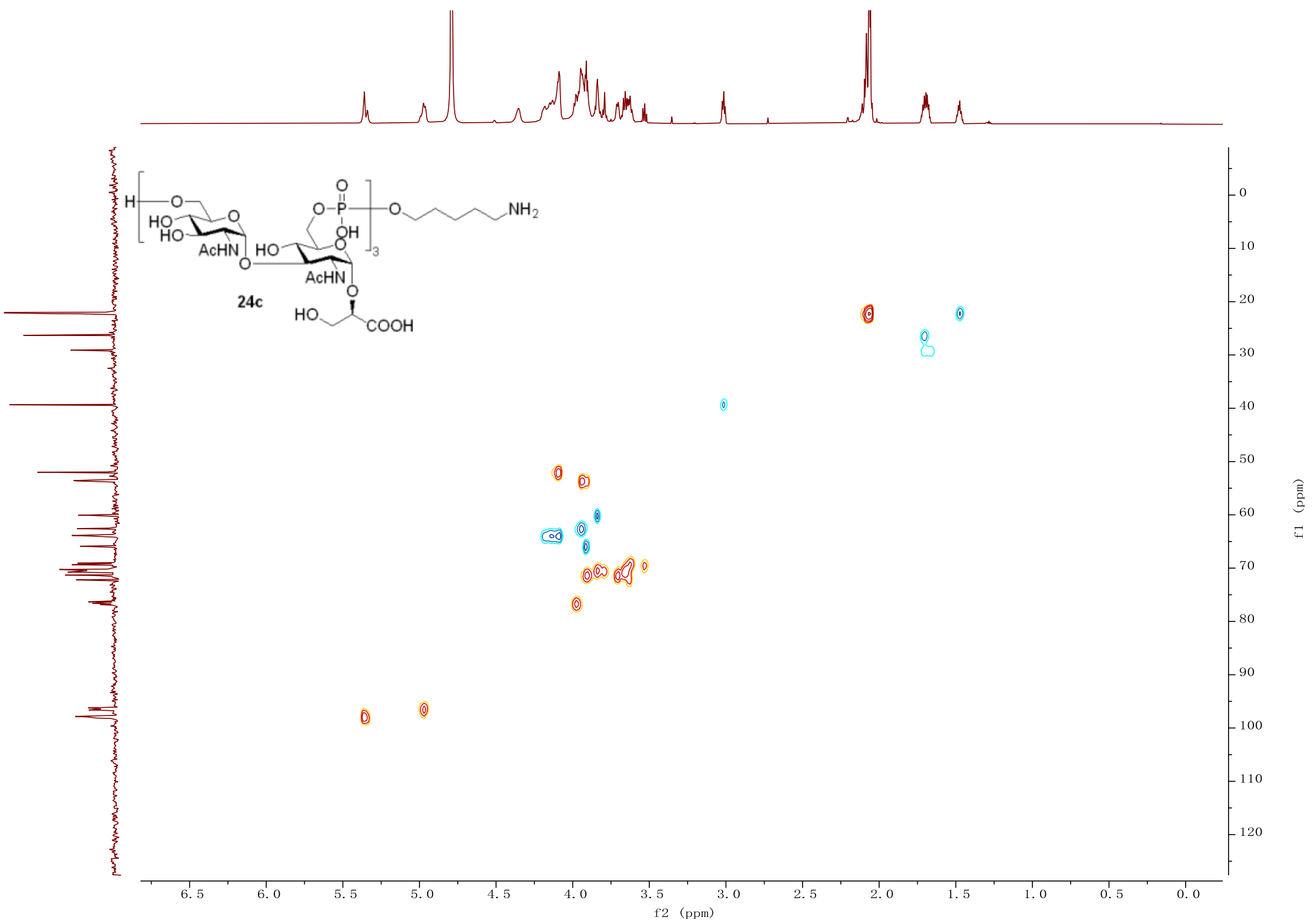

Figure S60. Compound **24c** HSQC NMR (D_2O, 800 MHz)

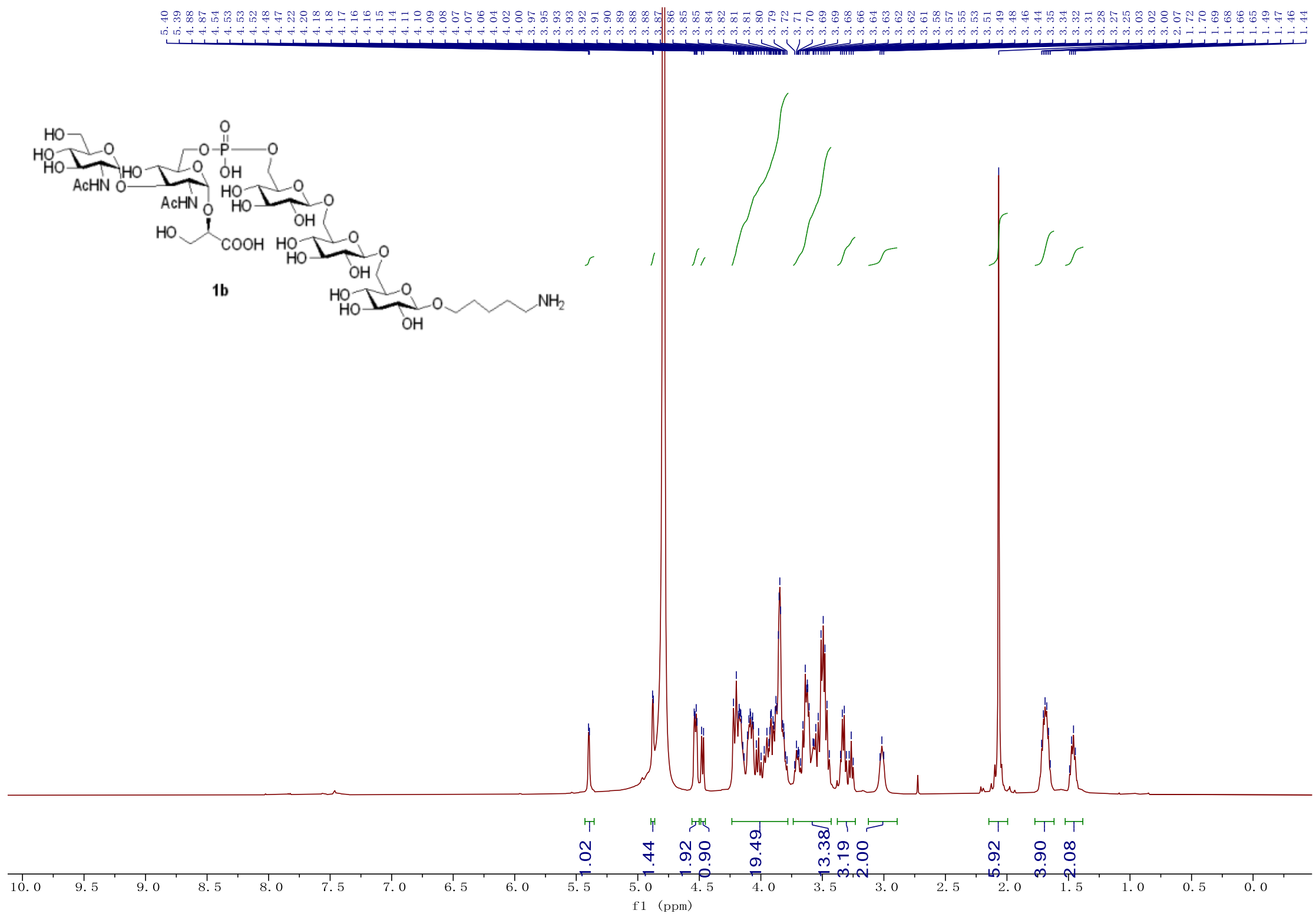

Figure S61. Compound **1b** ^{1}H NMR (D_2O, 500 MHz)

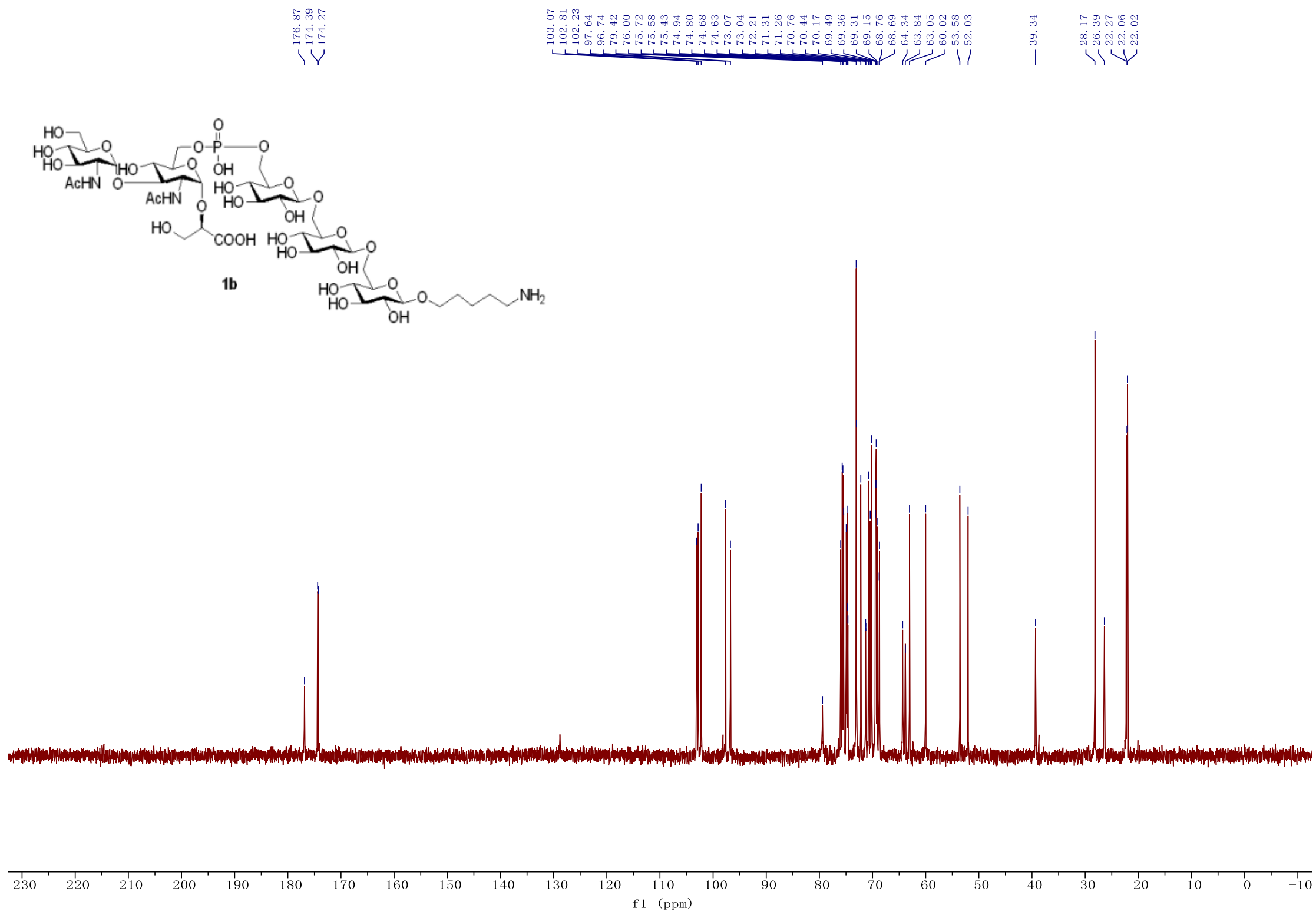

Figure S62. Compound **1b** ^{13}C NMR (D_2O, 151 MHz)

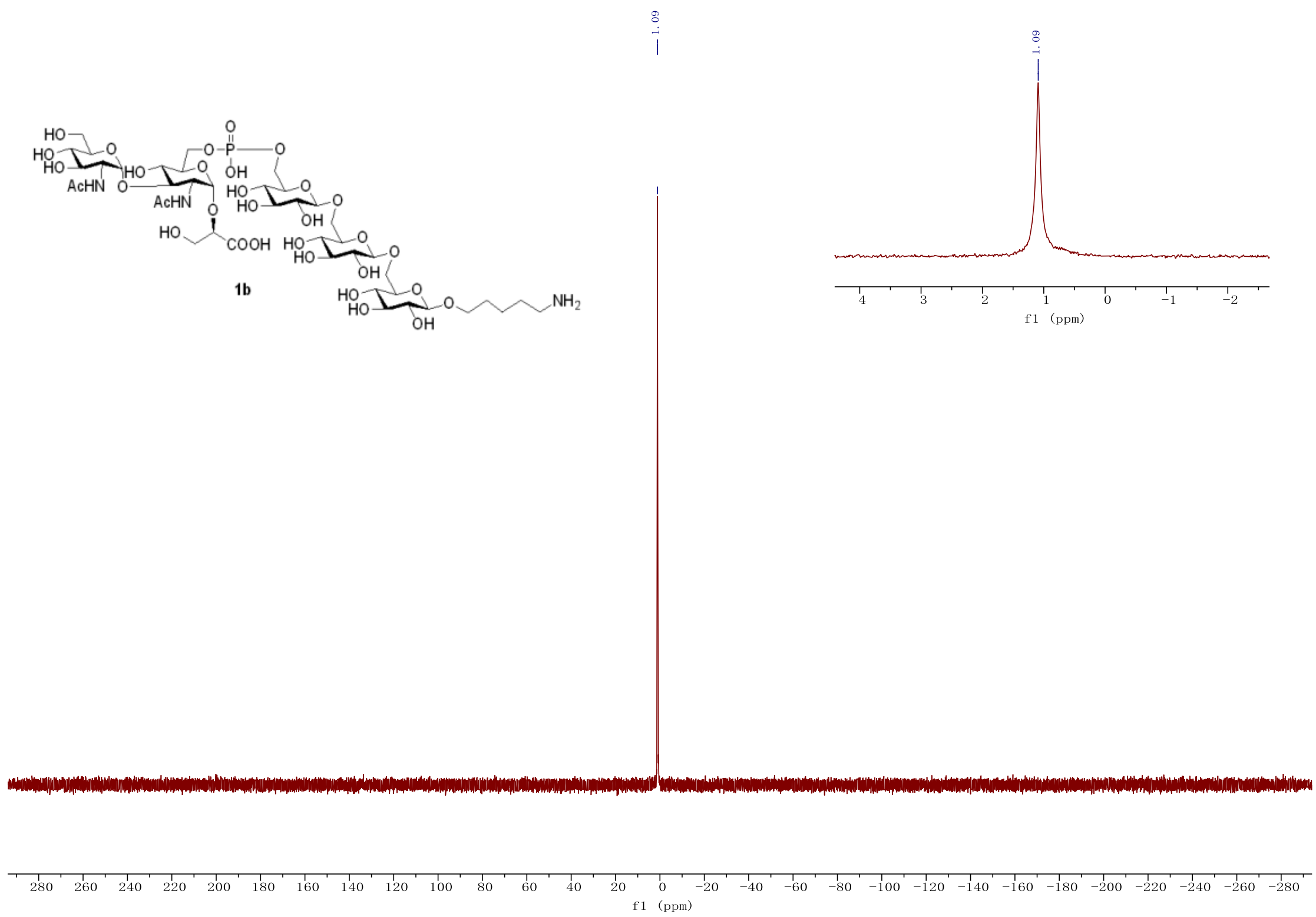

Figure S63. Compound **1b** ^{31}P NMR (D_2O, 202 MHz)

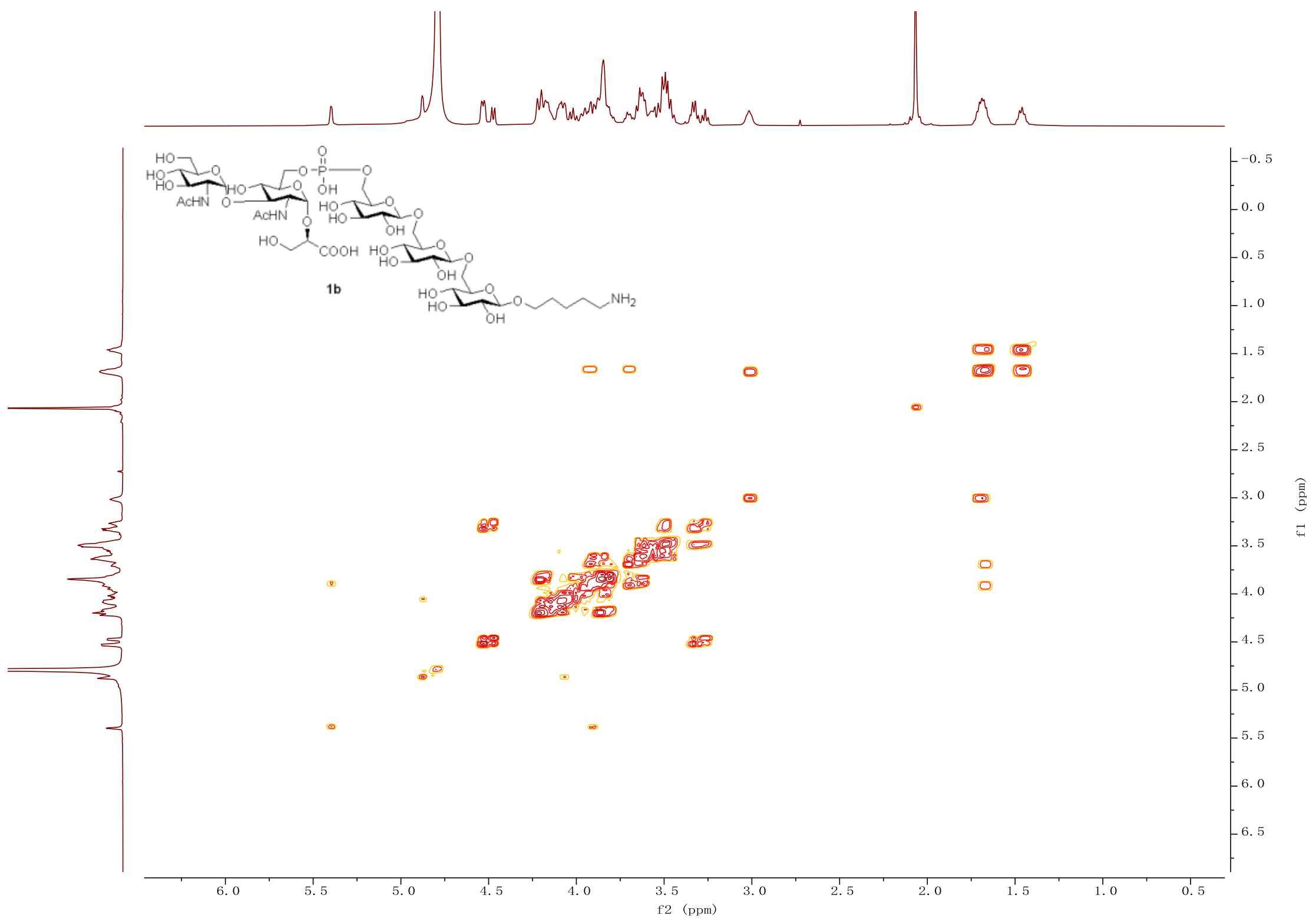

Figure S64. Compound **1b** COSY NMR (D_2O, 600 MHz)

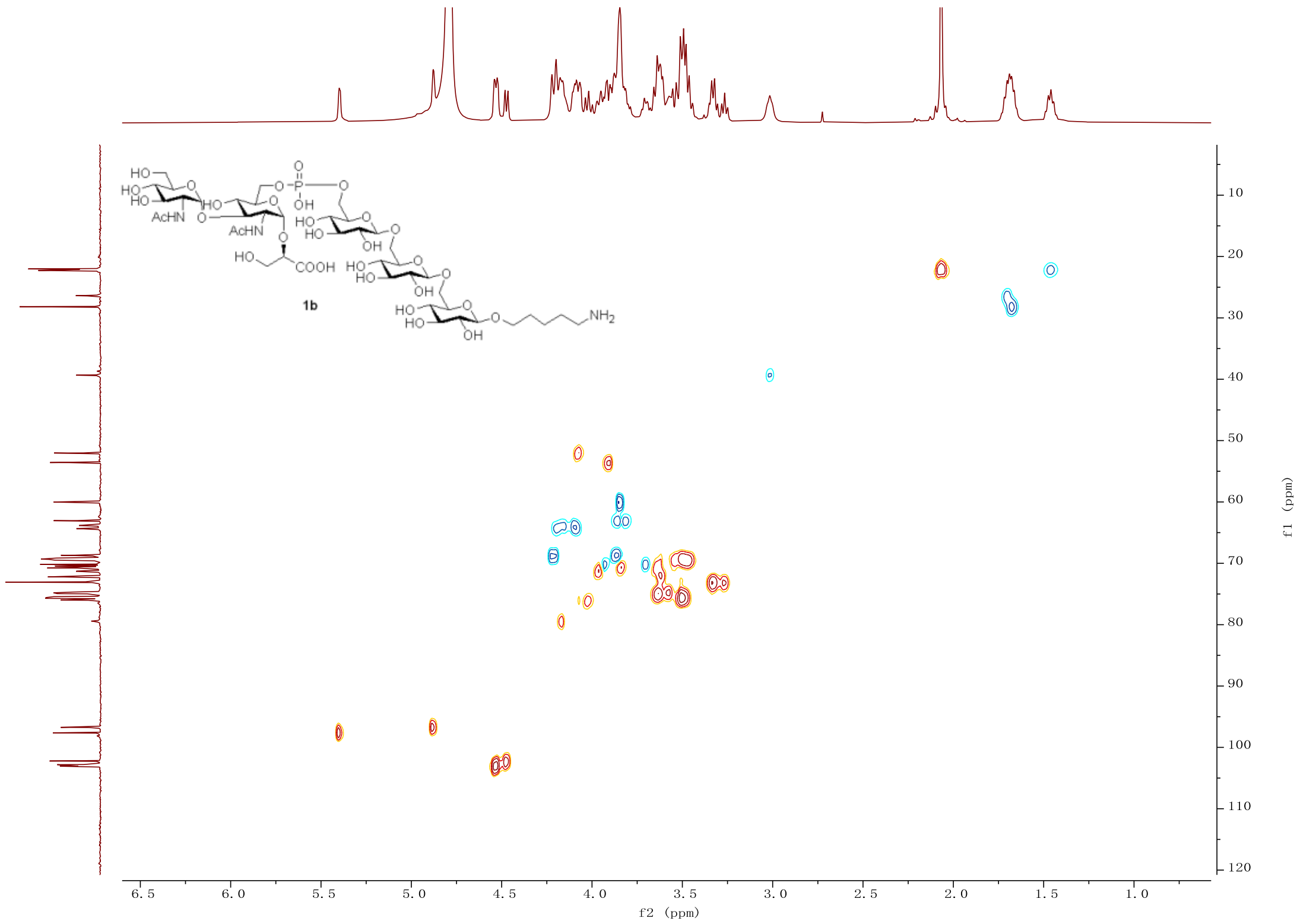

Figure S65. Compound **1b** HSQC NMR (D_2O, 600 MHz)

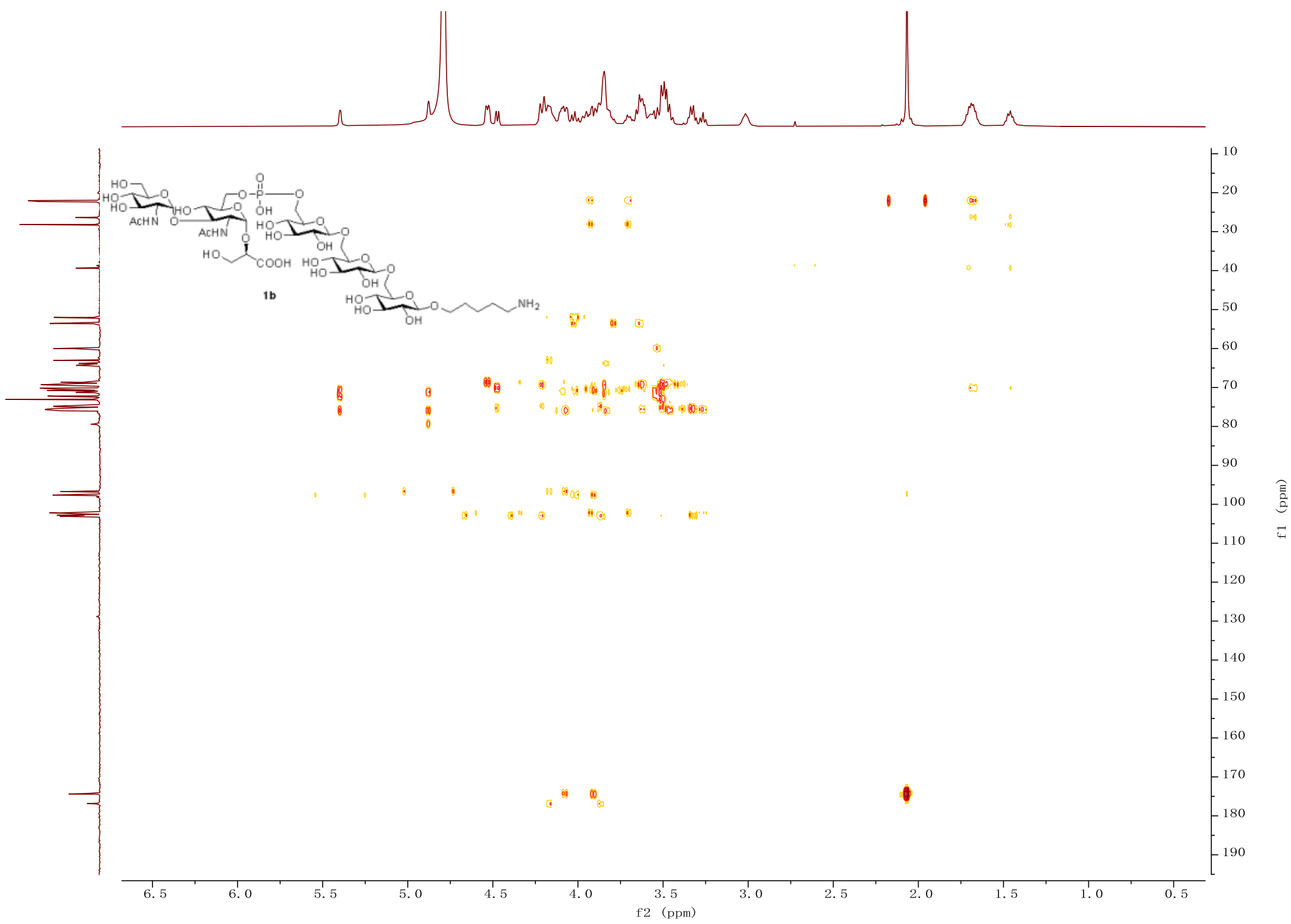

Figure S66. Compound **1b** HMBC NMR (D_2O, 600 MHz)

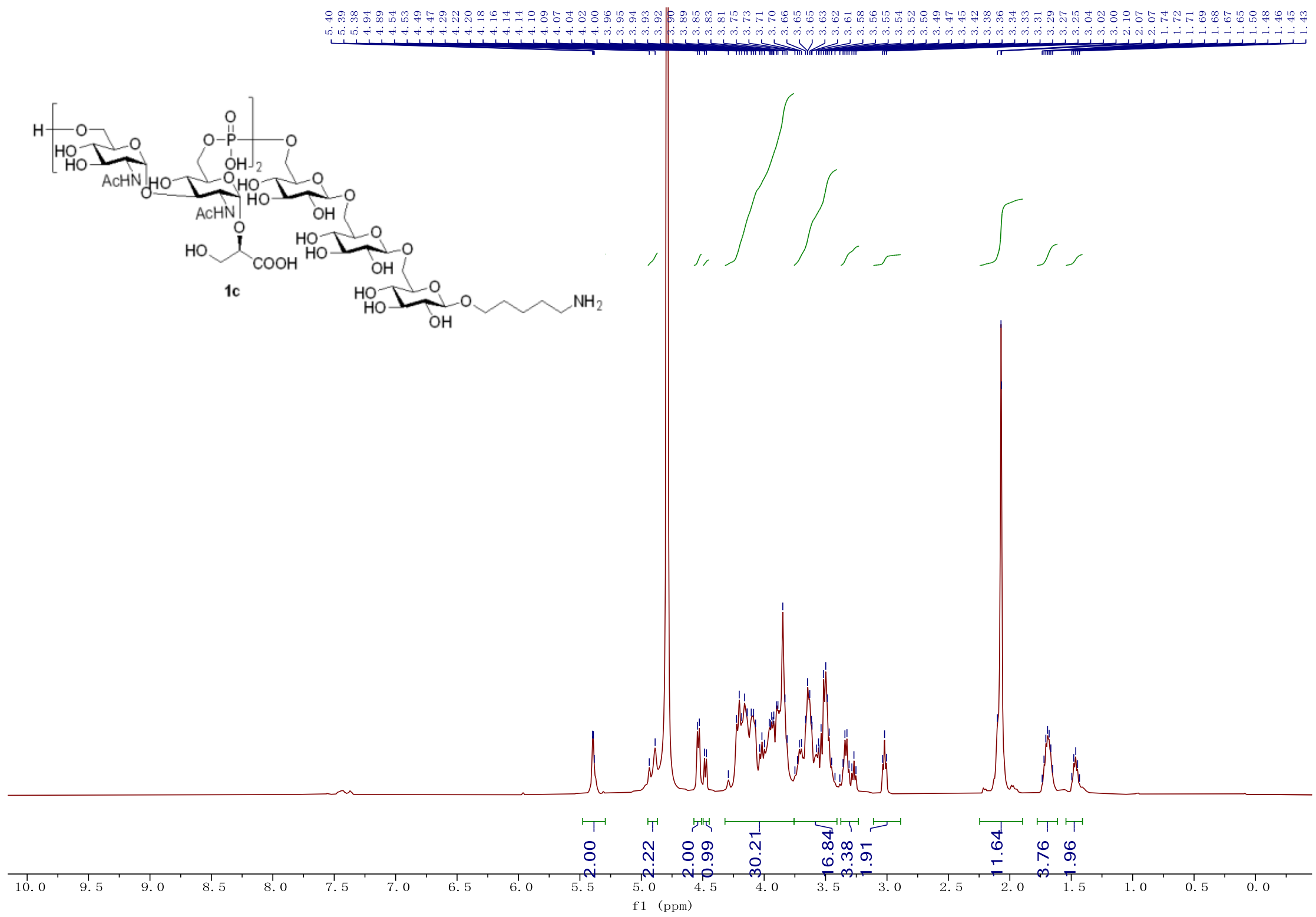

Figure S67. Compound **1c** ^{1}H NMR (D_2O, 500 MHz)

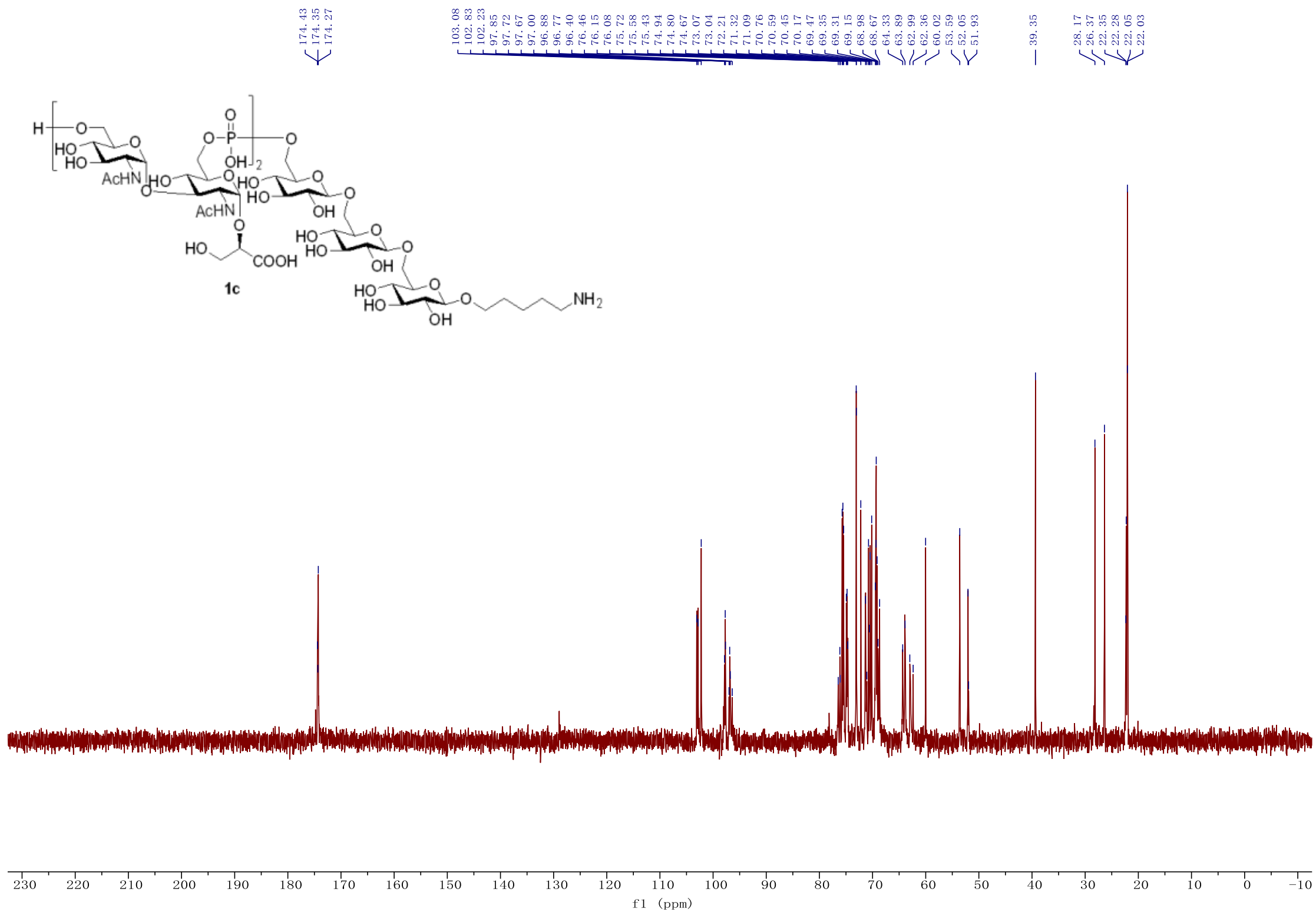

Figure S68. Compound **1c** ^{13}C NMR (D_2O, 151 MHz)

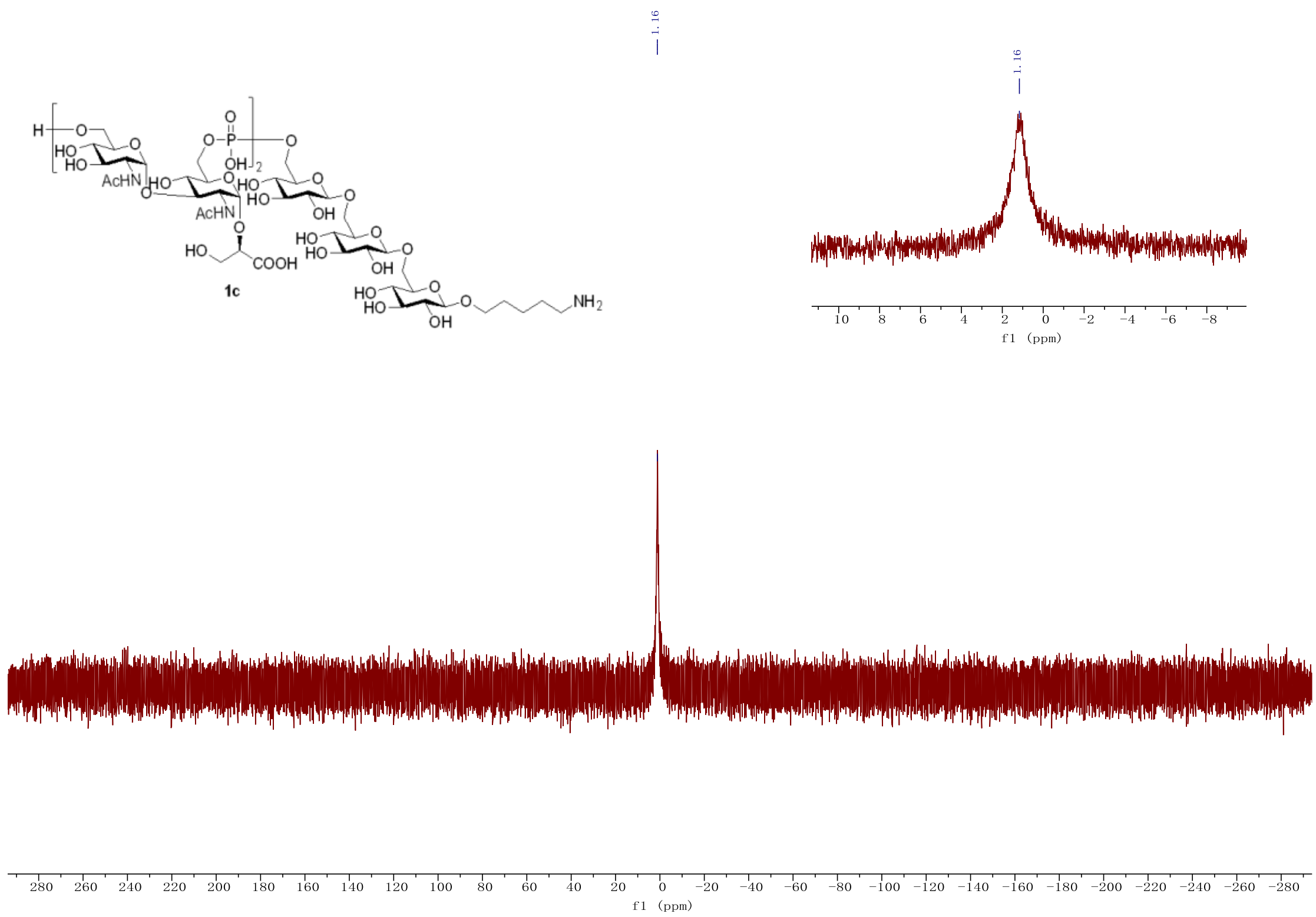

Figure S69. Compound **1c** ^{31}P NMR (D_2O, 202 MHz)

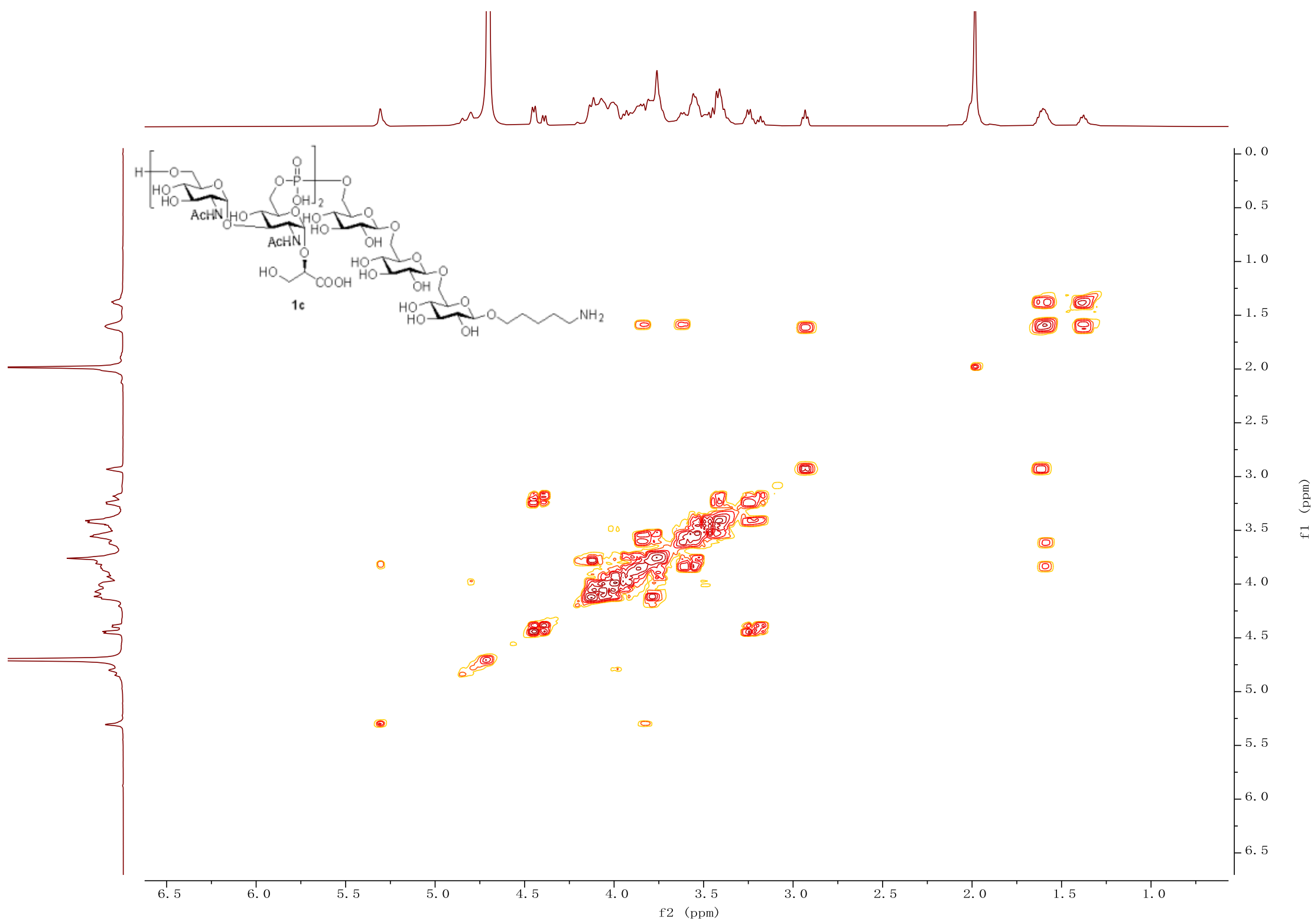

Figure S70. Compound **1c** COSY NMR (D_2O, 600 MHz)

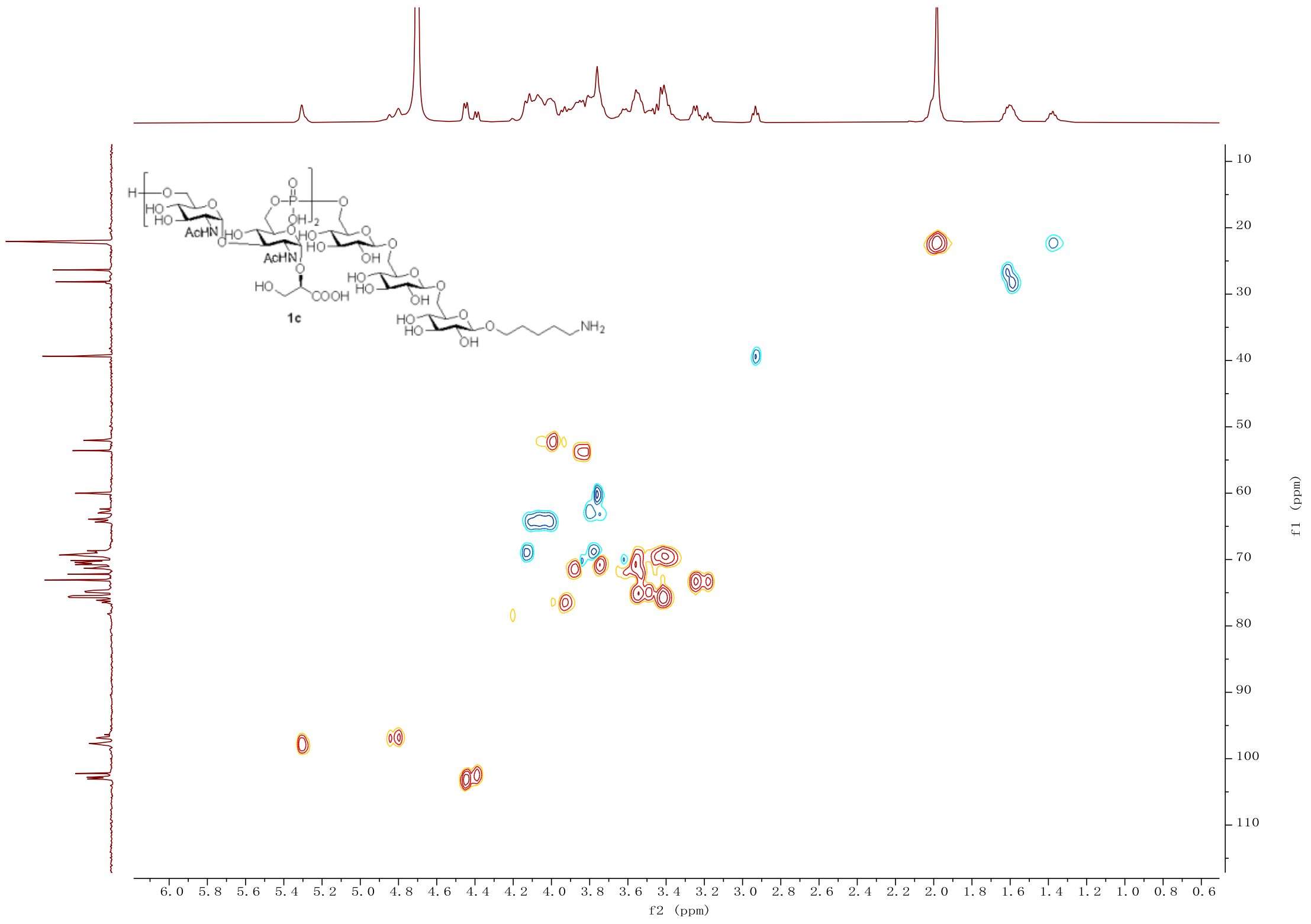

Figure S71. Compound **1c** HSQC NMR (D_2O, 600 MHz)

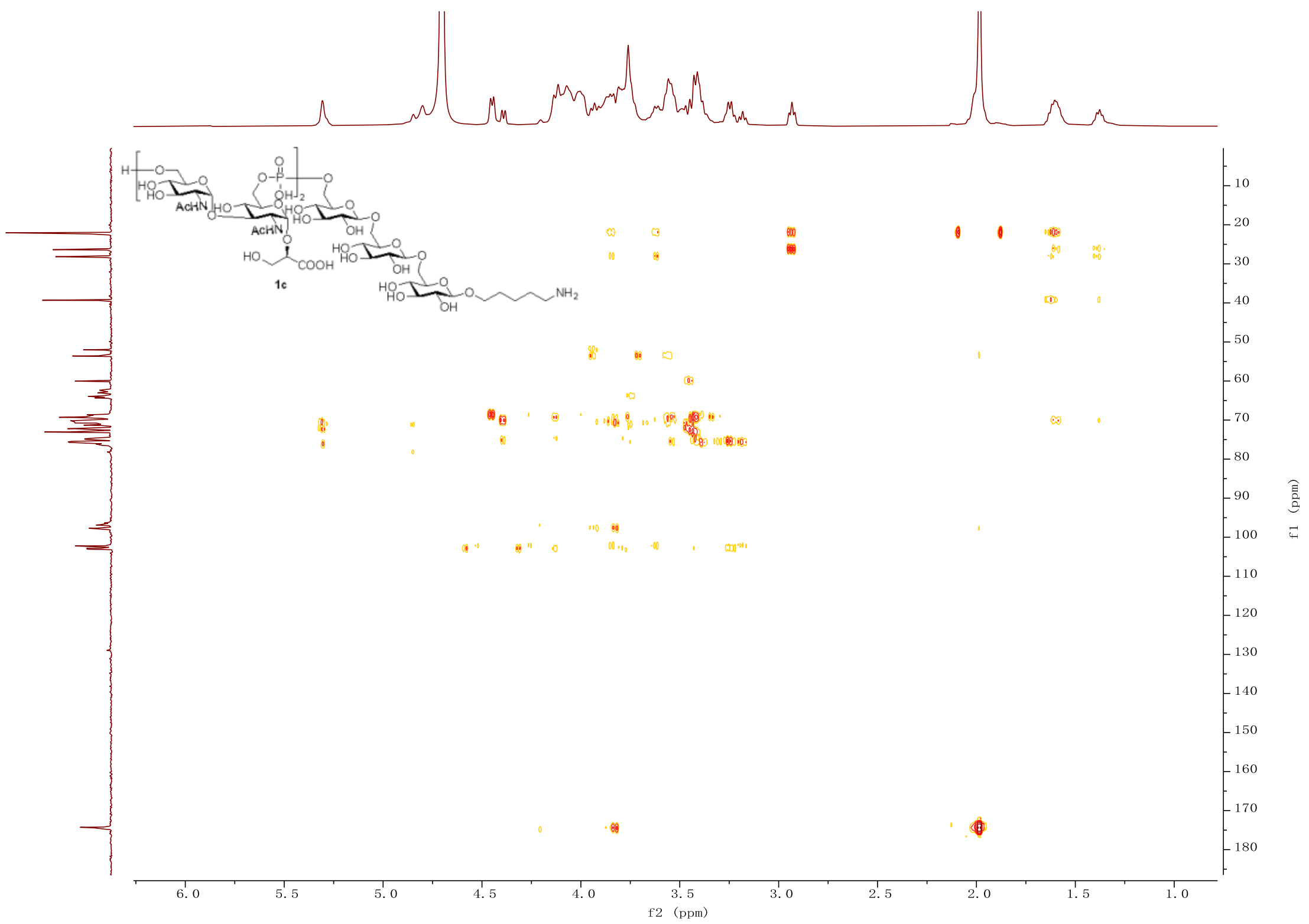

Figure S72. Compound **1c** HMBC NMR (D_2O, 600 MHz)

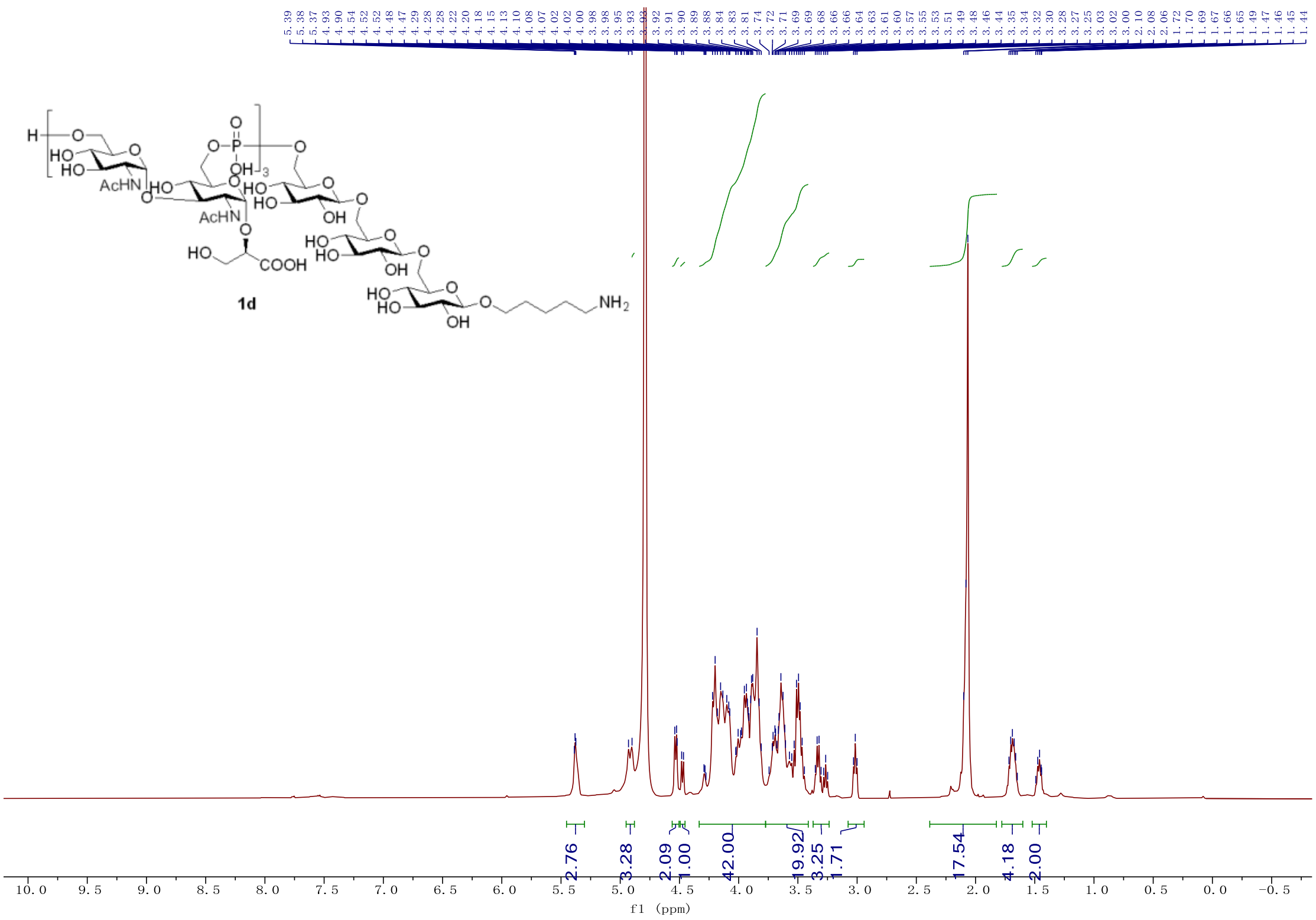

Figure S73. Compound **1d** ^{1}H NMR (D_2O, 500 MHz)

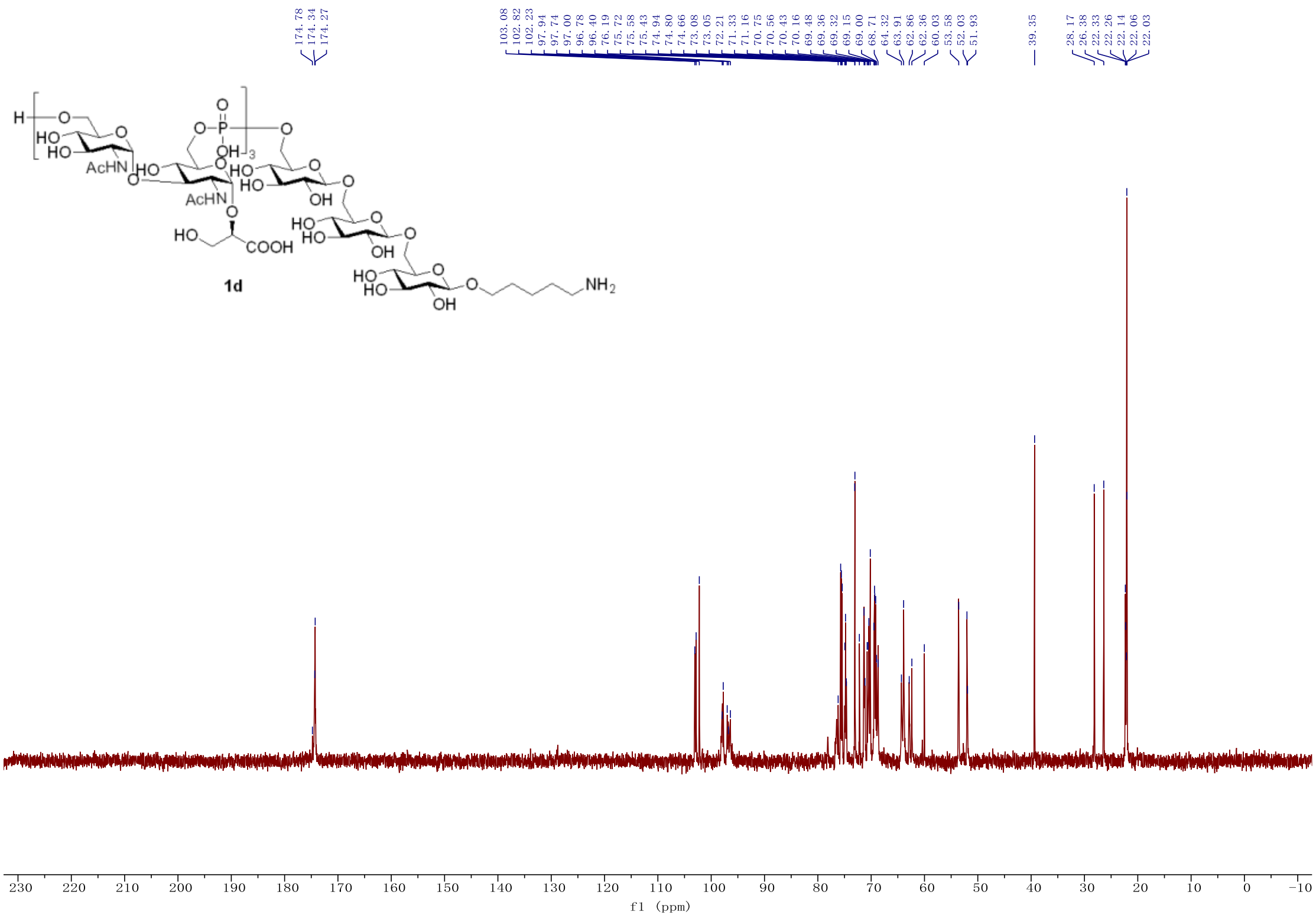

Figure S74. Compound **1d** ^{13}C NMR (D_2O, 151 MHz)

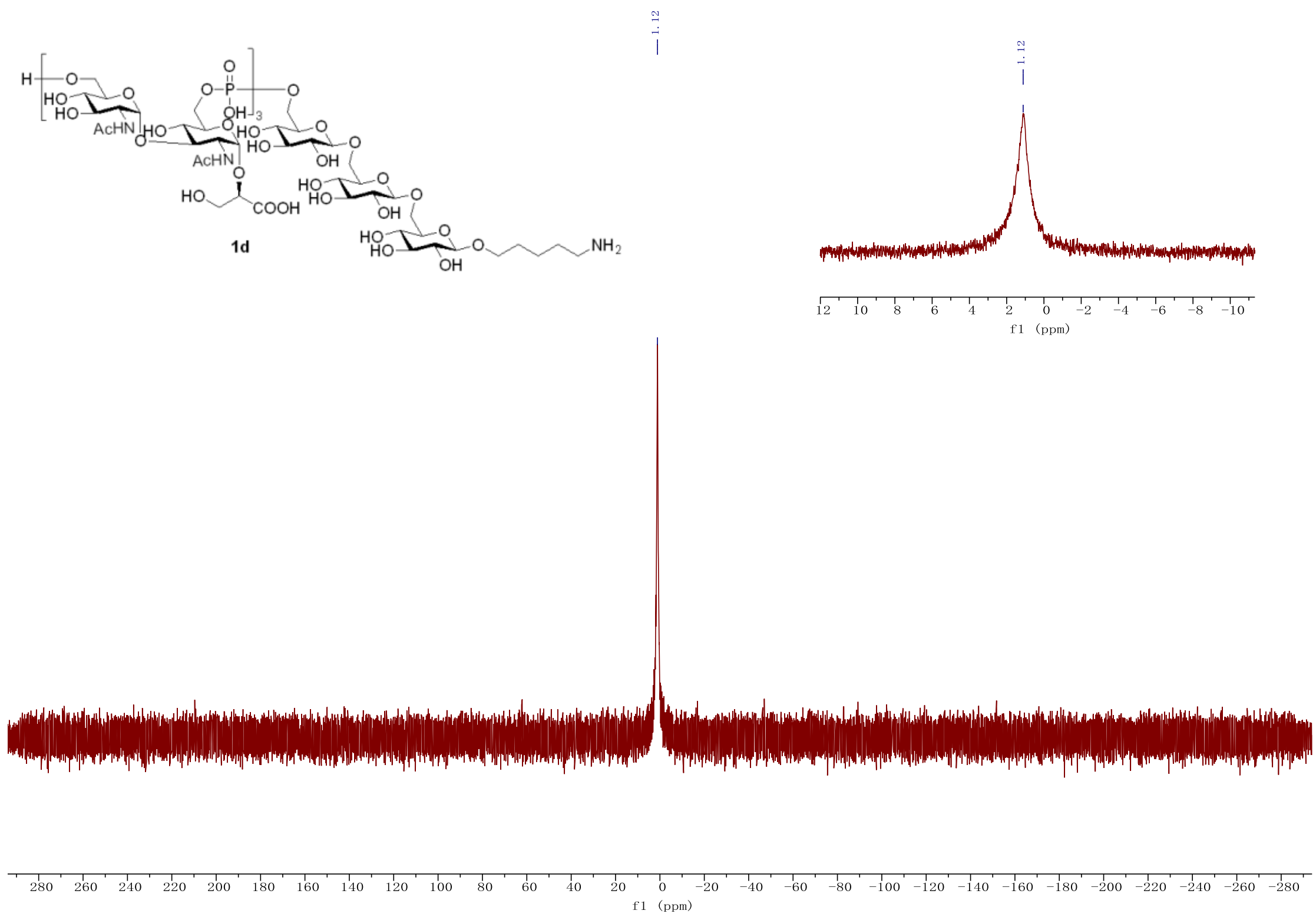

Figure S75. Compound **1d** ^{31}P NMR (D_2O, 202 MHz)

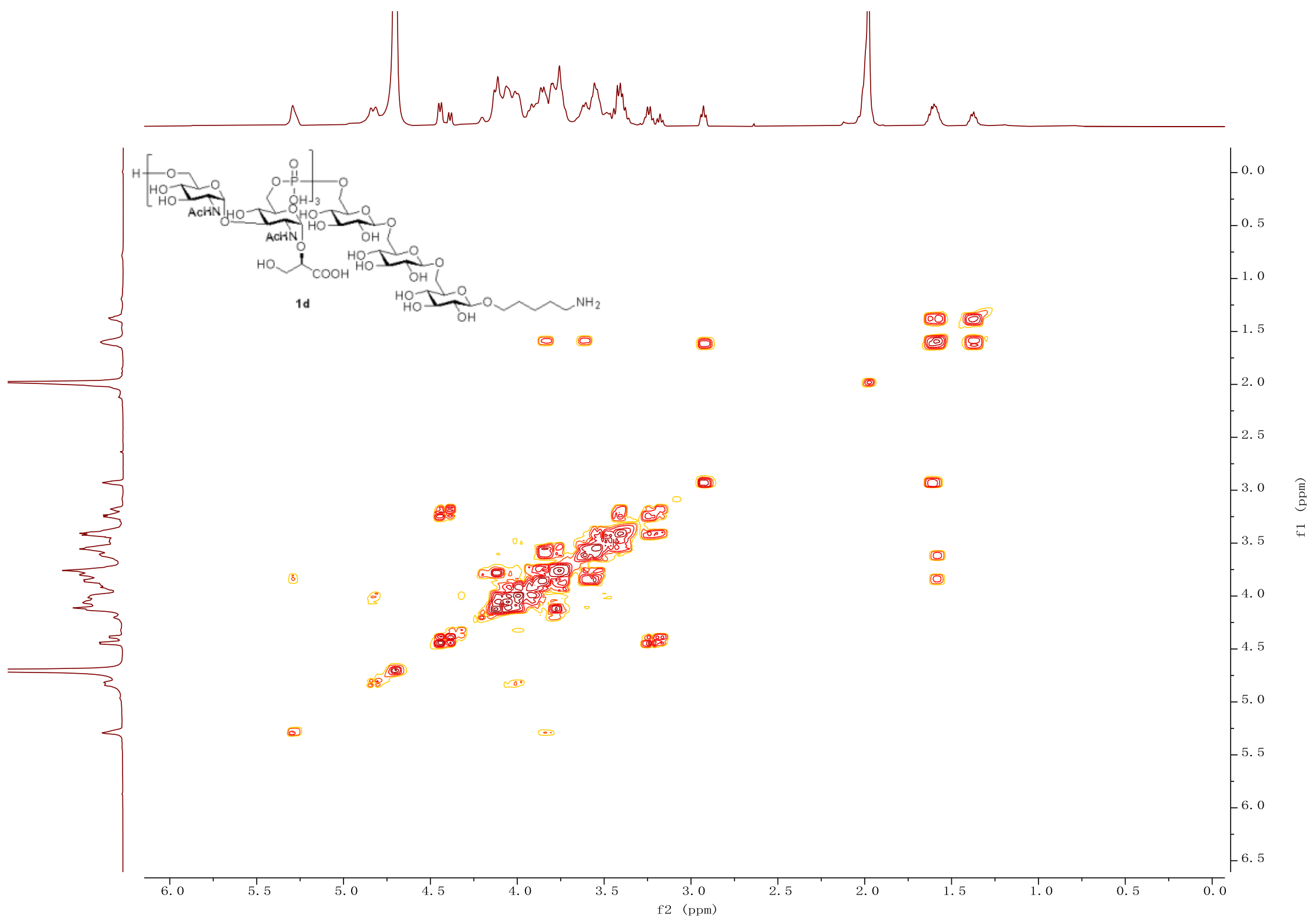

Figure S76. Compound **1d** COSY NMR (D_2O, 600 MHz)

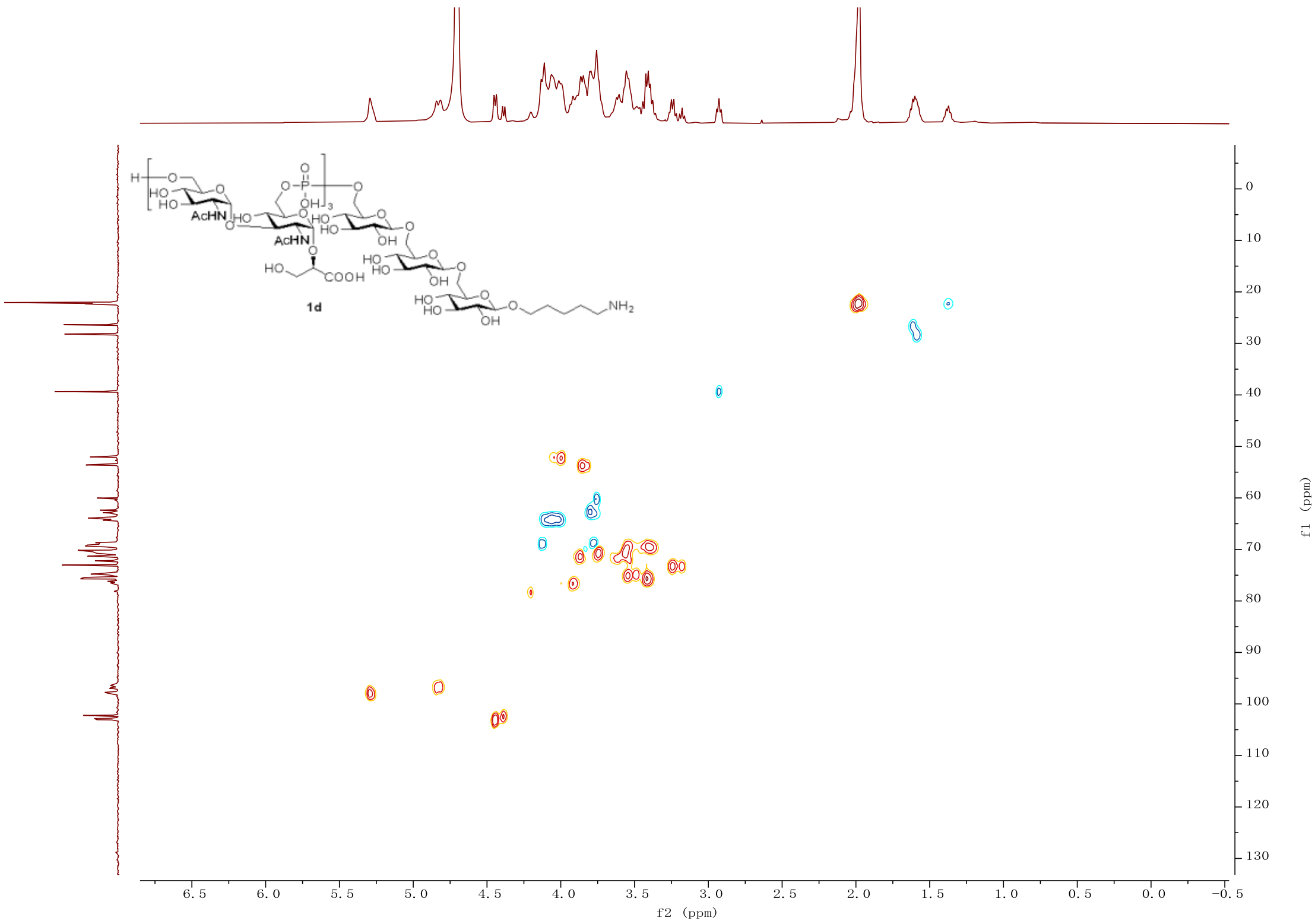

Figure S77. Compound **1d** HSQC NMR (D_2O, 600 MHz)

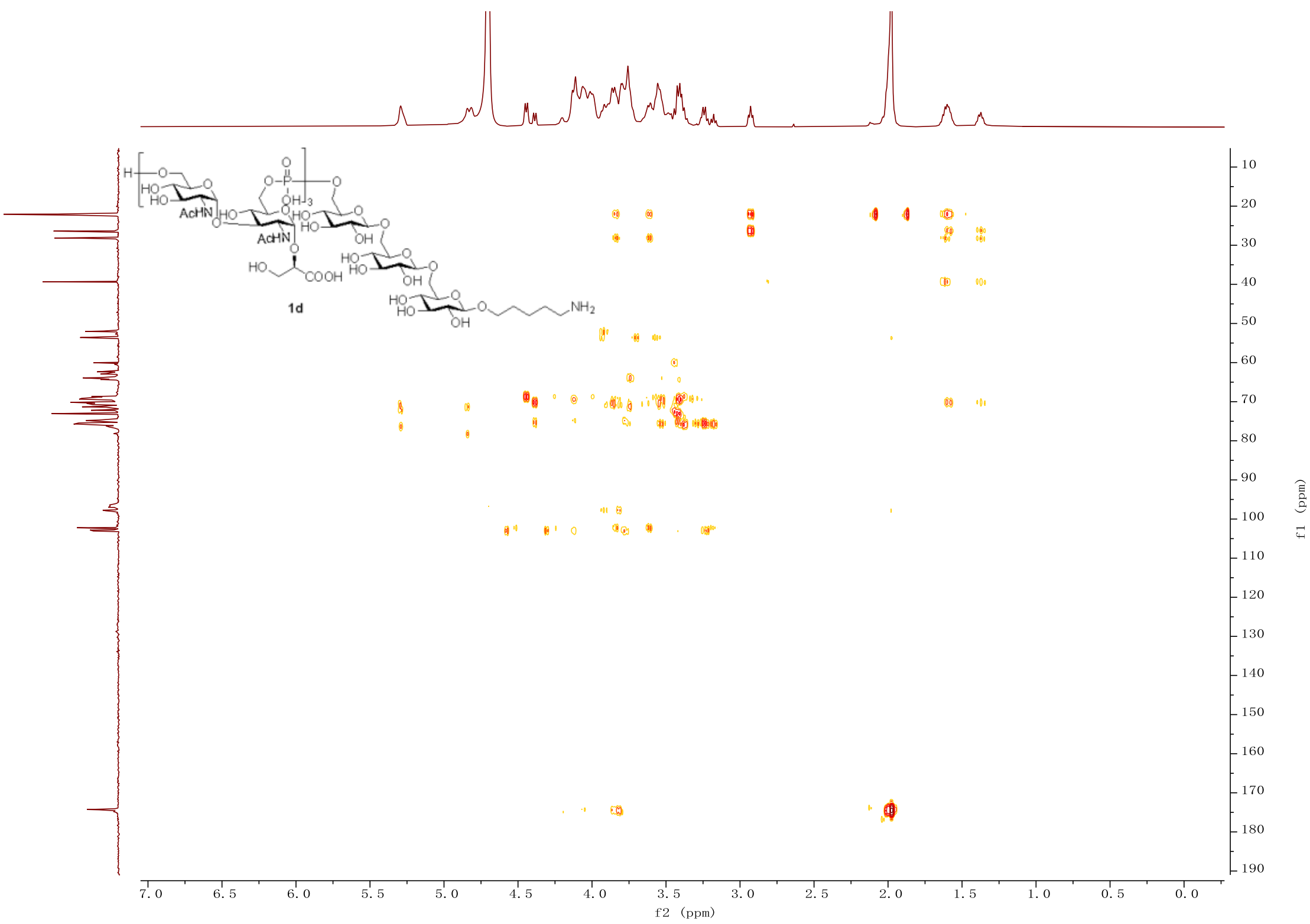

Figure S78. Compound **1d** HMBC NMR (D_2O, 600 MHz)

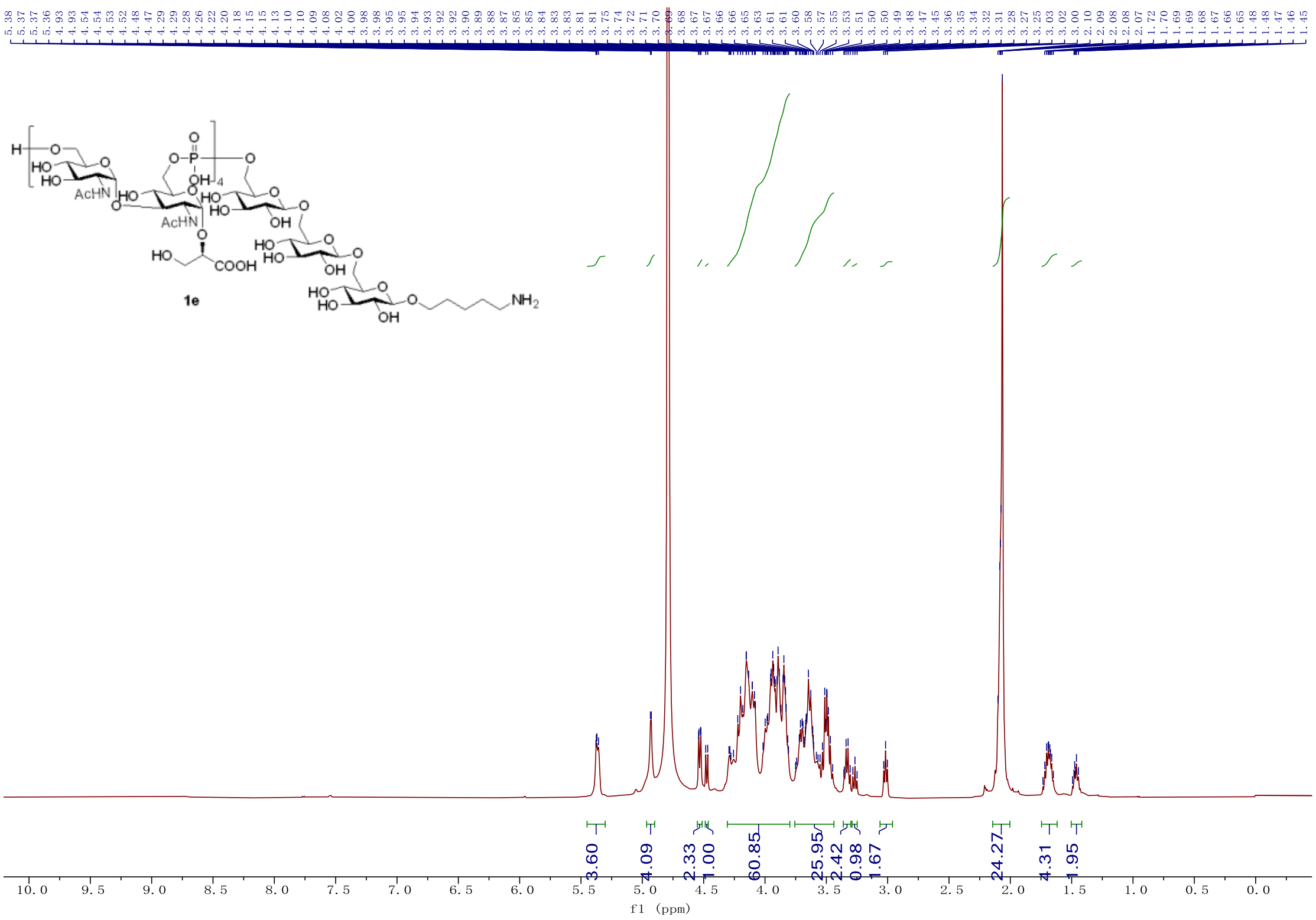

Figure S79. Compound **1e** ^{1}H NMR (D_2O, 500 MHz)

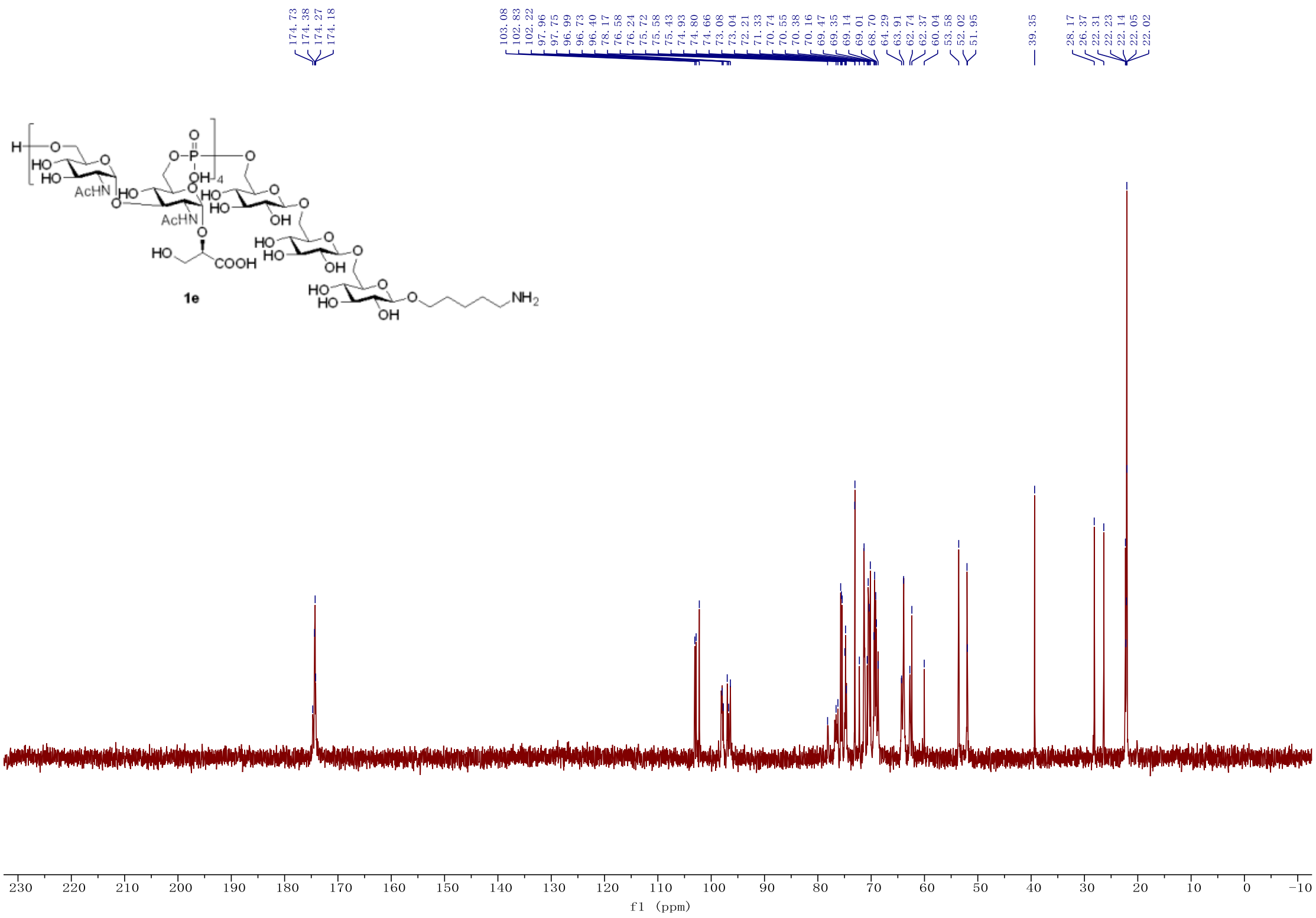

Figure S80. Compound **1e** ^{13}C NMR (D_2O, 151 MHz)

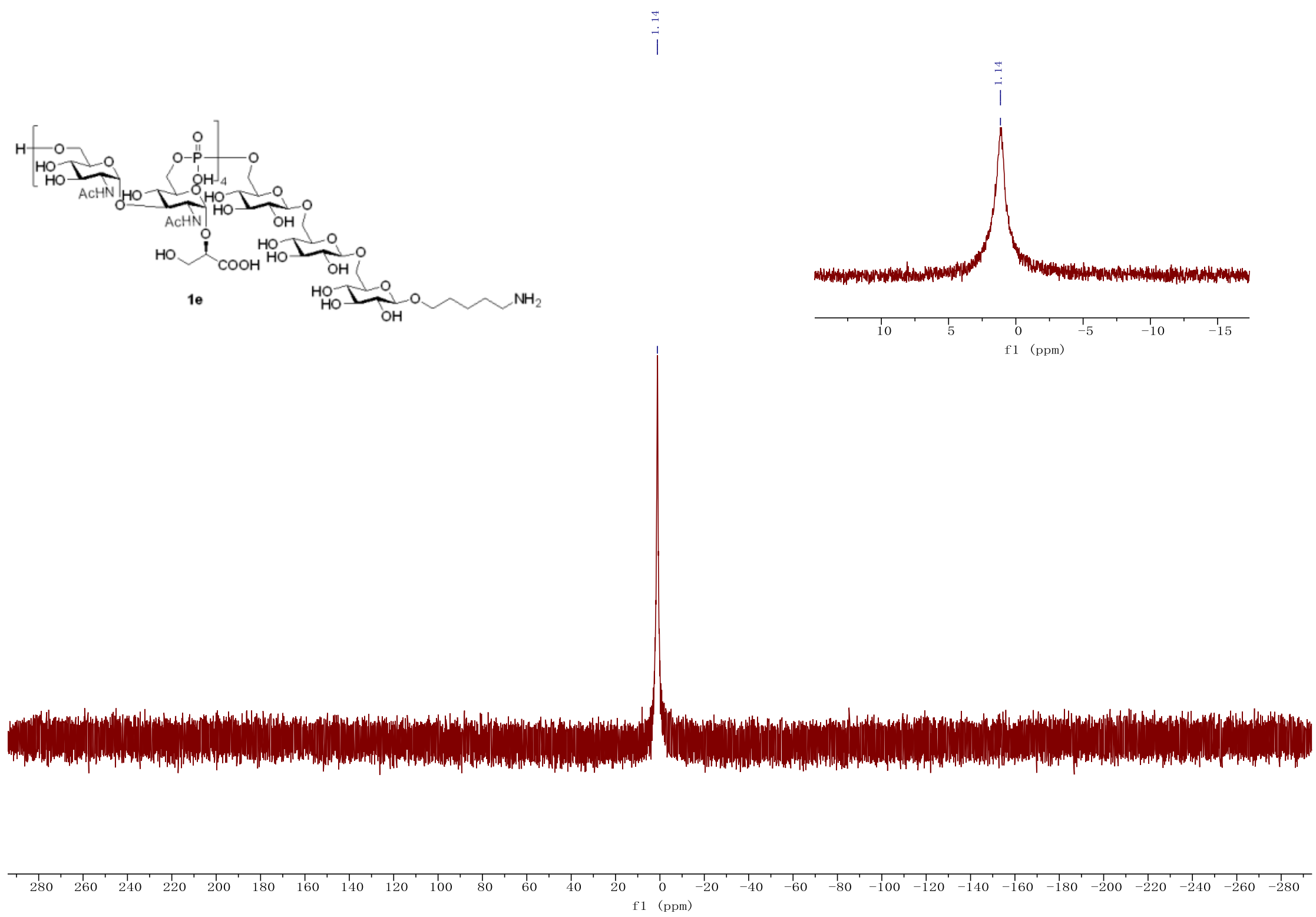

Figure S81. Compound **1e** ^{31}P NMR (D_2O, 202 MHz)

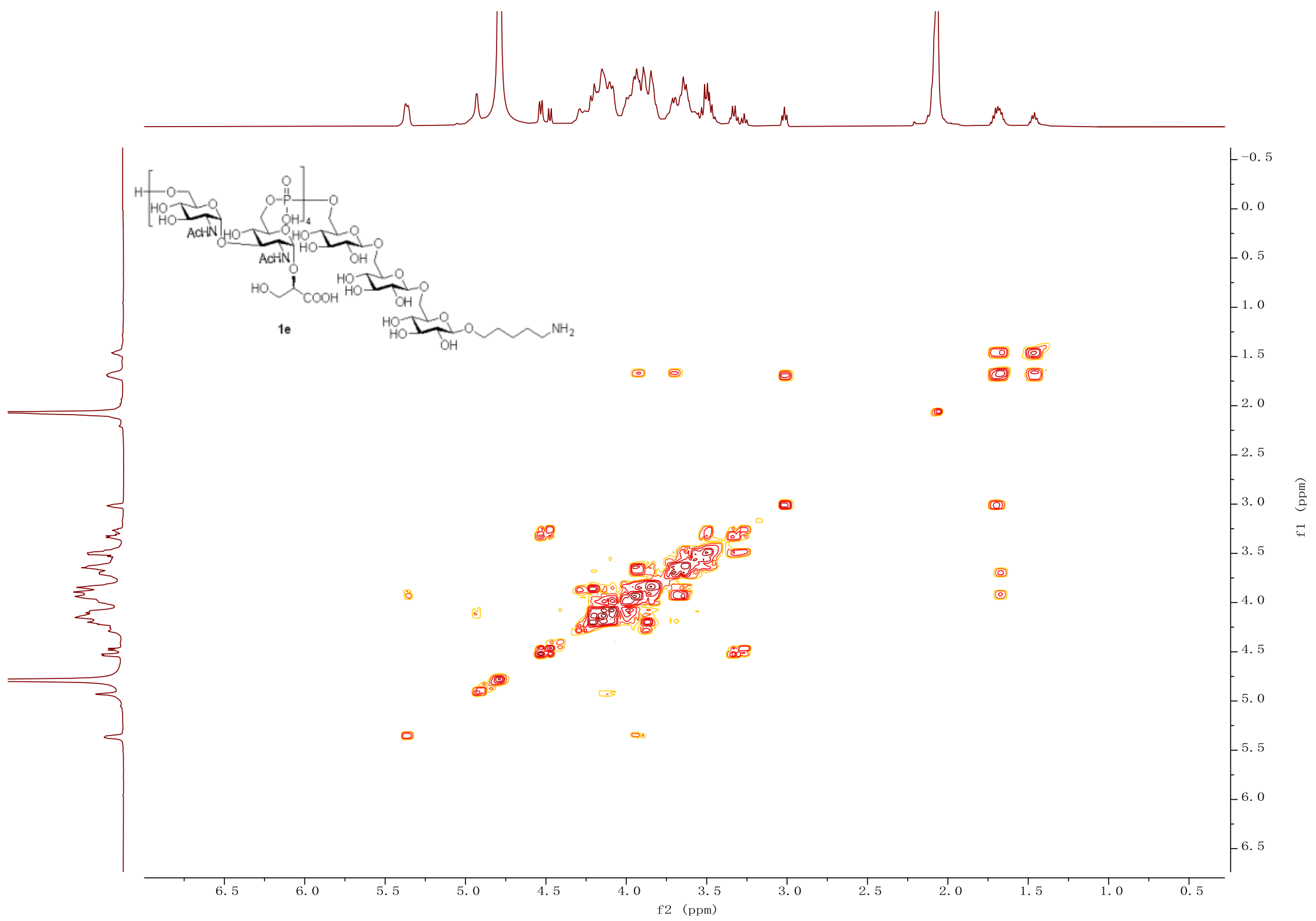

Figure S82. Compound **1e** COSY NMR (D_2O, 600 MHz)

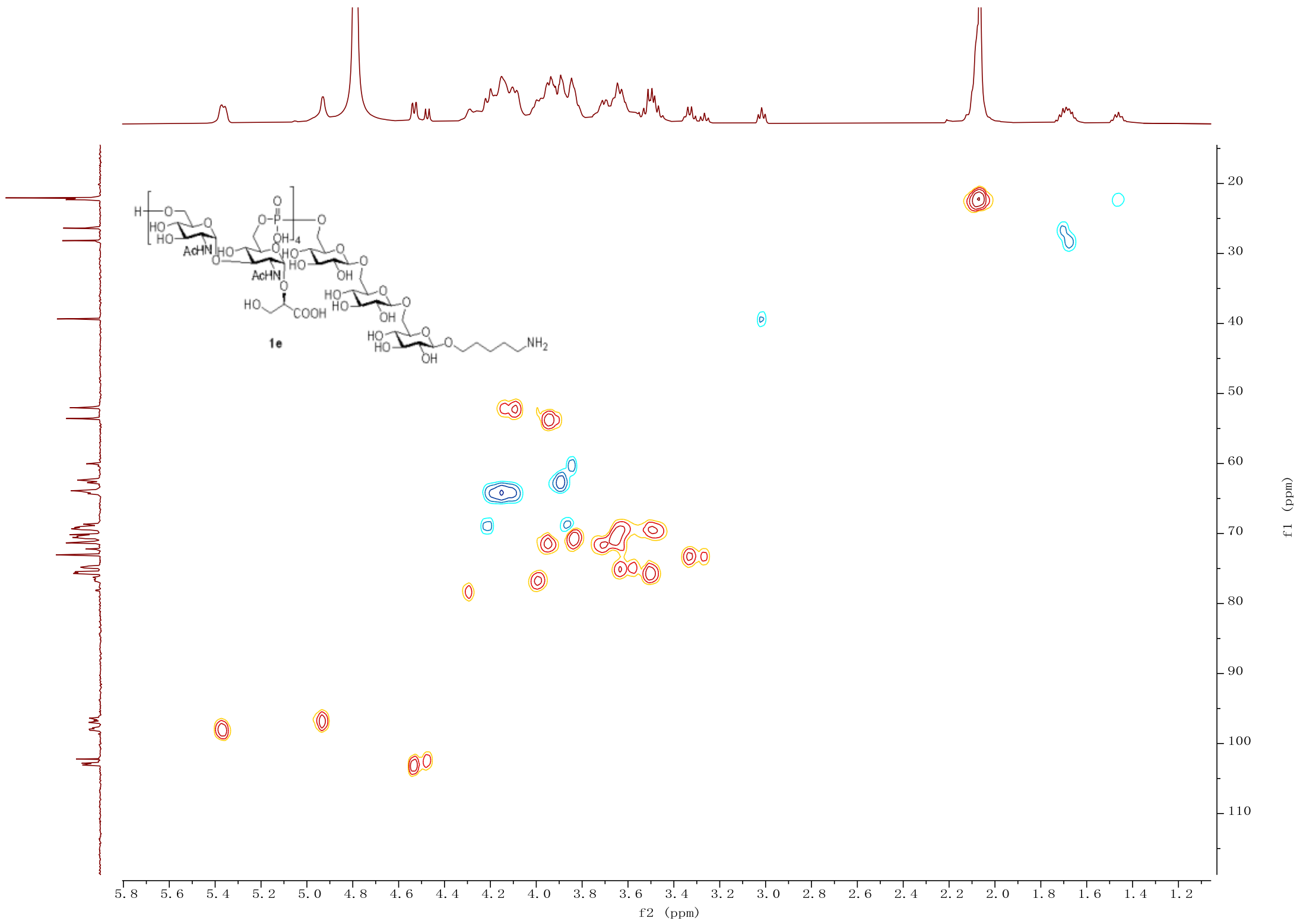

Figure S83. Compound **1e** HSQC NMR (D_2O, 600 MHz)

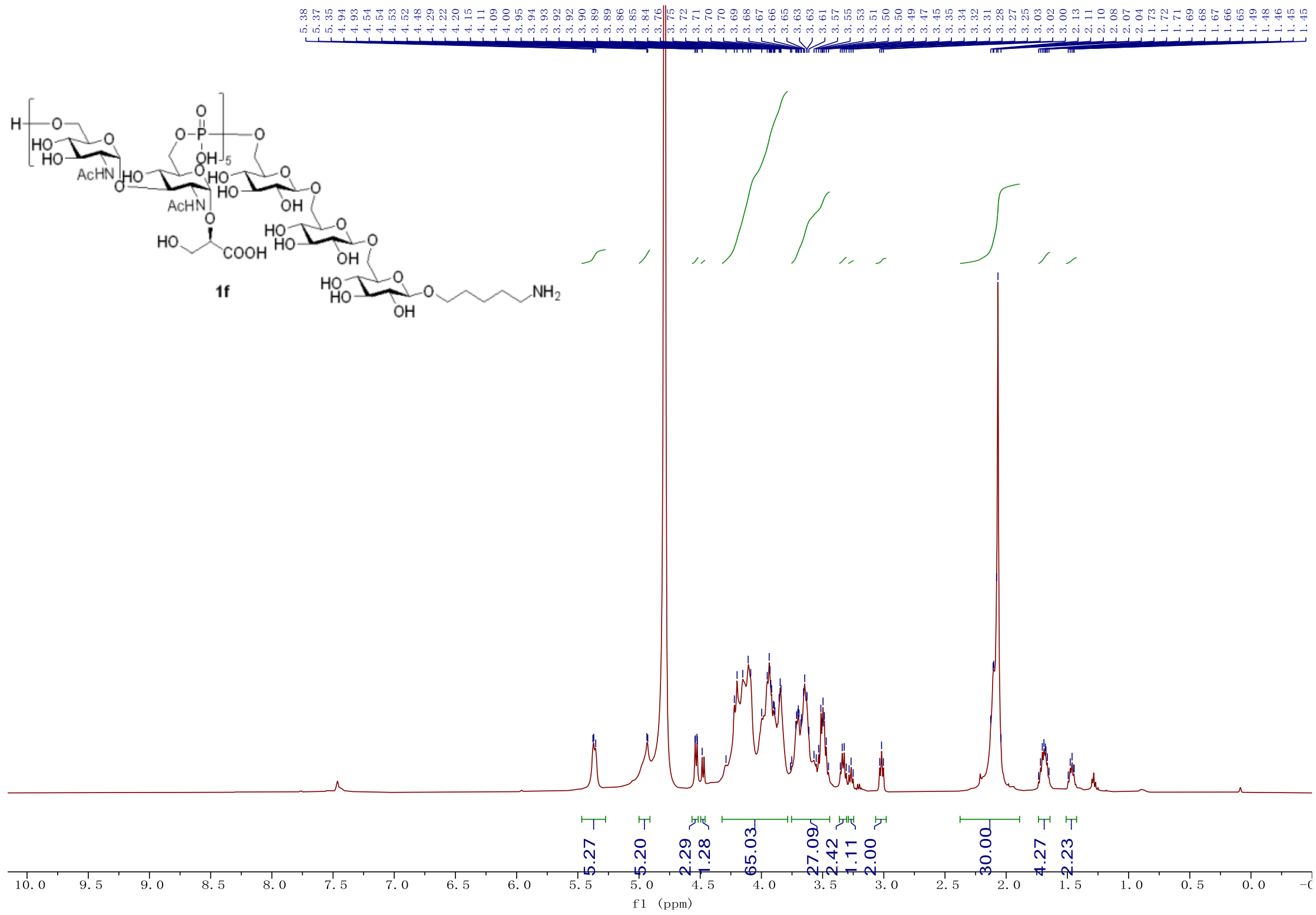

Figure S84. Compound **1f** ^1H NMR (D_2O, 600 MHz)

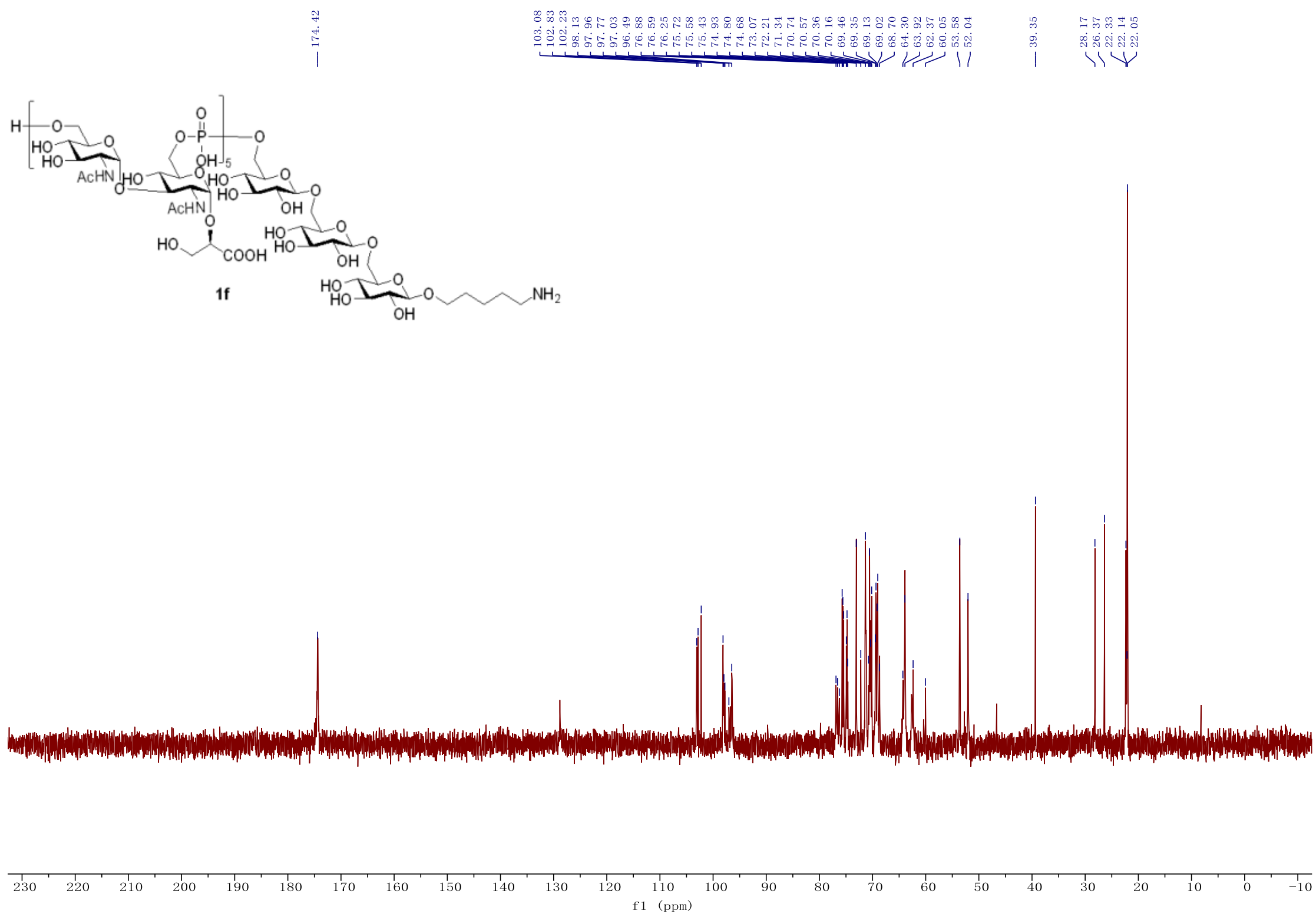

Figure S85. Compound **1f** ^{13}C NMR (D_2O, 151 MHz)

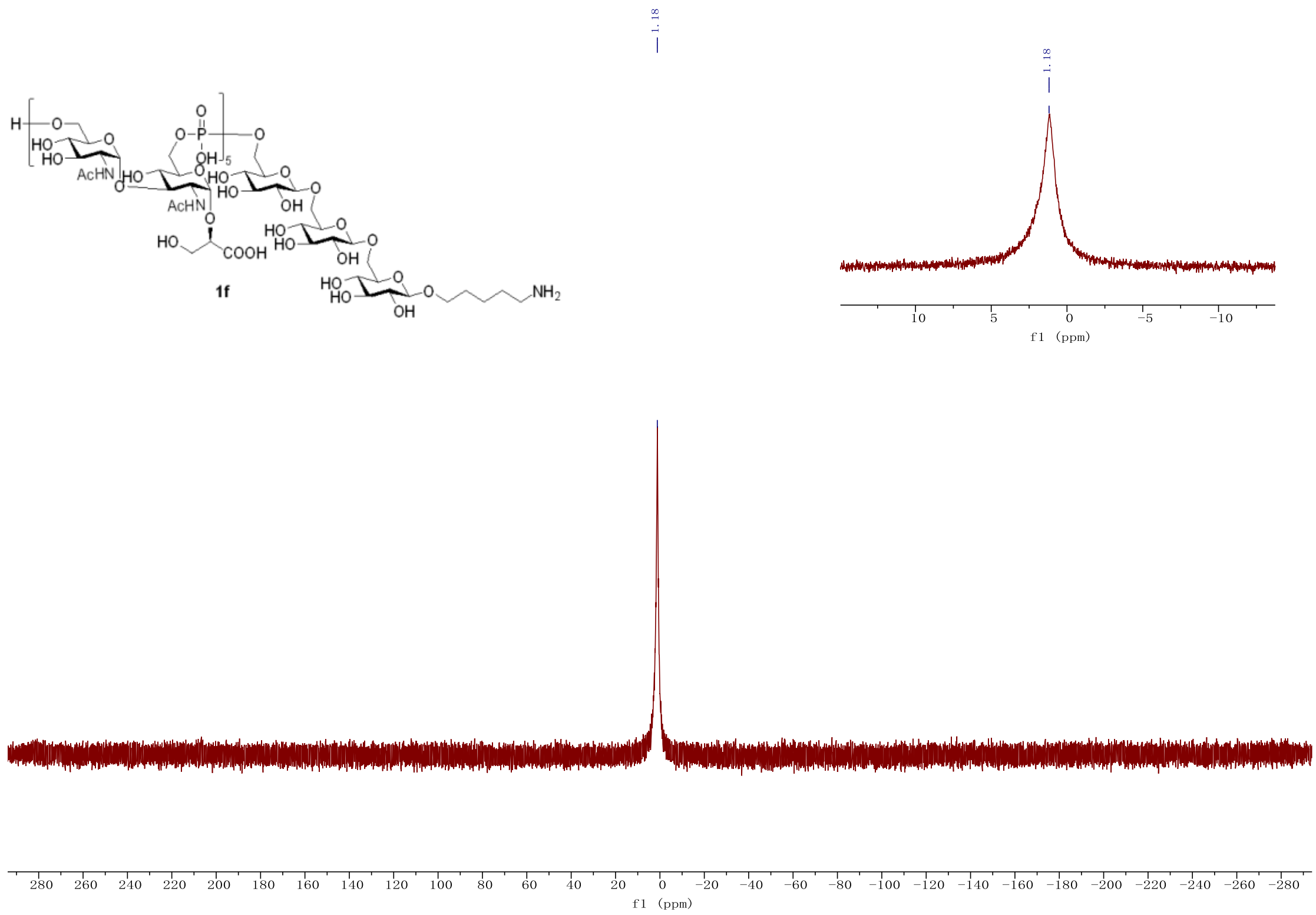

Figure S86. Compound **1f** ^{31}P NMR (D_2O, 202 MHz)

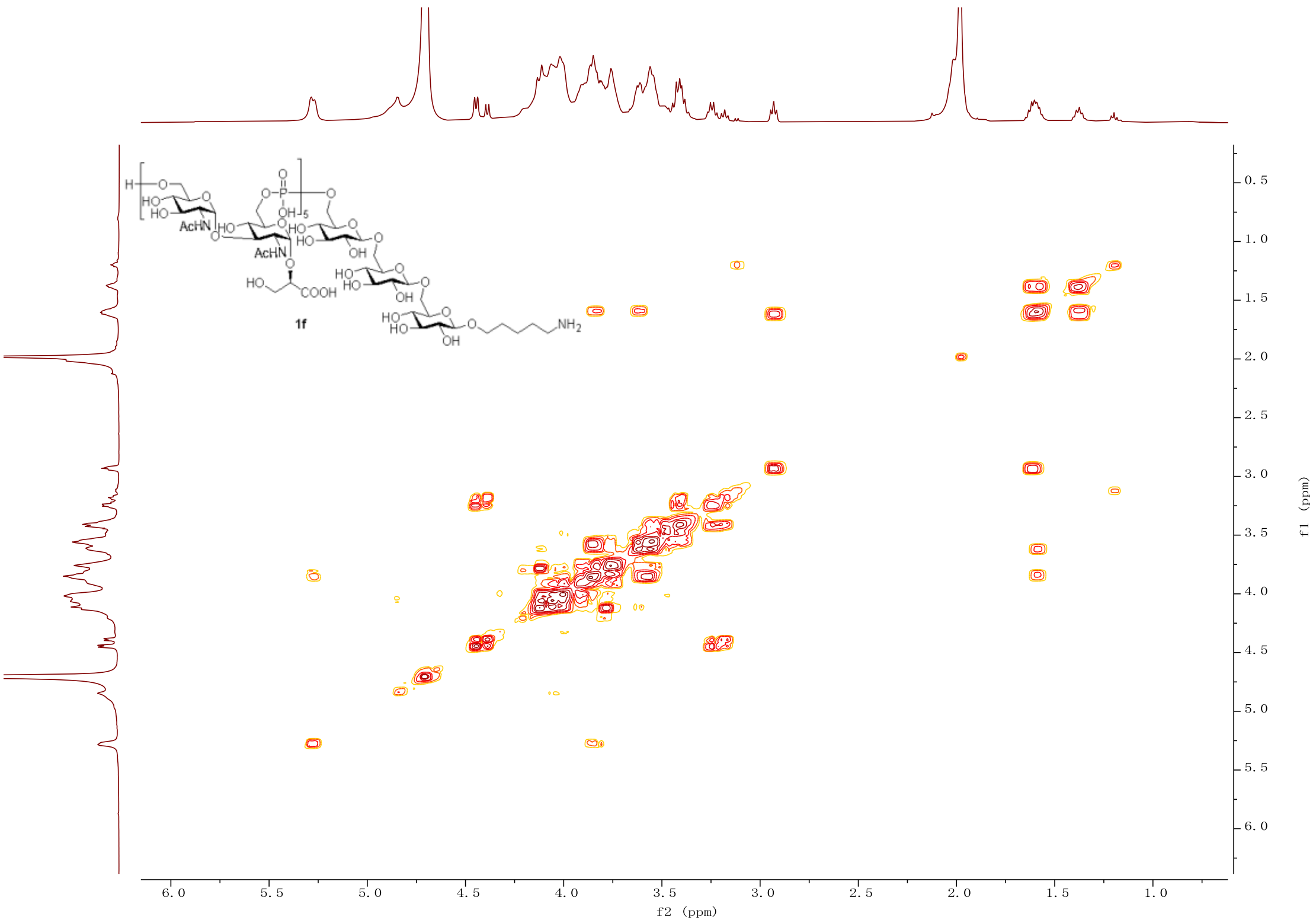

Figure S87. Compound **1f** COSY NMR (D_2O, 600 MHz)

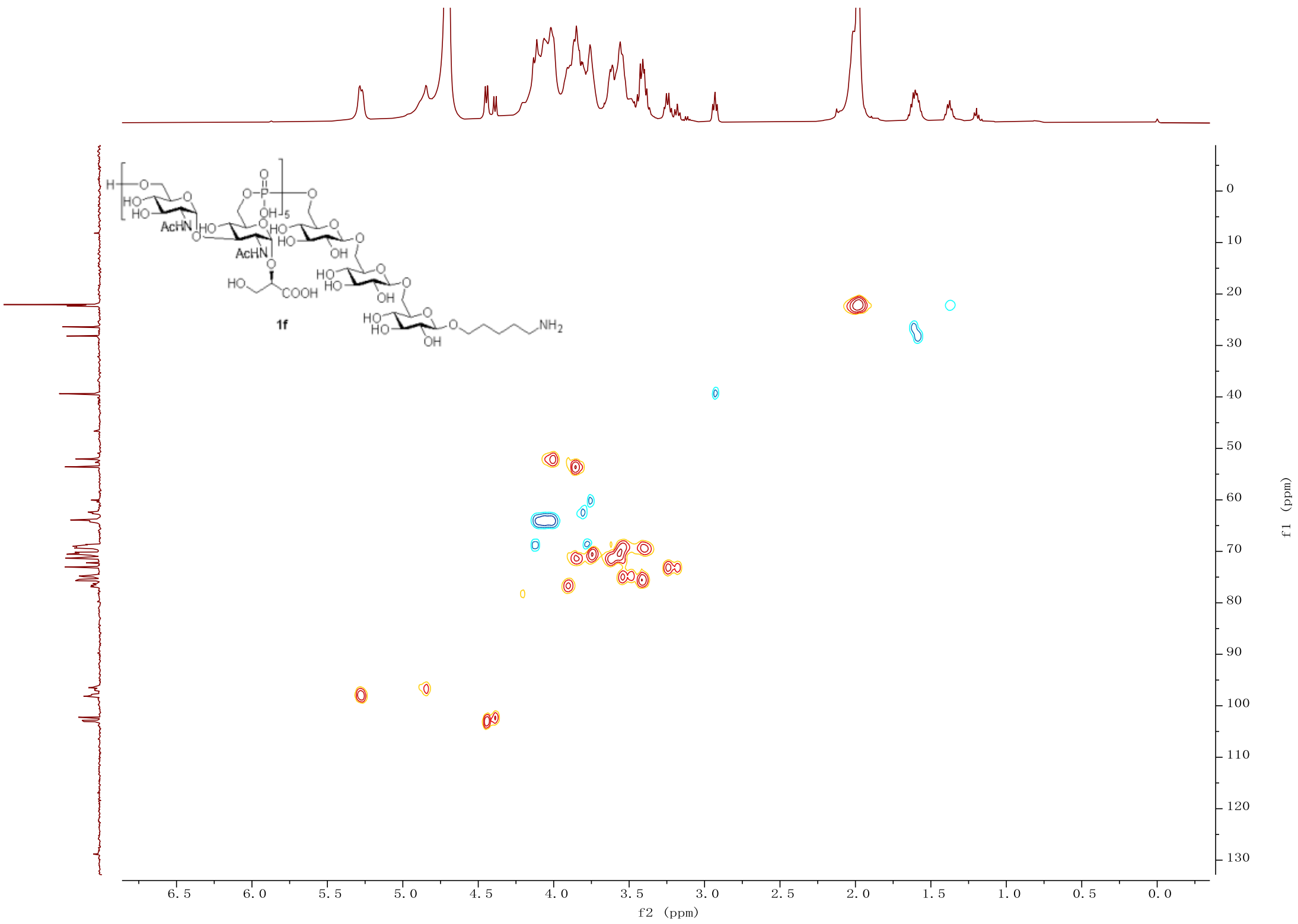

Figure S88. Compound **1f** HSQC NMR (D_2O, 600 MHz)

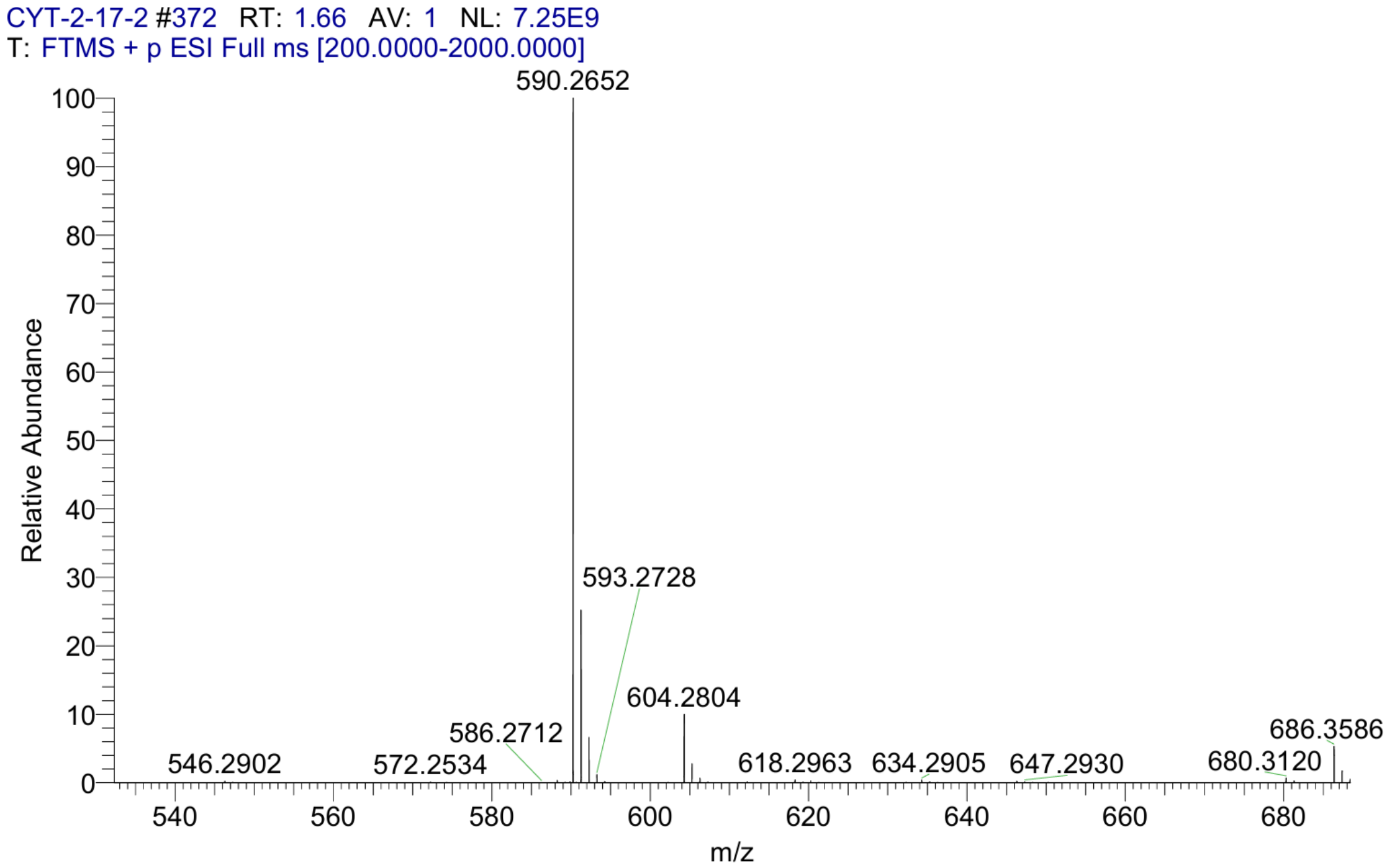

Figure S89. HRMS data of compound **1a**

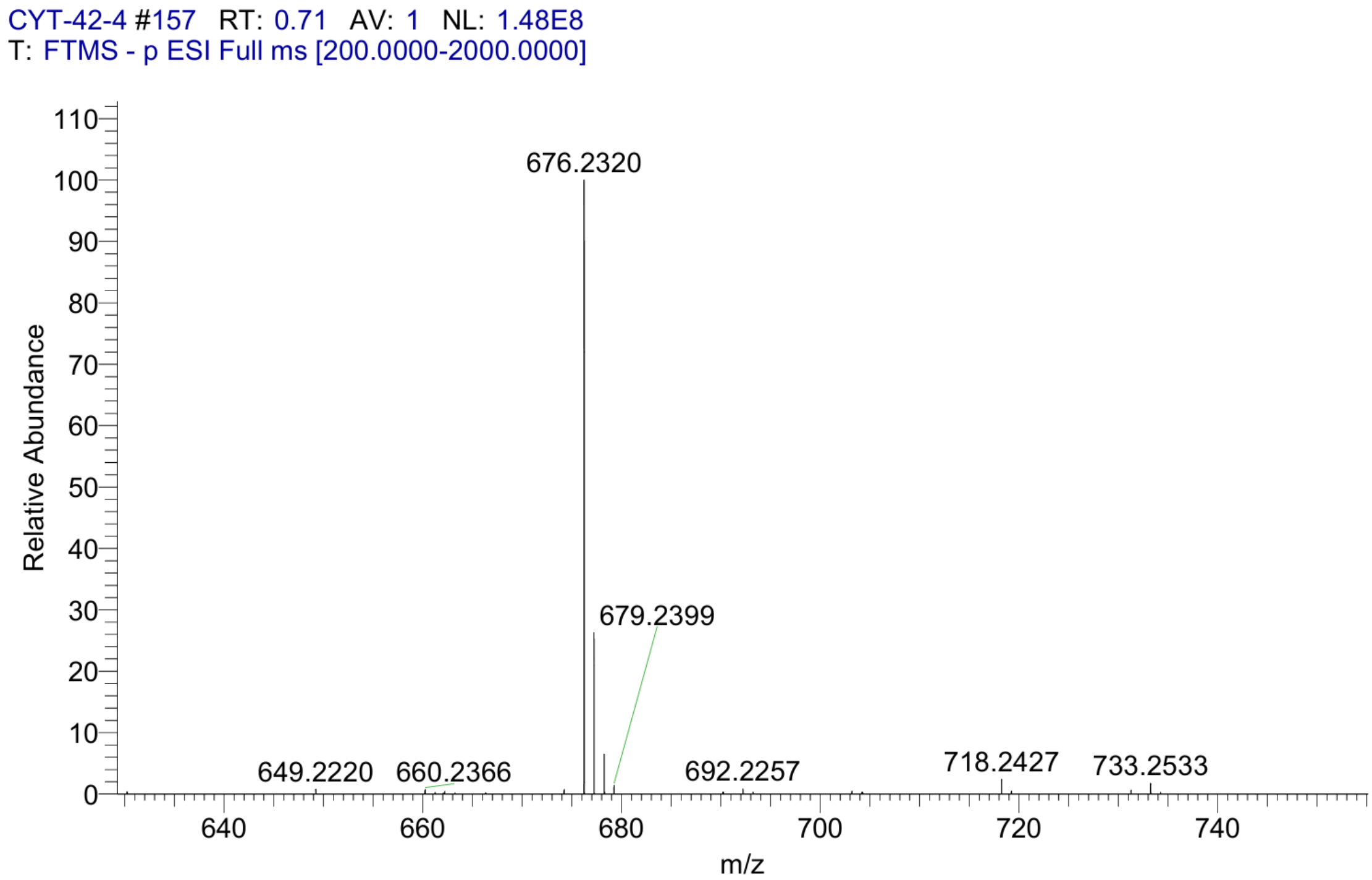

Figure S90. HRMS data of compound **24a**

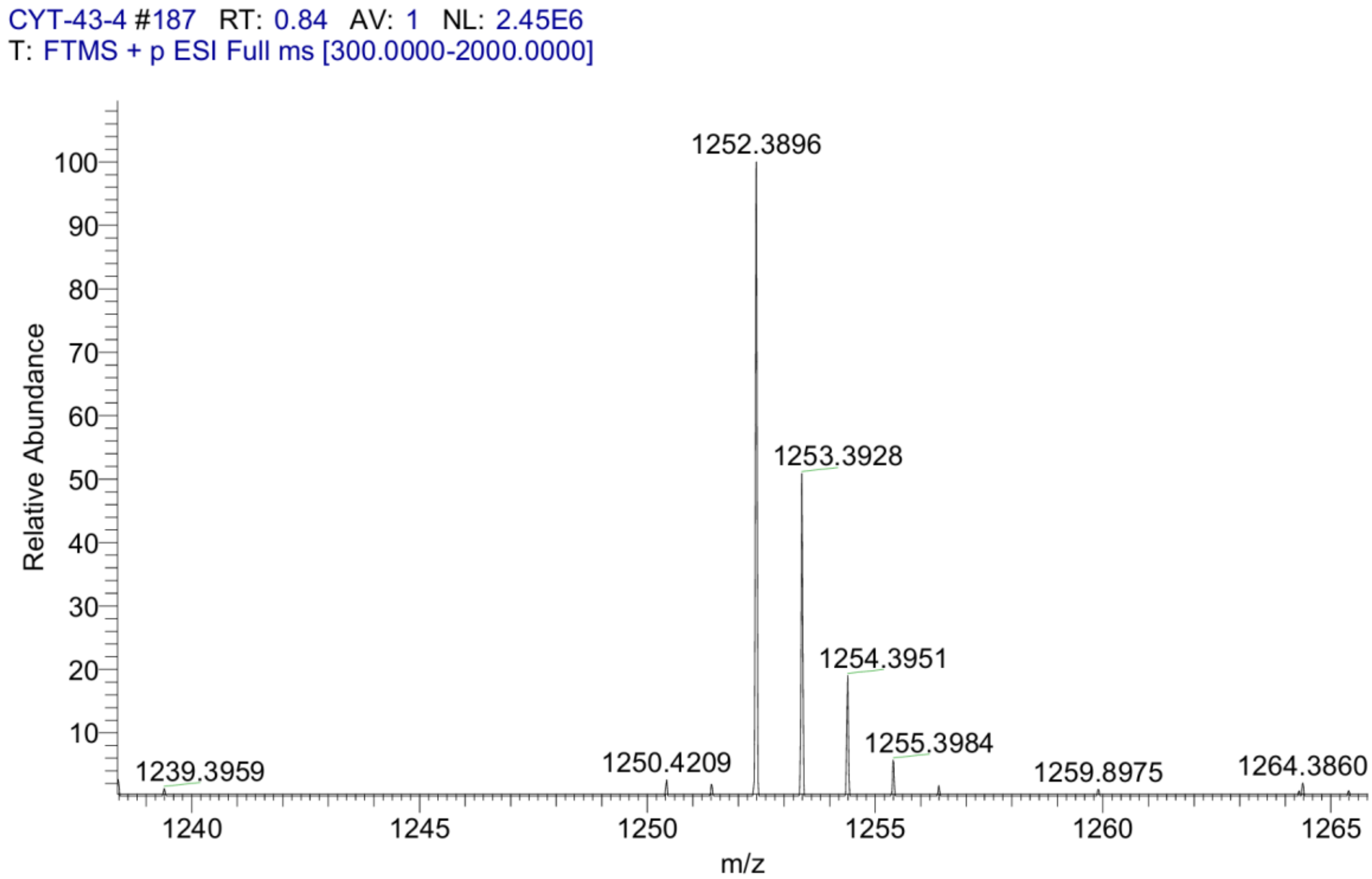

Figure S91. HRMS data of compound **24b**

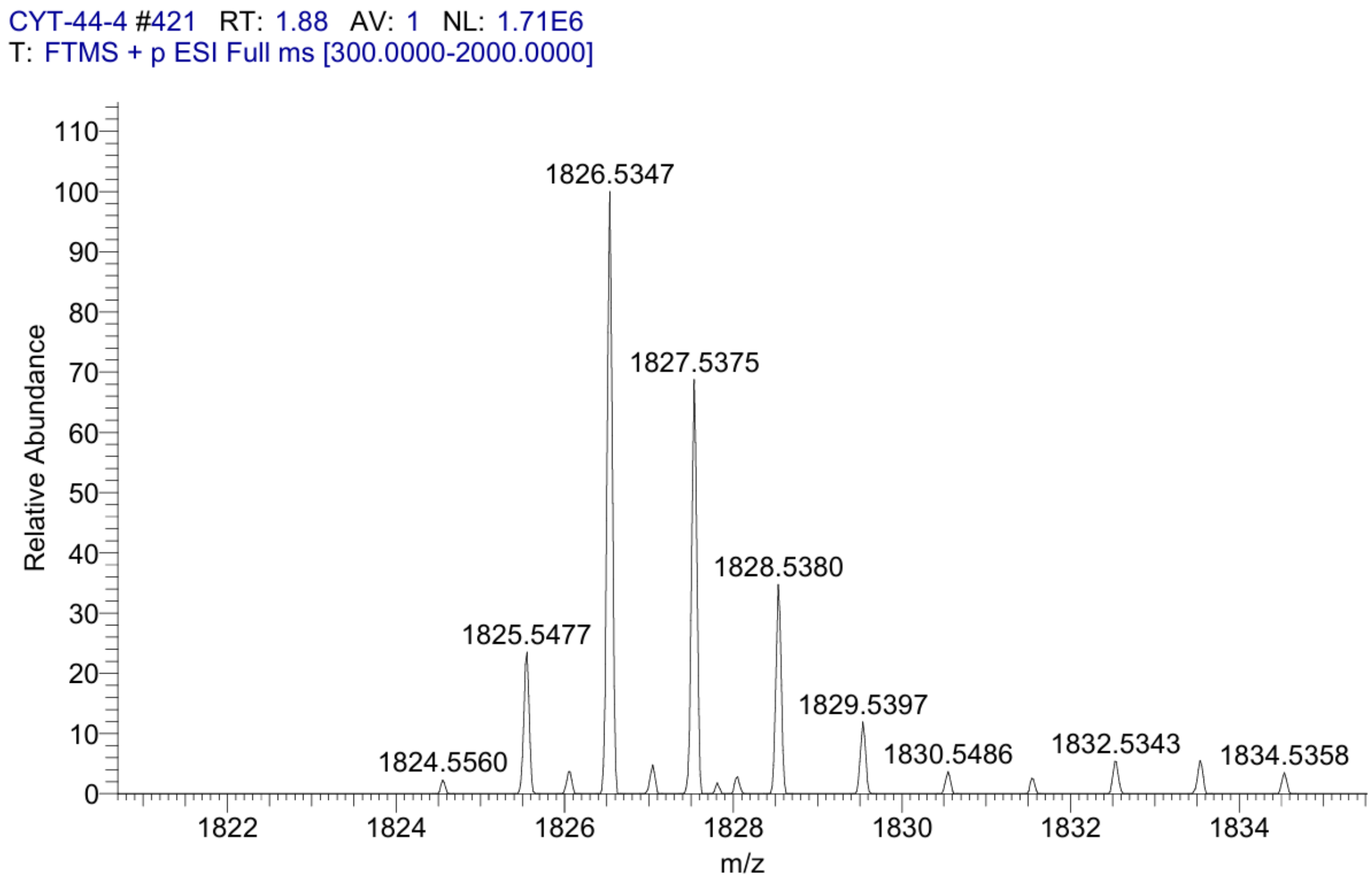

Figure S92. HRMS data of compound **24c**

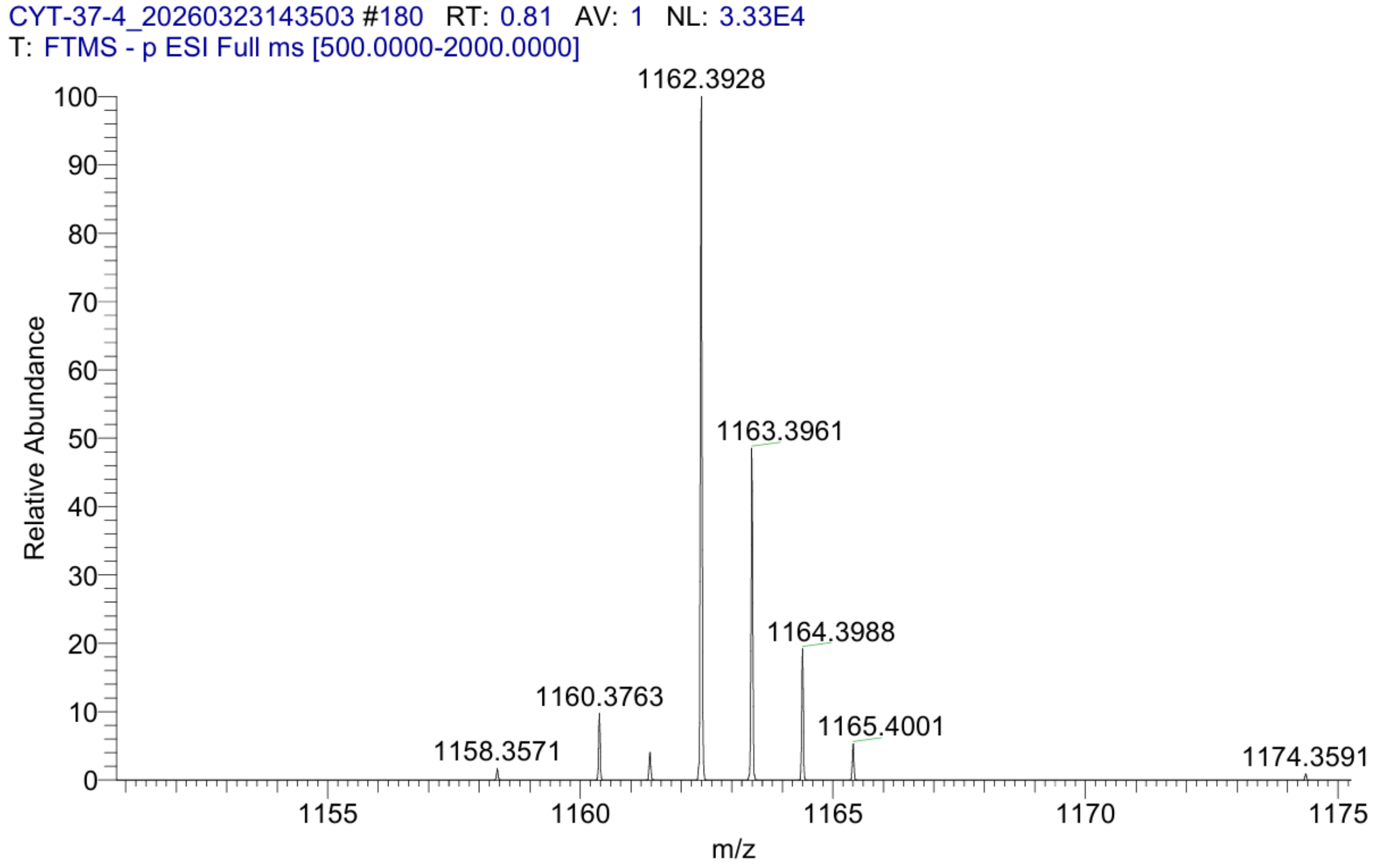

Figure S93. HRMS data of compound **1b**

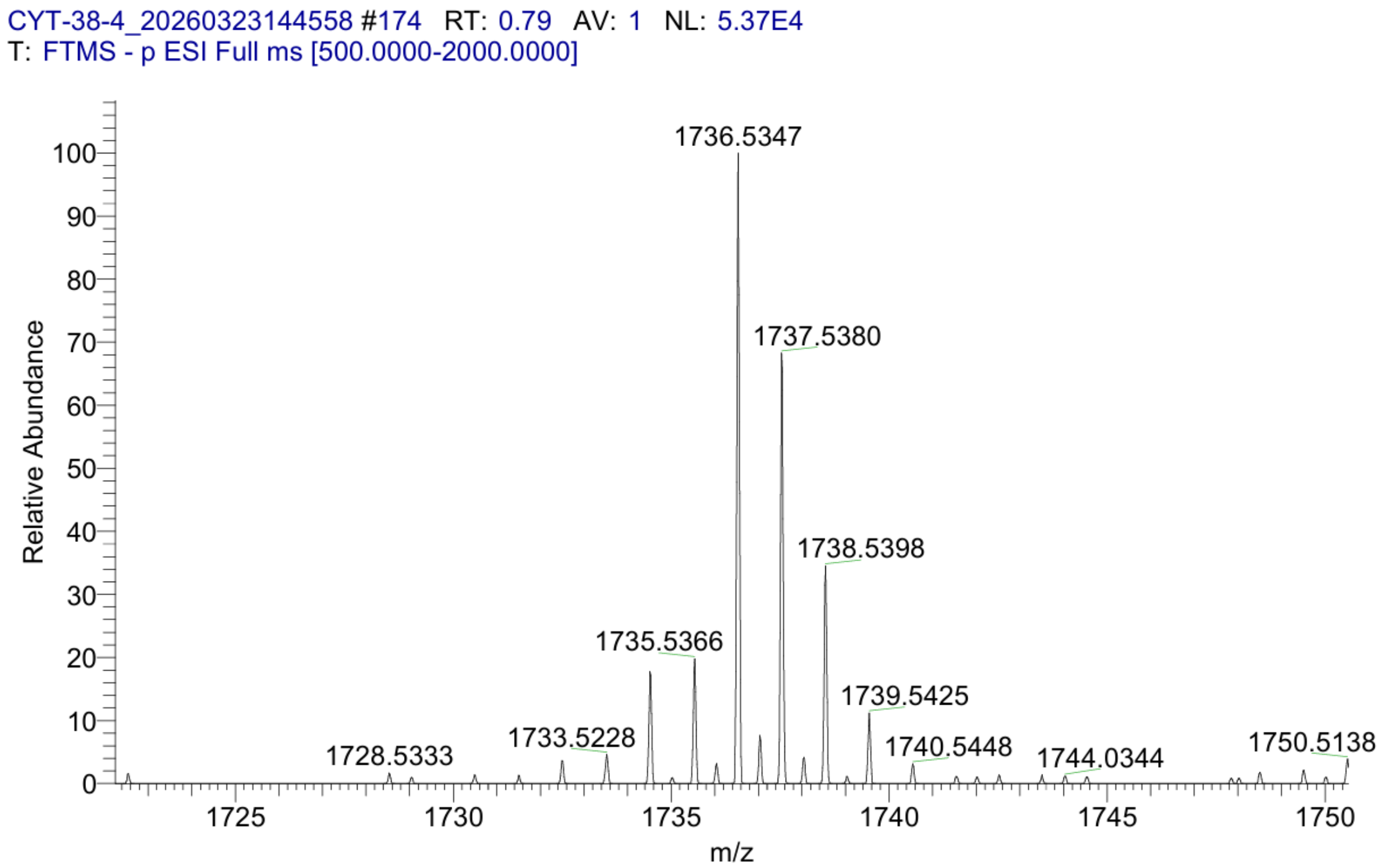

Figure S94. HRMS data of compound **1c**

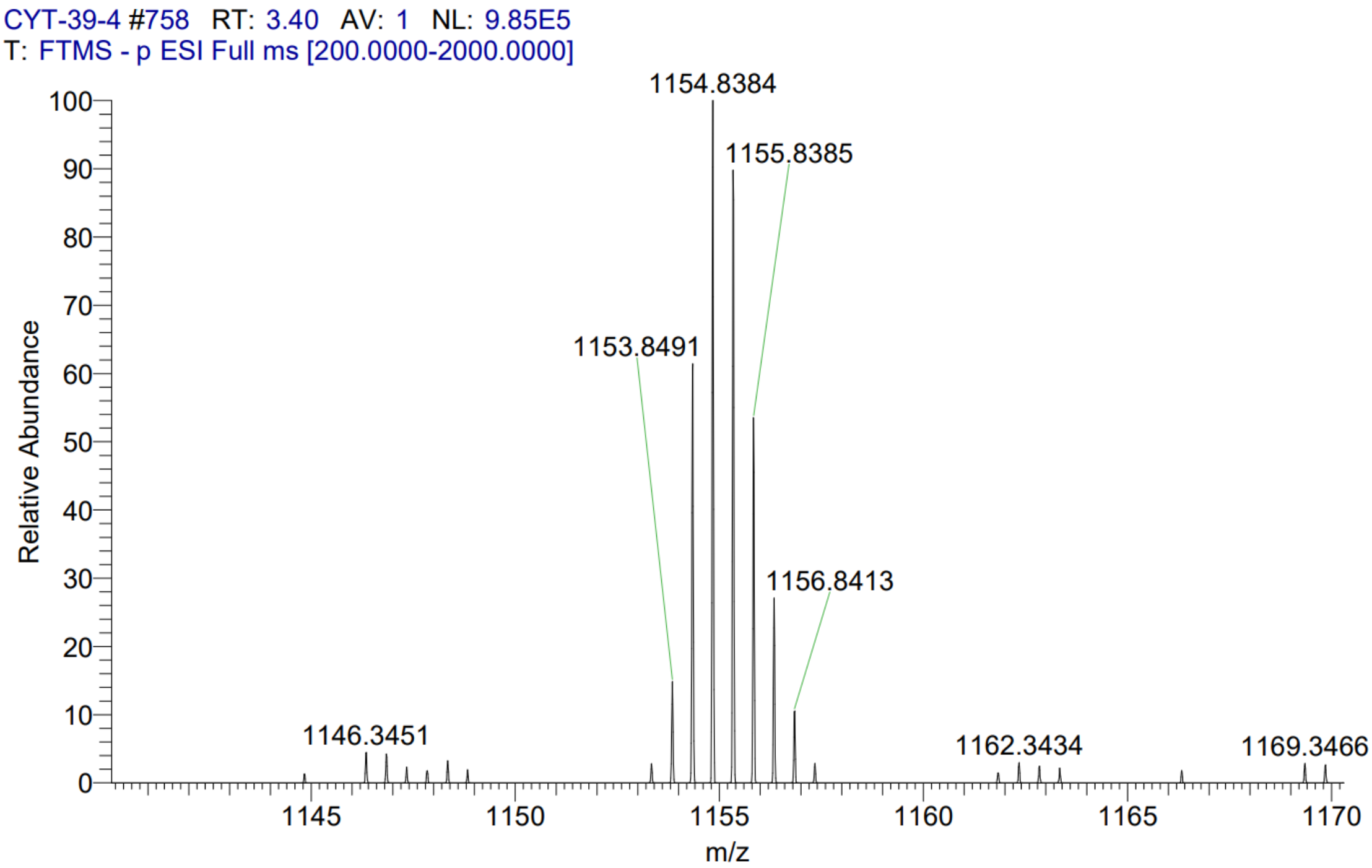

Figure S95. HRMS data of compound **1d**

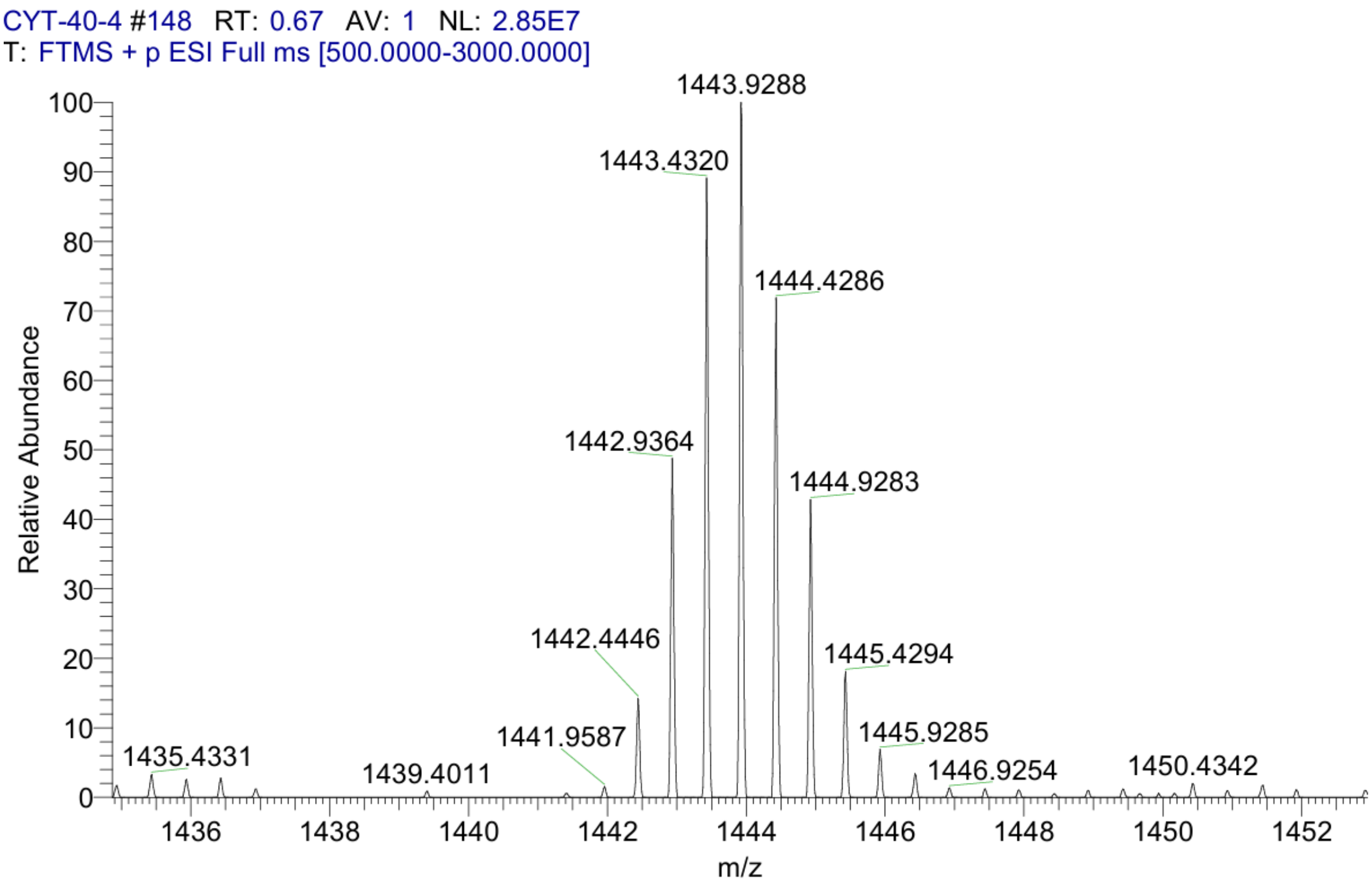

Figure S96. HRMS data of compound **1e**

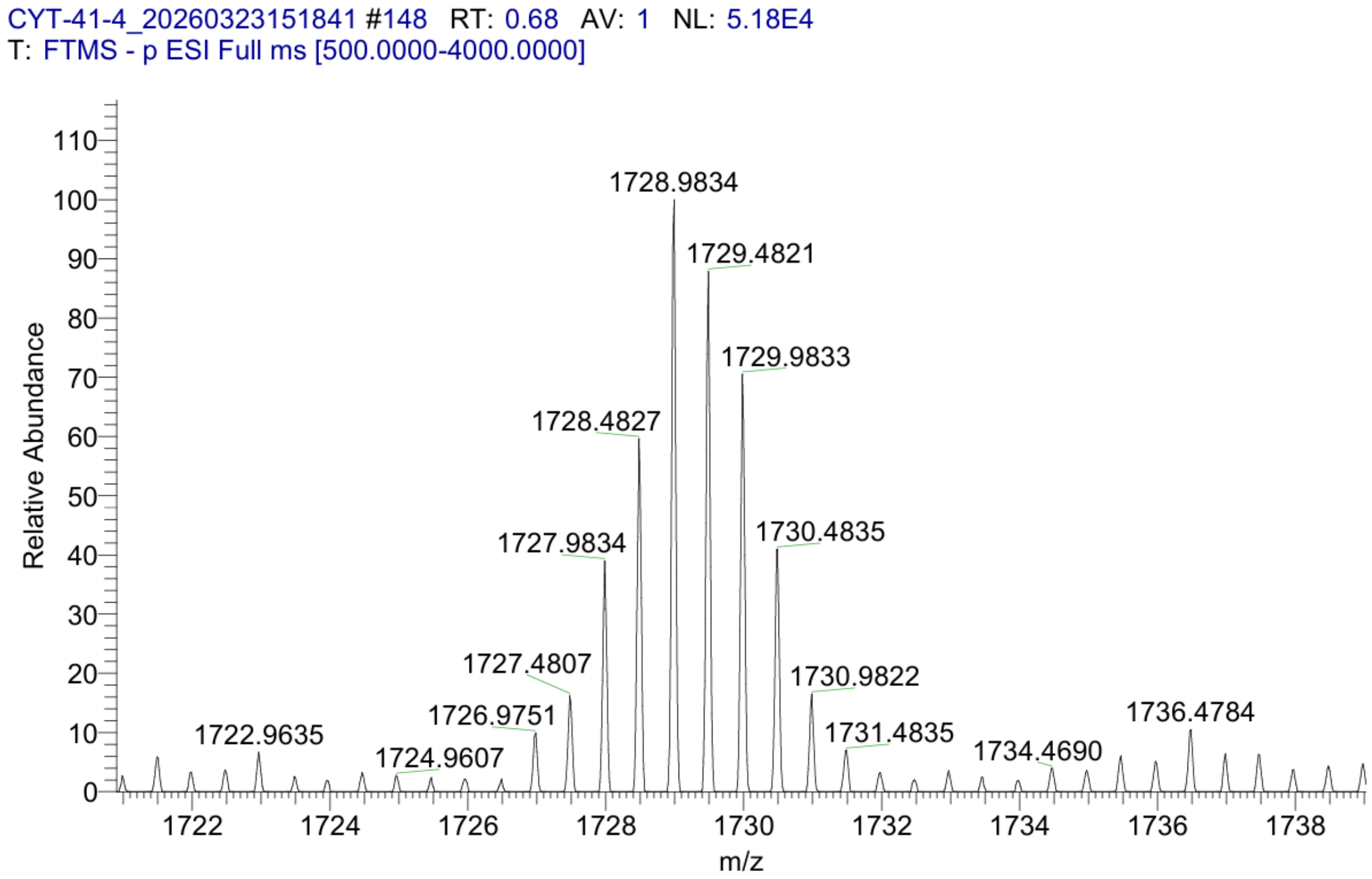

Figure S97. HRMS data of compound **1f**
